# Supplementary material for: Long Intergenic Non-Coding RNAs of Human Chromosome 18: Focus on Cancers
Source: Biomedicines. 2024 Feb 28;12(3):544. doi: 10.3390/biomedicines12030544 (PMC10968066; doi:10.3390/biomedicines12030544)
Supplement: Supplementary file 1 [file biomedicines-12-00544-s001.zip › Figure_S1_Ershov.pdf]

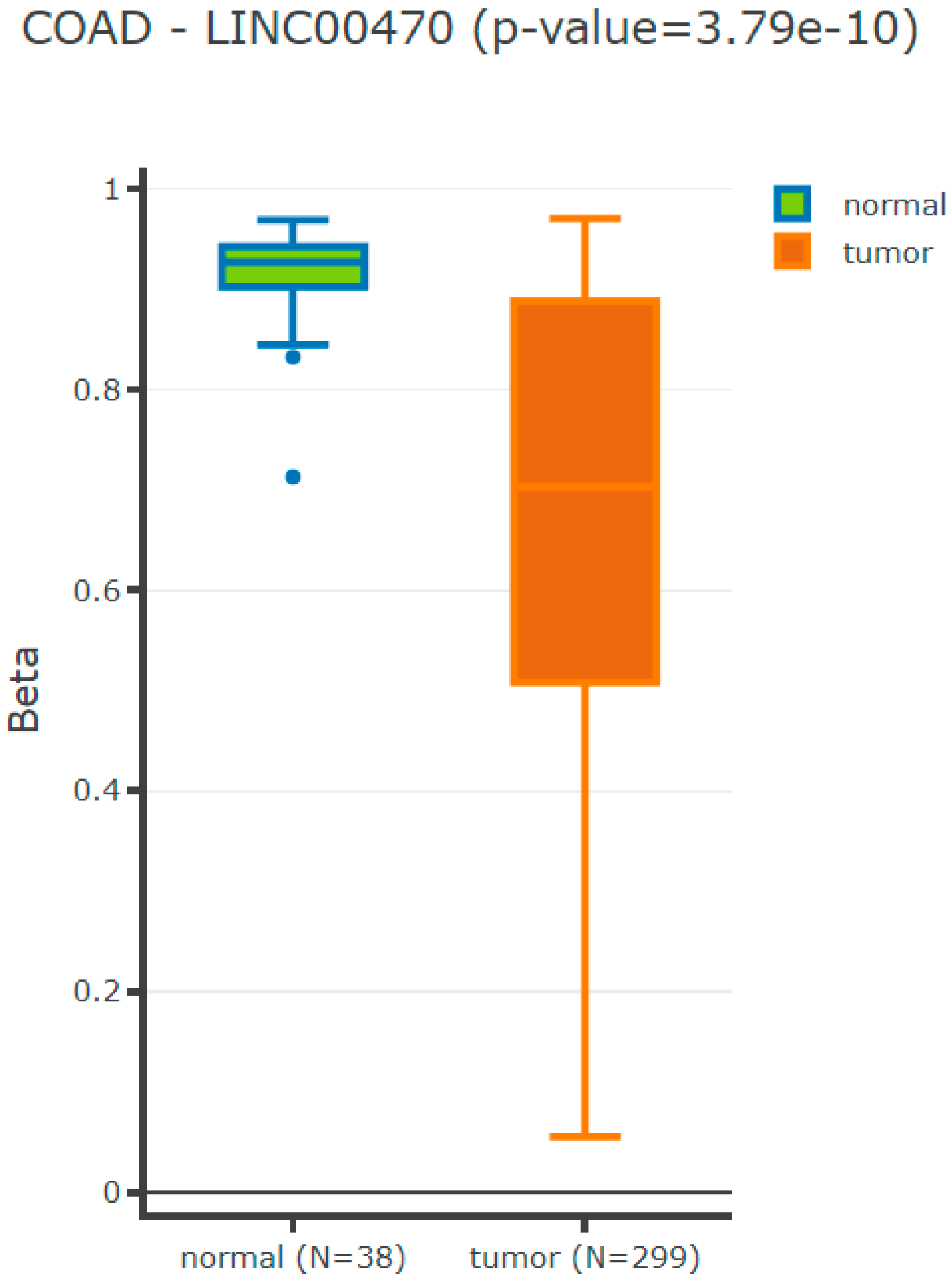

Figure S1-A. Differentially (tumor/normal tissues) methylated promoter region of *LINC00470* found in COAD cohort: |beta-value difference| > 0.2 and independent Student's t-test adjusted p-value ≤ 0.05; hypermethylation and hypomethylation, beta-values > 0.2 and < – 0.2 at adj. p-value ≤ 0.05, respectively). Differentially methylated genes were explored using DNMIVD: DNA Methylation Interactive Visualization Database [S1].

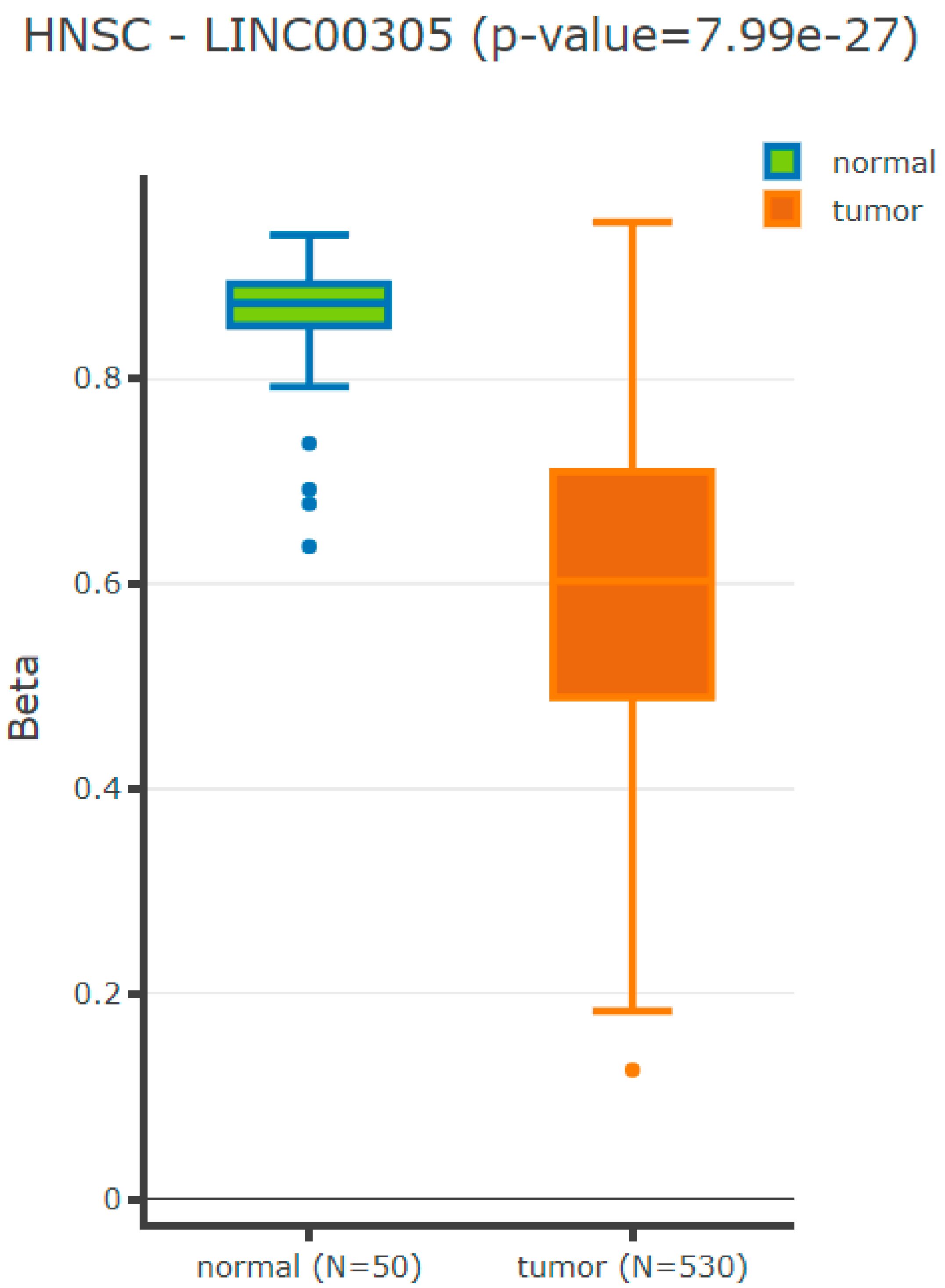

Figure S1-B. Differentially (tumor/normal tissues) methylated promoter region of *LINC00305* found in HNSC cohort: |beta-value difference| > 0.2 and independent Student's t-test adjusted p-value ≤ 0.05; hypermethylation and hypomethylation, beta-values > 0.2 and < – 0.2 at adj. p-value ≤ 0.05, respectively). Differentially methylated genes were explored using DNMIVD: DNA Methylation Interactive Visualization Database [S1].

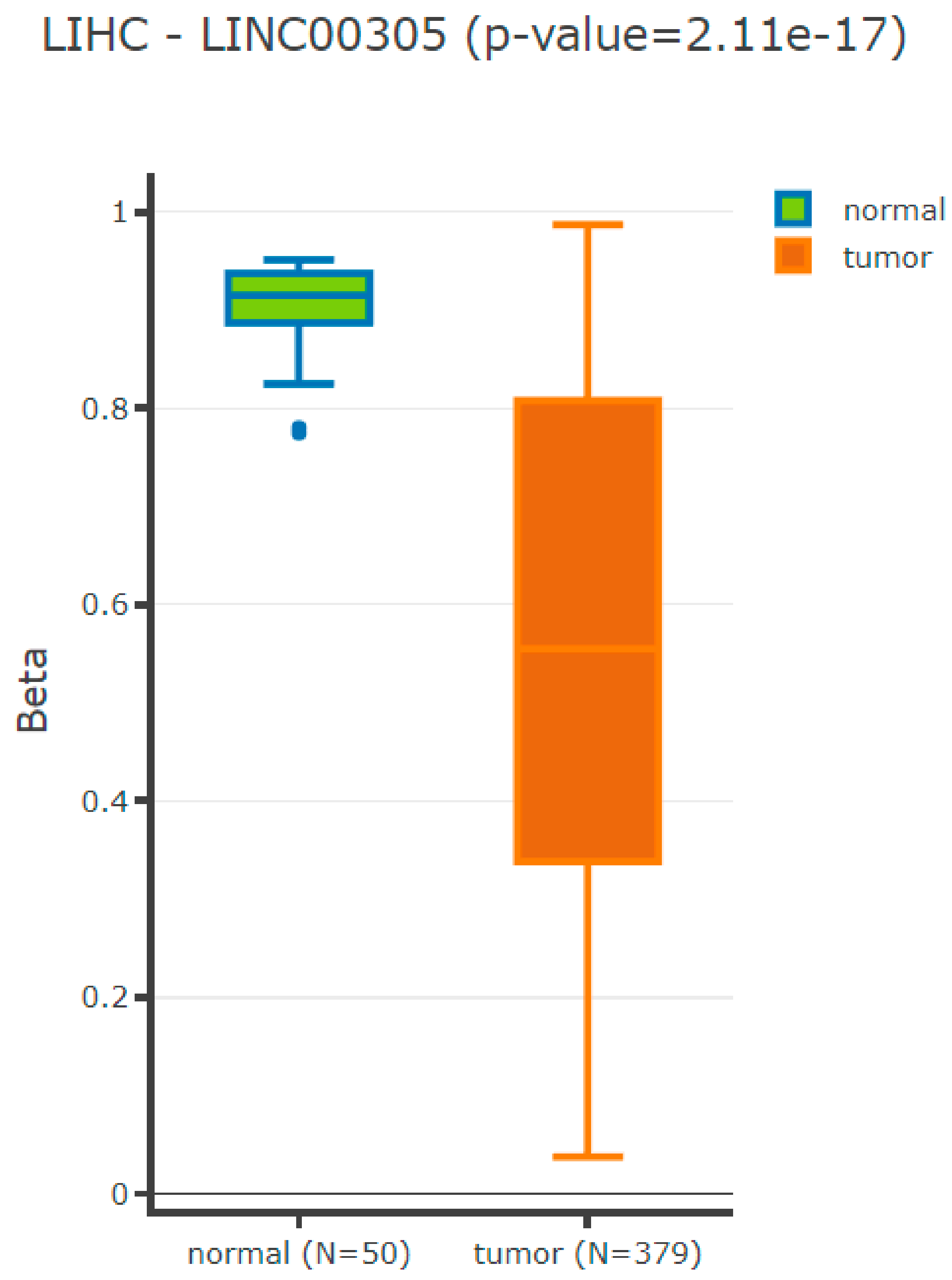

Figure S1-C. Differentially (tumor/normal tissues) methylated promoter region of *LINC00305* found in LIHC cohort: |beta-value difference| > 0.2 and independent Student's t-test adjusted p-value ≤ 0.05; hypermethylation and hypomethylation, beta-values > 0.2 and < – 0.2 at adj. p-value ≤ 0.05, respectively). Differentially methylated genes were explored using DNMIVD: DNA Methylation Interactive Visualization Database [S1].

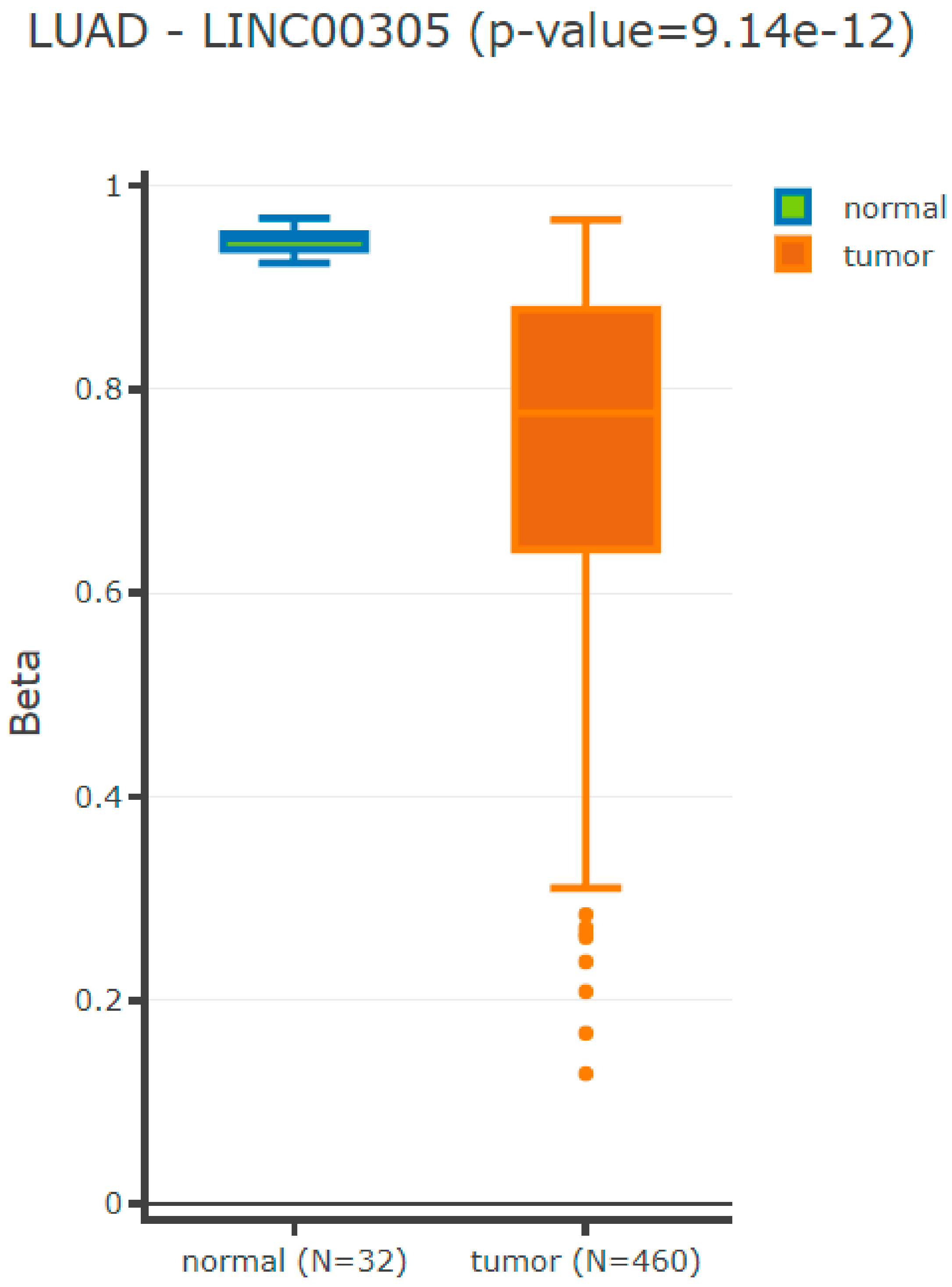

Figure S1-D. Differentially (tumor/normal tissues) methylated promoter region of *LINC00305* found in LUAD cohort: |beta-value difference| > 0.2 and independent Student's t-test adjusted p-value ≤ 0.05; hypermethylation and hypomethylation, beta-values > 0.2 and < – 0.2 at adj. p-value ≤ 0.05, respectively). Differentially methylated genes were explored using DNMIVD: DNA Methylation Interactive Visualization Database [S1].

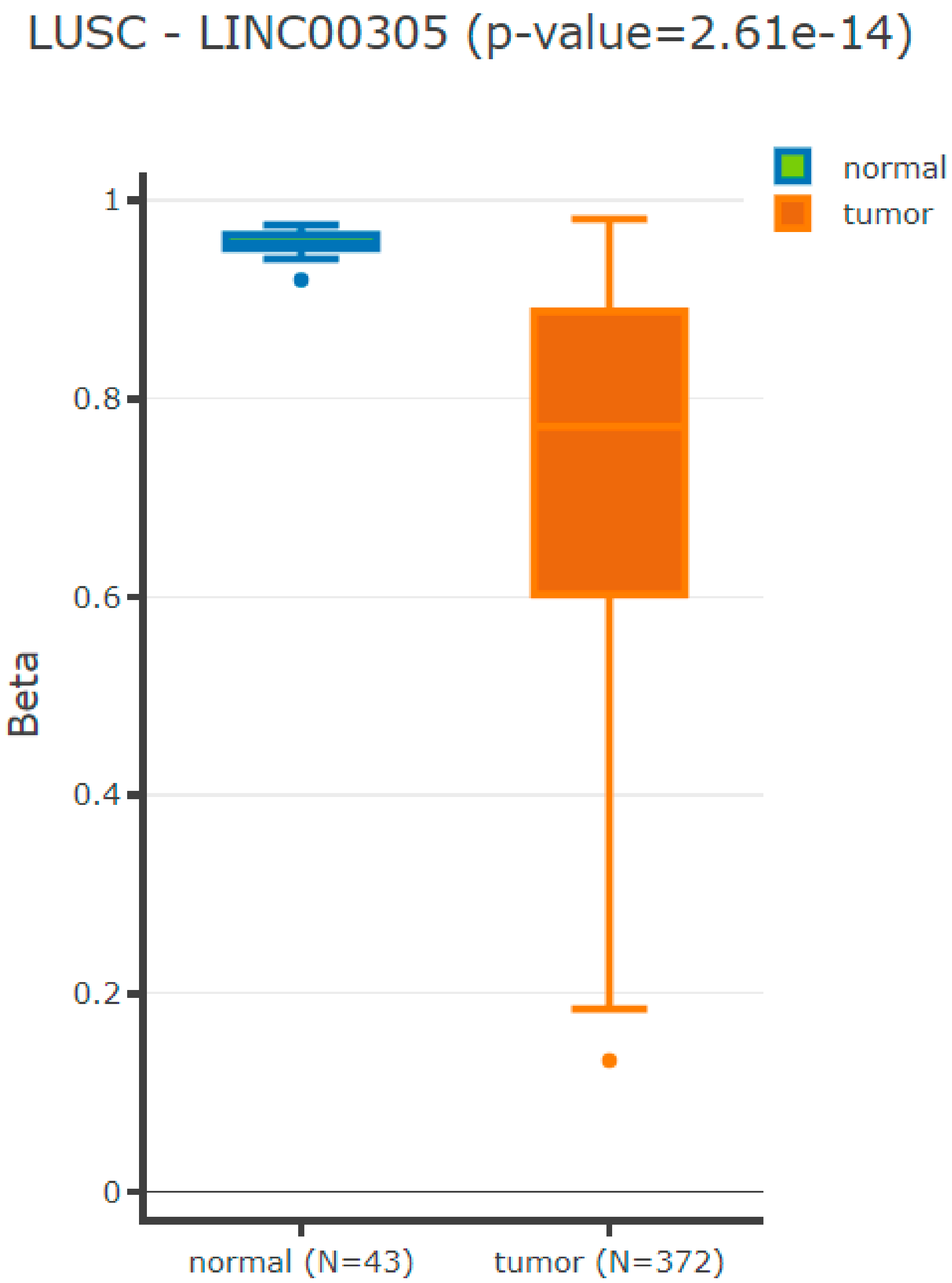

Figure S1-E. Differentially (tumor/normal tissues) methylated promoter region of *LINC00305* found in LUSC cohort: |beta-value difference| > 0.2 and independent Student's t-test adjusted p-value ≤ 0.05; hypermethylation and hypomethylation, beta-values > 0.2 and < – 0.2 at adj. p-value ≤ 0.05, respectively). Differentially methylated genes were explored using DNMIVD: DNA Methylation Interactive Visualization Database [S1].

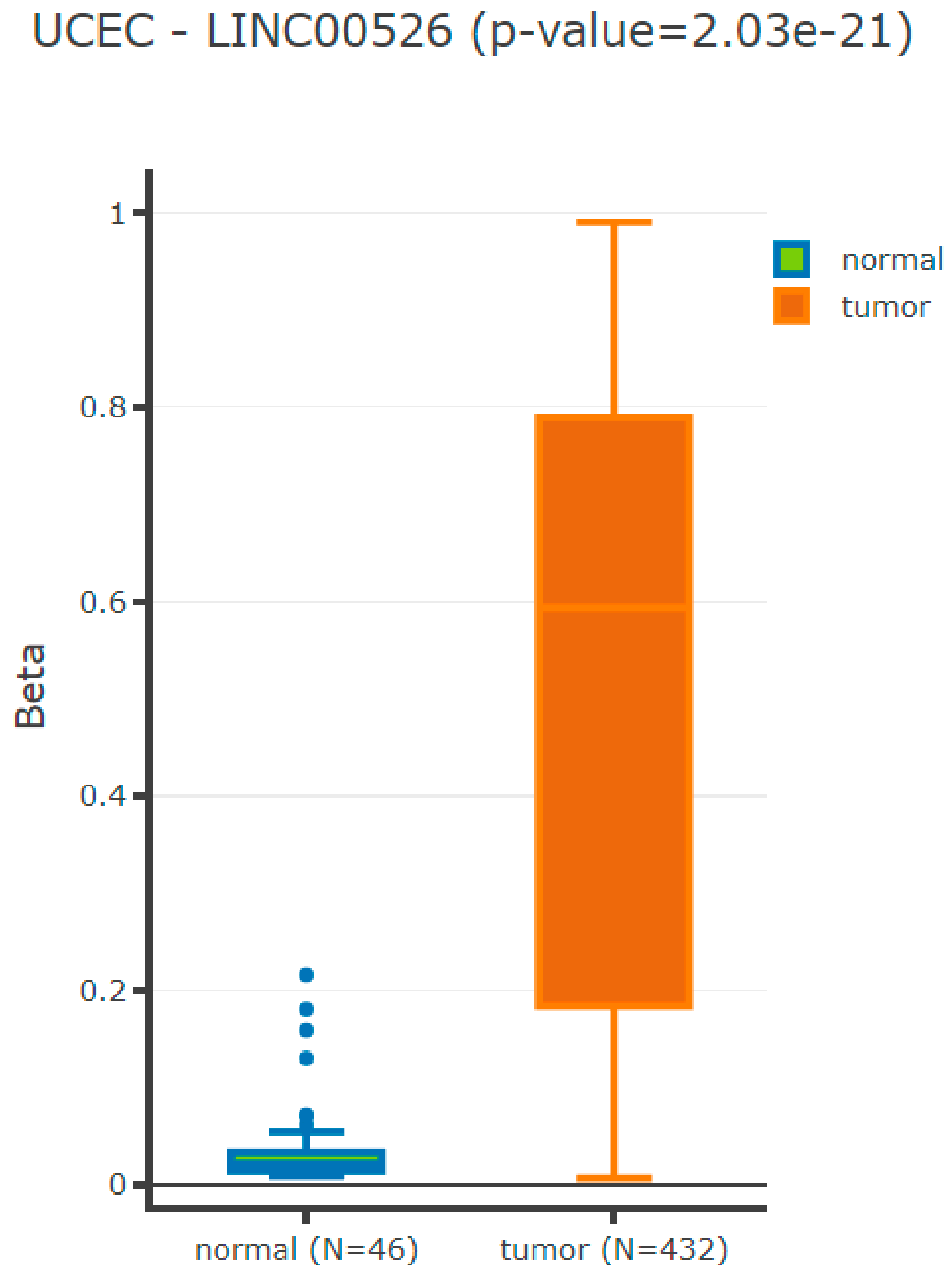

Figure S1-F. Differentially (tumor/normal tissues) methylated promoter region of *LINC00526* found in UCEC cohort: |beta-value difference| > 0.2 and independent Student's t-test adjusted p-value ≤ 0.05; hypermethylation and hypomethylation, beta-values > 0.2 and < – 0.2 at adj. p-value ≤ 0.05, respectively). Differentially methylated genes were explored using DNMIVD: DNA Methylation Interactive Visualization Database [S1].

References

[S1] Ding, W.; Chen, J.; Feng, G.; Chen, G.; Wu, J.; Guo, Y.; Ni, X.; Shi, T. DNMIVD: DNA Methylation Interactive Visualization Database. Nucleic Acids Res 2020, 48, D856–D862, doi:10.1093/nar/gkz830
