# Supplementary material for: Long Intergenic Non-Coding RNAs of Human Chromosome 18: Focus on Cancers
Source: Biomedicines. 2024 Feb 28;12(3):544. doi: 10.3390/biomedicines12030544 (PMC10968066; doi:10.3390/biomedicines12030544)
Supplement: Supplementary file 1 [file biomedicines-12-00544-s001.zip › Figure_S2_Ershov.pdf]

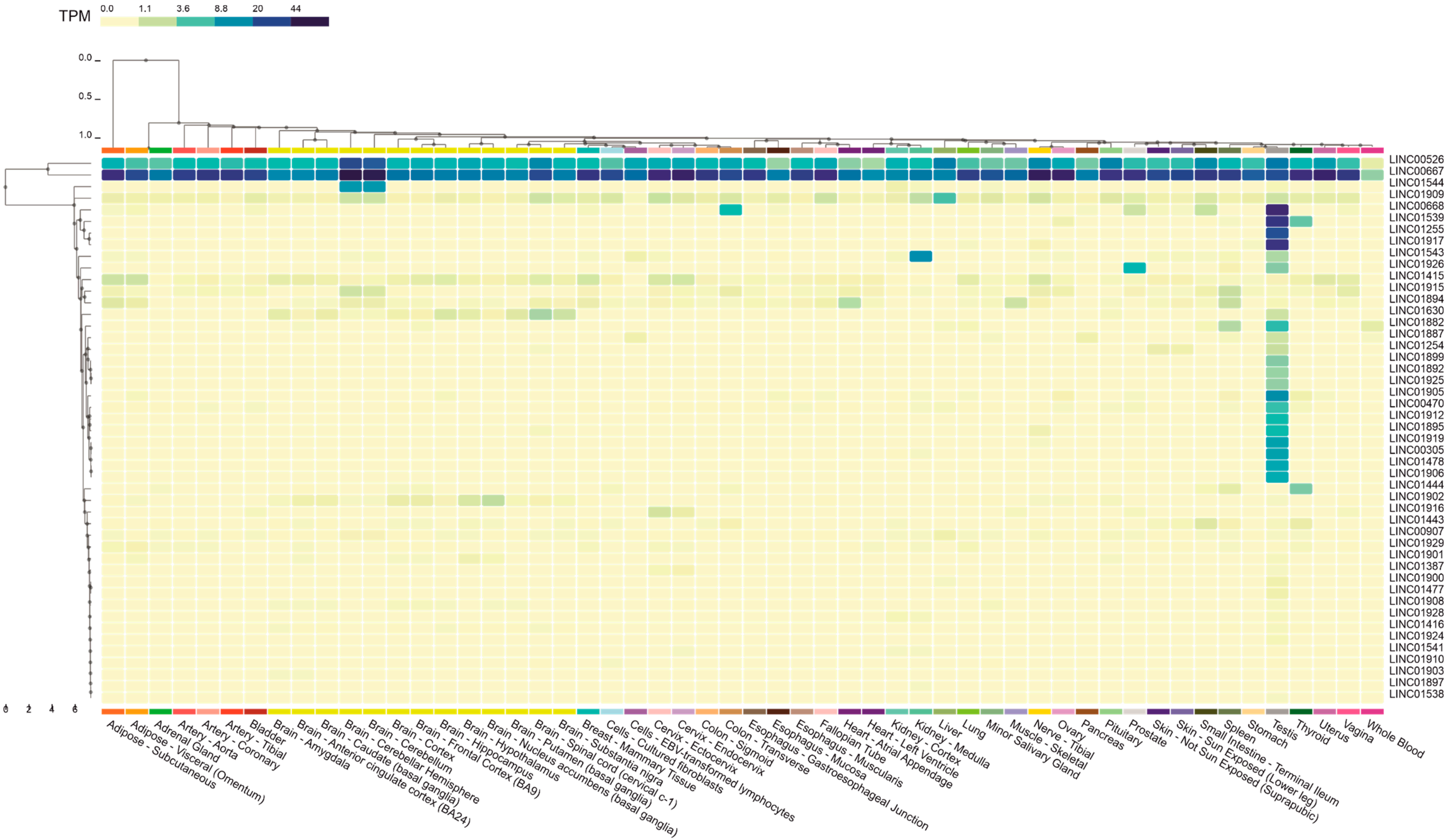


Figure S2. Gene expression patterns of lincRNAs of human chromosome 18 in normal tissues (this image was created using 'GTEx Multi Gene Query' data analysis interface of the Gene Tissue Expression Portal, https://www.gtexportal.org/home/). Abbreviations: TPM – Transcript per Million.
