# Supplementary material for: Long Intergenic Non-Coding RNAs of Human Chromosome 18: Focus on Cancers
Source: Biomedicines. 2024 Feb 28;12(3):544. doi: 10.3390/biomedicines12030544 (PMC10968066; doi:10.3390/biomedicines12030544)
Supplement: Supplementary file 1 [file biomedicines-12-00544-s001.zip › Figure_S3_Ershov.pdf]

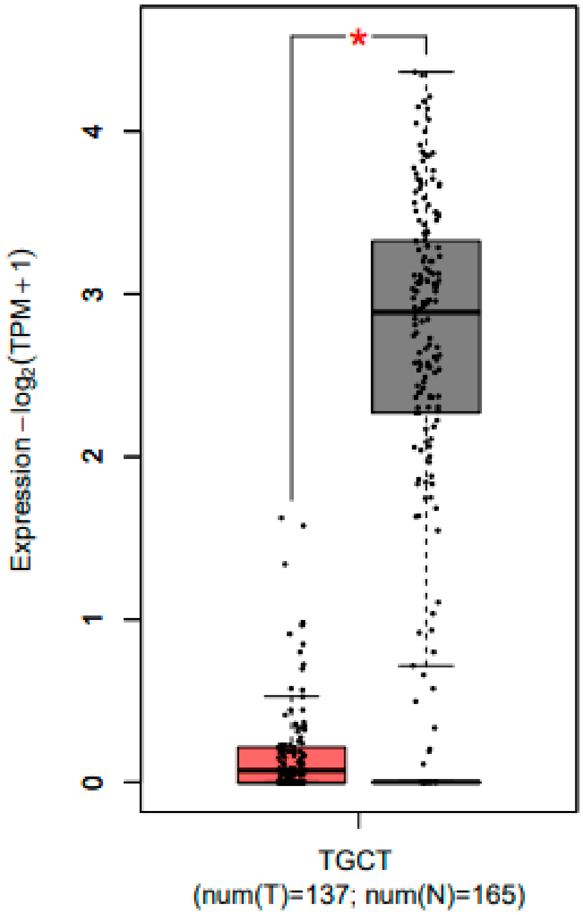

Figure S3-A. Differentially expressed *LINC00305* in testicular germ cell tumor (TGCT). Analysis was performed the web-based tool GEPIA2 at following settings: |$\log_2$fold change| > 1.5 tumor/normal tissue and p-value< 0.01 using TCGA (The Cancer Genome Atlas) and GTEx (Gene Tissue Expression databases) as data sources. The box-plot is an original image from GEPIA2.

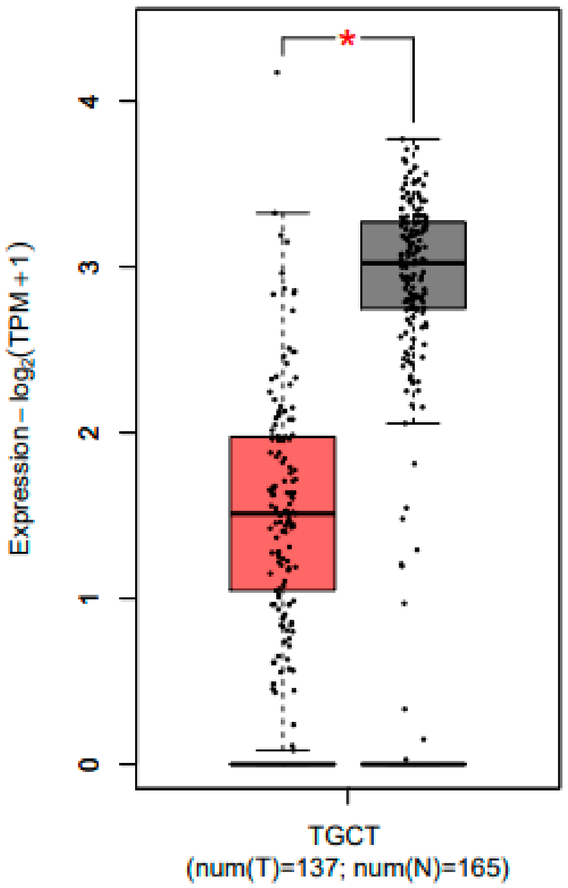

Figure S3-B. Differentially expressed *LINC00470* in testicular germ cell tumor (TGCT). Analysis was performed the web-based tool GEPIA2 at following settings: $|\log_2\text{fold change}| > 1.5$ tumor/normal tissue and p-value< 0.01 using TCGA (The Cancer Genome Atlas) and GTEx (Gene Tissue Expression databases) as data sources. The box-plot is an original image from GEPIA2.

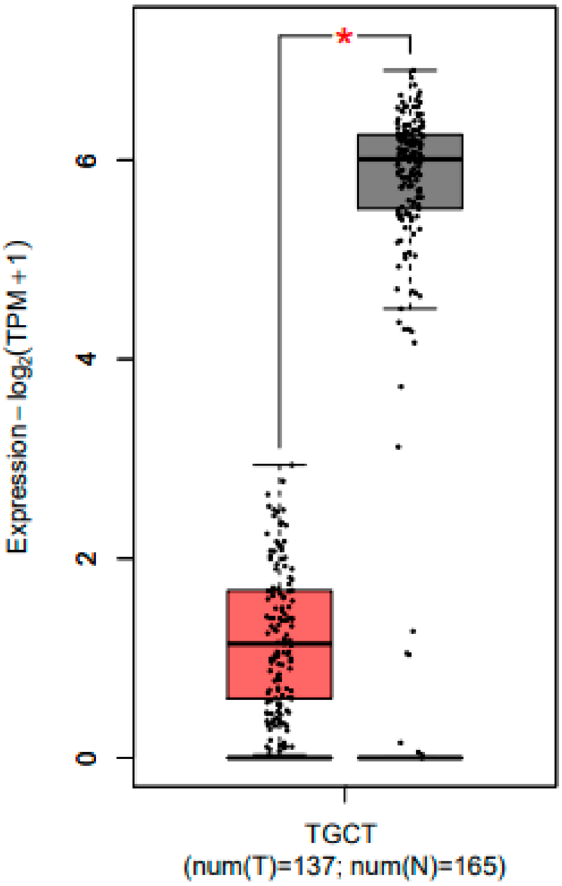

Figure S3-C. Differentially expressed *LINC00668* in testicular germ cell tumor (TGCT). Analysis was performed the web-based tool GEPIA2 at following settings: $|\log_2 \text{fold change}| > 1.5$ tumor/normal tissue and p-value< 0.01 using TCGA (The Cancer Genome Atlas) and GTEx (Gene Tissue Expression databases) as data sources. The box-plot is an original image from GEPIA2.

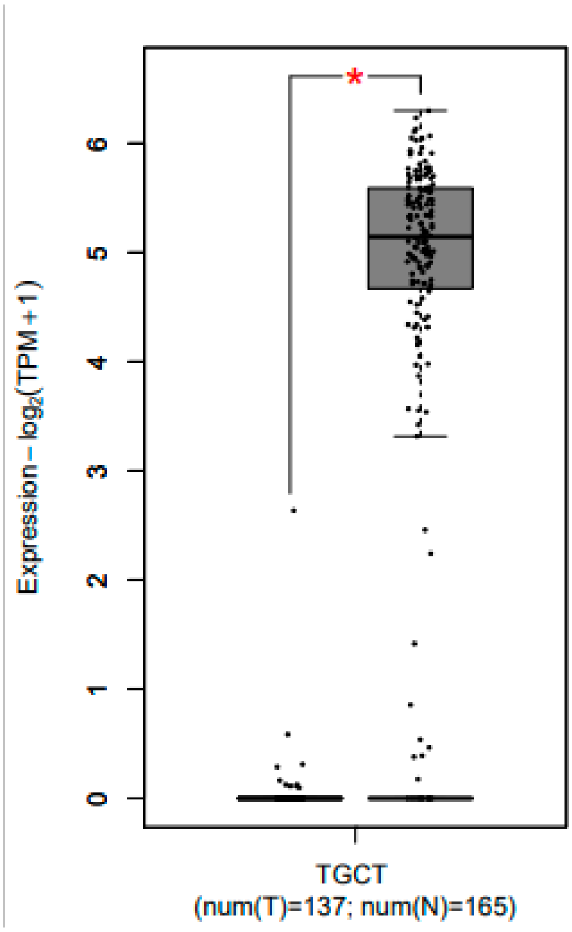

Figure S3-D. Differentially expressed *LINC01255* in testicular germ cell tumor (TGCT). Analysis was performed the web-based tool GEPIA2 at following settings: $|\log_2$fold change$| > 1.5$ tumor/normal tissue and p-value< 0.01 using TCGA (The Cancer Genome Atlas) and GTEx (Gene Tissue Expression databases) as data sources. The box-plot is an original image from GEPIA2.

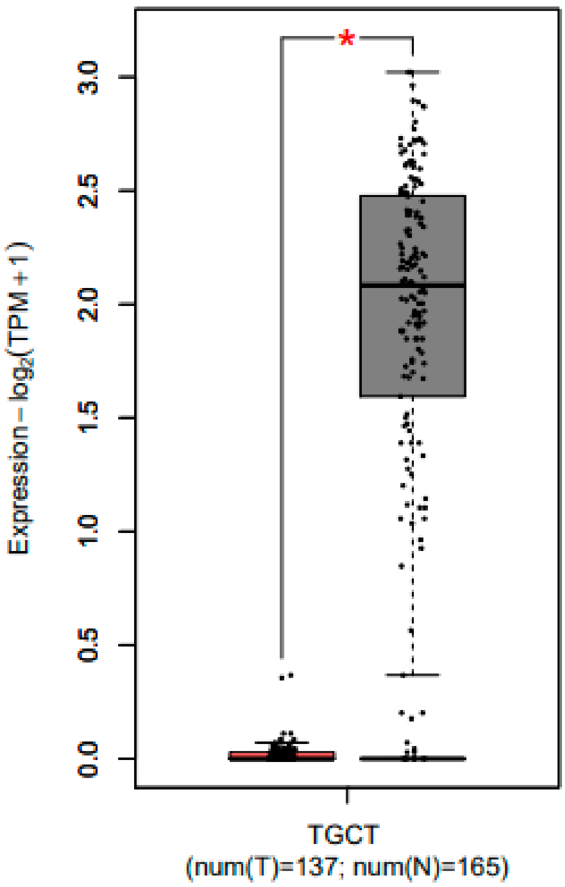

Figure S3-E. Differentially expressed *LINC01478* in testicular germ cell tumor (TGCT). Analysis was performed the web-based tool GEPIA2 at following settings: $|\log_2 \text{fold change}| > 1.5$ tumor/normal tissue and p-value< 0.01 using TCGA (The Cancer Genome Atlas) and GTEx (Gene Tissue Expression databases) as data sources. The box-plot is an original image from GEPIA2.

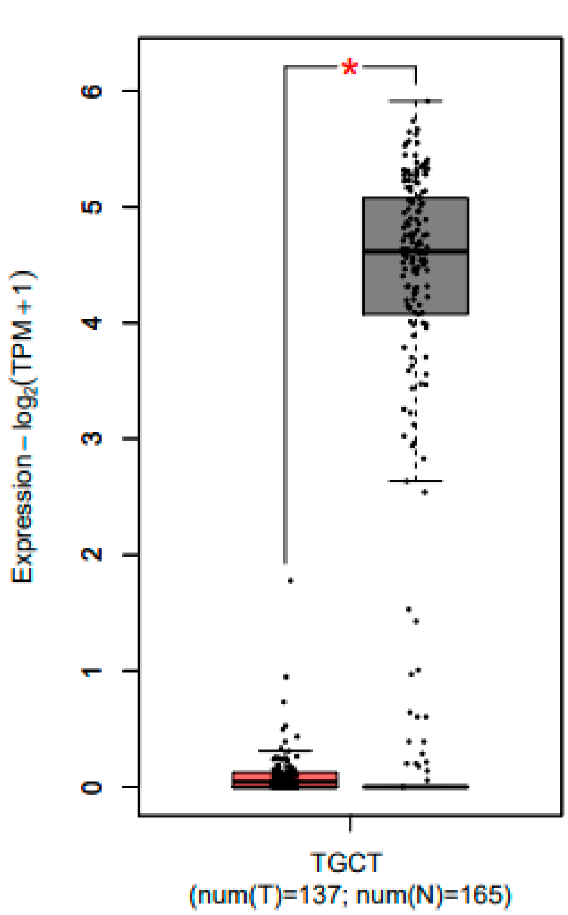

Figure S3-F. Differentially expressed *LINC01539* in testicular germ cell tumor (TGCT). Analysis was performed the web-based tool GEPIA2 at following settings: |log2fold change| > 1.5 tumor/normal tissue and p-value< 0.01 using TCGA (The Cancer Genome Atlas) and GTEx (Gene Tissue Expression databases) as data sources. The box-plot is an original image from GEPIA2.

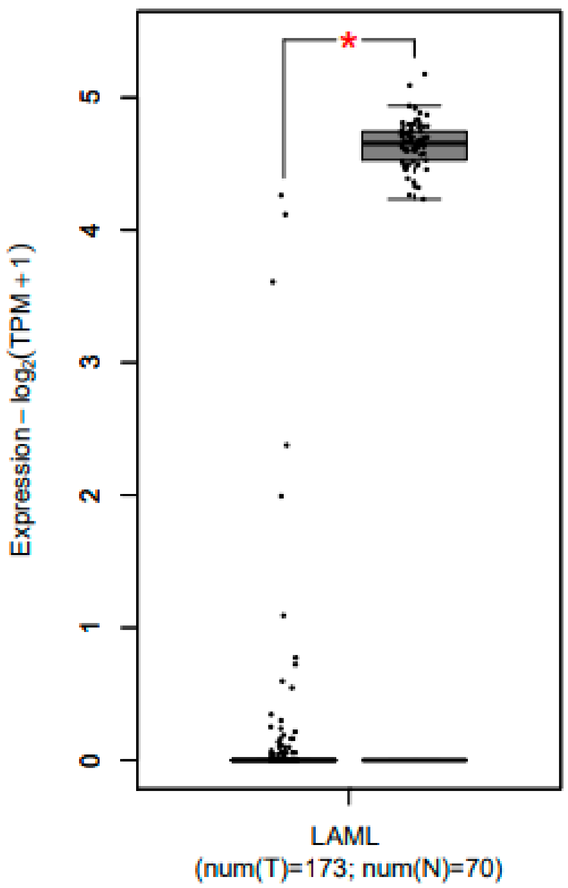

Figure S3-G. Differentially expressed *LINC00470* in acute myeloid leukemia (LAML). Analysis was performed the web-based tool GEPIA2 at following settings: $|\log_2$fold change$|$ > 1.5 tumor/normal tissue and p-value< 0.01 using TCGA (The Cancer Genome Atlas) and GTEx (Gene Tissue Expression databases) as data sources. The box-plot is an original image from GEPIA2.

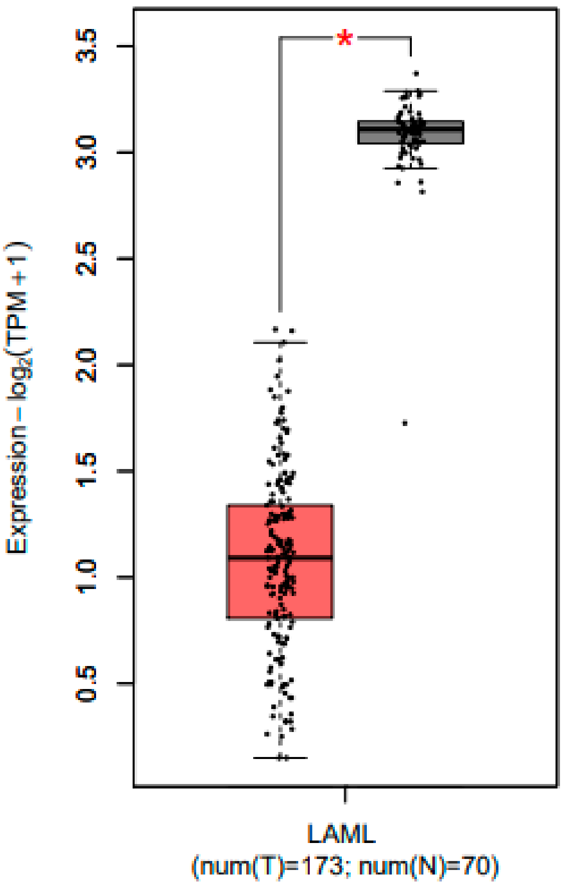

Figure S3-H. Differentially expressed *LINC00526* in acute myeloid leukemia (LAML). Analysis was performed the web-based tool GEPIA2 at following settings: |log2fold change| > 1.5 tumor/normal tissue and p-value< 0.01 using TCGA (The Cancer Genome Atlas) and GTEx (Gene Tissue Expression databases) as data sources. The box-plot is an original image from GEPIA2.

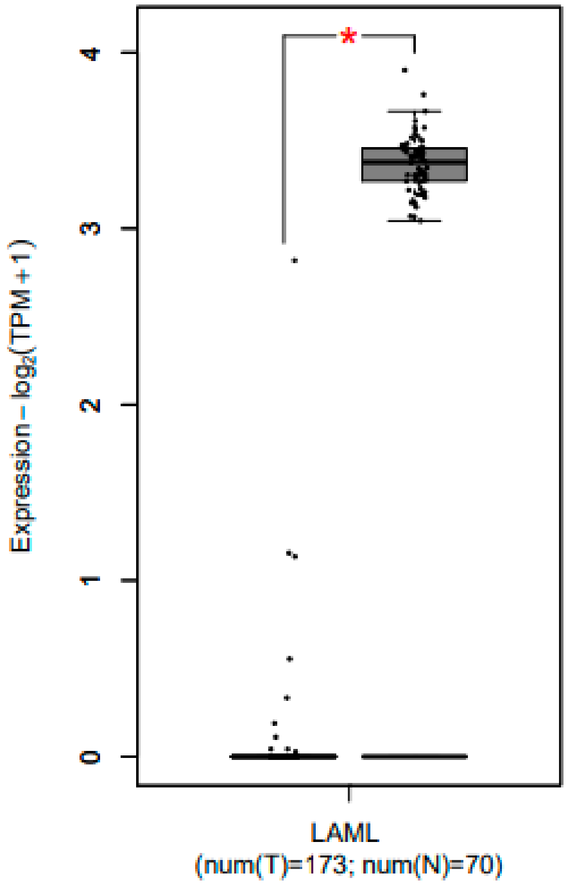

Figure S3-I. Differentially expressed *LINC00668* in acute myeloid leukemia (LAML). Analysis was performed the web-based tool GEPIA2 at following settings: |$\log_2$fold change| > 1.5 tumor/normal tissue and p-value< 0.01 using TCGA (The Cancer Genome Atlas) and GTEx (Gene Tissue Expression databases) as data sources. The box-plot is an original image from GEPIA2.

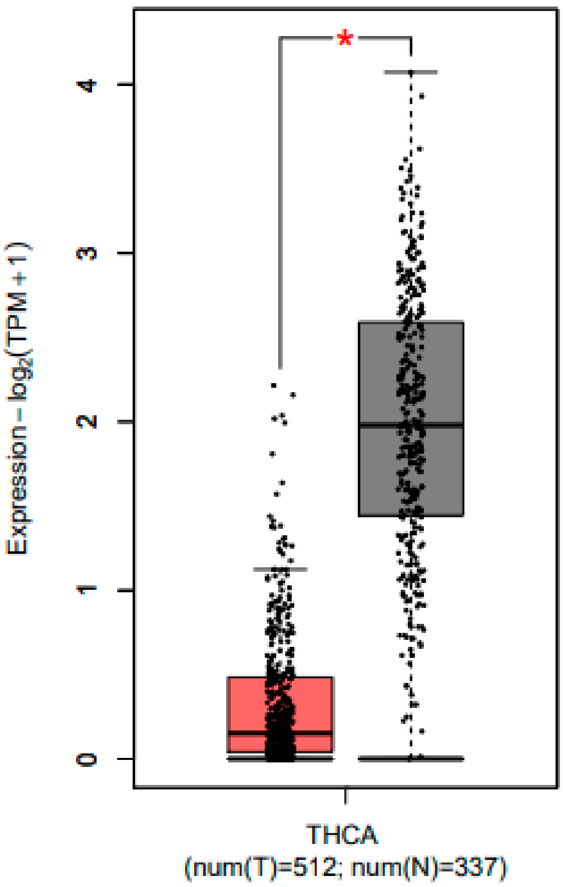

Figure S3-J. Differentially expressed *LINC01539* in thyroid cancer (THCA). Analysis was performed the web-based tool GEPIA2 at following settings: $|log_2 \text{fold change}| > 1.5$ tumor/normal tissue and p-value< 0.01 using TCGA (The Cancer Genome Atlas) and GTEx (Gene Tissue Expression databases) as data sources. The box-plot is an original image from GEPIA2.

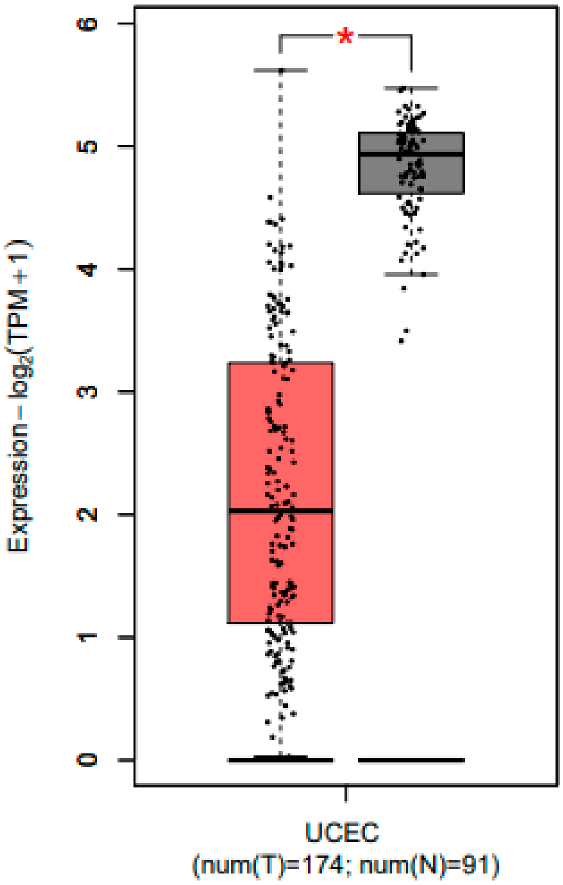

Figure S3-K. Differentially expressed *LINC00667* in uterine corpus endometrial cancer (UCEC). Analysis was performed the web-based tool GEPIA2 at following settings: |$\log_2$fold change| > 1.5 tumor/normal tissue and p-value< 0.01 using TCGA (The Cancer Genome Atlas) and GTEx (Gene Tissue Expression databases) as data sources. The box-plot is an original image from GEPIA2.

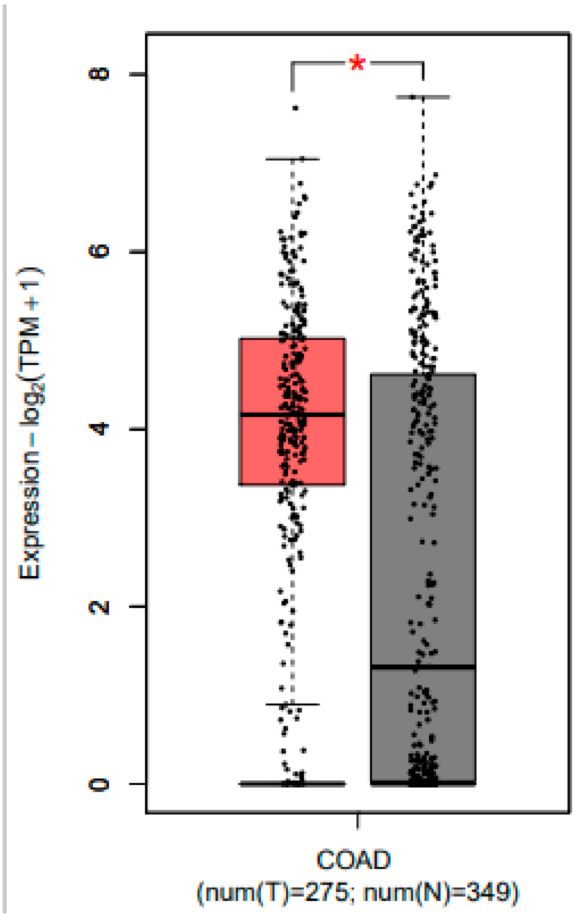

Figure S3-L. Differentially expressed *LINC00668* in uterine colorectal adenocarcinoma (COAD). Analysis was performed the web-based tool GEPIA2 at following settings: $|log_2fold\ change| > 1.5$ tumor/normal tissue and p-value< 0.01 using TCGA (The Cancer Genome Atlas) and GTEx (Gene Tissue Expression databases) as data sources. The box-plot is an original image from GEPIA2.

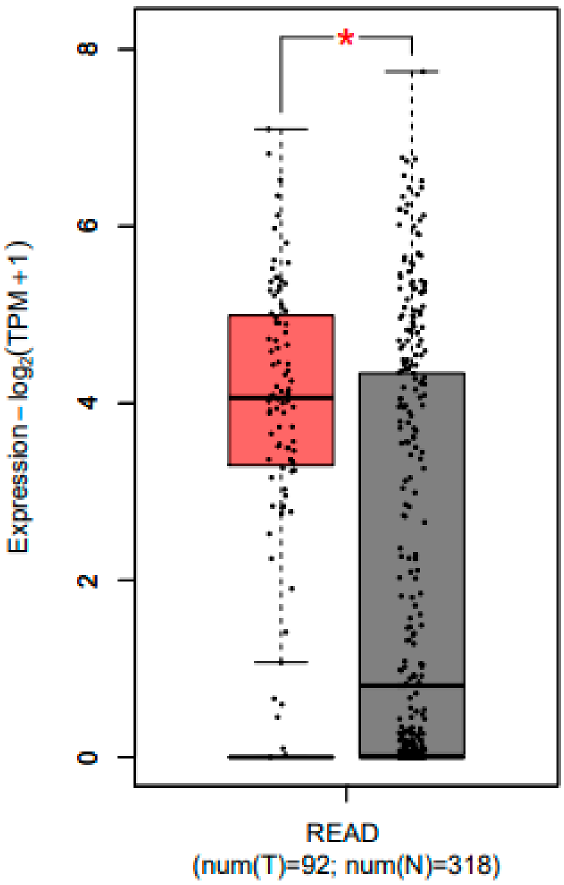

Figure S3-M. Differentially expressed *LINC00668* in rectal adenocarcinoma (READ). Analysis was performed the web-based tool GEPIA2 at following settings: |log_2fold change| > 1.5 tumor/normal tissue and p-value< 0.01 using TCGA (The Cancer Genome Atlas) and GTEx (Gene Tissue Expression databases) as data sources. The box-plot is an original image from GEPIA2.

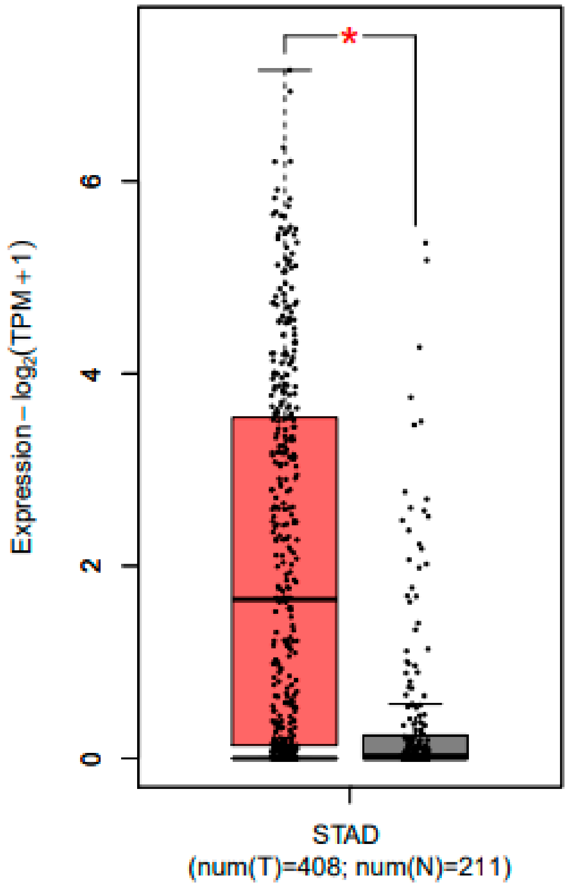

Figure S3-N. Differentially expressed *LINC00668* in stomach adenocarcinoma (STAD). Analysis was performed the web-based tool GEPIA2 at following settings: $|\log_2 \text{fold change}| > 1.5$ tumor/normal tissue and p-value< 0.01 using TCGA (The Cancer Genome Atlas) and GTEx (Gene Tissue Expression databases) as data sources. The box-plot is an original image from GEPIA2.

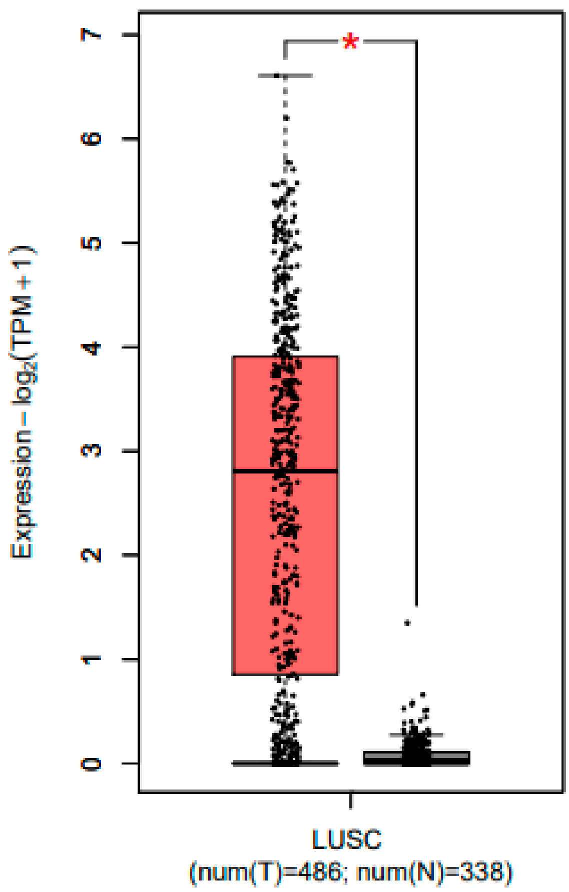

Figure S3-O. Differentially expressed *LINC00668* in lung squamous carcinoma (LUSC). Analysis was performed the web-based tool GEPIA2 at following settings: $|\log_2 \text{fold change}| > 1.5$ tumor/normal tissue and p-value< 0.01 using TCGA (The Cancer Genome Atlas) and GTEx (Gene Tissue Expression databases) as data sources. The box-plot is an original image from GEPIA2.

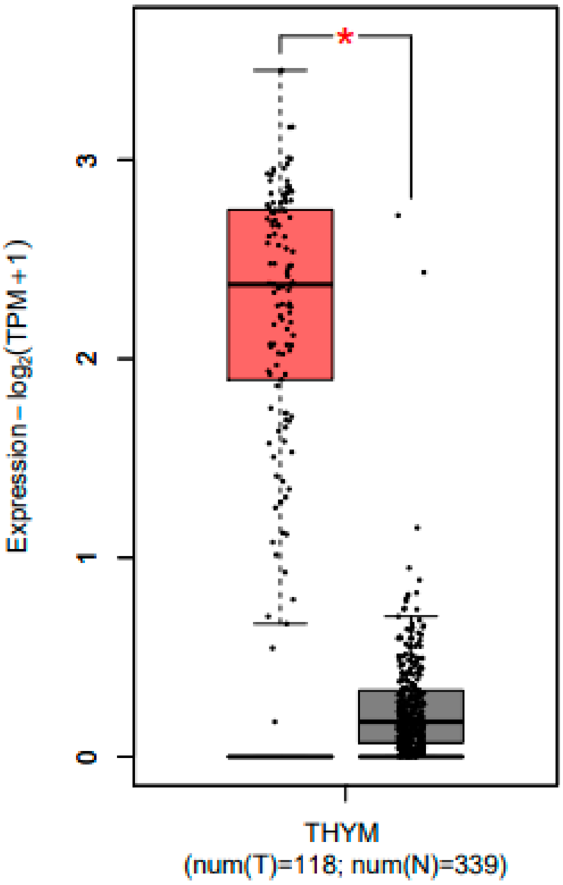

Figure S3-P. Differentially expressed *LINC00526* in thymoma (THYM). Analysis was performed the web-based tool GEPIA2 at following settings: $|\log_2\text{fold change}| > 1.5$ tumor/normal tissue and p-value< 0.01 using TCGA (The Cancer Genome Atlas) and GTEx (Gene Tissue Expression databases) as data sources. The box-plot is an original image from GEPIA2.

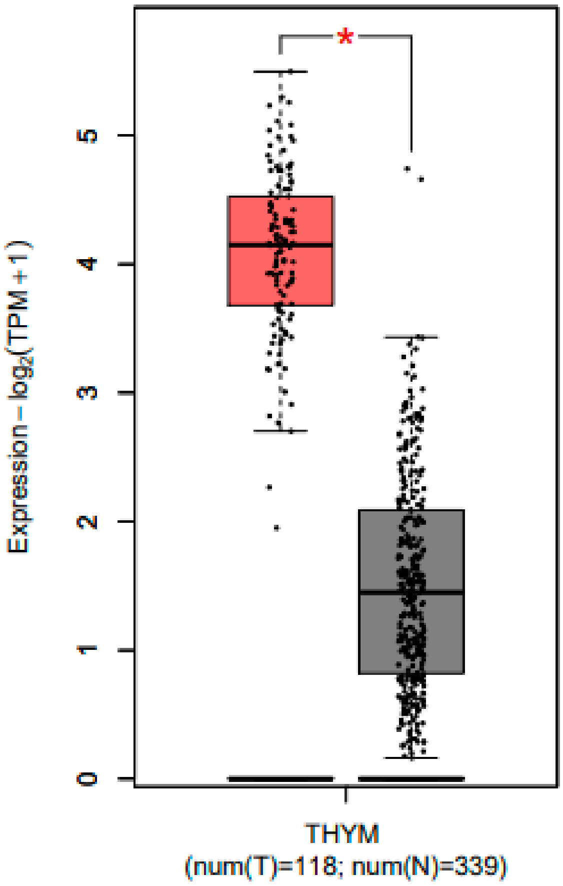

Figure S3-R. Differentially expressed *LINC00667* in thymoma (THYM). Analysis was performed the web-based tool GEPIA2 at following settings: $|\log_2\text{fold change}| > 1.5$ tumor/normal tissue and p-value< 0.01 using TCGA (The Cancer Genome Atlas) and GTEx (Gene Tissue Expression databases) as data sources. The box-plot is an original image from GEPIA2.

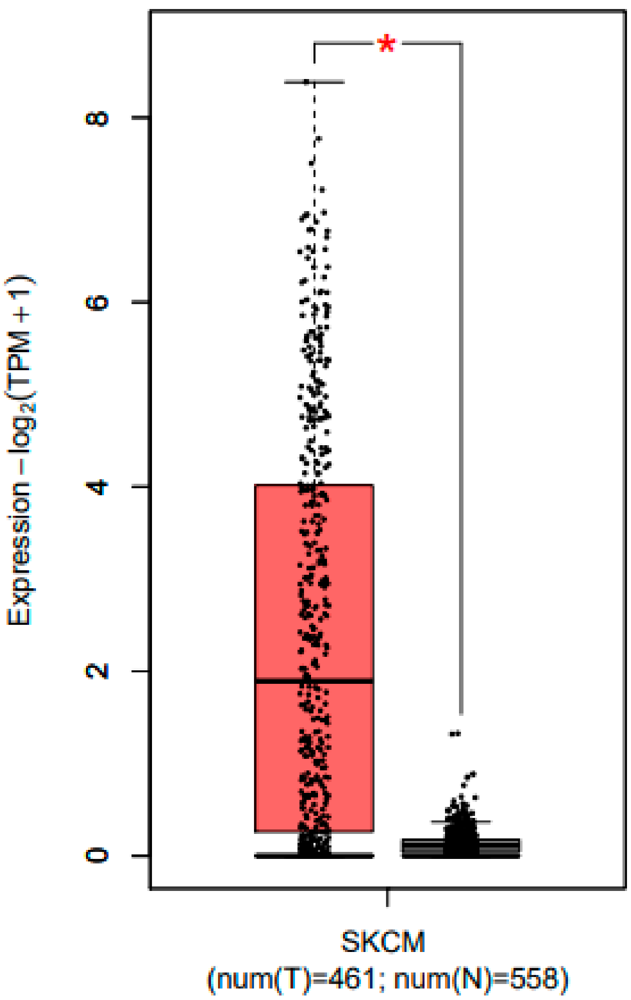

Figure S3-S. Differentially expressed *LINC01443* in skin cutaneous melanoma (SKCM). Analysis was performed the web-based tool GEPIA2 at following settings: $|\log_2$fold change$|$ > 1.5 tumor/normal tissue and p-value< 0.01 using TCGA (The Cancer Genome Atlas) and GTEx (Gene Tissue Expression databases) as data sources. The box-plot is an original image from GEPIA2.
