# Supplementary material for: Long Intergenic Non-Coding RNAs of Human Chromosome 18: Focus on Cancers
Source: Biomedicines. 2024 Feb 28;12(3):544. doi: 10.3390/biomedicines12030544 (PMC10968066; doi:10.3390/biomedicines12030544)
Supplement: Supplementary file 1 [file biomedicines-12-00544-s001.zip › Figure_S4_Ershov.pdf]

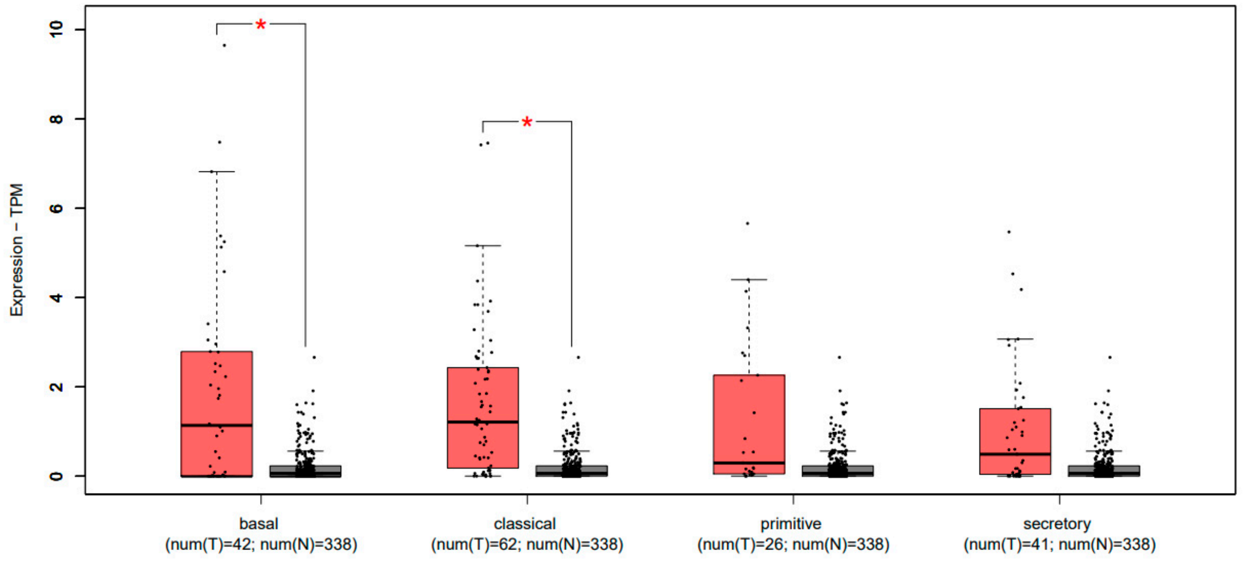

Figure S4-A. Subtype-specific expression of *LINC00470* in LUSC (lung squamous carcinoma) cohort in comparison with Gene Tissue Expression (GTEx) and TCGA normal cohorts. X-axis represents gene expression units as TPM (Transcript per Million). Analysis was performed using the web-based tool GEPIA2, asterisks are denoted p-values < 0.05. The box-plot is an original image from GEPIA2.

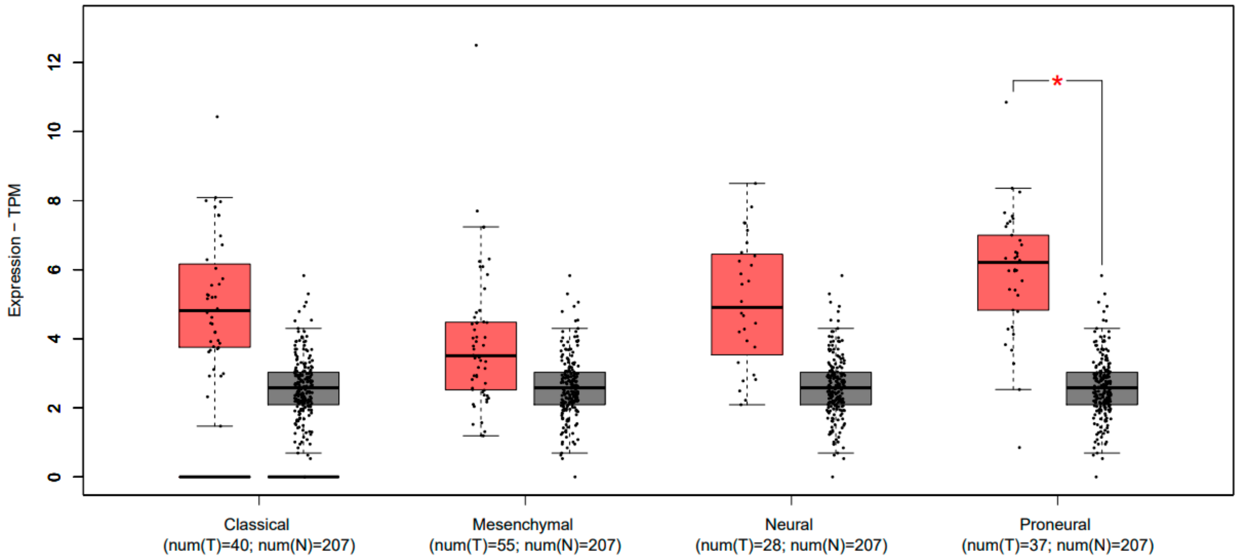

Figure S4-B. Subtype-specific expression of *LINC00526* in GBM (glioblastoma) cohort in comparison with Gene Tissue Expression (GTEx) and TCGA normal cohorts. X-axis represents gene expression units as TPM (Transcript per Million). Analysis was performed using the web-based tool GEPIA2, asterisks are denoted p-values < 0.05. The box-plot is an original image from GEPIA2

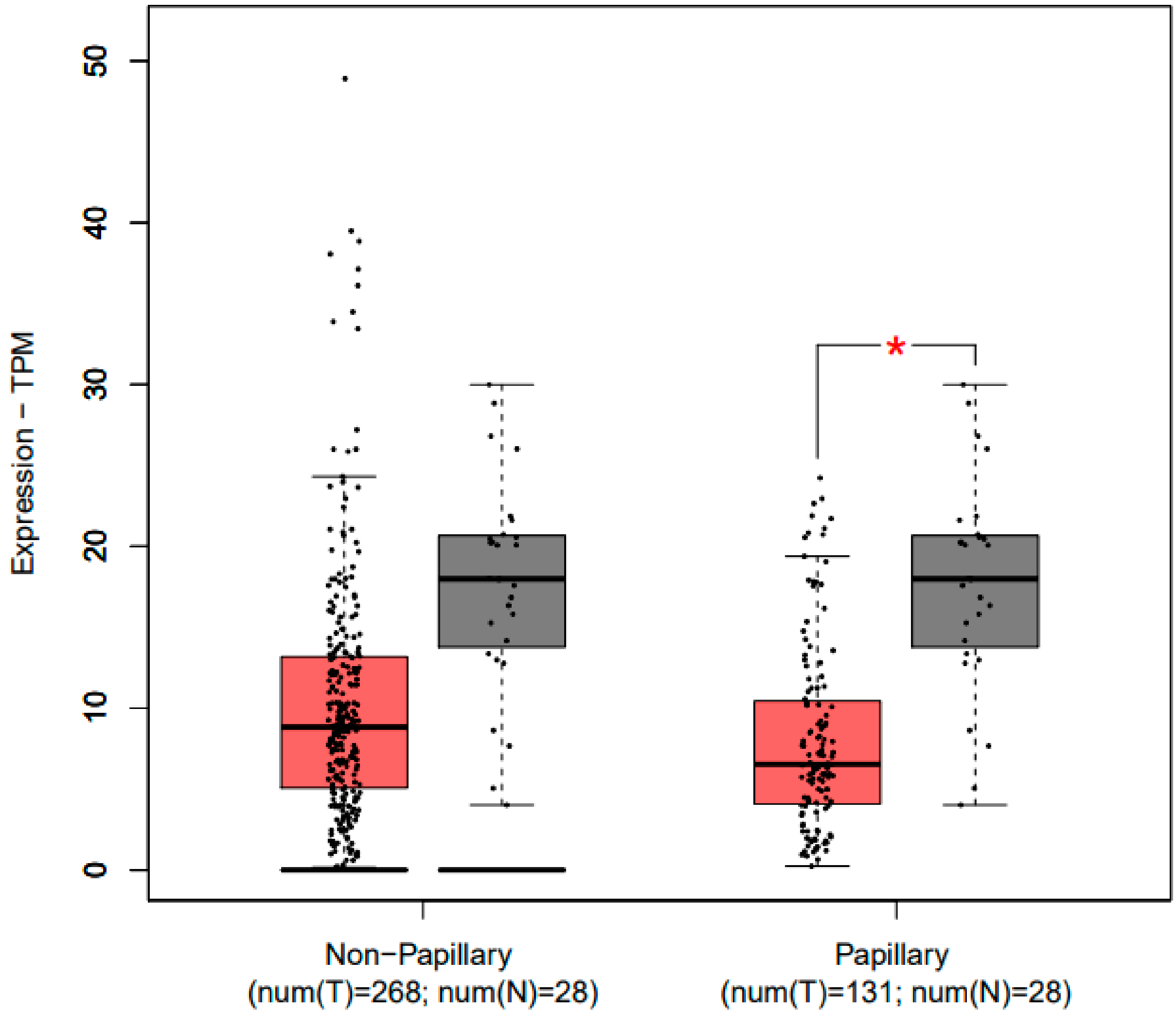

Figure S4-C. Subtype-specific expression of *LINC00667* in BLCA (bladder cancer) cohort in comparison with Gene Tissue Expression (GTEx) and TCGA normal cohorts. X-axis represents gene expression units as TPM (Transcript per Million). Analysis was performed using the web-based tool GEPIA2, asterisks are denoted p-values < 0.05. The box-plot is an original image from GEPIA2.

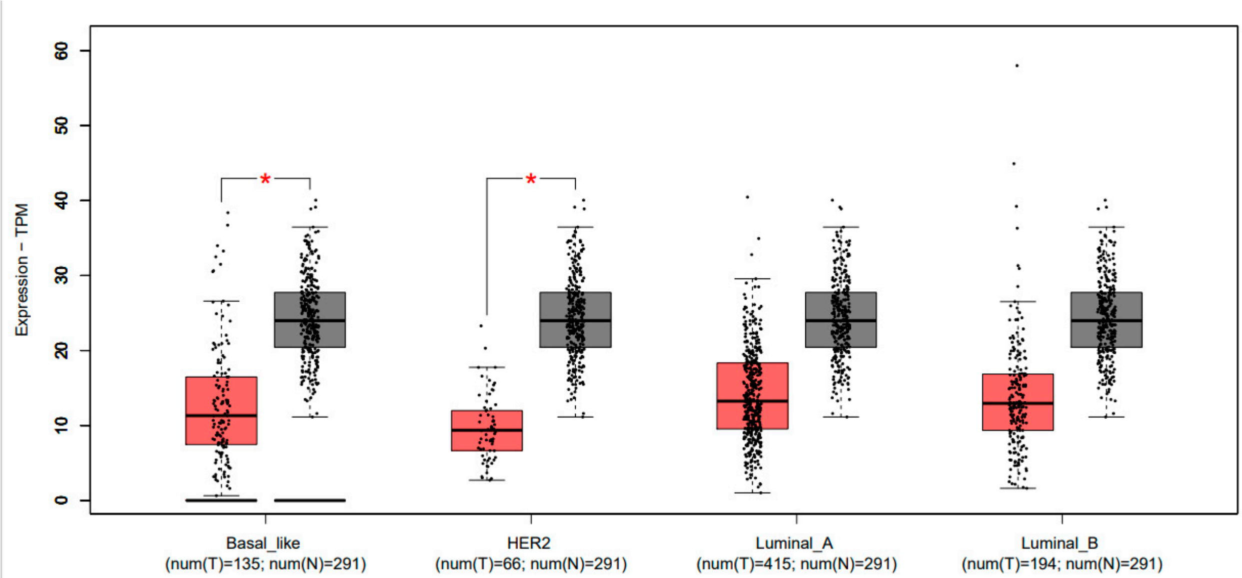

Figure S4-D. Subtype-specific expression of *LINC00667* in BRCA cohort in comparison with Gene Tissue Expression (GTEx) and TCGA normal cohorts. X-axis represents gene expression units as TPM (Transcript per Million). Analysis was performed using the web-based tool GEPIA2, asterisks are denoted p-values < 0.05. The box-plot is an original image from GEPIA2.

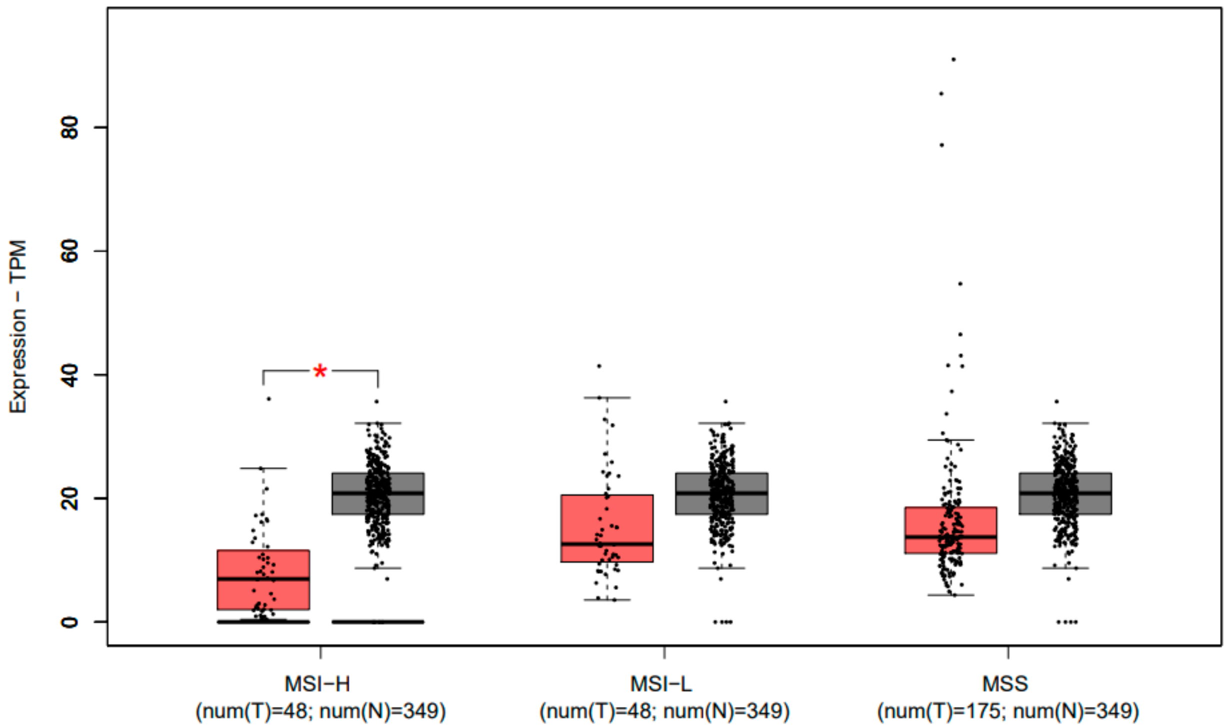

Figure S4-E. Subtype-specific expression of *LINC00667* in COAD (colorectal adenocarcinoma) cohort in comparison with Gene Tissue Expression (GTEx) and TCGA normal cohorts. X-axis represents gene expression units as TPM (Transcript per Million). Analysis was performed using the web-based tool GEPIA2, asterisks are denoted p-values < 0.05. The box-plot is an original image from GEPIA2.

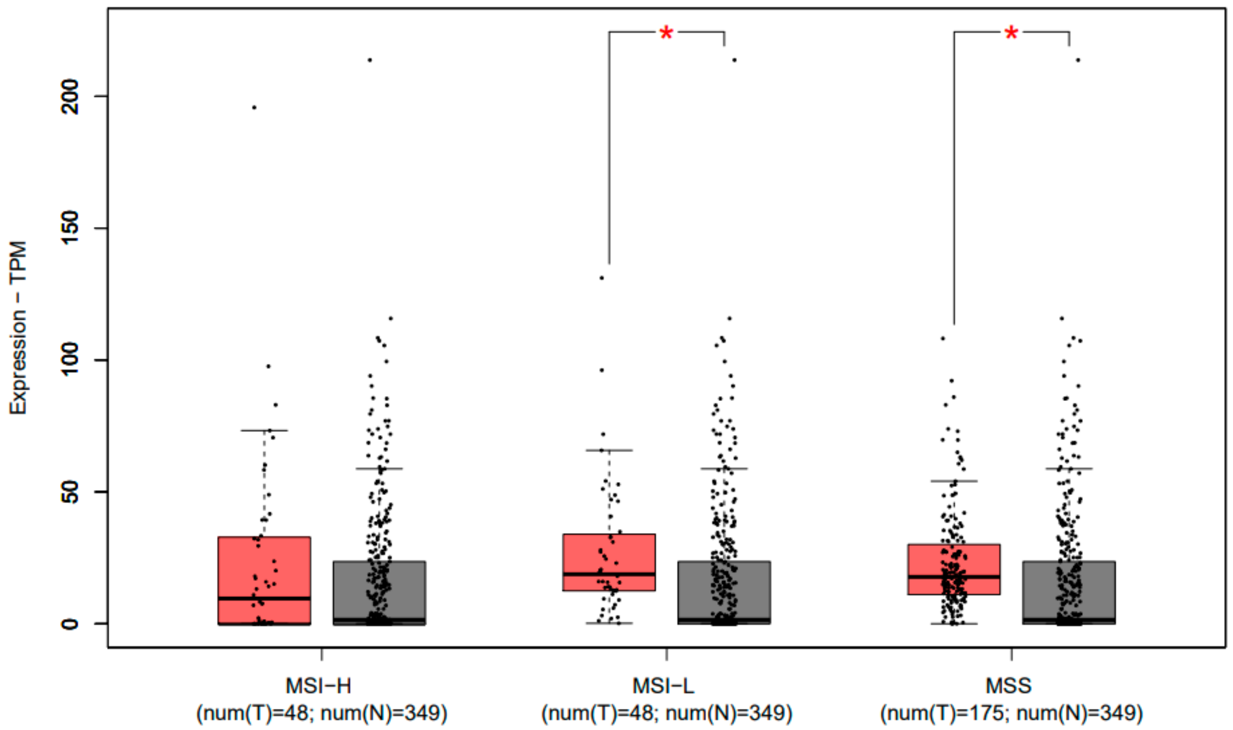

Figure S4-F. Subtype-specific expression of *LINC00668* in COAD (colorectal adenocarcinoma) cohort in comparison with Gene Tissue Expression (GTEx) and TCGA normal cohorts. X-axis represents gene expression units as TPM (Transcript per Million). Analysis was performed using the web-based tool GEPIA2, asterisks are denoted p-values < 0.05. The box-plot is an original image from GEPIA2.

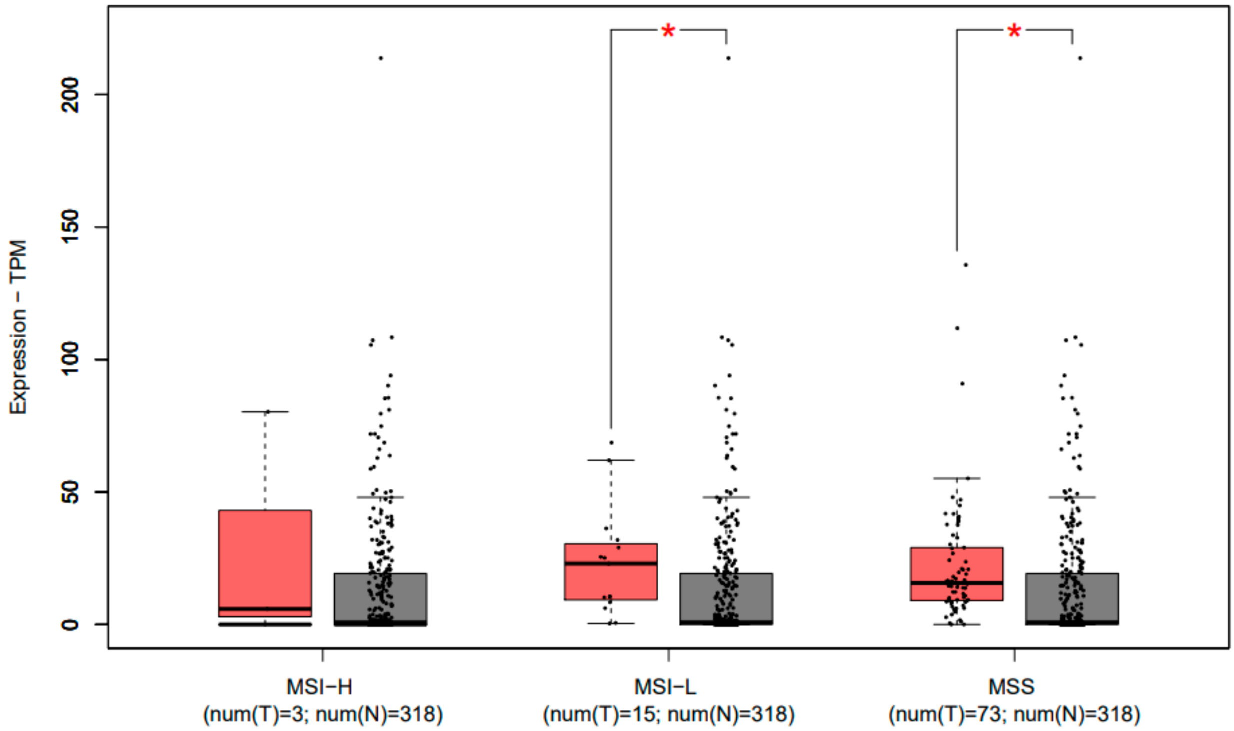

Figure S4-G. Subtype-specific expression of *LINC00668* in READ (rectal adenocarcinoma) cohort in comparison with Gene Tissue Expression (GTEx) and TCGA normal cohorts. X-axis represents gene expression units as TPM (Transcript per Million). Analysis was performed using the web-based tool GEPIA2, asterisks are denoted p-values < 0.05. The box-plot is an original image from GEPIA2.

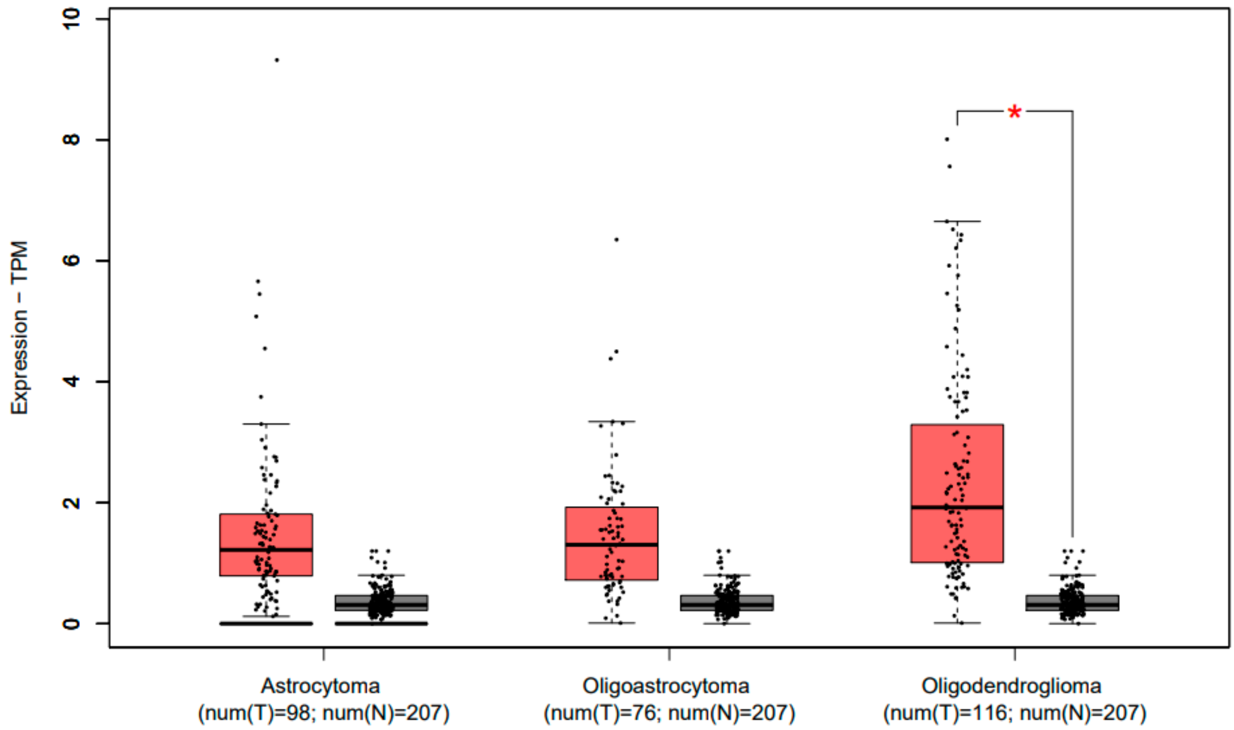

Figure S4-H. Subtype-specific expression of *LINC01415* in LGG (low grade glioma) cohort in comparison with Gene Tissue Expression (GTEx) and TCGA normal cohorts. X-axis represents gene expression units as TPM (Transcript per Million). Analysis was performed using the web-based tool GEPIA2, asterisks are denoted p-values < 0.05. The box-plot is an original image from GEPIA2.

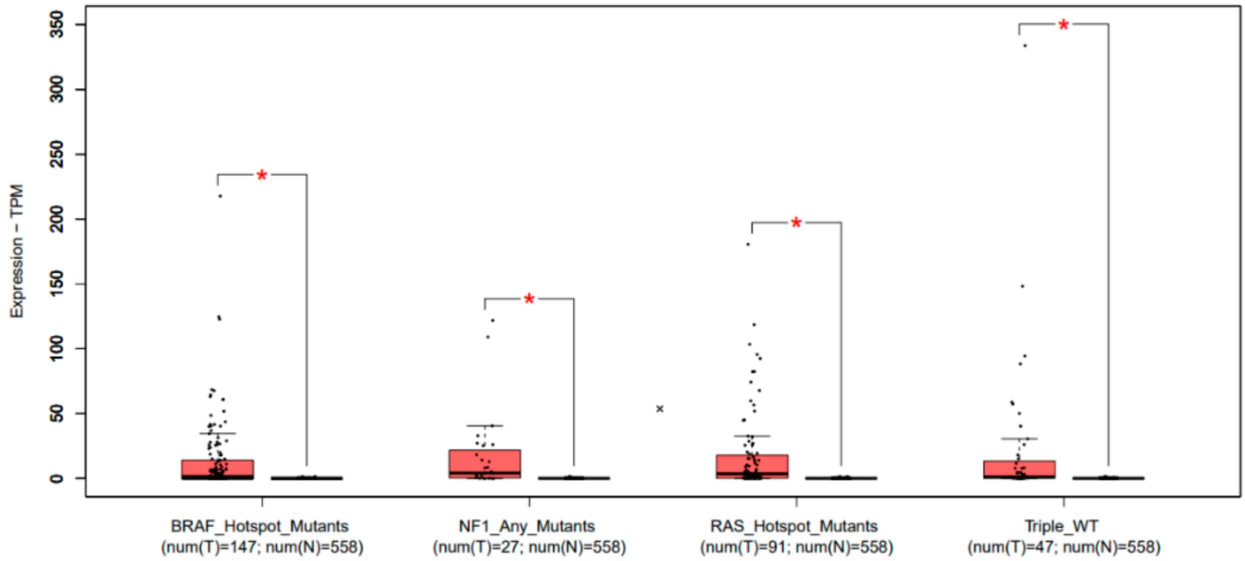

Figure S4-I. Subtype-specific expression of *LINC01443* in SKCM (skin cutaneous melanoma) cohort in comparison with Gene Tissue Expression (GTEx) and TCGA normal cohorts. X-axis represents gene expression units as TPM (Transcript per Million). Analysis was performed using the web-based tool GEPIA2, asterisks are denoted p-values < 0.05. The box-plot is an original image from GEPIA2.
