# Supplementary material for: Long Intergenic Non-Coding RNAs of Human Chromosome 18: Focus on Cancers
Source: Biomedicines. 2024 Feb 28;12(3):544. doi: 10.3390/biomedicines12030544 (PMC10968066; doi:10.3390/biomedicines12030544)
Supplement: Supplementary file 1 [file biomedicines-12-00544-s001.zip › Figure_S5_Ershov.pdf]

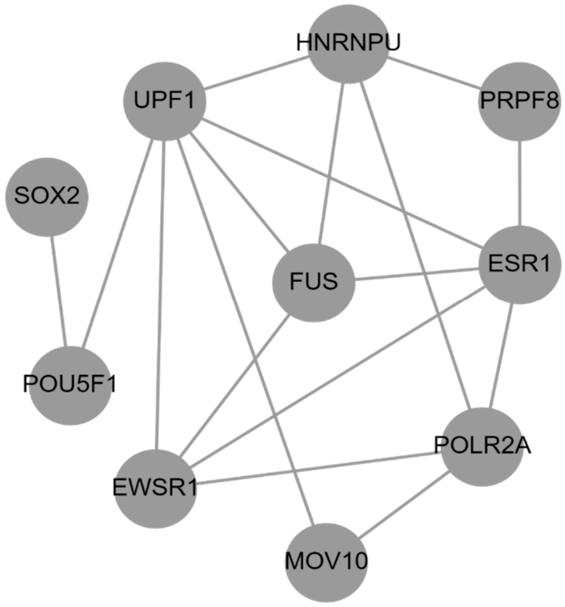

Figure S5. The subnetwork of protein-protein interactions of lincRNA-binding proteins using the Biogrid database as a data source (original output from WebGestalt server).
Abbreviations:
ESR1, estrogen receptor 1
EWSR1, EWS RNA binding protein 1
FUS, FUS RNA binding protein
HNRNPU, heterogeneous nuclear ribonucleoprotein U
MOV10, Mov10 RNA helicase
POLR2A, RNA polymerase II subunit A
POU5F1, POU class 5 homeobox 1
PRPF8, pre-mRNA processing factor 8
SOX2, SRY-box transcription factor 2
UPF1, UPF1 RNA helicase and ATPase
