# Supplementary material for: Long Intergenic Non-Coding RNAs of Human Chromosome 18: Focus on Cancers
Source: Biomedicines. 2024 Feb 28;12(3):544. doi: 10.3390/biomedicines12030544 (PMC10968066; doi:10.3390/biomedicines12030544)
Supplement: Supplementary file 1 [file biomedicines-12-00544-s001.zip › Figure_S6_Ershov.pdf]

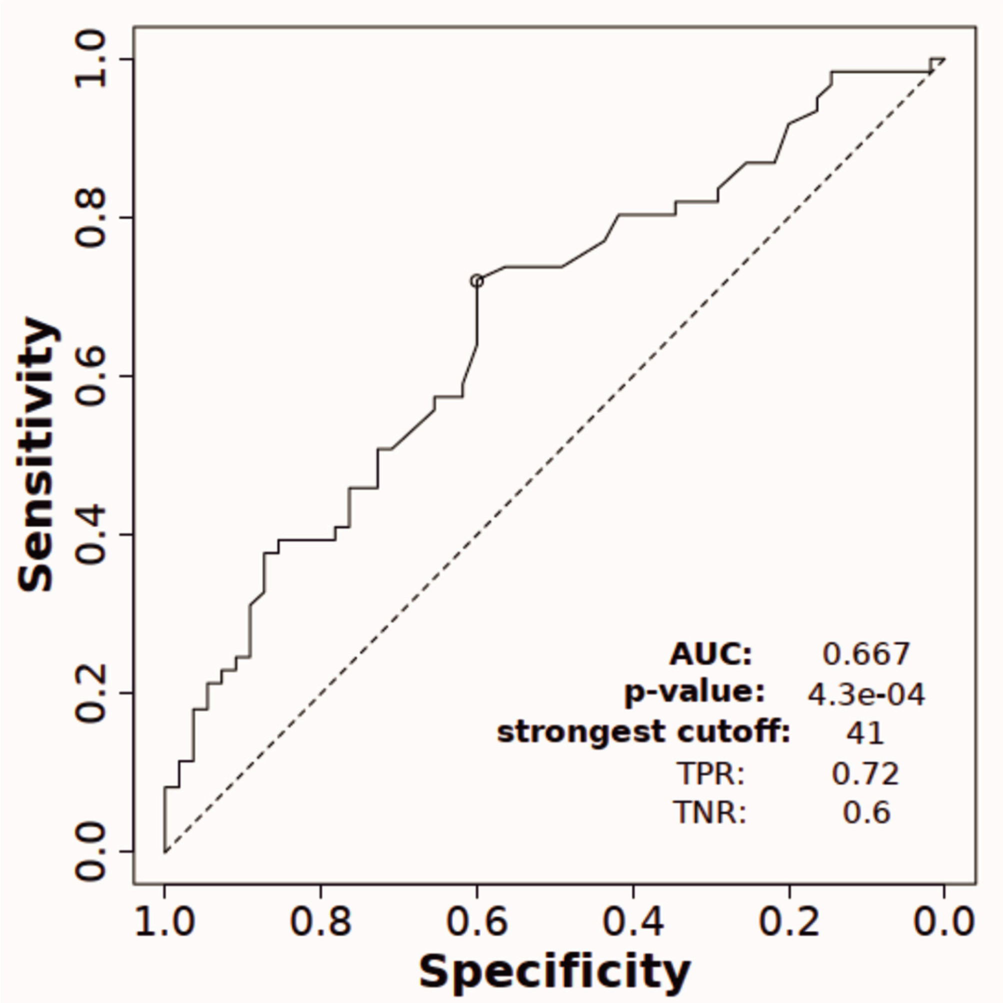

Figure S6-A. Sensitivity and specificity of a predictive model based on association of *LINC00667* gene expression levels in metastatic melanoma (61 non-responders and 55 responders) with response to inhibitor of CTLA-4 (ipilimumab). AUC-value = 0.67, Mann-Whitney test p-value = 0.0019, fold change of median gene expression values (non-responders vs responders) = 2.2. Analysis was performed using the web-based tool ROC-plotter.

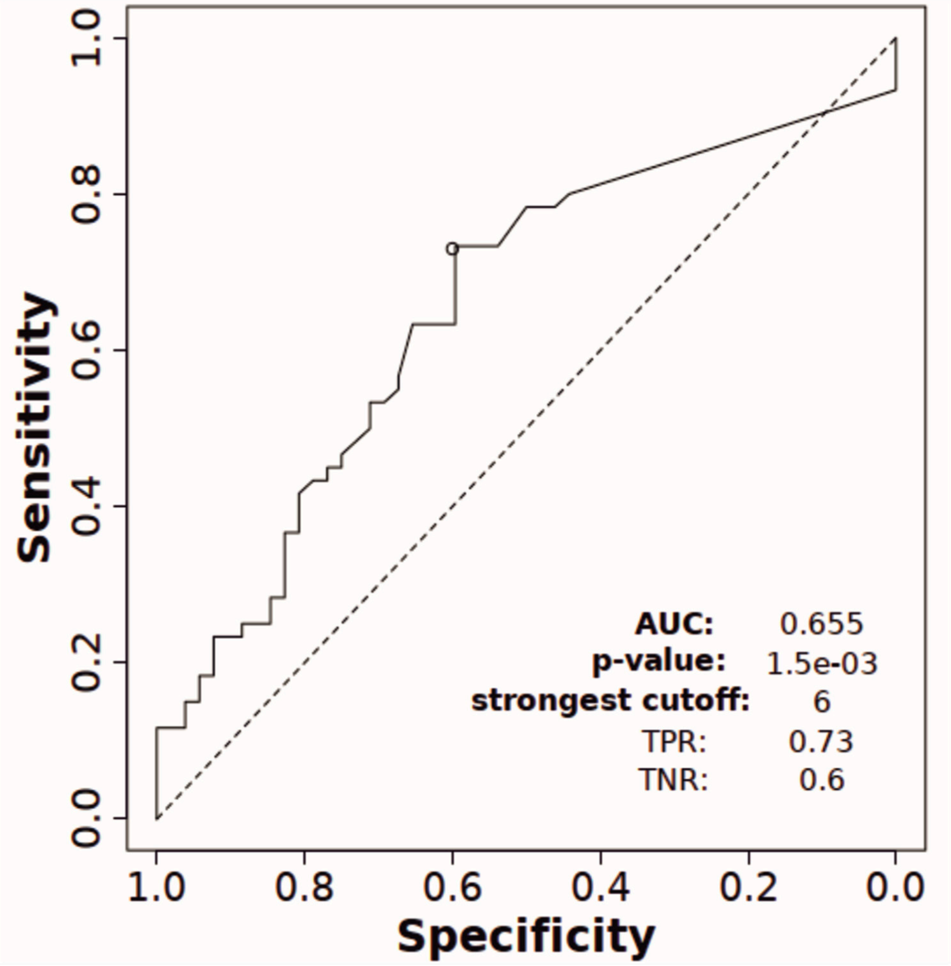

Figure S6-B. Sensitivity and specificity of a predictive model based on association of *LINC00526* gene expression levels in metastatic melanoma (61 non-responders and 55 responders) with response to inhibitor of CTLA-4 (ipilimumab). AUC-value = 0.65, Mann-Whitney test p-value = 0.0045, fold change of median gene expression values (non-responders vs responders) = 2.6. Analysis was performed using the web-based tool ROC-plotter.
