# Supplementary material for: Long Intergenic Non-Coding RNAs of Human Chromosome 18: Focus on Cancers
Source: Biomedicines. 2024 Feb 28;12(3):544. doi: 10.3390/biomedicines12030544 (PMC10968066; doi:10.3390/biomedicines12030544)
Supplement: Supplementary file 1 [file biomedicines-12-00544-s001.zip › Figure_S7_Ershov.pdf]

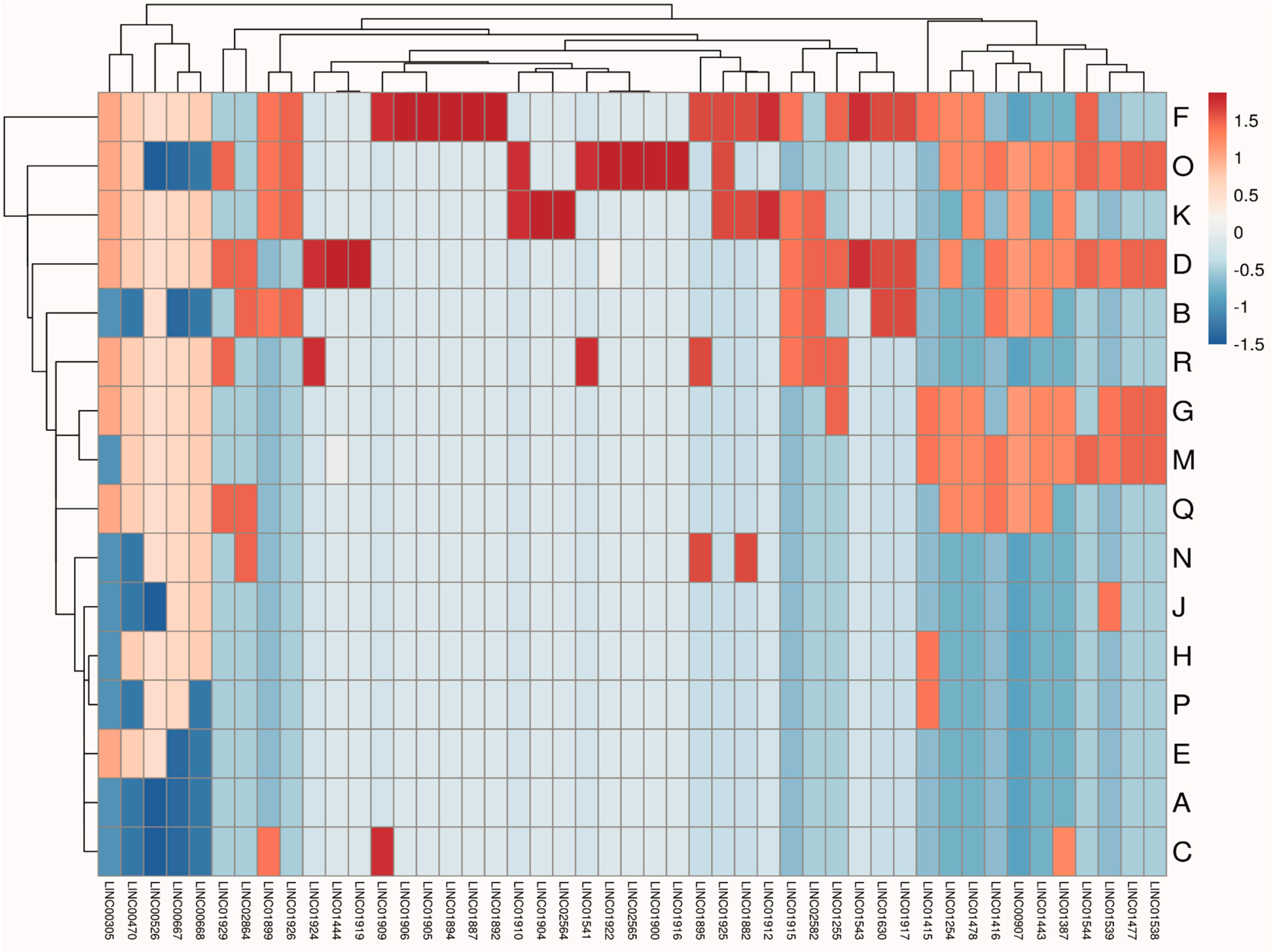

F
O
K
D
B
R
G
M
Q
N
J
H
P
E
A
C
1.5
1
0.5
0
-0.5
-1
-1.5
LINC00305
LINC00470
LINC00526
LINC00667
LINC00668
LINC01929
LINC02864
LINC01899
LINC01926
LINC01924
LINC01444
LINC01919
LINC01909
LINC01906
LINC01905
LINC01894
LINC01887
LINC01892
LINC01910
LINC01904
LINC02564
LINC01541
LINC01922
LINC02565
LINC01900
LINC01916
LINC01895
LINC01925
LINC01882
LINC01912
LINC01915
LINC02582
LINC01255
LINC01543
LINC01630
LINC01917
LINC01415
LINC01254
LINC01478
LINC01416
LINC00907
LINC01443
LINC01387
LINC01544
LINC01539
LINC01477
LINC01538

Figure S7. Clustered heat map of characteristics distribution of studied lincRNAs of human chromosome 18.
Principal component analysis (PCA) was performed using ClustVis (https://biit.cs.ut.ee/clustvis/) with following settings: pre-processing options – 'no transformation', 'row centering', 'no scaling' was applied to rows; SVD with imputation is used to calculate principal components. X- and Y-axis show PC-1 and PC-2 that explain 26.6% and 17.2% of the total variance, respectively.
Heat map options – columns are centered, no scaling is applied to columns, imputation is used for missing value estimation, rows are clustered using Manhattan distance and single linkage, and columns are clustered using Manhattan distance and average linkage.
Right color scale denotes cluster distances.

Figure legend

Hallmark symbol	Description of hallmark	Conditions
A	Somatic gene mutations	Cancer
B	Gene fusions	Cancer/Normal
C	Gene knockouts effects	Cancer/Normal
D	Copy number variations (amplifications or deletions)	Cancer
E	Differential promoter methylation	Cancer
F	Tissue-specific expression	Normal
G	Type-specific differential expression	Cancer
H	Subtype-specific differential expression	Cancer
J	Interactions with microRNAs	Cancer
K	Interactions with cellular proteins	Cancer
M	Predicted involvement in cancer-associated pathways	Cancer
N	smORF-proteins	Cancer
O	Potential prognostic value	Cancer
P	Potential predictive value	Cancer
Q	Gene expression signatures for drug predictions	Cancer
R	Literature data	Cancer
