# Supplementary material for: Long Intergenic Non-Coding RNAs of Human Chromosome 18: Focus on Cancers
Source: Biomedicines. 2024 Feb 28;12(3):544. doi: 10.3390/biomedicines12030544 (PMC10968066; doi:10.3390/biomedicines12030544)
Supplement: Supplementary file 1 [file biomedicines-12-00544-s001.zip › Table_S11_Ershov.pdf]

Table S11. Functional enrichment analysis of experimentally verified microRNAs interacting with lincRNAs of human chromosome 18.

Genes	microRNAs	Functional terms (Q-value<0.05)*
LINC00470	hsa-miR-106b-5p; hsa-miR-17-5p; hsa-miR-320c; hsa-miR-320d; hsa-miR-887-3p; hsa-miR-93-5p	positive regulation of mitotic nuclear division GO0045840; negative regulation of extrinsic apoptotic signaling pathway GO:2001240; regulation of tumor necrosis factor-mediated signaling pathway GO:0010803; positive regulation of epithelial to mesenchymal transition GO:0010718; cytokine activity GO:0005125; cytoskeleton organization GO:0007010
LINC00667	hsa-let-7b-5p; hsa-let-7g-5p; hsa-miR-105-5p; hsa-miR-106a-5p; hsa-miR-106b-5p; hsa-miR-122-5p; hsa-miR-124-3p; hsa-miR-129-2-3p; hsa-miR-142-5p; hsa-miR-148a-3p; hsa-miR-17-5p; hsa-miR-181a-5p; hsa-miR-181b-5p; hsa-miR-181d-5p; hsa-miR-196a-5p; hsa-miR-19a-3p; hsa-miR-19b-3p; hsa-miR-20a-5p; hsa-miR-26a-5p; hsa-miR-26b-5p; hsa-miR-296-3p; hsa-miR-301b-3p; hsa-miR-30a-5p; hsa-miR-30b-3p; hsa-miR-31-5p; hsa-miR-3187-3p; hsa-miR-335-5p; hsa-miR-33a-5p; hsa-miR-34a-5p; hsa-miR-3679-5p; hsa-miR-3934-5p; hsa-miR-429; hsa-miR-454-3p; hsa-miR-598-5p; hsa-miR-766-5p; hsa-miR-877-5p; hsa-miR-9-3p; hsa-miR-93-5p	positive regulation of cell migration GO:0030335; cadherin binding GO:0045296; cell surface GO:0009986; cell adhesion GO:0007155; negative regulation of apoptotic process GO:0043066; actin cytoskeleton GO:0015629; Ras signaling pathway, MAPK signaling pathway, PI3K-Akt signaling pathway, Wnt signaling pathway (KEGG**)
LINC00668	hsa-let-7a-5p; hsa-let-7b-5p; hsa-let-7c-5p; hsa-let-7d-5p; hsa-miR-98-5p; hsa-miR-103a-3p; hsa-miR-107; hsa-miR-204-3p; hsa-miR-204-5p; hsa-miR-20a-3p; hsa-miR-21-5p; hsa-miR-2114-5p; hsa-miR-2115-5p; hsa-miR-23b-3p; hsa-miR-34a-5p; hsa-miR-491-3p; hsa-miR-642b-5p; hsa-let-7e-5p	P-body GO:0000932; apoptotic process GO:0006915; chromatin remodeling GO:0006338; small cell lung cancer, breast cancer, apoptosis, hepatocellular carcinoma regulation of actin cytoskeleton, Ras signaling pathway (KEGG**)
LINC01544	hsa-miR-22-3p; hsa-miR-25-5p; hsa-miR-335-5p; hsa-miR-92b-3p	phosphatidylinositol 3-kinase activity GO:0035004; SREBP-SCAP-Insig complex GO:0032937; tubulin deacetylation GO:0090042; regulation of nucleocytoplasmic transport GO:0046822; negative regulation of protein glutathionylation GO:0010734
LINC01909	hsa-miR-518d-5p; hsa-miR-520c-5p; hsa-miR-526a-5p; hsa-miR-4698; hsa-miR-548at-3p; hsa-miR-548o-3p; hsa-miR-616-5p	G2M transition of mitotic cell cycle GO0000086; protein phosphatase 2A binding GO:0051721; translation factor activity, RNA binding GO:0008135; endosome organization GO:0007032
LINC01926	hsa-miR-183-5p; hsa-miR-29a-3p; hsa-miR-29b-3p; hsa-miR-93-5p; hsa-miR-15a-5p	cell-cell adhesion mediated by integrin GO:0033631; DNA N-glycosylase activity GO:0019104; depyrimidination GO:0045008; glucose import GO:0046323; glycogen metabolic process GO:0005977; negative regulation of mesenchymal stem cell differentiation GO:2000740; receptor catabolic process GO:0032801; mismatched DNA binding GO:0030983; D-glucose transmembrane transporter activity GO:0055056

*Over-Representation Analysis (ORA) was performed using the miRNA Enrichment and Annotation (miEAA) web-based tool v.2.1 [S1] (https://ccb-compute2.cs.uni-saarland.de/mieaa/).
**the KEGG database - Kyoto Encyclopedia of Genes and Genomes (https://www.kegg.jp/kegg/pathway.html).

References

[S1] Aparicio-Puerta E, Hirsch P, Schmartz GP, Kern F, Fehlmann T, Keller A. miEAA 2023: updates, new functional microRNA sets and improved enrichment visualizations. Nucleic Acids Res. 2023;51(W1):W319-W325.
