# Supplementary material for: Long Intergenic Non-Coding RNAs of Human Chromosome 18: Focus on Cancers
Source: Biomedicines. 2024 Feb 28;12(3):544. doi: 10.3390/biomedicines12030544 (PMC10968066; doi:10.3390/biomedicines12030544)
Supplement: Supplementary file 1 [file biomedicines-12-00544-s001.zip › Table_S12_Ershov.pdf]

Table S12. Diagnostic value* of predicted microRNAs interacting with lincRNAs

lincRNA / microRNA interaction	Sample	Cancer type	Changes	n, healthy/cancer patients
LINC00470 / hsa-miR-17-5p	Serum	Non-small-cell lung cancer	up	58/94
LINC00470 / hsa-miR-320d	Plasma	Colorectal neoplasms	down	30/80
LINC00667 / hsa-let-7b-5p	Plasma	Breast neoplasms	up	185/185
LINC00667 / hsa-let-7b-5p	Plasma	Prostatic neoplasms	down	40/152
LINC00667 / hsa-miR-122-5p	Plasma	Breast neoplasms	up	185/185
LINC00667 / hsa-miR-196a-5p	Plasma	Mouth Neoplasms	up	53/90
LINC00667 / hsa-miR-19a-3p	Blood	Colorectal neoplasms	up	53/42
LINC00667 / hsa-miR-19b-3p	Serum	Non-small-cell lung cancer	up	58/94
LINC00667 / hsa-miR-26a-5p	Plasma	Hepatocellular carcinoma	down	66/196
LINC00667 / hsa-miR-335-5p	Blood	Colorectal neoplasms	up	53/42
LINC00667 / hsa-miR-34a-5p	Serum	Colorectal neoplasms	up	28/35
LINC00668 / hsa-miR-107	Plasma	Pancreatic neoplasms	down	80/100
LINC00668 / hsa-miR-21-5p	Serum	Breast neoplasms	up	55/89
LINC00668 / hsa-let-7e-5p	Serum	Thyroid cancer	up	96/106
LINC01544 / hsa-miR-335-5p	Blood	Colorectal neoplasms	up	53/42
LINC01926 / hsa-miR-29a-3p	Plasma	Colorectal neoplasms	up	59/37
LINC01926 / hsa-miR-29b-3p	Serum	Thyroid cancer	down	41/79

*according to the PlasmiR database [S1] (https://dianalab.e-ce.uth.gr/plasmir/#/).

References

[S1] Tastsoglou S, Miliotis M, Kavakiotis I, Alexiou A, Gkotsi EC, Lambropoulou A, Lygnos V, Kotsira V, Maroulis V, Zisis D, Skoufos G, Hatzigeorgiou AG. PlasmiR: A Manual Collection of Circulating microRNAs of Prognostic and Diagnostic Value. Cancers (Basel). 2021;13(15):3680.
