# Supplementary material for: Long Intergenic Non-Coding RNAs of Human Chromosome 18: Focus on Cancers
Source: Biomedicines. 2024 Feb 28;12(3):544. doi: 10.3390/biomedicines12030544 (PMC10968066; doi:10.3390/biomedicines12030544)
Supplement: Supplementary file 1 [file biomedicines-12-00544-s001.zip › Table_S15_Ershov.pdf]

Table S15. Associations of individual lincRNAs of human chromosome 18 with pathways in cancers (predicted via pathway terms enrichment analysis of highly co-expressed genes)

Pathways / Genes	AJ	AMPK	ErbB	FA	FoxO	MAPK	mTOR	Hippo	p53	PI3K-Akt
LINC00470	CHOL<br>KIRC	CHOL<br>KIRC	CHOL<br>KIRC	CHOL<br>KIRC	CHOL<br>KIRC	CHOL<br>KIRC	CHOL<br>KIRC	KIRC	CHOL<br>KIRC	KIRC
LINC00526	CHOL<br>THYM<br>UVM	CHOL<br>KICH<br>PAAD<br>TGCT<br>THYM<br>UVM	CHOL<br>PAAD<br>TGCT<br>THYM<br>UVM	CHOL<br>PAAD<br>THYM<br>UVM	CHOL<br>KICH<br>PAAD<br>PCPG<br>TGCT<br>THYM<br>UVM	CHOL<br>PAAD<br>TGCT<br>THYM<br>UVM	CHOL<br>KICH<br>PAAD<br>PCPG<br>TGCT<br>THYM<br>UVM	CHOL<br>TGCT<br>THYM<br>UVM	THYM<br>UVM	
LINC00667	KIRC<br>LGG<br>TGCT	CHOL<br>KIRC<br>LGG<br>TGCT<br>UVM	KIRC<br>LGG<br>TGCT	KIRC<br>LGG<br>TGCT	CHOL<br>KIRC<br>LGG<br>TGCT	CHOL<br>KIRC<br>LGG<br>TGCT	CHOL<br>KIRC<br>LGG<br>TGCT	KIRC<br>LGG<br>TGCT	KIRC<br>KIRP<br>LGG<br>TGCT	KIRP<br>TGCT
LINC00668	THCA	THCA			THCA	THCA	THCA	CHOL		
LINC00907	UVM	UVM	UVM	UVM	UVM	UVM	UVM	UVM	UVM	UVM
LINC01254	GBM<br>KIRP	KIRP	KIRP	GBM<br>STAD	GBM<br>KIRP	KIRP	KIRP	KIRP	KIRP	
LINC01378							KIRC	KIRC		
LINC01415	UVM	LUAD<br>UVM	UVM	LUAD<br>UVM	UVM	UVM	UVM	UVM		
LINC01416	ESCA	ESCA	ESCA	ESCA<br>KICH	ESCA	ESCA	ESCA	ESCA	ESCA	ESCA
LINC01443				LUAD						
LINC01444			UCEC	UCEC	UCEC	UCEC		UCEC		UCEC
LINC01477	MESO			ACC						
LINC01478	LUSC	LUSC	LUSC	LUSC	LUSC	LUSC	LUSC		LUSC	LUSC
LINC01538					PAAD		PAAD			
LINC01539	TGCT			TGCT			TGCT	TGCT		TGCT
LINC01541	HNSC<br>LUAD	HNSC		HNSC<br>LUAD						HNSC
LINC01544	UVM									

*co-expressed genes with Pearson correlation coefficient $r \geq 0.7$ in different cancer types were retrieved from TCGA database using web-based tool GEPIA2; functional enrichment of gene set with KEGG pathway term was performed using web-based tool NCpath (terms were selected by adj. p-value < 0.01).

KEGG pathways terms: AJ - adherent junction, AMPK - 5' AMP-activated protein kinase signaling pathway, ErbB - ErbB signaling pathway, FA - focal adhesion, FoxO - FoxO signaling pathway, MAPK - MAPK signaling pathway, mTOR - mTOR signaling pathway, Hippo - Hippo signaling pathway, p53 – p53 signaling pathway, PI3K-Akt – signaling pathway with participation of phosphoinositide 3-kinase and Akt kinase.

ACC - Adrenocortical carcinoma,
CHOL - Cholangiocarcinoma,
ESCA - Esophageal carcinoma,
HNSC - Head and neck squamous cell carcinoma,
KIRC - Kidney renal clear cell carcinoma,
KIRP - Kidney renal papillary cell carcinoma,
KICH - Kidney chromophobe,
LGG – Low-grade glioma,
LUAD - Lung adenocarcinoma,
LUSC - Lung squamous cell carcinoma,
MESO - Mesothelioma,
PAAD - Pancreatic adenocarcinoma,
TGCT - Testicular germ cell tumors,
UCEC - Uterine Corpus Endometrial Carcinoma,
UVM - Uveal Melanoma
