# Supplementary material for: Long Intergenic Non-Coding RNAs of Human Chromosome 18: Focus on Cancers
Source: Biomedicines. 2024 Feb 28;12(3):544. doi: 10.3390/biomedicines12030544 (PMC10968066; doi:10.3390/biomedicines12030544)
Supplement: Supplementary file 1 [file biomedicines-12-00544-s001.zip › Table_S17_Ershov.pdf]

Table S17. Cancer-associated biologic processes and interactions between lincRNAs of human chromosome 18 and cellular proteins*

Cancer type	lincRNA	Protein symbol	Influenced process in cancer cells**
Gastric cancer	LINC00470	PTEN	proliferation(+); migration(+); invasion(+)
Liver carcinoma	LINC00470	ILF2	proliferation(+)
Liver carcinoma	LINC00470	ILF3	proliferation(+)
Malignant glioma	LINC00526	AXL	proliferation(+); migration(+); invasion(+); growth(+); PI3K/AKT/NF-kB signaling pathway(+)
Nephroblastoma	LINC00667	IKBKB	cancer progression(+)
Esophageal cancer	LINC00667	SLC2A3	proliferation(+); invasion(+); migration(+)
Gastric cancer	LINC00668	CDKN2B	proliferation(+)
Oral squamous cell carcinoma	LINC00668	VEGFA	cancer progression(+)
Gastric cancer	LINC00668	E2F1	proliferation(+)
Gastric cancer	LINC00668	CDKN1C	proliferation(+)
Colorectal cancer	LINC00668	USP47	proliferation(+); migration(+); apoptosis (-); malignant transformation(+)
Gastric cancer	LINC00668	SUZ12	proliferation(+)
Gastric cancer	LINC00668	EZH2	proliferation(+)
Gastric cancer	LINC00668	CDKN1B	proliferation(+)
Gastric cancer	LINC00668	CDKN2A	proliferation(+)
Non-small cell lung cancer	LINC00668	KLF7	proliferation(+); invasion(+); migration(+); apoptosis(-)
Non-small cell lung cancer	LINC00668	STAT3	proliferation(+); invasion(+); migration(+); apoptosis(-)
Breast cancer	LINC02582	CHEK1	radiosensitivity(-)

* Data was retrieved from LncBase v.3.0 [S1] (https://diana.e-ce.uth.gr/lncbasev3/interactions).
** (+) and (-) – induction and inhibition of biological process, respectively.
Abbreviations:
PTEN, phosphatase and tensin homolog;
ILF2, interleukin enhancer binding factor 2;
ILF3, interleukin enhancer binding factor 3;
AXL, AXL receptor tyrosine kinase;
IKBKB, inhibitor of nuclear factor kappa B kinase subunit beta;
SLC2A3, solute carrier family 2 member 3;
CDKN2B, cyclin dependent kinase inhibitor 2B;
VEGFA, vascular endothelial growth factor A;
E2F1, E2F transcription factor 1;
CDKN1C, cyclin dependent kinase inhibitor 1C;
USP47, ubiquitin specific peptidase 47;
SUZ12, SUZ12 polycomb repressive complex 2 subunit;
EZH2, enhancer of zeste 2 polycomb repressive complex 2 subunit;
CDKN1B, cyclin dependent kinase inhibitor 1B;
CDKN2A, cyclin dependent kinase inhibitor 2A;
KLF7, KLF transcription factor 7;
STAT3, signal transducer and activator of transcription 3;
CHEK1, checkpoint kinase 1.

References

[S1] Karagkouni D, Paraskevopoulou MD, Tastsoglou S, Skoufos G, Karavangeli A, Pierros V, Zacharopoulou E, Hatzigeorgiou AG. DIANA-LncBase v3: indexing experimentally supported miRNA targets on non-coding transcripts. Nucleic Acids Res. 2020;48(D1):D101-D110.
