# Supplementary material for: Long Intergenic Non-Coding RNAs of Human Chromosome 18: Focus on Cancers
Source: Biomedicines. 2024 Feb 28;12(3):544. doi: 10.3390/biomedicines12030544 (PMC10968066; doi:10.3390/biomedicines12030544)
Supplement: Supplementary file 1 [file biomedicines-12-00544-s001.zip › Table_S18_Ershov.pdf]

Table S18. Predicted interactors (microRNAs and RNA-binding proteins) based on the nucleotide sequences of lincRNAs' open reading frames, encoding small proteins

Genes	SmProt ID (SPROHSA…)	Length, a.a.r*	Peptide Atlas	microRNAs**	RNA-binding proteins***
LINC00526	171627	16	n/d	no	NUPL2, **PABPN1**, RBM45, **SRSF10**, **TRA2A**
	351374	19	n/d	no	**MBNL1**, PPRC1, RBFOX1, **RBM4**, RBM4B
LINC00667	381877	22	n/d	no	PABPC1, SART3
	166854	23	n/d	no	FUBP3, RBM15B, RBM41
	150256	29	n/d	no	PTB3
	46960	33	n/d	no	RBM4B, SAMD4A
	300118	39	n/d	**miR-526b**, **miR-1827**, miR-9851	**CNOT4**, **HNRNPA1**, **HNRNPL**, **HNRNPM**, HNRPLL, **IGF2BP2**, **IGF2BP3**, KHDRBS2, NUPL2, PABPC1, PABPC3, PABPC4, PABPC5, PABPN1L, SART3, **SRSF10**, YBX2
	168229	40	n/d	no	RBM4B, SAMD4A
	166850	45	n/d	no	NUPL2, PABC1, PABC3, PABC4, SART3
	244128	46	n/d	miR-4700, miR-548ad, miR-8089, miR-4667	RBM15B, RBM41
	166853	50	n/d	miR-548z, miR-548h, **miR-548d**, miR-548bb, miR-548ac	**RBM5**
	228790	60	n/d	miR-568	NUPL2, PABPC1, PABPC3, PABPC4, SART3
	271103	67	n/d	no	**EIF4G2**, FUS, HNRNPH2, **MBNL1**, **PCBP1**, **PCBP2**, PPRC1, RBM22, **RBM4**, RBM45, SAMD4A, SRSF9, SRSF8, ZC3H10
LINC00668	94911	33	n/d	no	SAMD4A, **SRSF2**, SRSF8
	74203	38	n/d	no	SAMD4A, SRSF2, SRSF8
	226469	66	n/d	**miR-6836**, miR-5006, miR-4755, miR-4446, miR-7110, miR-6829, miR-6791	NUPL2, **SRSF10**, **SRSF2**
	226470	70	n/d	**miR-6836**, miR-6829, miR-6791	SNRPA, **SRSF2**, SRSF8
	226471	86	n/d	**miR-6836**, miR-6829, miR-6791	**HNRNPU**, SAMD4A, SNRPA, **SRSF2**, SRSF8
LINC01882	392610	23	n/d	no	BRUNOL4, BRUNOL5, **RBM38**
	138350	28	n/d	no	SRSF9, YBX2
LINC01895	9535	8	n/d	no	ENOX1, SRSF10
	283247	19	n/d	no	**RBM24**
	250413	29	n/d	no	**CNOT4**
	199922	41	n/d	**miR-671**, miR-2276	**CNOT4**, **FXR1**, **LIN28A**, RBM22, **RBM25**, SNRPA, **SRSF10**, SRSF2
	206729	53	n/d	no	NUPL2
LINC02864	297396	34	n/d	no	PTB3, **PTBP3**
	194114	46	n/d	no	BRUNOL4, HNRNPF, SNRPA, **TRA2A**
	297394	55	n/d	miR-6817, miR-4769	**HNRNPH1**, **LIN28**, PABPN1, **SRSF2**, **TRA2A**

*amino acid residues;
**predicted using the miRDB portal [S1] (https://mirdb.org), target score cut-off ≥ 75;
***predicted using the RBPmap portal [S2] (http://rbpmap.technion.ac.il), p-value < 0.001;
n/d – no data.
Cancer-associated microRNAs and RBPs are highlighted with bold font.
Literature evidence of microRNAs and RNA-binding proteins (RBPs) associations with cancers was explored using MalaCards server: the human disease database [S3] (http://www.malacards.org/).
Cancer associations were selected by MalaCards InFormaTion Scores (MIFTS) > 70

References

[S1] Chen Y, Wang X. miRDB: an online database for prediction of functional microRNA targets. Nucleic Acids Res. 2020;48(D1):D127-D131.

[S2] Paz I, Argoetti A, Cohen N, Even N, Mandel-Gutfreund Y. RBPmap: A Tool for Mapping and Predicting the Binding Sites of RNA-Binding Proteins Considering the Motif Environment. Methods Mol Biol. 2022;2404:53-65.
