# Supplementary material for: Long Intergenic Non-Coding RNAs of Human Chromosome 18: Focus on Cancers
Source: Biomedicines. 2024 Feb 28;12(3):544. doi: 10.3390/biomedicines12030544 (PMC10968066; doi:10.3390/biomedicines12030544)
Supplement: Supplementary file 1 [file biomedicines-12-00544-s001.zip › Table_S1_Ershov.pdf]

Table S1. Main characteristics of lincRNAs encoded by genes of human chromosome 18

Genes	Chromosome loci	Length, nt	Transcripts*	Pathogenic gene variants*	Subcellular localization**
LINC00305	18q22.1	885	28	9	not found
LINC00470	18p11.32	2231	70	49	exosome, chromatin, cytosol, membrane, nucleolus, nucleoplasm, nucleus
LINC00526	18p11.31	1322	14	23	exosome
LINC00667	18p11.31	3979	71	17	exosome, chromatin, nucleoplasm, nucleus
LINC00668	18p11.31	1751	35	2	exosome
LINC00907	18q12.3	1865	65	4	exosome, cytosol, chromatin, membrane, nucleus
LINC01254	18p11.22	1921	4	1	not found
LINC01255	18p11.21	526	75	13	not found
LINC01387	18p11.31	603	21	4	exosome
LINC01415	18q21.2	6201	1	0	exosome
LINC01416	18q21.2	2325	6	6	nucleoplasm
LINC01443	18p11.21	1871	5	0	exosome
LINC01444	18p11.21	763	14	0	not found
LINC01477	18q12.3	1240	72	108	not found
LINC01478	18q12.3	2069	50	42	exosome
LINC01538	18q22.1	2418	8	5	exosome
LINC01539	18q21.2	2093	84	68	exosome
LINC01541	18q22.3	3123	21	0	not found
LINC01543	18q11.2	770	19	3	exosome
LINC01544	18q21.33	3217	12	1	not found
LINC01601	18q12.3	955	0	0	not found
LINC01630	18q21.2	1799	57	4	not found
LINC01882	18p11.21	574	15	1	not found
LINC01887	18p11.22	2244	34	10	not found
LINC01892	18p11.31	1207	9	0	nucleolus
LINC01894	18q11.2	1497	23	15	exosome
LINC01895	18p11.31	481	30	4	not found
LINC01897	18q21.31	297	7	2	not found
LINC01899	18q22.3	575	22	64	not found
LINC01900	18q11.2	401	12	8	not found
LINC01901	18q12.3	1115	5	2	not found
LINC01902	18q12.3	4621	0	0	not found
LINC01903	18q22.1	2147	3	0	not found
LINC01904	18p11.32	891	2	0	not found
LINC01905	18q21.2	575	82	67	exosome
LINC01906	18p11.21	477	3	11	exosome
LINC01908	18q11.2	921	34	28	not found
LINC01909	18q22.2	815	9	0	nucleus, exosome
LINC01910	18q22.2	2637	4	0	not found
LINC01912	18q22.1	1932	28	84	not found
LINC01915	18q11.2	2321	50	52	exosome
LINC01916	18q22.1	1277	3	0	not found
LINC01917	18q21.2	1037	21	234	not found
LINC01919	18q21.2	568	27	9	not found
LINC01922	18q22.3	1714	5	1	not found
LINC01924	18q22.1	1722	17	49	not found
LINC01925	18p11.32	500	8	2	not found

LINC01926	18q21.32	491	9	2	exosome
LINC01928	18p11.21	504	0	0	not found
LINC01929	18q21.2	3067	6	3	not found
LINC02564	18p11.32	826	12	6	not found
LINC02565	18q21.31	478	36	259	not found
LINC02582	18q22.3	1181	14	3	ribosome-free cytosol
LINC02837	18q21.1	2203	7	2	not found
LINC02856	18p11.22	1058	4	0	not found
LINC02864	18q22.3	2674	29	6	chromatin, cytosol, exosome, nucleolus, nucleoplasm, nucleus
LINC02879	18q12.1	1794	0	0	not found
LINC02958	18q11.2	1328	0	0	not found
LINC02974	18p11.31	463	0	0	not found
LINC02979	18p11.21	527	0	0	not found
LINC03035	18q21.2	1003	0	0	not found
LINC03069	18q21.2	2558	0	0	not found
LINC03092	18q21.2	2820	0	0	not found
LINC03110	18q21.32	249	0	0	not found
LINC03111	18q21.32	571	0	0	not found

*according to the LncBook v. 2.0 database [S1].
**according to the RNAlocate database [S2].

References

[S1] Li Z, Liu L, Feng C, Qin Y, Xiao J, Zhang Z, Ma L. LncBook 2.0: integrating human long non-coding RNAs with multi-omics annotations. Nucleic Acids Res. 2023;51(D1):D186-D191.

[S2] Zhang T, Tan P, Wang L, Jin N, Li Y, Zhang L, Yang H, Hu Z, Zhang L, Hu C, Li C, Qian K, Zhang C, Huang Y, Li K, Lin H, Wang D. RNALocate: a resource for RNA subcellular localizations. Nucleic Acids Res. 2017;45(D1):D135-D138.
