# Supplementary material for: Long Intergenic Non-Coding RNAs of Human Chromosome 18: Focus on Cancers
Source: Biomedicines. 2024 Feb 28;12(3):544. doi: 10.3390/biomedicines12030544 (PMC10968066; doi:10.3390/biomedicines12030544)
Supplement: Supplementary file 1 [file biomedicines-12-00544-s001.zip › Table_S20_Ershov.pdf]

Table S20. Anticancer drug candidates predicted by transcriptomic signatures of linc-genes encoding lincRNAs of human chromosome 18 in cancer cell lines.

Predicted drugs (p-value <0.1)	Drug concentration**	Cell line	Cancer type
*Canagliflozin**	0.25 µM	THP1	Leukemia
Conivaptan	0.03 µM	YAPC	Pancreatic carcinoma
Darapladib	0.08 µM	PC3	Prostate adenocarcinoma
Dipraglurant	0.03 µM	PC3	Prostate adenocarcinoma
Fluticasone-propionate	0.08 µM	JURKAT	Leukemia
Givinostat	0.66 µM	HAP1	Chronic myeloid leukemia
Ibrutinib	0.66 µM	TMD8	Lymphoma
Lapatinib	0.66 µM	BJAB	Lymphoma
Lucitanib	0.08 µM	PC3	Prostate adenocarcinoma
Methapyrilene	0.66 µM	HEPG2	Liver carcinoma
Primaquine	0.37 µM	HT29	Colon adenocarcinoma
Quizartinib	0.08 uM	MCF10A	Breast carcinoma
Rebastinib	0.37 µM	JURKAT	Leukemia
Talinolol	0.37 µM	MDAMB231	Breast adenocarcinoma
Tizanidine	0.12 µM	PC3	Prostate adenocarcinoma
Tozasertib	0.12 µM	OCILY19	Lymphoma

* Repositioning drugs as anti-cancer agents are highlighted with italic font;
**Cell lines were exposed to drugs for 24 h.
*** SigCom-Library web-based tool (https://maayanlab.cloud/sigcom-lincs/#/SignatureSearch/UpDown) was used to analyze transcriptomic profiling data of cancer cell lines being exposed to various concentrations of drugs.
