# Supplementary material for: Long Intergenic Non-Coding RNAs of Human Chromosome 18: Focus on Cancers
Source: Biomedicines. 2024 Feb 28;12(3):544. doi: 10.3390/biomedicines12030544 (PMC10968066; doi:10.3390/biomedicines12030544)
Supplement: Supplementary file 1 [file biomedicines-12-00544-s001.zip › Table_S21_Ershov.pdf]

Table S21. Nucleotide sequences of lincRNAs of human chromosome 18

DNA

>NR_027245.2 Homo sapiens long intergenic non-protein coding RNA 305 (LINC00305), transcript variant 2, long non-coding RNA

AATGATGGACTCTGATTTCCACACCCCTTTCCTGTGAACTTGTCTCCAATTTAGTCTGTCAAGGTCCCTTTCTTGTG
AGTTAATTTCTCCCTAGTGCTACTTCTCAGATTGGCCAGTGACTCACAGAGTGGCACAAGTACAAATCCAGCCTGCC
TTGCATCCAACACAGATTCCAGGACCACTGAAACCAGCTGAGGCATCCTGAAAGCCTAAGATTGACCACACCAGAGG
AACCAGACTGCCCTTCACAAGGATGGAAATTATCTGGTTCCACCCCTGGATATGATCAACCTTCATAGACTGTGCAT
TATACATGCCATCTCTGCGACCTGCAAAGATGAAAAGGGAAAGCAGGAGATGGAAACTGGTCAGCAGCCTTCTGGTT
TATCAGCCACACTTACAAAGGTCAAATGTGCAAAGCGTCAGAAGACAGTGGTTAGAGTGAGATTCTACATGCTCTCA
ATGAAGAATAAAGCATGCAGGAAGAACCTTTCAAAAGGTTACAACCAGAGACCTGAAGGAAGCAAGGAAGAAAGCCA
CATGGTTGTCAAAGAGAAGAGGAAAGGAGATCACTAAGCTGGATGACGTTTTCTGTTTCTTTGAAAACAGAGAAGAT
GACAAGTTAGGGAGAGATCAGAGGTCATACTGTGAAGCACCTTTGGATAAACGGCAGGGACTTTGGATTTTCTTTTT
GAATATAAGCATAATCCATTGAAAGGTCTCATGCAGGGAAGTGGCAAAACACTTTTAAAATTTTTCATCTTTTCTTT
CTGAAGCAGAGTTCACTCTCCATTTATTTGTCTTCTTTGATATCTCTCAATAGAATGTTGCAGTTTTAGTATTCTCC
TATTAAACATTTGTTTAACTTTAAAAAAAAAAAAAAAA

RNA

>NR_027245.2 Homo sapiens long intergenic non-protein coding RNA 305 (LINC00305), transcript variant 2, long non-coding RNA

AAUGAUGGACUCUGAUUUCCACACCCCUUUCCUGUGAACUUGUCUCCAAUUUAGUCUGUCAAGGUCCCUUUCUUGUG
AGUUAAUUUCUCCCUAGUGCUACUUCUCAGAUUGGCCAGUGACUCACAGAGUGGCACAAGUACAAAUCCAGCCUGCC
UUGCAUCCAACACAGAUUCCAGGACCACUGAAACCAGCUGAGGCAUCCUGAAAGCCUAAGAUUGACCACACCAGAGG
AACCAGACUGCCCUUCACAAGGAUGGAAAUUAUCUGGUUCCACCCCUGGAUAUGAUCAACCUUCAUAGACUGUGCAU
UAUACAUGCCAUCUCUGCGACCUGCAAAGAUGAAAAGGGAAAGCAGGAGAUGGAAACUGGUCAGCAGCCUUCUGGUU
UAUCAGCCACACUUACAAAGGUCAAAUGUGCAAAGCGUCAGAAGACAGUGGUUAGAGUGAGAUUCUACAUGCUCUCA
AUGAAGAAUAAAGCAUGCAGGAAGAACCUUUCAAAAGGUUACAACCAGAGACCUGAAGGAAGCAAGGAAGAAAGCCA
CAUGGUUGUCAAAGAGAAGAGGAAAGGAGAUCACUAAGCUGGAUGACGUUUUCUGUUUCUUUGAAAACAGAGAAGAU
GACAAGUUAGGGAGAGAUCAGAGGUCAUACUGUGAAGCACCUUUGGAUAAACGGCAGGGACUUUGGAUUUUCUUUUU
GAAUAUAAGCAUAAUCCAUUGAAAGGUCUCAUGCAGGGAAGUGGCAAAACACUUUUAAAAUUUUUCAUCUUUUCUUU
CUGAAGCAGAGUUCACUCUCCAUUUAUUUGUCUUCUUUGAUAUCUCUCAAUAGAAUGUUGCAGUUUUAGUAUUCUCC
UAUUAAACAUUUGUUUAACUUUAAAAAAAAAAAAAAAA

DNA

>NR_023925.1 Homo sapiens long intergenic non-protein coding RNA 470 (LINC00470), transcript variant 1, long non-coding RNA
AATGCCTTAAAGAAAGGACTGATCTCCCCTGAAGAAGAGAGAATTCTGCCTCTAGATTGCTTACAATTTG
AACTGGAACATCAGCTGTTCTCTAGGTCTTAAGTCTGCTGCTCTAACCTACAAATTTTGGACATGCCAAT
CTCCACAGTCACATGGGCCAATTCCTTAGTTTCTCTGGAGAATCCTGACTAATGTATGCAATGTCTTTAT
TTCATCCTCATTCTTAAAGGATATTTTCATCAGCAACACCACACCCTCCTGATCACCATAGCTTTATGAT
CTGAGCTTTCATCTGGTATCATTTCCTTTCCGTCTGAAGAACTTGCTTTCCTTGCAACAACAAGAAACAA
ATTTATTAGCTAACCTAACCACTAATGACGCAAGAGACAATTCTAAGGACTTTCAAAACAGCAAAGTAGG
AGCAGCTGCTACCTCTAGGGATGAGGGAGAAACTCAAAAATGCATACAAAAACCATTGCATGAAAAGTGA
CTGGATTTGTACATAGCGTCAGGAGATGTGCGTAGTGTCAAAGTATCTCATCACACATTACTTGATAATT
ACAAATGAGAAAAATGAACCTTCACAGTGGCAAGACTTGACTTCTACCTCTTTCAAAAAGATGCAATTGT
CCAATTATTGGTGAAATTGTCATTTCATGCTATTGGCTATTTGAAATTCCTCCTCTAATTTCAGAATAAA
TCACTGAAATTGACATCGGCCAGTCTGAATTTCAAGAAATTACCTGCTGAAGACAAGAGGGATCTCTTCT
TCAGATTTGCAGTCTGGGGAAGACACAGCCTCTACTGTACTTTAGAACCTGAGATATGGTGGTGGAGGGA
GCCCTGGGTCGAGTGGTAAGATTCACCCTTAGGTTAGTATTGACGTAAGGTGACGAGGAGCTGTAGACAA
AAGATTGTAACCATAAGAACTTCATAGTTTTTGTATTTTCACCGAGCTTATATTTGGTGTGTTTTTTGTC
TTTTCTTTATGATTATCAATAAAATGCTTGAAAGGAGATGAGGTTGGGGAATAATTTTTGGGAATACCAC
AAAAGACACTTTTGTGATGGAAATCCTTAAAAAGACACAATCCATTACCTCATTGGGTTCAAAAGGCAAT
TGTGAACTACTGTGGAGTTTGGAAAGAAGCAATGAGGTAATCAAGGATACTGTTGACAATCTAGCTTATC
CTATGGATGGAAGGAAATTGAAACTAATGGAGGCGAGGCTGGTAAAACAATAGGGTTTGAGACAATTCTG
TGGCATTAGAAATGAAAGAGGAAGGTGAATCCGGGAGACGGAGCTTGCAGTGAGCTGAGATCGCGCCACT
GCACTCCAGCCTGGGCGACAGAGCGAGACTCCATCTCAAAAAAAAAAAAAAAAGAAAGAAAGAAAAGAAAG
AGGAAGTAATAATCTGTGAAATTTTTCCTTAGGAACTTATTGGCAATTTAAAAATGAATTTGTTAAGCCA
TGCTGGTTCTGACCCAAAAGCCATTCCCCAGCCTTCCTCACTCCCCTCTTTCACTACTGGCAGAGATTGT
CTCTCATTTTACAAGCTGAAAATGCCAGATGCTTGCTTTTACAGTCTTCCTTACACCCAGAGCATGTGCA
TATGTTTAAACGGTCAAGAAGAAGTCATAACATGGGTGTCTGGGAGAGCTTTTATCCCACAAAAACAACA
CTTCACTCAAAAAACAAACCAAACAAAGAAAAATTCTCCTTCCTGCCATTGGATGTGAGGCTCAGACCTC
TAGTAACCATTTTGTGACCACAAAGCAACAAGCCTGAGGAAAAGTCCTACACGCTGAGCAACAGGCAGAA
ATATTGCCATCGCTGAGTTGCAGAAACAAATCTAGAGATGTTTTGCTTCTGTAATTATTTTTTATGGGAG
ATTACAAGTGGGTTTACTGTTCACTTTTCAAATCTTATTTCTCTATGATGTTTAGCTTGGGTAAATTTTA
CCTTAAATCCACTTTTTTATGTAAGGTAACATATTTGTCGGTTTCAAGGATTAAGATGTGGGCATACTTG
GAGGCCATTATTTTGCCCACCACAGGTGAAAAAGGAAGTGTTATTCTTAAATCATTTGGAAGGATCTCTG
TGTAAATGCAAGAGCGAGACAAGAAAATGCTGTCATTCTTTTGATATGGACTCGAATTTCCACTTCATGG
TTGTCTGCTTCCTTTTTAGAGTATTATTTATCCTCCTAATAAAAAGAAAGTGAAATTTCCC

RNA

>NR_023925.1 Homo sapiens long intergenic non-protein coding RNA 470 (LINC00470), transcript variant 1, long non-coding RNA
AAUGCCUUAAAGAAAGGACUGAUCUCCCCUGAAGAAGAGAGAAUUCUGCCUCUAGAUUGCUUACAAUUUGAACUGGA
ACAUCAGCUGUUCUCUAGGUCUUAAGUCUGCUGCUCUAACCUACAAAUUUUGGACAUGCCAAUCUCCACAGUCACAU
GGGCCAAUUCCUUAGUUUCUCUGGAGAAUCCUGACUAAUGUAUGCAAUGUCUUUAUUUCAUCCUCAUUCUUAAAGGA
UAUUUUCAUCAGCAACACCACACCCUCCUGAUCACCAUAGCUUUAUGAUCUGAGCUUUCAUCUGGUAUCAUUUCCUU
UCCGUCUGAAGAACUUGCUUUCCUUGCAACAACAAGAAACAAAUUUAUUAGCUAACCUAACCACUAAUGACGCAAGA
GACAAUUCUAAGGACUUUCAAAACAGCAAAGUAGGAGCAGCUGCUACCUCUAGGGAUGAGGGAGAAACUCAAAAAUG
CAUACAAAAACCAUUGCAUGAAAAGUGACUGGAUUUGUACAUAGCGUCAGGAGAUGUGCGUAGUGUCAAAGUAUCUC
AUCACACAUUACUUGAUAAUUACAAAUGAGAAAAAUGAACCUUCACAGUGGCAAGACUUGACUUCUACCUCUUUCAA
AAAGAUGCAAUUGUCCAAUUAUUGGUGAAAUUGUCAUUUCAUGCUAUUGGCUAUUUGAAAUUCCUCCUCUAAUUUCA
GAAUAAAUCACUGAAAUUGACAUCGGCCAGUCUGAAUUUCAAGAAAUUACCUGCUGAAGACAAGAGGGAUCUCUUCU
UCAGAUUUGCAGUCUGGGGAAGACACAGCCUCUACUGUACUUUAGAACCUGAGAUAUGGUGGUGGAGGGAGCCCUGG
GUCGAGUGGUAAGAUUCACCCUUAGGUUAGUAUUGACGUAAGGUGACGAGGAGCUGUAGACAAAAGAUUGUAACCAU
AAGAACUUCAUAGUUUUUGUAUUUUCACCGAGCUUAUAUUUGGUGUGUUUUUUGUCUUUUCUUUAUGAUUAUCAAUA
AAAUGCUUGAAAGGAGAUGAGGUUGGGGAAUAAUUUUUGGGAAUACCACAAAAGACACUUUUGUGAUGGAAAUCCUU
AAAAAGACACAAUCCAUUACCUCAUUGGGUUCAAAAGGCAAUUGUGAACUACUGUGGAGUUUGGAAAGAAGCAAUGA
GGUAAUCAAGGAUACUGUUGACAAUCUAGCUUAUCCUAUGGAUGGAAGGAAAUUGAAACUAAUGGAGGCGAGGCUGG
UAAAACAAUAGGGUUUGAGACAAUUCUGUGGCAUUAGAAAUGAAAGAGGAAGGUGAAUCCGGGAGACGGAGCUUGCA
GUGAGCUGAGAUCGCGCCACUGCACUCCAGCCUGGGCGACAGAGCGAGACUCCAUCUCAAAAAAAAAAAAAAAAGAAA
GAAAGAAAAGAAAGAGGAAGUAAUAAUCUGUGAAAUUUUUCCUUAGGAACUUAUUGGCAAUUUAAAAAUGAAUUUGU
UAAGCCAUGCUGGUUCUGACCCAAAAGCCAUUCCCCAGCCUUCCUCACUCCCCUCUUUCACUACUGGCAGAGAUUGU
CUCUCAUUUUACAAGCUGAAAAUGCCAGAUGCUUGCUUUUACAGUCUUCCUUACACCCAGAGCAUGUGCAUAUGUUU
AAACGGUCAAGAAGAAGUCAUAACAUGGGUGUCUGGGAGAGCUUUUAUCCCACAAAAACAACACUUCACUCAAAAAA
CAAACCAAACAAAGAAAAAUUCUCCUUCCUGCCAUUGGAUGUGAGGCUCAGACCUCUAGUAACCAUUUUGUGACCAC

AAAGCAACAAGCCUGAGGAAAAGUCCUACACGCUGAGCAACAGGCAGAAAUAUUGCCAUCGCUGAGUUGCAGAAACA
AAUCUAGAGAUGUUUUGCUUCUGUAAUUAUUUUUUAUGGGAGAUUACAAGUGGGUUUACUGUUCACUUUUCAAAUCU
UAUUUCUCUAUGAUGUUUAGCUUGGGUAAAUUUUACCUUAAAUCCACUUUUUUAUGUAAGGUAACAUAUUUGUCGGU
UUCAAGGAUUAAGAUGUGGGCAUACUUGGAGGCCAUUAUUUUGCCCACCACAGGUGAAAAAGGAAGUGUUAUUCUUA
AAUCAUUUGGAAGGAUCUCUGUGUAAAUGCAAGAGCGAGACAAGAAAAUGCUGUCAUUCUUUUGAUAUGGACUCGAA
UUUCCACUUCAUGGUUGUCUGCUUCCUUUUUAGAGUAUUAUUUAUCCUCCUAAUAAAAAGAAAGUGAAAUUUCCC

DNA

>NR_026849.1 Homo sapiens long intergenic non-protein coding RNA 526 (LINC00526), long non-coding RNA

GCGGACTCCGCGGACAAGGCCAGGCCTAGCCTTGAGCTCCGTCGGGCAGGGCCGCCGGGATTGGTGTCCG
GCAGAGCGTGAGCCCAGCGGGCTGGAGTCGGCGAGCGGGAGTGAAAGAAGAAAAGCTAGAGAGCGAAGGC
AAAGCCCGAGGAGAGAGGGCCGCGGTCGGCGGAGGAATCTGACTGCACGGTTGCGTGCGCTCACTCCCGG
AGGGCTCCCTCAGGCGGGCGGCTCCGCTAGTGGCTAAAGGCAAAGCATTCCGGGGCGCGGCGCATGAAGT
TGAGCTTCGTCCCTGCTAGCCGCCGCTTTCTCCCCAAAAATACATCCTAGCCTTAATGTTTATGCCTCCA
TTGCCCCAGTTCTTATCTGTTTTGCTCAATGTCTCATAGCTACAAGAAGGCAATTTCTGACGAAGCCCTC
CGTCCCTTCCAAATGGATTATTTTGGCGGGCTTCCACCCGGACAGTATGCCACCCGAATGACTGGACAAG
TGCACGGGAGCGGCTGTCATTTGCGGAGTGCGCCTTGCGATCTAGGCGCCTCACAGCGCAAATTATCCAG
TAATTTCTCTGAAATCGATGCTGGTTTGTTTTCCCAAGGCAAATCAGCAGCTTATACAGACATTGGGGCC
ACAAAGCCGGTGGAACAACGGGAGACGGCTTCCTGAGTGTCAGGTCCTCCAAGATGAGCTTAAACTTCGG
GTGGTGGGCAGGCTCGGTAGGCGGGAAAGGCCCGTGTCCAGATGAATGCAGTCCATGTTTATATTGATGA
AAGACACGTATACCATTTGGTCCAGCTCAACCATTCTTAACAAGTGCTGGCTGTGAGCACTGGTGTGTGT
GTGCCTGCGTGCAACCAGCAATGTAAGTGATGACCAGCTGCATGATGCCTTAATTTGAGTGGACAAAGTC
GGTCAAGAACCACTTTCAGGCTTCTGGGTCTCCAAGTCTTTGTCTCCCTGTATCCAAACGAGGCATTCCA
AGTTTGACAGCATGGAACATTGCATGTCCGAAATACCAACCGTGAAACTGGGTAAAAAGCTTCAGGAGGT
GATATTGATGTGTATAGTTTAGGAAATAAAACCCAATACTTACTTCAAAGAACGTAATTTGTGGCTTTTT
AGAGAGAAGGATACTTTGACAGTTTTCATTTGTCGTCTACATGTATATGGTACGTTTCATTCTGCCTTTT
AATTAATTTGGTATTTTACTATGAAAACTACTTTGTATTAATAAAATAATGTATTTCCAATGTTATAAGT
TGTGTTATAACAGTTTCAAAAGCCAAATAAACAAGATGCATTTTGAAAAAAAAAAAAAAAAA

RNA

>NR_026849.1 Homo sapiens long intergenic non-protein coding RNA 526 (LINC00526), long non-coding RNA

GCGGACUCCGCGGACAAGGCCAGGCCUAGCCUUGAGCUCCGUCGGGCAGGGCCGCCGGGAUUGGUGUCCGGCAGAGC
GUGAGCCCAGCGGGCUGGAGUCGGCGAGCGGGAGUGAAAGAAGAAAAGCUAGAGAGCGAAGGCAAAGCCCGAGGAGA
GAGGGCCGCGGUCGGCGGAGGAAUCUGACUGCACGGUUGCGUGCGCUCACUCCCGGAGGGCUCCCUCAGGCGGGCGG
CUCCGCUAGUGGCUAAAGGCAAAGCAUUCCGGGGCGCGGCGCAUGAAGUUGAGCUUCGUCCCUGCUAGCCGCCGCUU
UCUCCCCAAAAAUACAUCCUAGCCUUAAUGUUUAUGCCUCCAUUGCCCCAGUUCUUAUCUGUUUUGCUCAAUGUCUC
AUAGCUACAAGAAGGCAAUUUCUGACGAAGCCCUCCGUCCCUUCCAAAUGGAUUAUUUUGGCGGGCUUCCACCCGGA
CAGUAUGCCACCCGAAUGACUGGACAAGUGCACGGGAGCGGCUGUCAUUUGCGGAGUGCGCCUUGCGAUCUAGGCGC
CUCACAGCGCAAAUUAUCCAGUAAUUUCUCUGAAAUCGAUGCUGGUUUGUUUUCCCAAGGCAAAUCAGCAGCUUAUA
CAGACAUUGGGGCCACAAAGCCGGUGGAACAACGGGAGACGGCUUCCUGAGUGUCAGGUCCUCCAAGAUGAGCUUAA
ACUUCGGGUGGUGGGCAGGCUCGGUAGGCGGGAAAGGCCCGUGUCCAGAUGAAUGCAGUCCAUGUUUAUAUUGAUGA
AAGACACGUAUACCAUUUGGUCCAGCUCAACCAUUCUUAACAAGUGCUGGCUGUGAGCACUGGUGUGUGUGUGCCUG
CGUGCAACCAGCAAUGUAAGUGAUGACCAGCUGCAUGAUGCCUUAAUUUGAGUGGACAAAGUCGGUCAAGAACCACU
UUCAGGCUUCUGGGUCUCCAAGUCUUUGUCUCCCUGUAUCCAAACGAGGCAUUCCAAGUUUGACAGCAUGGAACAUU
GCAUGUCCGAAAUACCAACCGUGAAACUGGGUAAAAAGCUUCAGGAGGUGAUAUUGAUGUGUAUAGUUUAGGAAAUA
AAACCCAAUACUUACUUCAAAGAACGUAAUUUGUGGCUUUUUAGAGAGAAGGAUACUUUGACAGUUUUCAUUUGUCG
UCUACAUGUAUAUGGUACGUUUCAUUCUGCCUUUUAAUUAAUUUGGUAUUUUACUAUGAAAACUACUUUGUAUUAAU
AAAAUAAUGUAUUUCCAAUGUUAUAAGUUGUGUUAUAACAGUUUCAAAAGCCAAAUAAACAAGAUGCAUUUUGAAAA
AAAAAAAAAAAAA

DNA

>NR_015389.1 Homo sapiens long intergenic non-protein coding RNA 667 (LINC00667), long non-coding RNA

GAGCGGGGAGCAAGGCCTGCGGGGAGGCGCAGGATGGACGCGTTGGCTGTCATGATGTAGGACCTCCTGA
CCTAGAACCATGCCCGGCGCAGGGAGAACAACGAGCTTACGGACCGGGTGCGGGGGCTGCTGTGCGAGAA
AGCCTACCTGCTGGCCCGGGAGCGTTCGCCGGCCCACCTGGTGGCTTTCCCTGGACGTTTAACGGCGACT
CCGCCCGGCCTCCCGACTTTTTGATGCAGGCGTTCTCTTACATGACTTTCTTCGAGGCTAGATTCTCGAA
AGACATCCTGAAGGTGGCTTTCTTAATTAGCTGCCTCACCGGCTGGCGGAGCAGTTCGTAGTCGCCTACA
TCGAGAGGGAGAGCCCCGTCCTGGCTCAGTACTGGGGCTTCGTGGACGCGCTGTAGCGGGTCTTCGCCGT
CGTGGGTAGGAAACAGTCGGGCCGGATCCCCGGTGTCTGGGGAGCGGCTGCCGGCCGGGCCCGCGCGAGC
CGCTTCACTCCTACAAGCCCGGCTCACTAGTTCCAAGCTGCTGGACTTTGACCCTCGCTGGGAAGCTTGA
TCGCCGCCTGTTCTCGCCAATCTCTATGCCTTTGCACTGCCCAGCAACCCGGTCACTGCCCAGGACCTGT
TAAAAGTTGAATGGACCTCCCAGAATCACGCCGCTGCCACCGCCGTTTCTAGAGACGGCTTTTGGCCACC
TTTGAGAATCAGGGTCAAACTTGGAAGGGCCTGCATTTGTCACACTTCCTCGGACACTAATTTGGATAGT
TCAGGCCCAGAGACGGCTTCCCGGGTTAGTGTGCCTGACAACACTGAAGAAAAATTGTGACATTTTCCTT
CCCCAGAATTACTTCGTTTTATACATTTCACTTCTCTTCTGAAATCACAGCAATGCCAGTTTGCGCCTTT
TGGTCTGAGGAACGCAGGCCCTGGCCACTACCAGGAAAAAATCCTTTGCACATGGCCTGCTATCAACAAT
CCTCTACAATGATTCTCAGCATCACCTAGGGAGCTTATGTAAAATCTCAAATTCCTGGGCCCTATCCCCA
GAGTTTCTTATCAATTTCCTTGATGGAGAACTATCAAAGCAGTCAAAGCTATCAAAATAAAAAAGAATTC
TGATTGGGTGCAGTGGCTCACACCTGTCATTCCAGCACTTTGAGAGGCCAAGGTGGGCAGATCACATGAG
GCCAGGAGTTCAAGACCAGCCTGGCCAACATGGCAGAAACCCCATCTCTACTAAAAATACCAAAATTAGC
CGGGCATGGTGGAACACACCTGCAATCCCAGCTACTCGGGAGGCTGAGGCAAGAGAATCGCTTGAACCTG
GGAGGCAGAAGTTGCAGTGAAACAAGTTGGTGCCAATGCATTCCAGCCTGGATGATGGAGCGAGACTCTG
TCTCAAGAAAAAAAAAACAAAAACAAAAACAGAACTCTGCCTCATAGCGTCTTTTAAGTTATACACTGGA
CCTATCCTTTCCTCACTGACAGACATTTTAAAAATTTTTTGGTAAGGCCTAGTTCATATAAAATGTAATC
CAAGCCAAAAGTTAACAAGAATAAGGGGAGGAAAGGGGACTCCAATAGCAGAGAAAGGTATTTACCTGGG
ATATACACTGCAAGAAAATCAAAGCTATAAGAAACGTCCATGAATAGTAGCCATAAGGCATCAGAGTGAT
AAAATTCCTGTCCCTAGGAGGGAATATTGGAGTTTGCCAGAGAAACAGAATGAGAGAGACAGAGAGGTTT
ATTGTAGGAATTGGCTCATATGATTACGGAGGCTGAGAAGACCCACGATCTGCCATCTGCAAGCTGGAGA
ATCAGGAAAGCTGGAGGTGTAATTCAGTCAAGTCCAATGGCCAGAGAAGCAAGTGTACTGATATCCAAGA
GCAGGAGAAAATAGATGTCCCAGAACAAGCAGAGAGGCTGATTTTGTCCTTCCTCTGCCTTTTTGTTTCA
TATGGGGCACTGAATGGACTGATGCCCATCCACATTAGTGAGGGTGGATCTTCTTTACTCAGTCTACCAG
TAGAAATGTCAATGACTTCCAGAAACACCCTCACCAACACACGTGGAAATAATGTTTTACCAGGTATCTG
GGCATCCCTTGGTTCACTCAAGTTGACACAAAATTAACCATCACAGAAGGAGACTGGCCTTACTCTGAAA
TTAGGAAACTAAAGAAGTGACCAGAATGGAGACTAGGTAGAGACAACTAGTTCTCTACCAAACATGTACA
GTTATTCGTTGGTATCTGAAGGGGATTGGTTCCAGGAACTCTCAGGGATACCAAAATCTGCAGGTGCTCA
AGTCATTTATATAAAATATTACAGTATTTGCATATAACCTTTGCACATCTTCCATATACTTTAAATCATC
TCTACATTACTTATAATAATGAATGTGTAAATGCTATGAAAATAGTTACTACACTATTGTTTATTTGTAT
TTTTATTGAATTGTTTTGGGGTGGGGGGCAGCTGTATCTTTCTTAGTAATAGAACCCCTGGTTTTAGCTG
GGCACATGACTGCCCTCAATAAAGATTAAAGTACCCCAGCCTTCCTTGAGATTGTGGCCATGTGACTGAA
CTTTAGACAGTGAGATATAAGCAGATATCTTCTGTGGCAGTGTTAGGAAACTATTAAAGACAGTAAGAAC
ATTGCCCTTTGCCTTCTTTTTCCTTTCTTCATTTTTCTGCCTGGAATGCAAATGTGATGGCTAACACCCT
AGCAGCCATTTTAGATTATGAAGATGAGAGTCACTCCCCAGGAAGGGTGGACCTGAAAGCTGGATTCTAG
ATGTGGAATGACAATACCAGCCCTGGACTACCTACCTGTAGGCTGTGTGTGTGTGTGTGTGATTGACAGA
GAGAGAGAGAGATAGATACCACAAGTAACACAAAGCATCATACCTGGGACTGGGAAGGTTAATGTTTAAA
CATTTTAAATGTGGCATTGGTTTGTTGAAGACAGTCAAGCAGTAGGTGCTGCAGGCTTTGTTGAGGCTAG
AAAGCTGTTGGTCTTTATTAGGTGATGGCAAAACAATGGATCACATTTTCATGTGCTGTGTCTTGGGAGG
CAAATGATGAGAATGTAGGATTGGGGAAAATGGTAAGGAAAATCCAAAATGTTCATGTTTTCTAACCCCT
TCTTGCCAATCTTAAAAGAAGGACAAACAGGCTAGATTAAGCAAGTCTACAGTTACAATTGGAAGGAAAC
ACAGCCGTACTAAGGAAGTACTCTGTCTGTGGCCTGCAGTCTAGTTTGACTAGGTCCTGTATTTGAGCTT
TGAAAGAGTAAAGAATACAAGTGCTCGTATACCCCTGAAGTAGTTACCAGCAGTGACTGCAAGGAGGTAA
TCTTATGGGATGAAACTATAACTCTGTATTAACCATCATATATAGCATATATGTGTATATGTATGTGTGT
GTGTGTATATGTGTATATAAATATATGTGTGTGTATATACACACATGTACACACCCTACAACCCAGTGAT
TTCAAAAAATTAAACATTTGTTTAGCTCATGAATCTGTGGGTTGGGTGAACATTTCTATTGGTCTTGGCT
GGGCTCACCTGTACCCAGTCAGCTGCAGGGTCTAGCTCTTCTTATCTTGGCTGGGATCTCACACATGTCT
AGGATCAGCTGCTTTACTAGTCTAGGATTGTCTTGGCTGAGACAGGTGGACTACTCAGTTCTCCTCCACT
GCCTTATCCTTCAGCTGGCCAGCCCAGAAATGTTGCCATGTTTACAGCATAAGAGCAAGCAAGAAAGAAT
AATATGCAACCACTTTTCCAAGCCTAAGGTGAAAGGGCGGGCAGAACCTGTGGTGTTAGGAGGCAACTCA
TACCAGCCCATGGGTCTACCGTTTAATTTTCAGAAATTTGGCAAGCTGATTGTTAACACAGCTGTTGTTT
AATTTATGTACTTGTAATTAAATAATTTACATTAAAACAAAGATAATAGGTACTCAAAA

RNA

>NR_015389.1 Homo sapiens long intergenic non-protein coding RNA 667 (LINC00667), long non-coding RNA

GAGCGGGGAGCAAGGCCUGCGGGGAGGCGCAGGAUGGACGCGUUGGCUGUCAUGAUGUAGGACCUCCUGACCUAGAA
CCAUGCCCGGCGCAGGGAGAACAACGAGCUUACGGACCGGGUGCGGGGGCUGCUGUGCGAGAAAGCCUACCUGCUGG
CCCGGGAGCGUUCGCCGGCCCACCUGGUGGCUUUCCCUGGACGUUUAACGGCGACUCCGCCCGGCCUCCCGACUUUU
UGAUGCAGGCGUUCUCUUACAUGACUUUCUUCGAGGCUAGAUUCUCGAAAGACAUCCUGAAGGUGGCUUUCUUAAUU
AGCUGCCUCACCGGCUGGCGGAGCAGUUCGUAGUCGCCUACAUCGAGAGGGAGAGCCCCGUCCUGGCUCAGUACUGG
GGCUUCGUGGACGCGCUGUAGCGGGUCUUCGCCGUCGUGGGUAGGAAACAGUCGGGCCGGAUCCCCGGUGUCUGGGG
AGCGGCUGCCGGCCGGGCCCGCGCGAGCCGCUUCACUCCUACAAGCCCGGCUCACUAGUUCCAAGCUGCUGGACUUU
GACCCUCGCUGGGAAGCUUGAUCGCCGCCUGUUCUCGCCAAUCUCUAUGCCUUUGCACUGCCCAGCAACCCGGUCAC
UGCCCAGGACCUGUUAAAAGUUGAAUGGACCUCCCAGAAUCACGCCGCUGCCACCGCCGUUUCUAGAGACGGCUUUU
GGCCACCUUUGAGAAUCAGGGUCAAACUUGGAAGGGCCUGCAUUUGUCACACUUCCUCGGACACUAAUUUGGAUAGU
UCAGGCCCAGAGACGGCUUCCCGGGUUAGUGUGCCUGACAACACUGAAGAAAAAUUGUGACAUUUUCCUUCCCCAGA
AUUACUUCGUUUUAUACAUUUCACUUCUCUUCUGAAAUCACAGCAAUGCCAGUUUGCGCCUUUUGGUCUGAGGAACG
CAGGCCCUGGCCACUACCAGGAAAAAAUCCUUUGCACAUGGCCUGCUAUCAACAAUCCUCUACAAUGAUUCUCAGCA
UCACCUAGGGAGCUUAUGUAAAAUCUCAAAUUCCUGGGCCCUAUCCCCAGAGUUUCUUAUCAAUUUCCUUGAUGGAG
AACUAUCAAAGCAGUCAAAGCUAUCAAAAUAAAAAAGAAUUCUGAUUGGGUGCAGUGGCUCACACCUGUCAUUCCAG
CACUUUGAGAGGCCAAGGUGGGCAGAUCACAUGAGGCCAGGAGUUCAAGACCAGCCUGGCCAACAUGGCAGAAACCC
CAUCUCUACUAAAAAUACCAAAAUUAGCCGGGCAUGGUGGAACACACCUGCAAUCCCAGCUACUCGGGAGGCUGAGG
CAAGAGAAUCGCUUGAACCUGGGAGGCAGAAGUUGCAGUGAAACAAGUUGGUGCCAAUGCAUUCCAGCCUGGAUGAU
GGAGCGAGACUCUGUCUCAAGAAAAAAAAAAACAAAAACAAAAACAGAACUCUGCCUCAUAGCGUCUUUUAAGUUAUA
CACUGGACCUAUCCUUUCCUCACUGACAGACAUUUUAAAAAUUUUUUGGUAAGGCCUAGUUCAUAUAAAAUGUAAUC
CAAGCCAAAAGUUAACAAGAAUAAGGGGAGGAAAGGGGACUCCAAUAGCAGAGAAAGGUAUUUACCUGGGAUAUACA
CUGCAAGAAAAUCAAAGCUAUAAGAAACGUCCAUGAAUAGUAGCCAUAAGGCAUCAGAGUGAUAAAAUUCCUGUCCC
UAGGAGGGAAUAUUGGAGUUUGCCAGAGAAACAGAAUGAGAGAGACAGAGAGGUUUAUUGUAGGAAUUGGCUCAUAU
GAUUACGGAGGCUGAGAAGACCCACGAUCUGCCAUCUGCAAGCUGGAGAAUCAGGAAAGCUGGAGGUGUAAUUCAGU
CAAGUCCAAUGGCCAGAGAAGCAAGUGUACUGAUAUCCAAGAGCAGGAGAAAAUAGAUGUCCCAGAACAAGCAGAGA
GGCUGAUUUUGUCCUUCCUCUGCCUUUUUGUUUCAUAUGGGGCACUGAAUGGACUGAUGCCCAUCCACAUUAGUGAG
GGUGGAUCUUCUUUACUCAGUCUACCAGUAGAAAUGUCAAUGACUUCCAGAAACACCCUCACCAACACACGUGGAAA
UAAUGUUUUACCAGGUAUCUGGGCAUCCCUUGGUUCACUCAAGUUGACACAAAAUUAACCAUCACAGAAGGAGACUG
GCCUUACUCUGAAAUUAGGAAACUAAAGAAGUGACCAGAAUGGAGACUAGGUAGAGACAACUAGUUCUCUACCAAAC
AUGUACAGUUAUUCGUUGGUAUCUGAAGGGGAUUGGUUCCAGGAACUCUCAGGGAUACCAAAAUCUGCAGGUGCUCA
AGUCAUUUAUAUAAAAUAUUACAGUAUUUGCAUAUAACCUUUGCACAUCUUCCAUAUACUUUAAAUCAUCUCUACAU
UACUUAUAAUAAUGAAUGUGUAAAUGCUAUGAAAAUAGUUACUACACUAUUGUUUAUUUGUAUUUUUAUUGAAUUGU
UUUGGGGUGGGGGGCAGCUGUAUCUUUCUUAGUAAUAGAACCCCUGGUUUUAGCUGGGCACAUGACUGCCCUCAAUA
AAGAUUAAAGUACCCCAGCCUUCCUUGAGAUUGUGGCCAUGUGACUGAACUUUAGACAGUGAGAUAUAAGCAGAUAU
CUUCUGUGGCAGUGUUAGGAAACUAUUAAAGACAGUAAGAACAUUGCCCUUUGCCUUCUUUUUCCUUUCUUCAUUUU
UCUGCCUGGAAUGCAAAUGUGAUGGCUAACACCCUAGCAGCCAUUUUAGAUUAUGAAGAUGAGAGUCACUCCCCAGG
AAGGGUGGACCUGAAAGCUGGAUUCUAGAUGUGGAAUGACAAUACCAGCCCUGGACUACCUACCUGUAGGCUGUGUG
UGUGUGUGUGUGAUUGACAGAGAGAGAGAGAGAUAGAUACCACAAGUAACACAAAGCAUCAUACCUGGGACUGGGAA
GGUUAAUGUUUAAACAUUUUAAAUGUGGCAUUGGUUUGUUGAAGACAGUCAAGCAGUAGGUGCUGCAGGCUUUGUUG
AGGCUAGAAAGCUGUUGGUCUUUAUUAGGUGAUGGCAAAACAAUGGAUCACAUUUUCAUGUGCUGUGUCUUGGGAGG
CAAAUGAUGAGAAUGUAGGAUUGGGGAAAAUGGUAAGGAAAAUCCAAAAUGUUCAUGUUUUCUAACCCCUUCUUGCC
AAUCUUAAAAGAAGGACAAACAGGCUAGAUUAAGCAAGUCUACAGUUACAAUUGGAAGGAAACACAGCCGUACUAAG
GAAGUACUCUGUCUGUGGCCUGCAGUCUAGUUUGACUAGGUCCUGUAUUUGAGCUUUGAAAGAGUAAAGAAUACAAG
UGCUCGUAUACCCCUGAAGUAGUUACCAGCAGUGACUGCAAGGAGGUAAUCUUAUGGGAUGAAACUAUAACUCUGUA
UUAACCAUCAUAUAUAGCAUAUAUGUGUAUAUGUAUGUGUGUGUGUAUAUGUGUAUAUAAAUAUAUGUGUGUGUA
UAUACACACAUGUACACACCCUACAACCCAGUGAUUUCAAAAAAUUAAACAUUUGUUUAGCUCAUGAAUCUGUGGGU
UGGGUGAACAUUUCUAUUGGUCUUGGCUGGGCUCACCUGUACCCAGUCAGCUGCAGGGUCUAGCUCUUCUUAUCUUG
GCUGGGAUCUCACACAUGUCUAGGAUCAGCUGCUUUACUAGUCUAGGAUUGUCUUGGCUGAGACAGGUGGACUACUC
AGUUCUCCUCCACUGCCUUAUCCUUCAGCUGGCCAGCCCAGAAAUGUUGCCAUGUUUACAGCAUAAGAGCAAGCAAG
AAAGAAUAAUAUGCAACCACUUUUCCAAGCCUAAGGUGAAAGGGCGGGCAGAACCUGUGGUGUUAGGAGGCAACUCA
UACCAGCCCAUGGGUCUACCGUUUAAUUUUCAGAAAUUUGGCAAGCUGAUUGUUAACACAGCUGUUGUUUAAUUUAU
GUACUUGUAAUUAAAUAAUUUACAUUAAAACAAAGAUAAUAGGUACUCAAAA

DNA
>NR_034100.1 Homo sapiens long intergenic non-protein coding RNA 668 (LINC00668), long non-coding RNA
GGGGCGGCCTGGGACTCGGGGGCGGGGTCAGTCATATAAGGCTGTGCCCAGCGCTTTTGGAAGCAGTAAG
TCCAGCCCGAGGCTAAGGAGGTGTTAACCACCGAAGGGAGGTAGAATGTTTTTCCCCACCAGAGGAGGCA
GCGACCACGTCTCCTCTATGGAGGCATTCAAGAGCCGTCCAGCTGAAGCAGCATCACTGTCTGAGCTCGG
AAGGCACAATCCACATAGGTCTGCATGGTCCACCGAGCTGCATACCCACGGGGCCAGCGGGAGGTGGGCA
GCTGCTGGGCTCTCTTCTGAAGCAGACAGGATCTCACTCTGTTGCTGAGGCTGGATCACAGCTCCCTGCA
ACCTTGAACTCTCCCTCAAGCAATTCTCCCCACTCTGCCTTCCAAAGCACTAGCATTATAGGCCTAAGCC
ACCACTCCCATCCACTGTAGTGTAAACTGTCTCCTTCAATGTTTCCAATAGTTGCGGAGCAGATCAGATA
AGGGTTCTTCCTGTCTGTTGCTTCAAGTTTCATTCTCTCTTTAAACAATACAAGGTTGGCTTCCATGGTT
CCTTCTTAAAGAATGTTGAAGGTGTGTCTTCAGATTCATTTAGTGTTCGTGGAACCCCAGGGAAAGCTGA
TGTAAAAACCTCTTTTTTCTCCCATATGTCTCAAAAAGTTGTATTTTCTGGGTCCAAGGGATCTGCAAGC
CTCCTAAAGGCATTTCCATTGTCACTACCACCAGGTGTGAACTGTAATCTGGCACGTATAGTTCCAAGAA
CTGTCATAATAGATGCTGAAGAAACATTGTGAAGTTAACTCGCTGTTACCAACTGTGAAGTCATTAGCTA
GAGGAATCTTGGGCGGTCTGAAATCTGAGATACTGTGGAAAGAACAGAAAGATCCTGTATCTTTCCTATA
ATTGTTCTACTGGAAGTTGTCATTTTACACAGGAGACATTCTGTTTTATTTATTTTCTTTTGAGACAGGG
TCTCACTCTGTCGCCCAGGCTGGAGTGCAGTGTTGTTACCACAGGTCACTGGTGCCTCCACTGCCTGAGG
ACTGCTCAAGCAATCCTCCTGCCTCAGTCCCCTGAGGAGCTGGGGCCACAGGCCTGCACCACCATGCCTG
GCTAATTATTTTTATTTGTAGAGACAAAGTCTATCTTGCCTAGGCTGATCTCGAACTCCTGGGCTCAAGT
AATCCTCCCGCGGTGGCTTCTCAAAGTGCTTGGGATTACAGATGGTCTTGCTCTGTCACCCAGTCTGGAA
TGCAGTGGTGCCATCATAGCTCACTACAGCCTGAGCTGTACTGAGCTCGAGGGATCTTCCCGTCTCCGCC
TCCTAAGCAGCTGGGACTACAGGTAATGGAAGTGGGCAGAGAATGATCCTTTCCCTAGGCTCCAGTGTGT
AAGAATTAAAGAAAGAGGAAAGAAACACAATGGCTTGATGGTCGAGGACAGGTTTATTTTAAAGAAAACA
AACCCAAGAGTGGCTTCTGGCTGAGTTAGATCAGAGAAGAGAAGTGATTTTCTTGAAATGCATGAGGCTG
GAAAGGGAGCTGGCACTTAAAGTGGCGGTGTTTGTCCGAGAGGATGGTGCTCCTGCTCTGTCACAGTGGG
CATGGGGATTCAGGCCACTGCCACCGAATTTCAAGAGCCCAAGCACAGAGCCCAGAACTAAATTGTGAGG
GTTTAAAAAAAAAAAAAGCTTTATTGAAGTATAATTGACATACAATAAACTTGAATATTTAAAGTGTACA
A

RNA
>NR_034100.1 Homo sapiens long intergenic non-protein coding RNA 668 (LINC00668), long non-coding RNA
GGGGCGGCCUGGGACUCGGGGGCGGGGUCAGUCAUAUAAGGCUGUGCCCAGCGCUUUUGGAAGCAGUAAGUCCAGCC
CGAGGCUAAGGAGGUGUUAACCACCGAAGGGAGGUAGAAUGUUUUUCCCCACCAGAGGAGGCAGCGACCACGUCUCC
UCUAUGGAGGCAUUCAAGAGCCGUCCAGCUGAAGCAGCAUCACUGUCUGAGCUCGGAAGGCACAAUCCACAUAGGUC
UGCAUGGUCCACCGAGCUGCAUACCCACGGGGCCAGCGGGAGGUGGGCAGCUGCUGGGCUCUCUUCUGAAGCAGACA
GGAUCUCACUCUGUUGCUGAGGCUGGAUCACAGCUCCCUGCAACCUUGAACUCUCCCUCAAGCAAUUCUCCCCACUC
UGCCUUCCAAAGCACUAGCAUUAUAGGCCUAAGCCACCACUCCCAUCCACUGUAGUGUAAACUGUCUCCUUCAAUGU
UUCCAAUAGUUGCGGAGCAGAUCAGAUAAGGGUUCUUCCUGUCUGUUGCUUCAAGUUUCAUUCUCUCUUUAAACAAU
ACAAGGUUGGCUUCCAUGGUUCCUUCUUAAAGAAUGUUGAAGGUGUGUCUUCAGAUUCAUUUAGUGUUCGUGGAACC
CCAGGGAAAGCUGAUGUAAAAACCUCUUUUUUCUCCCAUAUGUCUCAAAAAGUUGUAUUUUCUGGGUCCAAGGGAUC
UGCAAGCCUCCUAAAGGCAUUUCCAUUGUCACUACCACCAGGUGUGAACUGUAAUCUGGCACGUAUAGUUCCAAGAA
CUGUCAUAAUAGAUGCUGAAGAAACAUUGUGAAGUUAACUCGCUGUUACCAACUGUGAAGUCAUUAGCUAGAGGAAU
CUUGGGCGGUCUGAAAUCUGAGAUACUGUGGAAAGAACAGAAAGAUCCUGUAUCUUUCCUAUAAUUGUUCUACUGGA
AGUUGUCAUUUUACACAGGAGACAUUCUGUUUUAUUUAUUUUCUUUUGAGACAGGGUCUCACUCUGUCGCCCAGGCU
GGAGUGCAGUGUUGUUACCACAGGUCACUGGUGCCUCCACUGCCUGAGGACUGCUCAAGCAAUCCUCCUGCCUCAGU
CCCCUGAGGAGCUGGGGCCACAGGCCUGCACCACCAUGCCUGGCUAAUUAUUUUUAUUUGUAGAGACAAAGUCUAUC
UUGCCUAGGCUGAUCUCGAACUCCUGGGCUCAAGUAAUCCUCCCGCGGUGGCUUCUCAAAGUGCUUGGGAUUACAGA
UGGUCUUGCUCUGUCACCCAGUCUGGAAUGCAGUGGUGCCAUCAUAGCUCACUACAGCCUGAGCUGUACUGAGCUCG
AGGGAUCUUCCCGUCUCCGCCUCCUAAGCAGCUGGGACUACAGGUAAUGGAAGUGGGCAGAGAAUGAUCCUUUCCCU
AGGCUCCAGUGUGUAAGAAUUAAAGAAAGAGGAAAGAAACACAAUGGCUUGAUGGUCGAGGACAGGUUUAUUUUAAA
GAAAACAAACCCAAGAGUGGCUUCUGGCUGAGUUAGAUCAGAGAAGAGAAGUGAUUUUCUUGAAAUGCAUGAGGCUG
GAAAGGGAGCUGGCACUUAAAGUGGCGGUGUUUGUCCGAGAGGAUGGUGCUCCUGCUCUGUCACAGUGGGCAUGGGG
AUUCAGGCCACUGCCACCGAAUUUCAAGAGCCCAAGCACAGAGCCCAGAACUAAAUUGUGAGGGUUUAAAAAAAAAA
AAAGCUUUAUUGAAGUAUAAUUGACAUACAAUAAACUUGAAUAUUUAAAGUGUACAA

DNA

>Homo sapiens long intergenic non-protein coding RNA 907(LINC00907), transcript variant 1, long non-coding RNA.

GCACTTCTCTCTTCTGCCTCCTAGTGAAAAAGGTGCCTGCTTCTCCTTCTGCCATGATTATAAGTTTCCTGAGGCTT
CCCCAGCCATGCGTAACTGAGTGAGGACCTAGAATTTTAATGTCTAATATGCGGTGTGAATTAAAGAAGCGGCCTTG
CTATGCTGACCTTTTCAATATTCTAATCTTTGCTTTGCTGACCTCTCAGGCCTCTTCCTGGCAACAGTTAGGCTTTT
GAGAGGAGAGAGAGCTGAGACTGTTGGCTCCTTCCCATTTATGAGAGAGCCACATGACATCAGCCCATAGACTGTGG
AGAGTATTCCAGATCTATCACATTCTGCGATTCTGCGATTCCCAGAAGGCTGGTCATGAAAAAATTCCCTCTTCAGT
TTACATATATGGCAGTGCTCCAGTGCCATGACAGTTTTAATCTTCTGGTTCCATCTGCATAGATTTCAGTCTCTAAT
TGAGGAAAATCATCTGTTTTTCTCAGGAGCCCCTTGATTCAAGTTTACACAAAATGTGCCTTTGGAGACTATCCTGT
TAATTTAAACAGTTCAGATCCCACTTGTATGTGAGACAGATGCTTCTCTGGCCATCAGCCAGGTACCACTAAAGGAG
GTACAGGCACTTCTAGATATCTGTTGAGGCCTGGAAGGGGCAAAACAATTCAGGAAGCAGTTGAAGATAAAGGGGTT
TACCATGGAAGAGAAGATCCAGGTGCCTTCATGGAAATGCTTGTGGTTGAAAAGAGATGAAGATGAGGGTCCCTGGA
AAGAGGAAGAACCAGCCAGGAAAATTATACCATTAGCAAGACTGGCATCAGCAAGTAGTTCGCAGTGTTGGTTGTTC
TTGGTTTCGCAGGATTTTATAGGAGATGGACCTTCTCCTAAAAGTCTTATCTTCTAAGTCTCCTAAGTCTTGGACTT
TTCAAGGCCAAAGATTTAAAGGCCAAGTGGACCATACGGGAGATCTAGTTTCCCATCCAGACAGCCAAGCTCATGCA
TCCCTGGATTTGGATGGATGGTTTCCCATGGGGCCTTGGACAGATTGGACCCAGCACTGGCACAGGGTTGACTTGGA
GGAATAAGCTCCTGGAAACCTCAATTAGCGTTTGGGGTATGAACATTAAACAATACTGAATCTGGAAACAATACATC
ACACAGCGGGTCCAAGGAAATAAGCAGAAGGCTGGCAGCAGGCCAGGGCCAGGGGCCAAATCGCTCCTCAAAGACAA
TTCTTCATGGAGAAATGACAGCAGGTTTGAGGACAGCAGAATTTCGCTCTTGTTGCCCAGGGCTGGAGTGCAATGGT
GCAATCTCGGCTCACCACAACCACTGCCTCCTGGGTTCAAGCGATTCTCCTGCCTCAGCCTCCTGAGTAGCTGGGAT
TACAGGAAGTACAAAAACATCCTTGCCGTTTGCTGGAATGATGTACTGCTGAAGGCAATGGACACTGGGGTTGTTTG
TCCCTAATCACAAGAACCTCAGTAAACATCAGGCACTCAGTGCTCTCCAGACTGCAGTGGTGGTGGAGCTGAGAGGA
CTGTGTTTGGAGGAGTAAATGTCCACTCTCATGCTATTTTATTTCTCAGGCCCTTTGTCATGGCCTTCTTAGTGGTA
AGATCTGGAAGGGTACAATATGGTGATAGATGTAACTTTTGCTTTTGAGAGGTAAGGTGAAGTGAGGCCTGAGTAAA
GATTCACCAGTTTAGGAAGACATTGCAGTTTGTTTCTATTTGTTCAATTCAGTTTACTAGGTTAGAGAAGAAGATAG
AGACTTCAAGCAAGGCCTGGCACATAAATCTTCCCTTGGCTAAATAGTCAATCATTAAAATCTCTCTTGAGGATGTA
AAAAAAAAAAAAAAAAA

RNA

>Homo sapiens long intergenic non-protein coding RNA 907(LINC00907), transcript variant 1, long non-coding RNA.

GCACUUCUCUCUUCUGCCUCCUAGUGAAAAAGGUGCCUGCUUCUCCUUCUGCCAUGAUUAUAAGUUUCCUGAGGCUU
CCCCAGCCAUGCGUAACUGAGUGAGGACCUAGAAUUUUAAUGUCUAAUAUGCGGUGUGAAUUAAAGAAGCGGCCUUG
CUAUGCUGACCUUUUCAAUAUUCUAAUCUUUGCUUUGCUGACCUCUCAGGCCUCUUCCUGGCAACAGUUAGGCUUUU
GAGAGGAGAGAGAGCUGAGACUGUUGGCUCCUUCCCAUUUAUGAGAGAGCCACAUGACAUCAGCCCAUAGACUGUGG
AGAGUAUUCCAGAUCUAUCACAUUCUGCGAUUCUGCGAUUCCCAGAAGGCUGGUCAUGAAAAAAUUCCCUCUUCAGU
UUACAUAUAUGGCAGUGCUCCAGUGCCAUGACAGUUUUAAUCUUCUGGUUCCAUCUGCAUAGAUUUCAGUCUCUAAU
UGAGGAAAAUCAUCUGUUUUUCUCAGGAGCCCCUUGAUUCAAGUUUACACAAAAUGUGCCUUUGGAGACUAUCCUGU
UAAUUUAAACAGUUCAGAUCCCACUUGUAUGUGAGACAGAUGCUUCUCUGGCCAUCAGCCAGGUACCACUAAAGGAG
GUACAGGCACUUCUAGAUAUCUGUUGAGGCCUGGAAGGGGCAAAACAAUUCAGGAAGCAGUUGAAGAUAAAGGGGUU
UACCAUGGAAGAGAAGAUCCAGGUGCCUUCAUGGAAAUGCUUGUGGUUGAAAAGAGAUGAAGAUGAGGGUCCCUGGA
AAGAGGAAGAACCAGCCAGGAAAAUUAUACCAUUAGCAAGACUGGCAUCAGCAAGUAGUUCGCAGUGUUGGUUGUUC
UUGGUUUCGCAGGAUUUUAUAGGAGAUGGACCUUCUCCUAAAAGUCUUAUCUUCUAAGUCUCCUAAGUCUUGGACUU
UUCAAGGCCAAAGAUUUAAAGGCCAAGUGGACCAUACGGGAGAUCUAGUUUCCCAUCCAGACAGCCAAGCUCAUGCA
UCCCUGGAUUUGGAUGGAUGGUUUCCCAUGGGGCCUUGGACAGAUUGGACCCAGCACUGGCACAGGGUUGACUUGGA
GGAAUAAGCUCCUGGAAACCUCAAUUAGCGUUUGGGGUAUGAACAUUAAACAAUACUGAAUCUGGAAACAAUACAUC
ACACAGCGGGUCCAAGGAAAUAAGCAGAAGGCUGGCAGCAGGCCAGGGCCAGGGGCCAAAUCGCUCCUCAAAGACAA
UUCUUCAUGGAGAAAUGACAGCAGGUUUGAGGACAGCAGAAUUUCGCUCUUGUUGCCCAGGGCUGGAGUGCAAUGGU
GCAAUCUCGGCUCACCACAACCACUGCCUCCUGGGUUCAAGCGAUUCUCCUGCCUCAGCCUCCUGAGUAGCUGGGAU
UACAGGAAGUACAAAAACAUCCUUGCCGUUUGCUGGAAUGAUGUACUGCUGAAGGCAAUGGACACUGGGGUUGUUUG
UCCCUAAUCACAAGAACCUCAGUAAACAUCAGGCACUCAGUGCUCUCCAGACUGCAGUGGUGGUGGAGCUGAGAGGA
CUGUGUUUGGAGGAGUAAAUGUCCACUCUCAUGCUAUUUUAUUUCUCAGGCCCUUUGUCAUGGCCUUCUUAGUGGUA
AGAUCUGGAAGGGUACAAUAUGGUGAUAGAUGUAACUUUUGCUUUUGAGAGGUAAGGUGAAGUGAGGCCUGAGUAAA
GAUUCACCAGUUUAGGAAGACAUUGCAGUUUGUUUCUAUUUGUUCAAUUCAGUUUACUAGGUUAGAGAAGAAGAUAG

AGACUUCAAGCAAGGCCUGGCACAUAAAUCUUCCCUUGGCUAAAUAGUCAAUCAUUAAAAUCUCUCUUGAGGAUGUA
AAAAAAAAAAAAAAAAA

DNA
>NR_110775.1 Homo sapiens long intergenic non-protein coding RNA 1254 (LINC01254), long non-coding RNA
GCCTTCTTGGCCGAGTGGTGGCATTCTGCCTGCCCCCTGTGGGCATCATTGAGATTCTGCTCCCCAGCTT
CCGCAGTGCTCCGTTTTGTGATGGAAAGTCAGCATCCCTTGCCTTAGGAGAAACCCAGGTATCTGGTGAT
GGAAGAACTTTTGAGGGAAGCTTGAGACTGGCCAGTCCACACTCTGCTTAACCAAGGGAAGGGCACCACT
TCTACCACCAGGAGATACCTGCTGTGGGACACAGTGCTGCTCCAGCCATGCAGAAGCTCACACAGGCCTG
CAGCAGTTATTCATCCGTGCACAGCTGCCACTGGCCAGCAGAGCCTGTGACGGGGCCTTCACAGGTAATT
CCCAAGATCAAAACAGCCTCTTGTACTTAAAATAATTGCTTTGCCACCATTTCCAAGATGCTGCAGAGTG
CTTCTGAATTCTGTTCATTTCAAAGAACCCACTTCCTCTATTTGAAAAACGGGTATTTTAATGTCTGCCA
GGCCACAATTCCATGAGGCATGATGGTGTGCAGAATGTTCTGGAGATTTCCATAAGAAAGATCTCTAAGG
GCCAGAACTGACTAAAGTCATGTAGGCCACCTCTAAAAGAGGCAAGGCTCAAAGATAAATACTAGCATCA
AAAGAGAGGTCCTTTGGTGTGATATGCCCCATGTCATTCAGCTTTGTTGTGGCCAGTCATCGTTGGTGGC
AGCTGACTGGTGGCAGAATCACACCCACTGTGTGATTCTCTGCAGAAGGCTGGTGATTTGAGGATGGACA
GGAAGTTCTGAACTCAGGAATACCATGCATTTTCACAAGCGTTTTCACTTTGCCTTAAGATTTGTAGAAG
AGTTAAGTTGGGATCCCATCATCAGCAAAACCTAACACCACCATCCTGAGATAAGCTAAGAAAAGCTAAG
AAAAAGATAAGCTGAGAAAAGCTAAGGAAAGATTTGTCAACCTTTCTTCTAGATCAGAACAGCAAACGTG
ATGCAGGATTTCCTGAGGTTGGGACCGGACCGAAACGTTTTCCATCTTCTATTTTTGTTTACTCAGCTTC
AACTGCCACCTTTTATGCTATCCTGGGGGCAAGGGGGCACCCATGTGAATGAGAGATACTGGATTTGGCC
AAGATCATTAGGCAAGGGTGCCGTTCCCACACAGTAATGCCTTGCAGGCACAAGGATGGAAAATTATGCC
AGGAATGCGATACCGCCAAGCCTGAACACAGAGAGGACACGACGTTGGCACAGGCCTGTTGGGTCTTGAC
TTCAGCCTGCAGAGGAGCTGGAGGAGGCTGGCAGACTCTGCTCACAAATGGGAAGGCCCAGCTGCCAATG
GCTTTTCCTCTCTGCCCATGTAACACTTTTTGTGGGGAATGTGTGGACACAGCTTGAGGCTTTGTATCCA
CCCTGTCAGTTGGTGAGATAGAGAAGAGATGGGCTGATGGTTATTGAGGCACTACTGCTCAGATGGGGCA
TTGTTGGAGCCCATAGATGGGGCATTGTTGGAGTTGTAATGAGAGAAGTTGCTAGTGGCCCCTGGAGCAA
GACACCTGGAGCTTCTATTCAGCTTGCTCTTCTTCAACATCCATGCTTCTGGAACTTGCATTCCTCACAG
TGAAGGATGGAAGCCCTTTGGCCTTGCCCTCCCGCCCAGCTGATGGGATGAGAGGCAGATGCAGGCAGCT
GTCAGGAGAGCAAAGGGAAGGGAGATACTGGGTCCGGAAACCCTGCTGCTGCTGCGACAAGTTCTCCGGG
TCCAGGCTGTGATTTCCACAATCTGCATCCAAACCACAGAGTGAAGCCGAAATTGTGTGAATGATCGGGA
GTGTTTGCAGAGTTCAACCACAGTGTTATCACGTTTTATCCAAATCTCCTAGTCCAAAAAAGAATACCAC
GCCCTGTTTTTATCTCAAAAAAAAAAAAAAA

RNA
>LINC01254
GCCUUCUUGGCCGAGUGGUGGCAUUCUGCCUGCCCCCUGUGGGCAUCAUUGAGAUUCUGCUCCCCAGCUU
CCGCAGUGCUCCGUUUUGUGAUGGAAAGUCAGCAUCCCUUGCCUUAGGAGAAACCCAGGUAUCUGGUGAU
GGAAGAACUUUUGAGGGAAGCUUGAGACUGGCCAGUCCACACUCUGCUUAACCAAGGGAAGGGCACCACU
UCUACCACCAGGAGAUACCUGCUGUGGGACACAGUGCUGCUCCAGCCAUGCAGAAGCUCACACAGGCCUG
CAGCAGUUAUUCAUCCGUGCACAGCUGCCACUGGCCAGCAGAGCCUGUGACGGGGCCUUCACAGGUAAUU
CCCAAGAUCAAAACAGCCUCUUGUACUUAAAAUAAUUGCUUUGCCACCAUUUCCAAGAUGCUGCAGAGUG
CUUCUGAAUUCUGUUCAUUUCAAAGAACCCACUUCCUCUAUUUGAAAAACGGGUAUUUUAAUGUCUGCCA
GGCCACAAUUCCAUGAGGCAUGAUGGUGUGCAGAAUGUUCUGGAGAUUUCCAUAAGAAAGAUCUCUAAGG
GCCAGAACUGACUAAAGUCAUGUAGGCCACCUCUAAAAGAGGCAAGGCUCAAAGAUAAAUACUAGCAUCA
AAAGAGAGGUCCUUUGGUGUGAUAUGCCCCAUGUCAUUCAGCUUUGUUGUGGCCAGUCAUCGUUGGUGGC
AGCUGACUGGUGGCAGAAUCACACCCACUGUGUGAUUCUCUGCAGAAGGCUGGUGAUUUGAGGAUGGACA
GGAAGUUCUGAACUCAGGAAUACCAUGCAUUUUCACAAGCGUUUUCACUUUGCCUUAAGAUUUGUAGAAG
AGUUAAGUUGGGAUCCCAUCAUCAGCAAAACCUAACACCACCAUCCUGAGAUAAGCUAAGAAAAGCUAAG
AAAAAGAUAAGCUGAGAAAAGCUAAGGAAAGAUUUGUCAACCUUUCUUCUAGAUCAGAACAGCAAACGUG
AUGCAGGAUUUCCUGAGGUUGGGACCGGACCGAAACGUUUUCCAUCUUCUAUUUUUGUUUACUCAGCUUC
AACUGCCACCUUUUAUGCUAUCCUGGGGGCAAGGGGGCACCCAUGUGAAUGAGAGAUACUGGAUUUGGCC
AAGAUCAUUAGGCAAGGGUGCCGUUCCCACACAGUAAUGCCUUGCAGGCACAAGGAUGGAAAAUUAUGCC
AGGAAUGCGAUACCGCCAAGCCUGAACACAGAGAGGACACGACGUUGGCACAGGCCUGUUGGGUCUUGAC
UUCAGCCUGCAGAGGAGCUGGAGGAGGCUGGCAGACUCUGCUCACAAAUGGGAAGGCCCAGCUGCCAAUG
GCUUUUCCUCUCUGCCCAUGUAACACUUUUUGUGGGGAAUGUGUGGACACAGCUUGAGGCUUUGUAUCCA
CCCUGUCAGUUGGUGAGAUAGAGAAGAGAUGGGCUGAUGGUUAUUGAGGCACUACUGCUCAGAUGGGGCA
UUGUUGGAGCCCAUAGAUGGGGCAUUGUUGGAGUUGUAAUGAGAGAAGUUGCUAGUGGCCCCUGGAGCAA
GACACCUGGAGCUUCUAUUCAGCUUGCUCUUCUUCAACAUCCAUGCUUCUGGAACUUGCAUUCCUCACAG
UGAAGGAUGGAAGCCCUUUGGCCUUGCCCUCCCGCCCAGCUGAUGGGAUGAGAGGCAGAUGCAGGCAGCU
GUCAGGAGAGCAAAGGGAAGGGAGAUACUGGGUCCGGAAACCCUGCUGCUGCUGCGACAAGUUCUCCGGG
UCCAGGCUGUGAUUUCCACAAUCUGCAUCCAAACCACAGAGUGAAGCCGAAAUUGUGUGAAUGAUCGGGA
GUGUUUGCAGAGUUCAACCACAGUGUUAUCACGUUUUAUCCAAAUCUCCUAGUCCAAAAAAGAAUACCAC
GCCCUGUUUUUAUCUCAAAAAAAAAAAAAAA

DNA

>NR_110778.1 Homo sapiens long intergenic non-protein coding RNA 1255 (LINC01255), transcript variant 1, long non-coding RNA

GCTTGCAGGTCCAAACCACTGGTAGCAGCCCCTGGTATATCCAGGACATCAGCACATAGAACCAGTGAAA
GGGTCCTCCCTCATAGTGACGCATGTCATAGACTCAGATTTATACAAACAAATGCTGAGCAAACCATGAA
GAGTCTCTGGTATTTTCAACAATATCATCACAGAGGAACTGTATTGATTTCCTTGAACTTTTCAAATGTA
GAGGAGCCTTTAGAAGGAAGCCTTGAGCATGAGGACAGACCCCCCTCATCCAACCACCACCTGACTCTGT
GTGAACCTGGAACTGTTAGAATGTCTGTGCTGTGATAAAAGGTCATCTCCCTACAATGTTATGATACAGA
CCTCAAACCTACAAACATGAGGAACTTCTGCCTCCAGGTTAACTGCTTACCCACTGTACAAGACACGTTA
CACACAGTCCAGGAGGCGGAGCTTCTGAGATATCTAGAAACAGCTTAGAGGCAAAATGACAAAATAAAGC
AGCAACAACAAATAAAACCAATCTTCTCGAACTGGC

RNA

>LINC01255

GCUUGCAGGUCCAAACCACUGGUAGCAGCCCCUGGUAUAUCCAGGACAUCAGCACAUAGAACCAGUGAAA
GGGUCCUCCCUCAUAGUGACGCAUGUCAUAGACUCAGAUUUAUACAAACAAAUGCUGAGCAAACCAUGAA
GAGUCUCUGGUAUUUUCAACAAUAUCAUCACAGAGGAACUGUAUUGAUUUCCUUGAACUUUUCAAAUGUA
GAGGAGCCUUUAGAAGGAAGCCUUGAGCAUGAGGACAGACCCCCCUCAUCCAACCACCACCUGACUCUGU
GUGAACCUGGAACUGUUAGAAUGUCUGUGCUGUGAUAAAAGGUCAUCUCCCUACAAUGUUAUGAUACAGA
CCUCAAACCUACAAACAUGAGGAACUUCUGCCUCCAGGUUAACUGCUUACCCACUGUACAAGACACGUUA
CACACAGUCCAGGAGGCGGAGCUUCUGAGAUAUCUAGAAACAGCUUAGAGGCAAAAUGACAAAAUAAAGC
AGCAACAACAAAUAAAACCAAUCUUCUCGAACUGGC

DNA
>NR_120518.1 Homo sapiens long intergenic non-protein coding RNA 1387 (LINC01387), long non-coding RNA
GTATTTCAAAGCTCTCTGGGTCCTGGGGATGAGCAGTGCCCTCTCATCCTGTAATAATCGTGGACATCAC
ACACTGATGTACAGACATGGTGCCAGCGCCTCCCTTCCTTGGGGTCTTGGAAAACCCGGTGCCACAATGG
GATCTAAGTATCCTTGGATCAATACGGATTCGTGTTTCTCACACCGAAGTTCAGGGCTCTGGCAGCTCCA
GGTCCCCTGAGGCCCTGAGGAAAGAGTCACTTGAAGTGGAGTGGACCCTGGTACTACTTGCAATTCCTCC
AAGAATTCAACCCAGCCAGCAAGACGGAGGGCCTCCCAAATGCTGCGATCTGCTCCGTGCTGCTCTGCTG
GGCCGTCACTGCCCCCTGTGCGTTCCTGCGGGCGAGGTCTTCTCCCAGAAGCGAGACAATGAACAGGACA
GAAGTGAATTCATTGGCCAGACCCTCAAACTGTTGGTGAAGAGGAACGTTTCCTTGGAATTAAGCTGCCG
CTAAGTAAAGGTCAAGGCACGGGGCAGCTGGATGTCCGTGTGGATCCTGCAAATGGAGAGGCTCTGCTGT
GGAAACAGAATGGAACTTAACCTCAGCTAAAACAGTGGTGGAG

RNA
>LINC01387
GUAUUUCAAAGCUCUCUGGGUCCUGGGGAUGAGCAGUGCCCUCUCAUCCUGUAAUAAUCGUGGACAUCAC
ACACUGAUGUACAGACAUGGUGCCAGCGCCUCCCUUCCUUGGGGUCUUGGAAAACCCGGUGCCACAAUGG
GAUCUAAGUAUCCUUGGAUCAAUACGGAUUCGUGUUUCUCACACCGAAGUUCAGGGCUCUGGCAGCUCCA
GGUCCCCUGAGGCCCUGAGGAAAGAGUCACUUGAAGUGGAGUGGACCCUGGUACUACUUGCAAUUCCUCC
AAGAAUUCAACCCAGCCAGCAAGACGGAGGGCCUCCCAAAUGCUGCGAUCUGCUCCGUGCUGCUCUGCUG
GGCCGUCACUGCCCCCUGUGCGUUCCUGCGGGCGAGGUCUUCUCCCAGAAGCGAGACAAUGAACAGGACA
GAAGUGAAUUCAUUGGCCAGACCCUCAAACUGUUGGUGAAGAGGAACGUUUCCUUGGAAUUAAGCUGCCG
CUAAGUAAAGGUCAAGGCACGGGGCAGCUGGAUGUCCGUGUGGAUCCUGCAAAUGGAGAGGCUCUGCUGU
GGAAACAGAAUGGAACUUAACCUCAGCUAAAACAGUGGUGGAG

DNA
>NR_145452.1 Homo sapiens long intergenic non-protein coding RNA 1415 (LINC01415), transcript variant 1, long non-coding RNA
GGTAAACAAAGGCAATTCGTTCTGTAAAAATACTCTAGATTCTTTCTGAGGACTGGGCACTACACTGCTC
ATAGTGGGCACCTAAAGTCAGCATTCAGCCAACTCAGCGGGCTTTAAGATGGAGACTCTTAAACCTCCTG
CATCAAAAAGACATAAGGGAATGTCCCTGAGAAGAGCCTGTGTCTCCTTTGAAGAGAGAGAGAGAGATTG
ATTCTGCCTCCTTGCAATTCGAACCGGCATTTTCCAGAACACTCCAATTTGAATCACAGTGGAGGGTCAT
GGGAGGGATTTGGCACAGGGGAGTCTTGGGTCTTTGGTTTTCCACATTGATCAATGGGATTGATTTTTTC
TGTTTTTGTTTTTGTTTTAAAAGAAGAGAATTAGTTCCTTTTTCTGTCTCCAGGATTGAAAACATGAAGG
CAAAGAAGAGAAACTCACAATATGTCATTAACTTTGGGTTATCAATATTAGTGGAAAGAATCAATGGTGC
AAAGAATCCTAAAGGACTAGATAATCCGCATATTCGACTTCCTGATTATGTGTTACTGACACTAACATTT
CAGAGTGTGGAGACCCTGCCCCATCTGGGGAGAGGGAGAGACAAAGACCCCCTGCTTCTGGTACTTGATT
CACACATAATGCCCTCCATTTACTGCTTTCAAGAAATAACCTGTTTCTAATCTTAAATTTCAATCTACTG
ATCACAAAATCATAGAGATCTTAGCCTTGAAAGAGACTTCATGAGACCATGTGACCAACTTTCTGACGCA
GGATTAGAACATCCGACATTAAAATGTCTCAGTGCAGCAGAATTTCCCAGCGACTTCGGTGGCGGGAGAG
CAGCACCTCTCAGGCACAGCTGTGCGCATCCCTCGGGCCCCACCGACCGCACCATCTGACCGCGGGAAAC
GAAATCTGAGCTCAGGAATTTGAACTGCCCCACGCACGTTCGTTCCCGGATAAATCTCCTAGAGGACCGT
GATTTTTCCACTTAGAGATTATTTAGGGAGATGTCCTGGATGGGACAAGTAGACAAATGGCGGCCCGAGG
AAAATGCAGGAAACTCTACATTGTAGACGCTTAAGCACCAACTGTCTGGGCCCCGGGGCAGGGAACGGGA
GACATTATCCACCGGAAACATCACACCCCCGGGGCTCCTGGCATTTTCTACATCAGTGAATGTGCCAGCG
AAGAAGAGTGTGGGGAAATTTTCTGAACATAGAATTCATATTCCTGAAACGTTAAGTCGTGAAGTGGGTG
CTTCAGCTGTCCCCCAGCTTTAGGATTTTAACCCAGCTTTGGTTTTGATCACCGAGAGTTATTCCTTATA
ATCAAATCAGCAAAAGCGTTCAATTACCTATTACTGGTTGAGAAAGATTCCGTTCCCCCAGCTCATTGTA
TCGGTTCTTGGACCCAAGATGTTAATTATCGGCCAGGCATTCACAGGGGGTTAATATGGCCCCCCTCGAC
ACCTTGCCTCGTACCAGAGACACCAAAAGCTGCCGCTGAGGGATAATAGAGAATGGGAAAAGCTGGTCAT
GAAAAACCTCTTTTATTCTTACTTGCAAGCTACCCTTTTGACATTCTTTTTTTAACACTAAATGGAGATT
ACTGAATTGATAACTTTTGGGTTTTCTCTGTCACACTGGGGTTTTTTTCCCCCCGTTTGTTTGTAAGGTT
TTTTTGGTTTTGGTTTTGGTTTTGGGGGGAGGGGAAGGGGGAAGTGGATAGGGAGATGGAAGAGTGTTCT
GTTACTGGTTTTATCAAATCCTGTTAGGTCTTGTTCTTCAGTGTAATGTAGAATGGAGAAGTTGCATAAG
TACATGATTGACAAACAAGGTCTCTTAGAAGAGAGTTAAGGAGTAGGAAATATAATGTGTCCTGGAGAAA
AAGAAAGAGAAAACCCTTAGTATGACACACTGGCCTTTAAATAGAGGACACATGAATATGCAAAGGAGAG
GCACGTGTGAAATAAGGTGTGCCTTGCTGAGCCCTCCCGCCCCCACCCCCCATGTATCAAGAACTTTACT
TACATTATCTCATCTAATTCTACCCACAACCCAAGAGGAAGGGCGATACTTTTAATTCGGGTTTTTACAG
ACAAGAAATTTGTATCTCAGAGGCCCAAAGTAAATTGCCTGAGGGCATATAGCTACTTAGGACAGAATTA
GAATTGATTAAAGAGTCAAAAATTCTTTGAGAATTAATGTTTTAAACTCTCGATTAGTTACCAAGGGAAT
CTGAGAAATTAATTAACTTTTGGGGGGATCTTTAGATTAGTGGGGAAATTATGTTAAGCCCAAAGAAAGA
CAAGCCCGTGAATTTTCTTGTTATCTATGAAGTGGTTTGGTGAAAGGTGTGACAATTTGAGAAAACTGAA
AAGTGCTGAAATTCCTTTAATAAGGTTCTGTTTAAACTGATAGAGCTACCAGATATAATTAGGCAGATAT
CCATTTTCTTCTGGAAGCTTCTATTATTTGAGAAGAAAACTGAAAAGCTTTAGACTGTTTCAAATTAAGG
ACAATTTGAATTTTAGTTGGAAAGCGGATAAACTATCTGTATGATTGAGTTTAAAAGGCATGGAAAGACT
ATTACGAAGTATTGATCCCACTCAGTGAGAGCAAGCACAGCTTTCAAATGATACAGATCTGGCTATGAAT
TTGAATGGTGACTGTGCCTAGCTGTGTGACCAAGGGCAACTTTCTTAACCTCTCTGGGCCTCTGAGTCCT
CGTCTTTAAAATGAGACAAATAAGCCCTTGCAAGACGCTTGTGATGATAAAATAAAAGAGTATGGGTGAA
AGTCCTTAACAGAATGCCTGATATGTAGCAGATACTCAACAGAGTTAGCAATGTTTCATTTAATTAAAGC
ATTTGAGAGCAGGATAGGGAGCACCACTAACTTCAGCTCTTCAACAATTTTGCCTGGGGCTGGCTCTGGG
GAATAGACTTGGATTCTCACGAGTATGTGAATCTTCAGTTACTTCCTTGAAAGGACAGACAGTTATAAAA
GCAAGCAGTGAAGTCTGGGAAGGTGGAGGAGAATGGTTATGGAAAGTTATCCCCACATCATAAGTCTTCC
TGATCTTGAGATCAAACAACAACACTTTCAGAAACAGCAAGAAATTAAGGCTGAAGCAAAATACCCCCTG
TATAATTTTTAAGTCAGTTAATAATGAAGGTGTAAATAAAACAGAAATGTCTCTGTTCTTACTTAAATCT
TGAACAAATAAGGGATTTTTTTCTTTAGTGTTTTTTAATAGTTTAATCATTTTTTAAGACACAATTTTTG
CCTAGTTACTGTTTACTAACTGCCCTGCCAAAAAATTAGTTATTACTTCAGGACTTATGTTTGAATAGAAC
TGCGAAATCCTGCTTTAAAAAGTACTATGAAACAATTCATCAAAATGTTTTCCTCTGATATTTTTTACCA
GGTAAAGGCAAGATTACTTCTGGAGATTTTCTTTGAAAATAATGATTTTTTTTTCCTGATTATGAAAGTA
ATACACGTTCATTATGGACAATTTAGTATAAAATGAGAGAACAAGCTATAAAGGAGCAAACGAGAACACC
CTTATTCTCACCAGTAAGAGTTAACCACTTTTAATATTTTGATGATTTCTTTCAGTCTTTTTACCCTTTA
TTTTTTTAGCCTTGGTACGAACCTATGGAGTATAAAATTGTGCATTCTTGTTTTTATATTTATTTTCTCC
CATGTAACTAAAAAGGATCTGTAACATTTTTAGTGACTGTCTAATTTTATATCATACCAATATGATATAT
TAGTATCATTTTTTAAGAAACAATAGAGAATTCTCTATTGTTGAGTGTCTGTGAGTCCCAGTTTTTGCTA
TTGTAAATTATGCTAAACAAAAATCTGTGTGCATAAATCTCTGAGTTTAAGATTATTTCGTTAAGATAGA
CTTCTATAACAAGAATTACTAGGTTCATATAATACTTCTAATGCTTTTGAAATATATTGTCGTATTGATT
TTAGCAGTTTTCAGTCCAAGCAGCAATAGATGAAGAGAGCTGTTCTCTCCTAATGCCTCTAGTCATAGTG
ACTGATACCACAGCCAAAACAAAAAAAAAAAACAACAACAAAAAAAACAAATCTTTTCTAATTTGATAGG
CTAAAGAAAAGATCTTGTTGTTTTATTTGTATTTTGGGAGGGCTACTGCCATTGAACAGCTTTTCCAGAT
GTTCATTAGCCATTTTCTCTTTTGTGAACTATCTATTACTGTCCTTTGCCTGTTTTTCTATTGAGGCCTT

AATTTTTTTAACTGTATTTATATAAACCCCACGTATCAGCCCTTTGTTGTAATTGTGGCAGTTTTTTCCC
ATTTTACTTTTTGCATTTTAATGTTATTCATGATTTTTGATACATAAAAATGTTAAAGATTTATTTAGAT
AAATGTATCAAGTATTTTTTAATTTTTTTATGTTGCTCTACAGAAAATCACCTAGATATTCACCTATTTT
TTTCTACTTGCATTTTTATGAGCTTGTTTTTACATTTAAGTCTTTATTGCTTTTGTACTTTATTGCATTG
TGAGTTAAGAATCTGATTTTTCCCTCAAATAGCTAGGCAGTTTTTCCAATACCGTCAACTCAGTGTCCAT
CTCCCCCTAGCTGATTTACTGTGCCTCCACATCAGAGACCTCTTTTGGTTGATAAAGATGCAGGGATGCT
CTTCACAGTACAGTTATTAACAGCCAGAGCAATATATGAGGATCAACTAATCGGAACCAGTCATAAGCAT
CAGATTGGGCCAGCAAGCACATCCCAGGTGAGGACCAAAATTGCAAATGGGACCTAAGAAGTTCCTGCAT
GCAACAAAAGTGGTTTCCTTTCAAAGAAGGATATGTTTAAATTAACTTAACATGTTTAAAGGGGGACTCT
CCTGTAAGTTTCATTTGGAGCACGTGGAGTTTACATTAGTTTCATTGTGTTCATTCAACAAACACTAGTT
AAGCCACCCCTATGGACAGGTTCTGTTTTAGTATCTCAATAGGTGAGAGAGAGAGAGAGAGACTCTAAACAG
ACAGACAGATAGATGATAGATAGACAGATAAATAGATAGATAATCTCCCTATTTCTCTCTTTCTGTGTGT
GTCTTTCTCATATTGTCAAAAAATGCTACTGAGCTCAGTGAAATTCACATTAAGTGGTAGTACCAATTTT
CTATTATTGATGTCACATCATTTCTCTTTCATTACAATAATTGTTACTGTTCTTTGGGATACATTCCCGG
TCAACTCATCCTTTCCTAATATGACACAAATTGCATCTGCCGGTAATTTGGTCTGCTTATTAGTTGATAG
ACATTGTAAGTCTTTGGTTTGTCCATCTGTTCTCATAGTTTGTCTGCTCCTTAAAGAATTGGATTATTTG
CTACACTTTTCAGGTGGCTTTATTTTGCATATATTTCTGAATTTTCTTTTTCATGATAACAATACCAGAA
GGATGTCCCAATTTTACATTCTATCTTTCCTTTTGCTTTGAAATAATTTAAATCACTTCAAGAAAGATTA
ATAAAGAAATTATATTTCTATCGAATCTTTATGCAATTAGGTTACTGAACTTTTGAAATCAAAGGAGGAA
ATCAGATTTTGTGGTTTTGCCCAGTATACTATAGAATGTCAATATTGTTCTATTATAAAAATAATATTCT
ATATATTATATTCTTATATTAGAACAATACTATTCTATATATAATATGCTAATATAAGAATAAGACAGCA
ATAGACCAATTGTTCCTGTTAAGGAAGACTTGTTGATTCACTGGTGCCCACCCATTGTAGATGGGGTGAG
AGGATCGGGACTCTGCTGGGATCTGACATCTGGGAATTCCCAAGGGTCCAACATTGGATGGACAAGTGGA
GCAGAAGAATCCATCATTAGGAGGAACAAAGGGTGAGGAATTCACGGGTCAATTGTGACTCCAGAGCCTG
GAAAACGAACAACAACCATGAGACTGCTACCAGAGCCAGACTCTATCCCGGGGAGAAGGAGTGTACAGAA
CCAAGCAGGTCCTTGCCACAGAGCATGGGAGAGAAATGGGGCCCCAAAGTGTCTATGAATTGACCGAAAA
TGAGGAGGAAAATGTTGACGTCATCCAATTAGCAGAGATTAACCAGATTAGGTTTTCTGGCACTTGGCAG
AATGGTAACTCAAAATAAAAACTGAGTTTAGTAAAAAACAA

RNA
>LINC01415
GGUAAACAAAGGCAAUUCGUUCUGUAAAAAUACUCUAGAUUCUUUCUGAGGACUGGGCACUACACUGCUC
AUAGUGGGCACCUAAAGUCAGCAUUCAGCCAACUCAGCGGGCUUUAAGAUGGAGACUCUUAAACCUCCUG
CAUCAAAAAGACAUAAGGGAAUGUCCCUGAGAAGAGCCUGUGUCUCCUUUGAAGAGAGAGAGAGAGAUUG
AUUCUGCCUCCUUGCAAUUCGAACCGGCAUUUUCCAGAACACUCCAAUUUGAAUCACAGUGGAGGGUCAU
GGGAGGGAUUUGGCACAGGGGAGUCUUGGGUCUUUGGUUUUCCACAUUGAUCAAUGGGAUUGAUUUUUUC
UGUUUUUGUUUUUGUUUUAAAAGAAGAGAAUUAGUUCCUUUUUCUGUCUCCAGGAUUGAAAACAUGAAGG
CAAAGAAGAGAAACUCACAAUAUGUCAUUAACUUUGGGUUAUCAAUAUUAGUGGAAAGAAUCAAUGGUGC
AAAGAAUCCUAAAGGACUAGAUAAUCCGCAUAUUCGACUUCCUGAUUAUGUGUUACUGACACUAACAUUU
CAGAGUGUGGAGACCCUGCCCCAUCUGGGGAGAGGGAGAGACAAAGACCCCCUGCUUCUGGUACUUGAUU
CACACAUAAUGCCCUCCAUUUACUGCUUUCAAGAAAUAACCUGUUUCUAAUCUUAAAUUUCAAUCUACUG
AUCACAAAAUCAUAGAGAUCUUAGCCUUGAAAGAGACUUCAUGAGACCAUGUGACCAACUUUCUGACGCA
GGAUUAGAACAUCCGACAUUAAAAUGUCUCAGUGCAGCAGAAUUUCCCAGCGACUUCGGUGGCGGGAGAG
CAGCACCUCUCAGGCACAGCUGUGCGCAUCCCUCGGGCCCCACCGACCGCACCAUCUGACCGCGGGAAAC
GAAAUCUGAGCUCAGGAAUUUGAACUGCCCCACGCACGUUCGUUCCCGGAUAAAUCUCCUAGAGGACCGU
GAUUUUUCCACUUAGAGAUUAUUUAGGGAGAUGUCCUGGAUGGGACAAGUAGACAAAUGGCGGCCCGAGG
AAAAUGCAGGAAACUCUACAUUGUAGACGCUUAAGCACCAACUGUCUGGGCCCCGGGGCAGGGAACGGGA
GACAUUAUCCACCGGAAACAUCACACCCCCGGGGCUCCUGGCAUUUUCUACAUCAGUGAAUGUGCCAGCG
AAGAAGAGUGUGGGGAAAUUUUCUGAACAUAGAAUUCAUAUUCCUGAAACGUUAAGUCGUGAAGUGGGUG
CUUCAGCUGUCCCCCAGCUUUAGGAUUUUAACCCAGCUUUGGUUUUGAUCACCGAGAGUUAUUCCUUAUA
AUCAAAUCAGCAAAAGCGUUCAAUUACCUAUUACUGGUUGAGAAAGAUUCCGUUCCCCCAGCUCAUUGUA
UCGGUUCUUGGACCCAAGAUGUUAAUUAUCGGCCAGGCAUUCACAGGGGGUUAAUAUGGCCCCCCUCGAC
ACCUUGCCUCGUACCAGAGACACCAAAAGCUGCCGCUGAGGGAUAAUAGAGAAUGGGAAAAGCUGGUCAU
GAAAAACCUCUUUUAUUCUUACUUGCAAGCUACCCUUUUGACAUUCUUUUUUUAACACUAAAUGGAGAUU
ACUGAAUUGAUAACUUUUGGGUUUUCUCUGUCACACUGGGGUUUUUUUCCCCCCGUUUGUUUGUAAGGUU
UUUUUGGUUUUGGUUUUGGUUUUGGGGGGAGGGGAAGGGGAAGUGGAUAGGGAGAUGGAAGAGUGUUCU
GUUACUGGUUUUAUCAAAUCCUGUUAGGUCUUGUUCUUCAGUGUAAUGUAGAAUGGAGAAGUUGCAUAAG
UACAUGAUUGACAAACAAGGUCUCUUAGAAGAGAGUUAAGGAGUAGGAAAUAUAAUGUGUCCUGGAGAAA
AAGAAAGAGAAAACCCUUAGUAUGACACACUGGCCUUUAAAUAGAGGACACAUGAAUAUGCAAAGGAGAG
GCACGUGUGAAAUAAGGUGUGCCUUGCUGAGCCCUCCCGCCCCCACCCCCCAUGUAUCAAGAACUUUACU
UACAUUAUCUCAUCUAAUUCUACCCACAACCCAAGAGGAAGGGCGAUACUUUUAAUUCGGGUUUUUACAG
ACAAGAAAUUUGUAUCUCAGAGGCCCAAAGUAAAUUGCCUGAGGGCAUAUAGCUACUUAGGACAGAAUUA
GAAUUGAUUAAAGAGUCAAAAAUUCUUUGAGAAUUAAUGUUUUAAACUCUCGAUUAGUUACCAAGGGAAU
CUGAGAAAUUAAUUAACUUUUGGGGGGAUCUUUAGAUUAGUGGGGAAAUUAUGUUAAGCCCAAAGAAAGA

CAAGCCCGUGAAUUUUCUUGUUAUCUAUGAAGUGGUUUGGUGAAAGGUGUGACAAUUUGAGAAAACUGAA
AAGUGCUGAAAUUCCUUUAAUAAGGUUCUGUUUAAACUGAUAGAGCUACCAGAUAUAAUUAGGCAGAUAU
CCAUUUUCUUCUGGAAGCUUCUAUUAUUUGAGAAGAAAACUGAAAAGCUUUAGACUGUUUCAAAUUAAGG
ACAAUUUGAAUUUUAGUUGGAAAGCGGAUAAACUAUCUGUAUGAUUGAGUUUAAAAGGCAUGGAAAGACU
AUUACGAAGUAUUGAUCCCACUCAGUGAGAGCAAGCACAGCUUUCAAAUGAUACAGAUCUGGCUAUGAAU
UUGAAUGGUGACUGUGCCUAGCUGUGUGACCAAGGGCAACUUUCUUAACCUCUCUGGGCCUCUGAGUCCU
CGUCUUUAAAAUGAGACAAAUAAGCCCUUGCAAGACGCUUGUGAUGAUAAAAUAAAAGAGUAUGGGUGAA
AGUCCUUAACAGAAUGCCUGAUAUGUAGCAGAUACUCAACAGAGUUAGCAAUGUUUCAUUUAAUUAAAGC
AUUUGAGAGCAGGAUAGGGAGCACCACUAACUUCAGCUCUUCAACAAUUUUGCCUGGGGCUGGCUCUGGG
GAAUAGACUUGGAUUCUCACGAGUAUGUGAAUCUUCAGUUACUUCCUUGAAAGGACAGACAGUUAUAAAA
GCAAGCAGUGAAGUCUGGGAAGGUGGAGGAGAAUGGUUAUGGAAAGUUAUCCCCACAUCAUAAGUCUUCC
UGAUCUUGAGAUCAAACAACAACACUUUCAGAAACAGCAAGAAAUUAAGGCUGAAGCAAAAUACCCCCUG
UAUAAUUUUUAAGUCAGUUAAUAAUGAAGGUGUAAAUAAAACAGAAAUGUCUCUGUUCUUACUUAAAUCU
UGAACAAAUAAGGGAUUUUUUUCUUUAGUGUUUUUUAAUAGUUUAAUCAUUUUUUAAGACACAAUUUUUG
CCUAGUUACUGUUUACUAACUGCCCUGCCAAAAAUUAGUUAUUACUUCAGGACUUAUGUUUGAAUAGAAC
UGCGAAAUCCUGCUUUAAAAAGUACUAUGAAACAAUUCAUCAAAAUGUUUUCCUCUGAUAUUUUUUACCA
GGUAAAGGCAAGAUUACUUCUGGAGAUUUUCUUUGAAAAUAAUGAUUUUUUUUUCCUGAUUAUGAAAGUA
AUACACGUUCAUUAUGGACAAUUUAGUAUAAAAUGAGAGAACAAGCUAUAAAGGAGCAAACGAGAACACC
CUUAUUCUCACCAGUAAGAGUUAACCACUUUUAAUAUUUUGAUGAUUUCUUUCAGUCUUUUUACCCUUUA
UUUUUUUAGCCUUGGUACGAACCUAUGGAGUAUAAAAUUGUGCAUUCUUGUUUUUAUAUUUAUUUUCUCC
CAUGUAACUAAAAAGGAUCUGUAACAUUUUUAGUGACUGUCUAAUUUUAUAUCAUACCAAUAUGAUAUAU
UAGUAUCAUUUUUUAAGAAACAAUAGAGAAUUCUCUAUUGUUGAGUGUCUGUGAGUCCCAGUUUUUGCUA
UUGUAAAUUAUGCUAAACAAAAAUCUGUGUGCAUAAAUCUCUGAGUUUAAGAUUAUUUCGUUAAGAUAGA
CUUCUAUAACAAGAAUUACUAGGUUCAUAUAAUACUUCUAAUGCUUUUGAAAUAUAUUGUCGUAUUGAUU
UUAGCAGUUUUCAGUCCAAGCAGCAAUAGAUGAAGAGAGCUGUUCUCUCCUAAUGCCUCUAGUCAUAGUG
ACUGAUACCACAGCCAAAACAAAAAAAAAAAAACAACAACAAAAAAAACAAAUCUUUUCUAAUUUGAUAGG
CUAAAGAAAAGAUCUUGUUGUUUUAUUUGUAUUUUGGGAGGGCUACUGCCAUUGAACAGCUUUUCCAGAU
GUUCAUUAGCCAUUUUCUCUUUUGUGAACUAUCUAUUACUGUCCUUUGCCUGUUUUUCUAUUGAGGCCUU
AAUUUUUUUAACUGUAUUUAUAUAAACCCCACGUAUCAGCCCUUUGUUGUAAUUGUGGCAGUUUUUUCCC
AUUUUACUUUUUGCAUUUUAAUGUUAUUCAUGAUUUUUGAUACAUAAAAAUGUUAAAGAUUUAUUUAGAU
AAAUGUAUCAAGUAUUUUUUAAUUUUUUUAUGUUGCUCUACAGAAAAUCACCUAGAUAUUCACCUAUUUU
UUUCUACUUGCAUUUUUAUGAGCUUGUUUUUACAUUUAAGUCUUUAUUGCUUUUGUACUUUAUUGCAUUG
UGAGUUAAGAAUCUGAUUUUUCCCUCAAAUAGCUAGGCAGUUUUUCCAAUACCGUCAACUCAGUGUCCAU
CUCCCCCUAGCUGAUUUACUGUGCCUCCACAUCAGAGACCUCUUUUGGUUGAUAAAGAUGCAGGGAUGCU
CUUCACAGUACAGUUAUUAACAGCCAGAGCAAUAUAUGAGGAUCAACUAAUCGGAACCAGUCAUAAGCAU
CAGAUUGGGCCAGCAAGCACAUCCCAGGUGAGGACCAAAAUUGCAAAUGGGACCUAAGAAGUUCCUGCAU
GCAACAAAAGUGGUUUCCUUUCAAAGAAGGAUAUGUUUAAAUUAACUUAACAUGUUUAAAGGGGGACUCU
CCUGUAAGUUUCAUUUGGAGCACGUGGAGUUUACAUUAGUUUCAUUGUGUUCAUUCAACAAACACUAGUU
AAGCCACCCCUAUGGACAGGUUCUGUUUUAGUAUCUCAAUAGGUGAGAGAGAGAGAGAGACUCUAAACAG
ACAGACAGAUAGAUGAUAGAUAGACAGAUAAAUAGAUAGAUAAUCUCCCUAUUUCUCUCUUUCUGUGUGU
GUCUUUCUCAUAUUGUCAAAAAAUGCUACUGAGCUCAGUGAAAUUCACAUUAAGUGGUAGUACCAAUUUU
CUAUUAUUGAUGUCACAUCAUUUCUCUUUCAUUACAAUAAUUGUUACUGUUCUUUGGGAUACAUUCCCGG
UCAACUCAUCCUUUCCUAAUAUGACACAAAUUGCAUCUGCCGGUAAUUUGGUCUGCUUAUUAGUUGAUAG
ACAUUGUAAGUCUUUGGUUUGUCCAUCUGUUCUCAUAGUUUGUCUGCUCCUUAAAGAAUUGGAUUAUUUG
CUACACUUUUCAGGUGGCUUUAUUUUGCAUAUAUUUCUGAAUUUUCUUUUUCAUGAUAACAAUACCAGAA
GGAUGUCCCAAUUUUACAUUCUAUCUUUCCUUUUGCUUUGAAAUAAUUUAAAUCACUUCAAGAAAGAUUA
AUAAAGAAAUUAUAUUUCUAUCGAAUCUUUAUGCAAUUAGGUUACUGAACUUUUGAAAUCAAAGGAGGAA
AUCAGAUUUUGUGGUUUUGCCCAGUAUACUAUAGAAUGUCAAUAUUGUUCUAUUAUAAAAAUAAUAUUCU
AUAUAUUAUAUUCUUAUAUUAGAACAAUACUAUUCUAUAUAUAAUAUGCUAAUAUAAGAAUAAGACAGCA
AUAGACCAAUUGUUCCUGUUAAGGAAGACUUGUUGAUUCACUGGUGCCCACCCAUUGUAGAUGGGGUGAG
AGGAUCGGGACUCUGCUGGGAUCUGACAUCUGGGAAUUCCCAAGGGUCCAACAUUGGAUGGACAAGUGGA
GCAGAAGAAUCCAUCAUUAGGAGGAACAAAGGGUGAGGAAUUCACGGGUCAAUUGUGACUCCAGAGCCUG
GAAAACGAACAACAACCAUGAGACUGCUACCAGAGCCAGACUCUAUCCCGGGGAGAAGGAGUGUACAGAA
CCAAGCAGGUCCUUGCCACAGAGCAUGGGAGAGAAAUGGGGCCCCAAAGUGUCUAUGAAUUGACCGAAAA
UGAGGAGGAAAAUGUUGACGUCAUCCAAUUAGCAGAGAUUAACCAGAUUAGGUUUUCUGGCACUUGGCAG
AAUGGUAACUCAAAAUAAAAACUGAGUUUAGUAAAAAACAA

DNA

>NR_110755.1 Homo sapiens long intergenic non-protein coding RNA 1416 (LINC01416), long non-coding RNA

AGTGTACAGAGCTGCTGTCTTTTGTAGATGGGAAAAAGGGTATTTGTTATGTCTCAGGAAAGCTGGATGA
CTATCTCATTCTCTGATGATGGAGAAGAATACTCACAACCTGCTCCCCACCTCCCACTGTGAAATGCCAA
TATGGTGGAAACAGAAGGGAGAAGAAAGAACAGCGGAGACACTGATCATTCGAAACCAAAGAAGCAGCTC
ATGCATAATGGAGAGCTATCTGCATGCAAATTGCTAGGAGTACTTCCTACTTTTCTGGGATATTGTATTT
AGCCCTCTGTACTCTATTCAGAAGAGTTCTGTTCCCCAGGCCAAGAAGGGGAAATACATTGAAGACAACA
TTAAGTGTTGGAACTAAATGCACAAGAATCAGGAGCATCTTAACGCCATGGCCTGAACATGATAGGTACT
GAAGAACTATGGATTAATTATTTTTGATTGATTGATTGATTGAAAATGTGTCACTATAAAAAAGCAAATG
TGAAGCTTACAGAGGGATAGTGGTGCAGTTAAATGCTAAAGTTAAATCTCCTTTTCTGGCAGCTGCAGAA
AGATTCCTATTCTGCCTCCTATACTTGCTCTGAGGATATTTAAATTGTAGAGCTCTAAGTGGCTGCCCTC
AATTTGCTTCAAAGATAATAACGTCACCTTACAGTTTATAAAGCACCTTGTTACATCCCTTTGGAGACCT
ACAGCAATCCCGTATGAATCTGGGGATTATTTATTCTTTTTTTCAAATAAGAAAACTGAAGCACAGAGGA
AGGAGTTACACATTAAAAGCCAGGGAACTTGTACATTAGTTAAGGTAATGCTAGCAGCTATAACAAATAA
ATGCCCAAATCTCAGAACTTAATACAATAAATCTTACTTTTTGATTATCCTATGGCACCATACGGTGTTC
TGGGTAGATGAAGAGTCTTCCACAAGGTATTTCAGGGATCTGTGGCTCTACCTTCCTCTAGTCTTCAAAT
CTTCTGCATCCAACAAGCAGATGGAGAATGAGGAATTGGAATAGGCATTCCTTAGTTTAAGCTCTTTGGT
CCAGAACTGAAACACATAACATCTGCTGATATCCTATGACTAGAACATCACCTGGCCCTCATCTGGATAC
AAGACGATGGTACTGAGAAATAATTGCAGCCTGGGCAGATGCTTTTCAGTGCAATTCTATTCTAAAGAAA
AGGCAGACTGAATGCTACATGCCAGTAAGTAGAATCTCCAGGACTCAAAAACAGACACATGACATTAAAT
GCAGTGCTCTTGCTACTACAACACACTGCCTCTGTATTCAGAGTTGCCTCAGCCTAGATCATAGTTTTGG
AGTGAGTTAGAACCTGATCCTAACACTTGACAGTATTAAAAGGGGGACTGTCACCAGTGGGACGAACCCT
TGAACTAAGCATGAGGAGAACCTGGCTTATGCCCTGGCTCTGCCCTATCTGACTGATTGAGTAAATCATG
AAATCACTCTGCATTTCTTTTTTTCAAAGTCATTGATACAAACCTATACTTCTCAACTTTTCCCAATTAA
GCTACTAGAATCTTACATTATTTTTCCCTAAGATGCTCAGACATTCCTATAATGTTATACGAGTCCGTGT
TATAAGAGCCCCTCAACAATAAGCGGTAGCTGATGAAACCATATCAATACATAATTCTATACAAAGGTAA
GAAACTGGAAACATTATTTGGCCTTCATAGACCTTGCTTGGATAACTGCTCAATTTCTATGAAGGTATCG
GAATTATTGGGAGGTGAAATTGGCCACTCCCGTAACTATTTCTGCCGATCTCCAATTGGGAACCATAGAG
CTTCATGACCTAAGGCTCCTACCAGTTACCATCTGAAAATGTCTTTTGTCTCTGTAATCTTGACAAGTGA
ATTCCAGTCACACCCATCCTAAGAAGAGTGTGCCTGGAAATACTGGTTTTCTTCTTCTCAAGCTGTCTGA
ATCCAGAAGTATATATTAATCTGGAAGTGGCTGGAGGTGATGGAAAAGAGAGGATGTTGAAAAAAGCACC
CTTGGTGAAGTGGAGCAGCAACTCGCTCAGAAGCCTCATGAGTCTGTAACATCGTTTGAACTCCAAAGCT
TTCTTCATCACTCATAAATCCATAATCATCACTCCACCTCGGAAAAATCTCACTCTGCTCTTCTCTATCT
TCTACATGAAATGTATCAGGAAGAAAAATTAACATAAGGAACTTTCACCAGCTTTATAAAATCTTAACCC
TTTTGTACACTTGTTTCTGATAATTTTAAGAACTAATATCAATAATCATTAAAACGTTGAACCGTGCCCA
AAAAAAAAAAAAAAA

RNA

>LINC01416

AGUGUACAGAGCUGCUGUCUUUUGUAGAUGGGAAAAAGGGUAUUUGUUAUGUCUCAGGAAAGCUGGAUGA
CUAUCUCAUUCUCUGAUGAUGGAGAAGAAUACUCACAACCUGCUCCCCACCUCCCACUGUGAAAUGCCAA
UAUGGUGGAAACAGAAGGGAGAAGAAAGAACAGCGGAGACACUGAUCAUUCGAAACCAAAGAAGCAGCUC
AUGCAUAAUGGAGAGCUAUCUGCAUGCAAAUUGCUAGGAGUACUUCCUACUUUUCUGGGAUAUUGUAUUU
AGCCCUCUGUACUCUAUUCAGAAGAGUUCUGUUCCCCAGGCCAAGAAGGGGAAAUACAUUGAAGACAACA
UUAAGUGUUGGAACUAAAUGCACAAGAAUCAGGAGCAUCUUAACGCCAUGGCCUGAACAUGAUAGGUACU
GAAGAACUAUGGAUUAAUUAUUUUUGAUUGAUUGAUUGAUUGAAAAUGUGUCACUAUAAAAAAGCAAAUG
UGAAGCUUACAGAGGGAUAGUGGUGCAGUUAAAUGCUAAAGUUAAAUCUCCUUUUCUGGCAGCUGCAGAA
AGAUUCCUAUUCUGCCUCCUAUACUUGCUCUGAGGAUAUUUAAAUUGUAGAGCUCUAAGUGGCUGCCCUC
AAUUUGCUUCAAAGAUAAUAACGUCACCUUACAGUUUAUAAAGCACCUUGUUACAUCCCUUUGGAGACCU
ACAGCAAUCCCGUAUGAAUCUGGGGAUUAUUUAUUCUUUUUUUCAAAUAAGAAAACUGAAGCACAGAGGA
AGGAGUUACACAUUAAAAGCCAGGGAACUUGUACAUUAGUUAAGGUAAUGCUAGCAGCUAUAACAAAUAA
AUGCCCAAAUCUCAGAACUUAAUACAAUAAAUCUUACUUUUUGAUUAUCCUAUGGCACCAUACGGUGUUC
UGGGUAGAUGAAGAGUCUUCCACAAGGUAUUUCAGGGAUCUGUGGCUCUACCUUCCUCUAGUCUUCAAAU
CUUCUGCAUCCAACAAGCAGAUGGAGAAUGAGGAAUUGGAAUAGGCAUUCCUUAGUUUAAGCUCUUUGGU
CCAGAACUGAAACACAUAACAUCUGCUGAUAUCCUAUGACUAGAACAUCACCUGGCCCUCAUCUGGAUAC
AAGACGAUGGUACUGAGAAAUAAUUGCAGCCUGGGCAGAUGCUUUUCAGUGCAAUUCUAUUCUAAAGAAA
AGGCAGACUGAAUGCUACAUGCCAGUAAGUAGAAUCUCCAGGACUCAAAAACAGACACAUGACAUUAAAU
GCAGUGCUCUUGCUACUACAACACACUGCCUCUGUAUUCAGAGUUGCCUCAGCCUAGAUCAUAGUUUUGG
AGUGAGUUAGAACCUGAUCCUAACACUUGACAGUAUUAAAAGGGGGACUGUCACCAGUGGGACGAACCCU
UGAACUAAGCAUGAGGAGAACCUGGCUUAUGCCCUGGCUCUGCCCUAUCUGACUGAUUGAGUAAAUCAUG
AAAUCACUCUGCAUUUCUUUUUUUCAAAGUCAUUGAUACAAACCUAUACUUCUCAACUUUUCCCAAUUAA
GCUACUAGAAUCUUACAUUAUUUUUCCCUAAGAUGCUCAGACAUUCCUAUAAUGUUAUACGAGUCCGUGU
UAUAAGAGCCCCUCAACAAUAAGCGGUAGCUGAUGAAACCAUAUCAAUACAUAAUUCUAUACAAAGGUAA

GAAACUGGAAACAUUAUUUGGCCUUCAUAGACCUUGCUUGGAUAACUGCUCAAUUUCUAUGAAGGUAUCG
GAAUUAUUGGGAGGUGAAAUUGGCCACUCCCGUAACUAUUUCUGCCGAUCUCCAAUUGGGAACCAUAGAG
CUUCAUGACCUAAGGCUCCUACCAGUUACCAUCUGAAAAUGUCUUUUGUCUCUGUAAUCUUGACAAGUGA
AUUCCAGUCACACCCAUCCUAAGAAGAGUGUGCCUGGAAAUACUGGUUUUCUUCUUCUCAAGCUGUCUGA
AUCCAGAAGUAUAUAUUAAUCUGGAAGUGGCUGGAGGUGAUGGAAAAGAGAGGAUGUUGAAAAAAGCACC
CUUGGUGAAGUGGAGCAGCAACUCGCUCAGAAGCCUCAUGAGUCUGUAACAUCGUUUGAACUCCAAAGCU
UUCUUCAUCACUCAUAAAUCCAUAAUCAUCACUCCACCUCGGAAAAAUCUCACUCUGCUCUUCUCUAUCU
UCUACAUGAAAUGUAUCAGGAAGAAAAAUUAACAUAAGGAACUUUCACCAGCUUUAUAAAAUCUUAACCC
UUUUGUACACUUGUUUCUGAUAAUUUUAAGAACUAAUAUCAAUAAUCAUUAAAACGUUGAACCGUGCCCA
AAAAAAAAAAAAAAA

DNA

>NR_104164.1 Homo sapiens long intergenic non-protein coding RNA 1443 (LINC01443), long non-coding RNA

ACTTCAGGCCACTCCTGCACCCCGGGACTTTCACTCTGAGAAATCCTTTACCGTGGAAGCAGGTTATGCT
GTACAATTGGAGGCATTGTACTGATCTCTTCCTACCACACTGAATATCGCATGATATCCTGAAAGTGATC
TAGATGAAGTTGTGCCAACAACATCATGACCTGCTGGATTCCACACTTCCCAGTGCAGCAGAGCCCTGAC
CTCACTCTCACATGCCTTATGTCCCTGGAATCACGTAACAGCCACCGCCAGGCAGTCATCGCAGAGAAAA
CAAGGAAAACACCACGTGGATTCCCTCGGGATGAGATCAGGTGCACGCTGCCAGCTCAATGGGTCCACCA
CCCACCAGAGACTCCAGCCCAGTGTCGCAGCCGGCGGGGCGGCACCCACTGCTCTCCCACTCCAGACCTG
ATTTCACATTCACATGGAGCCACGGTCAGGTGGTCTTCGGTCCCATAAAGCCTATGCATGTATTTTCCTC
AGAGAGCCCACGGAGGAGAGAGAGAGATGGCTAAAACAAGAAGAGATCGGTGGATGACTACATCTGCCGGCC
AGAAGACCACTCTGATAGCTTTCATGAGGATGACTGCGTCTCCCAGCCAAAAGGCCACTCTGATAGCTTC
CATGAGGATGACTGCATCTCCTGGCCAAAAGACCACTCTGATAGCTTCCATGAGCTCTCCCTGGGGCATC
CATGGAGAAGATATTTTTGAGGGAGAAATCCCCAATGCTTCTTGAATCTTGCAGCCCACACAGGGATTTC
CTACAAGCAACCCAGCCTTGAGCTATAAAGACCTGATCACTTTCCTGGGTGAAGACAGCAGACTGACTCA
GTTATTCTGTGGATAGGTGACTTGATCAATGAGTTGGCGAGAGTTCTAAGATGTGTCTTTCAGGCACATA
TCTCAGATTTGTAAAATTATTATTTATTTATTTAATTCATTTTTTTTTTTGAGATGGAGACTCACTCTGT
CACCCAGGCTGGAGTGCAGTGGCACAATCTCGGCTCACTGCAACCTCCACCTCCCAGGTTCAAATGATTC
TCCTGCCTCAGCCTCCTGAGTAGCCGGGATTACAGGCACCTGCCACCATGCCCAGCTAATCTTTGTATTT
TTAGTATAGACAGGGTTTCACCATATTGGCTGCACTGGTCTCCAACTCCTGACCTCAGGTGATCCACCTG
CCTCAGCCTCCCAAATTGCTGGGATTACAGGCATGAACCACTGTGCCTGGCCTCAGATTTGTAAGATAAT
TTAAACAAGACTCAGTGTCTCTGCATCTCACACTGGTTGTATATTGCATTAAAATGGTGATAATTCTCCC
CTAATCAAACTGTGCCCAATGCTGGCAAGGACACTAATGTTATGAAGACAAGAGGTAGCTGAAAAATAAA
GAGACAATAGCCACGAGACAGACCCAGAGGTCAGGCAGGGCAGGGTTGCCGTGAGGACATGGCTCGTCCC
ACAGGACCTGGGAACTGGTGGTCACAGCAGTGCAAGGTCCTGTTCTCTCCTCTGCAGGGACAGACAGGCC
ACCAGCCTGACAGAGACGGCATTAGTGGGCAGCTGCCAGGAACTAGCAGGGATTGCACTAGACTTTATAG
CGCCATAGTTCAGAATTGCTGGATTTGGAGACAAAATCCAGGTTTGAATTGTGATTCTATTTCTTACTGC
TCCGTGTCCTGGGGCAGCCAGGTCAGCTCTCTGAGCCCTATGGTCTCCATGGCTGAGTGAGAATGCCCGC
CTCCACTCAGAACCAGCCAGTGTGGTGCCAGCAACCTATCTAACACAAGCAAAGAGGATTTCTTAATGAA
AACATTTTGTCTTGCACAAAACAATACTCAATTTAAGAAACAGCAAATGCA

RNA

>LINC01443

ACUUCAGGCCACUCCUGCACCCCGGGACUUUCACUCUGAGAAAUCCUUUACCGUGGAAGCAGGUUAUGCU
GUACAAUUGGAGGCAUUGUACUGAUCUCUUCCUACCACACUGAAUAUCGCAUGAUAUCCUGAAAGUGAUC
UAGAUGAAGUUGUGCCAACAACAUCAUGACCUGCUGGAUUCCACACUUCCCAGUGCAGCAGAGCCCUGAC
CUCACUCUCACAUGCCUUAUGUCCCUGGAAUCACGUAACAGCCACCGCCAGGCAGUCAUCGCAGAGAAAA
CAAGGAAAACACCACGUGGAUUCCCUCGGGAUGAGAUCAGGUGCACGCUGCCAGCUCAAUGGGUCCACCA
CCCACCAGAGACUCCAGCCCAGUGUCGCAGCCGGCGGGGCGGCACCCACUGCUCUCCCACUCCAGACCUG
AUUUCACAUUCACAUGGAGCCACGGUCAGGUGGUCUUCGGUCCCAUAAAGCCUAUGCAUGUAUUUUCCUC
AGAGAGCCCACGGAGGAGAGAGAGAGAUGGCUAAAACAAGAAGAGAUCGGUGGAUGACUACAUCUGCCGGCC
AGAAGACCACUCUGAUAGCUUUCAUGAGGAUGACUGCGUCUCCCAGCCAAAAGGCCACUCUGAUAGCUUC
CAUGAGGAUGACUGCAUCUCCUGGCCAAAAGACCACUCUGAUAGCUUCCAUGAGCUCUCCCUGGGGCAUC
CAUGGAGAAGAUAUUUUUGAGGGAGAAAUCCCCAAUGCUUCUUGAAUCUUGCAGCCCACACAGGGAUUUC
CUACAAGCAACCCAGCCUUGAGCUAUAAAGACCUGAUCACUUUCCUGGGUGAAGACAGCAGACUGACUCA
GUUAUUCUGUGGAUAGGUGACUUGAUCAAUGAGUUGGCGAGAGUUCUAAGAUGUGUCUUUCAGGCACAUA
UCUCAGAUUUGUAAAAUUAUUAUUUAUUUAUUUAAUUCAUUUUUUUUUUUGAGAUGGAGACUCACUCUGU
CACCCAGGCUGGAGUGCAGUGGCACAAUCUCGGCUCACUGCAACCUCCACCUCCCAGGUUCAAAUGAUUC
UCCUGCCUCAGCCUCCUGAGUAGCCGGGAUUACAGGCACCUGCCACCAUGCCCAGCUAAUCUUUGUAUUU
UUAGUAUAGACAGGGUUUCACCAUAUUGGCUGCACUGGUCUCCAACUCCUGACCUCAGGUGAUCCACCUG
CCUCAGCCUCCCAAAUUGCUGGGAUUACAGGCAUGAACCACUGUGCCUGGCCUCAGAUUUGUAAGAUAAU
UUAAACAAGACUCAGUGUCUCUGCAUCUCACACUGGUUGUAUAUUGCAUUAAAAUGGUGAUAAUUCUCCC
CUAAUCAAACUGUGCCCAAUGCUGGCAAGGACACUAAUGUUAUGAAGACAAGAGGUAGCUGAAAAAUAAA
GAGACAAUAGCCACGAGACAGACCCAGAGGUCAGGCAGGGCAGGGUUGCCGUGAGGACAUGGCUCGUCCC
ACAGGACCUGGGAACUGGUGGUCACAGCAGUGCAAGGUCCUGUUCUCUCCUCUGCAGGGACAGACAGGCC
ACCAGCCUGACAGAGACGGCAUUAGUGGGCAGCUGCCAGGAACUAGCAGGGAUUGCACUAGACUUUAUAG
CGCCAUAGUUCAGAAUUGCUGGAUUUGGAGACAAAAUCCAGGUUUGAAUUGUGAUUCUAUUUCUUACUGC
UCCGUGUCCUGGGGCAGCCAGGUCAGCUCUCUGAGCCCUAUGGUCUCCAUGGCUGAGUGAGAAUGCCCGC
CUCCACUCAGAACCAGCCAGUGUGGUGCCAGCAACCUAUCUAACACAAGCAAAGAGGAUUUCUUAAUGAA
AACAUUUUGUCUUGCACAAAACAAUACUCAAUUUAAGAAACAGCAAAUGCA

>NR_110783.1 Homo sapiens long intergenic non-protein coding RNA 1444 (LINC01444), long non-coding RNA
GCCATAGTAGCTCTGGGAACTGAATCTCGGGGACCAGGAACAGTCTCGCATGTCATTGTAATCTGGATTC
ATGAACTCTGACTTTCCACAGTCTTTGAGTCACTGAGCACCAGGATCAACTTTCACAGAGGATCCAGATA
ATCAGCCAGCTTGAGTCACACTCCTGCAGCTTGGCTAGGGTAGGGATTCCTGACCAACAGTCCCATAAGA
TCTTATCCAGTCAGGAGGTACTGGACCAATCTGAAGTGCAGCCAAGACCGGGAGCCACAGACCTGGAACC
TGGGTACTCAAAGAGTTGTCCACGTGCCAGCAGCACTGGCCCCTCCAGGAGCTTGTTAGAAATGTAGAAC
CTCAGAGCCTACATTAAAGATATTGAATCGGAGTCTGCACGTCACATCTGAGAAGCCATGAGCTAAGCTG
ATGCCATTCAGCTGGGTAGCATCGCAGTCACTGTGCAACTTTTAAACCTACAGCTGCCCAGGCCCACTGC
AGACCTGCTAAATTCGAGCTCCTTGTAGTGGGGCCAGGGCCCTGGGACTTCACACAAAACTCTGCAAGTC
ATCACTGATGAGAAAACCAAGACTCGATGAGAGTCTCCTGAGCTCGGGGGAAGAAGCTGCATAACCGGCA
CACTTCCAGAAAGGACCGAGGAAGAGAGCGTCGCTTCAGCTAGGGATCTGATTAGTCATGCCTGGTAGGC
TCTGCTGAGCTGGGGGCTCTTGGGTTTTGCTTGTTGAGCTGAATTAATATATTAATGGATTTC

RNA
>LINC01444
GCCAUAGUAGCUCUGGGAACUGAAUCUCGGGGACCAGGAACAGUCUCGCAUGUCAUUGUAAUCUGGAUUC
AUGAACUCUGACUUUCCACAGUCUUUGAGUCACUGAGCACCAGGAUCAACUUUCACAGAGGAUCCAGAUA
AUCAGCCAGCUUGAGUCACACUCCUGCAGCUUGGCUAGGGUAGGGAUUCCUGACCAACAGUCCCAUAAGA
UCUUAUCCAGUCAGGAGGUACUGGACCAAUCUGAAGUGCAGCCAAGACCGGGAGCCACAGACCUGGAACC
UGGGUACUCAAAGAGUUGUCCACGUGCCAGCAGCACUGGCCCCUCCAGGAGCUUGUUAGAAAUGUAGAAC
CUCAGAGCCUACAUUAAAGAUAUUGAAUCGGAGUCUGCACGUCACAUCUGAGAAGCCAUGAGCUAAGCUG
AUGCCAUUCAGCUGGGUAGCAUCGCAGUCACUGUGCAACUUUUAAACCUACAGCUGCCCAGGCCCACUGC
AGACCUGCUAAAUUCGAGCUCCUUGUAGUGGGGCCAGGGCCCUGGGACUUCACACAAAACUCUGCAAGUC
AUCACUGAUGAGAAAACCAAGACUCGAUGAGAGUCUCCUGAGCUCGGGGGAAGAAGCUGCAUAACCGGCA
CACUUCCAGAAAGGACCGAGGAAGAGAGCGUCGCUUCAGCUAGGGAUCUGAUUAGUCAUGCCUGGUAGGC
UCUGCUGAGCUGGGGGCUCUUGGGUUUUGCUUGUUGAGCUGAAUUAAUAUAUUAAUGGAUUUC

>NR_110791.1 Homo sapiens long intergenic non-protein coding RNA 1477 (LINC01477), long non-coding RNA
GTCTCTGAGTCTCTGTCTCTCTCTGTCTCCTCACAGTCTGTTACTACTTAATACAGTGCTTATTGGCCTG
AGATTGGACATTATTTAAAAGCAATTTGGAGAGTTTTCTCCATTTCAGGCAAGAAAACATGCACTATTTT
TCTGATATGGCATGGGACATCGAAAAATAGTTTTGAACAAAATTTTGAAATTTTTGGCCCAAAGAAAGCA
AATACCCCCTGCAACAAACAAGATGTGTCACAATAAGTCATGATTTACAATCCCTTTCAAGACTGCCTGT
TGCCCTTAGCAATCCCTAAAGCAAAAACAAACAAAACAAATCTTTTCAGCATATCTTCATGAAACTGTCT
GGGATGTGACAAAACTTTGAGAAACAGTTTTGTGATAATGGTTTTGCACTTTATTGGTACTTCTCCACAG
GAGGTGGTGAATGGCTACTTTCAAATACATAGAGCTATCAAAGGCTAAAGTCAATAAAAAACCTCCATAG
AATAATTGGAGAGGAAAGAAAAGGGGGAAAAAAAGAACATAAAACGAATGAAAAGGCAAGTAGGACCTAT
AAATATAAAAGCCTAAGGAGTATATTTTGGGAGAACAGATGATGCAAAGCAAAATTGGGAATAGTTATTG
GAGGATCAAGGAAGGCAAATAAAACAATAAGATATATGATGCCTGTCCTCCTGCCTCAAGTCTAGCTCAT
CAGACAGTTTTTCATTCCATTTTGTATATATGAGTTTCATCTGACTACTGCCAACAGAAAGTGCTTGGAT
TTCCTGACTTTTTTCACTGTCAAAAGAAATAATAAGCTGATGAGATTCAAAGTACTTCTCCTTCCTGCCA
CCGCGTGAAGAAGGATGTGTTTGCTTCCCCTTCTGCCATAATTTCAACAGGAAAAGACAAGACGATACTG
TATGAAAGTCTGACCAACATAAAGGGTGCTACAAGGATGCCCCCAAGGCTCAGGATGTTGGAGAGTTGAG
GCACCATGAGTAACCACAATGCAGAATGAAGAATAGTTCTTCTGGTGTGAGGATCCACTGGGTTGCCACT
GACCCATTGCCTGAGGGTATGCGATGAAACCAGAATCAGCAACTGTAGATGGCAGCTCAGAAGGGGAAAC
GACACATCTAATAACCAACAGGAATGGCTATTAATTAGCTGAAATGACAATAATTAACAGAAATGACAAG
TCTAATAAATTTTCACTTAAGTGTTGTCACATGGCAAAAAAAAAAAAAAA

RNA
>LINC01477
GUCUCUGAGUCUCUGUCUCUCUCUGUCUCCUCACAGUCUGUUACUACUUAAUACAGUGCUUAUUGGCCUG
AGAUUGGACAUUAUUUAAAAGCAAUUUGGAGAGUUUUCUCCAUUUCAGGCAAGAAAACAUGCACUAUUUU
UCUGAUAUGGCAUGGGACAUCGAAAAAUAGUUUUGAACAAAAUUUUGAAAUUUUUGGCCCAAAGAAAGCA
AAUACCCCCUGCAACAAACAAGAUGUGUCACAAUAAGUCAUGAUUUACAAUCCCUUUCAAGACUGCCUGU
UGCCCUUAGCAAUCCCUAAAGCAAAAACAAACAAAACAAAUCUUUUCAGCAUAUCUUCAUGAAACUGUCU
GGGAUGUGACAAAACUUUGAGAAACAGUUUUGUGAUAAUGGUUUUGCACUUUAUUGGUACUUCUCCACAG
GAGGUGGUGAAUGGCUACUUUCAAAUACAUAGAGCUAUCAAAGGCUAAAGUCAAUAAAAAACCUCCAUAG
AAUAAUUGGAGAGGAAAGAAAAGGGGGAAAAAAAGAACAUAAAACGAAUGAAAAGGCAAGUAGGACCUAU
AAAUAUAAAAGCCUAAGGAGUAUAUUUUGGGAGAACAGAUGAUGCAAAGCAAAAUUGGGAAUAGUUAUUG
GAGGAUCAAGGAAGGCAAAUAAAACAAUAAGAUAUAUGAUGCCUGUCCUCCUGCCUCAAGUCUAGCUCAU
CAGACAGUUUUUCAUUCCAUUUUGUAUAUAUGAGUUUCAUCUGACUACUGCCAACAGAAAGUGCUUGGAU
UUCCUGACUUUUUUCACUGUCAAAAGAAAUAAUAAGCUGAUGAGAUUCAAAGUACUUCUCCUUCCUGCCA
CCGCGUGAAGAAGGAUGUGUUUGCUUCCCCUUCUGCCAUAAUUUCAACAGGAAAAGACAAGACGAUACUG
UAUGAAAGUCUGACCAACAUAAAGGGUGCUACAAGGAUGCCCCCAAGGCUCAGGAUGUUGGAGAGUUGAG
GCACCAUGAGUAACCACAAUGCAGAAUGAAGAAUAGUUCUUCUGGUGUGAGGAUCCACUGGGUUGCCACU
GACCCAUUGCCUGAGGGUAUGCGAUGAAACCAGAAUCAGCAACUGUAGAUGGCAGCUCAGAAGGGGAAAC
GACACAUCUAAUAACCAACAGGAAUGGCUAUUAAUUAGCUGAAAUGACAAUAAUUAACAGAAAUGACAAG
UCUAAUAAAUUUUCACUUAAGUGUUGUCACAUGGCAAAAAAAAAAAAAAA

DNA

>NR_110792.1 Homo sapiens long intergenic non-protein coding RNA 1478 (LINC01478), long non-coding RNA

GCACTTTTCTTTGTGCATTCACACGATGCATTTTCTTTTCTTATCACCAAACTGTTTTGAAAGATAAAAG
TACTTCCTGAATATGTGGATGGTGAGCCACGTGAATGGAAGACATGGAGATATGGACTGTTAACACCTTG
TCAAACATTTATTGTCCCTTCCACCTCTAAACTTCTGTGATAATAGGTGAACAGGACCCACGTGGTGCAG
AATACTGCGATACACACTGCGGAATAATTCAGTGCAGAACAACACGTGGATTGCTCAGTACAACTTGTTC
ATCACATCCTCCTGTTCACTGAAGAATTATGGAAATATTCTCTGAGCTGCTTTCCTCCCTGCCCAACGAG
TCATCTCTGTGTGAAAGGCCAAGCTTATAAATCCTGAAGAAAGAGACATTATACAAATGCAAGCTGTTGT
CACAACAATGATAAGAAATCAGGAGTCGAAAGATGTGGAGTAAGGGGGAATTTCAGAGAAACCACAAAAC
ACAAAATAGGAAACAGAGACAGGAGAAGATTGCTATGACAAAATTGCAGAAGGTGAAATCTTTGGCAGCC
TCTTATTTCTCTGGAGAATATGATATTGTCAGGCTGAGGTGGAAGAATCACATGGTCATACTGTTGAAGA
GGTCTTGAAACATTGGGCTTTCCTCTACAAGCGGAAGGATAGCACTGGCAGCCATCCACTGGCCCAGCAA
TATCCTGGATTGAGACGATATCATTTGCATTACTTGCTGAATGAAACATAGTGAAAATGACTGCAGATAA
CCTCAACTAAGTCCAAGATATCATTTTCAAATCTGGACCAGCCCTTGCCATCTAGACTGAACAGAGATTC
CAGTTTCCAGGAGAGTAGATGGAGTAATGATAAAAGCACCTCCCAAAACTTTTGCCTTGGTACTACAGAG
TGGCACATAACTAAACATTATCAAGGAATTTTACAGCCCTAGGCCTTCTATTACTGTGTATTACAGTCTG
CTGTAATCATTCTAATGGTCTAAGATGTTGCAAGTAATTTACTCACGGCCTGAACACCAGAAGTGCCAAA
ATTGCCTGGGAGTTTTATTCTGCATCCAACCAGTACCAGGAAGGAGGATGTATTACTTCTCGTTTCTCTA
CAAGTGTCAGTGCAAATCCAGCCATCAAACAAAACTTAGACCTAAAAGCTTTTAGATTACAGCGATTATT
GCAGATTTTACATGAGATAAAAATGTAATTGCTCCATTTCCTTTATTCTTTCTTTCATTTTCTTCCCTCT
CACTGTATATTTTAGATTGTCCTGCTTGACTGCCAGGTCAGACCCAGCACACAAAGATATCTGTCATAAG
ACTGGGGAGCTGCAGTGTTCTTTTTCATAAGAAATGAAATGCTTGAAATTTCTCTTCCAAATGAATCACA
AAACTTCCATGGTCATTTTCATTTTAAATTATTTTATGTCTATGTAAACTCATGCATTGCCTATAACGGA
ATAATATTCCCAGCCATGTGGATAGTGACAGAGAATGAGCTGAAGGCAGCAATACTTCACAGGCAAAGTG
GTACATGCATTTTTTTTTCATCTTGCTTTTTTCCTTTTCCATATTCACTCATCCTTTTAAATTCTTTGAA
TCTACTTTTTCCTCCCTCGCTCCCTTCCTATCCTCCATCTCTATCTTTGTCATTTATTAATTCTTTTTCT
TCTTTTTCTCCAGACTTGGATATTTCTCTATCCTCTCATCATTCTCTTTCATACACATTCTCTGACTTGT
AGGTCCTAAGATTCTCATATTCTAAAATTAACCCTCTGCTTAACAGGGTATGTTTATAAACCATTGGACT
GAGGAGGAATTGCCCAAGTTATCAAAGACATCCTATCAGGAATGTCAGCCTTTTACTCTCATTCATGCTC
AGAGAAACAGTGCTGCAGGGATTATAGCCTGGAATATGGCAGAACCAGATTTAAATTTGTTTGCCCTTT
GCCTCCTAATATCCATTTTCCCCTAAGTTTAACTGGGCATATTTCCCAGTTTGCCTTGCAAATGTGTTGC
CATATGACTGCACTTTGTCTAATAAAATGAAACTGAGTA

RNA

>LINC01478

GCACUUUUCUUUGUGCAUUCACACGAUGCAUUUUCUUUUCUUAUCACCAAACUGUUUUGAAAGAUAAAAG
UACUUCCUGAAUAUGUGGAUGGUGAGCCACGUGAAUGGAAGACAUGGAGAUAUGGACUGUUAACACCUUG
UCAAACAUUUAUUGUCCCUUCCACCUCUAAACUUCUGUGAUAAUAGGUGAACAGGACCCACGUGGUGCAG
AAUACUGCGAUACACACUGCGGAAUAAUUCAGUGCAGAACAACACGUGGAUUGCUCAGUACAACUUGUUC
AUCACAUCCUCCUGUUCACUGAAGAAUUAUGGAAAUAUUCUCUGAGCUGCUUUCCUCCCUGCCCAACGAG
UCAUCUCUGUGUGAAAGGCCAAGCUUAUAAAUCCUGAAGAAAGAGACAUUAUACAAAUGCAAGCUGUUGU
CACAACAAUGAUAAGAAAUCAGGAGUCGAAAGAUGUGGAGUAAGGGGGAAUUUCAGAGAAACCACAAAAC
ACAAAAUAGGAAACAGAGACAGGAGAAGAUUGCUAUGACAAAAUUGCAGAAGGUGAAAUCUUUGGCAGCC
UCUUAUUUCUCUGGAGAAUAUGAUAUUGUCAGGCUGAGGUGGAAGAAUCACAUGGUCAUACUGUUGAAGA
GGUCUUGAAACAUUGGGCUUUCCUCUACAAGCGGAAGGAUAGCACUGGCAGCCAUCCACUGGCCCAGCAA
UAUCCUGGAUUGAGACGAUAUCAUUUGCAUUACUUGCUGAAUGAAACAUAGUGAAAAUGACUGCAGAUAA
CCUCAACUAAGUCCAAGAUAUCAUUUUCAAAUCUGGACCAGCCCUUGCCAUCUAGACUGAACAGAGAUUC
CAGUUUCCAGGAGAGUAGAUGGAGUAAUGAUAAAAGCACCUCCCAAAACUUUUGCCUUGGUACUACAGAG
UGGCACAUAACUAAACAUUAUCAAGGAAUUUUACAGCCCUAGGCCUUCUAUUACUGUGUAUUACAGUCUG
CUGUAAUCAUUCUAAUGGUCUAAGAUGUUGCAAGUAAUUUACUCACGGCCUGAACACCAGAAGUGCCAAA
AUUGCCUGGGAGUUUUAUUCUGCAUCCAACCAGUACCAGGAAGGAGGAUGUAUUACUUCUCGUUUCUCUA
CAAGUGUCAGUGCAAAUCCAGCCAUCAAACAAAACUUAGACCUAAAAGCUUUUAGAUUACAGCGAUUAUU
GCAGAUUUUACAUGAGAUAAAAAUGUAAUUGCUCCAUUUCCUUUAUUCUUUCUUUCAUUUUCUUCCCUCU
CACUGUAUAUUUUAGAUUGUCCUGCUUGACUGCCAGGUCAGACCCAGCACACAAAGAUAUCUGUCAUAAG
ACUGGGGAGCUGCAGUGUUCUUUUUCAUAAGAAAUGAAAUGCUUGAAAUUUCUCUUCCAAAUGAAUCACA
AAACUUCCAUGGUCAUUUUCAUUUUAAAUUAUUUUAUGUCUAUGUAAACUCAUGCAUUGCCUAUAACGGA
AUAAUAUUCCCAGCCAUGUGGAUAGUGACAGAGAAUGAGCUGAAGGCAGCAAUACUUCACAGGCAAAGUG
GUACAUGCAUUUUUUUUUCAUCUUGCUUUUUUCCUUUUCCAUAUUCACUCAUCCUUUUAAAUUCUUUGAA
UCUACUUUUUCCUCCCUCGCUCCCUUCCUAUCCUCCAUCUCUAUCUUUGUCAUUUAUUAAUUCUUUUUCU
UCUUUUUCUCCAGACUUGGAUAUUUCUCUAUCCUCUCAUCAUUCUCUUUCAUACACAUUCUCUGACUUGU
AGGUCCUAAGAUUCUCAUAUUCUAAAAUUAACCCUCUGCUUAACAGGGUAUGUUUAUAAACCAUUGGACU
GAGGAGGAAUUGCCCAAGUUAUCAAAGACAUCCUAUCAGGAAUGUCAGCCUUUUACUCUCAUUCAUGCUC
AGAGAAACAGUGCUGCAGGGAUUAUAGCCUGGAAUAUGGCAGAACCAGAUUUAAAUUUGUUUGCCCUUU

GCCUCCUAAUAUCCAUUUUCCCCUAAGUUUAACUGGGCAUAUUUCCCAGUUUGCCUUGCAAAUGUGUUGC
CAUAUGACUGCACUUUGUCUAAUAAAAUGAAACUGAGUA

>NR_033983.1 Homo sapiens long intergenic non-protein coding RNA 1538 (LINC01538), long non-coding RNA

GGGATGCCCCGCAGGCCTGGACTGATCCACCAGGAGAACTTCAGTGCATCATGGGTCCTGCTATTGCTCA
TCCTCTTCCCTGGAAATGTGCGGTTTCACAGGATGGTAAACATCTTTCCCTGGAACAGAGCCTTGAGGAT
GCTATGGTGCAGTATGATGACCACCATCCCAGTCTGCCAAGGAATGAGTAACTTCCTGCAATTTAGGACT
TTCGGCCCTAAAACCAGTACAGTCCTGGACACACTAAGATTGTTGGTCACCCTAGAAGAATGGAGATCTG
GCGGCTACCACTCACATATACTCGTAGAACATTGATTGCAAGGTAACTTACTGGCTGAAGCAGGTGATGC
ATTGCTTCGGTGGGGCTGATGTCGAAAACCTTCAGCTTCCCAAGACAACACTGATCAGGCCTCCAGGAGC
ATTTCTCCTGCTCAGTGTCACAGTGCCACTGCAGAGCGCAATTCCTGGGGCACGTCGGACATTCAGTAAA
TACGTTTATTGAATGAATAAAACATAACACAGTAAGTAATTCATCTTAAAGTCAACCATTTACATTACTT
TTTATCCCTGAATACCCTTCCTCTACCTGTCAATTCAGCAGTCCAAGGGAAAAACCAGCTTGGTCAACAT
TAAATTTGGAGCTCCCACATCATATATTTTCATAGAACTAAACTAAGACATAGTTTAAAGAGAAAATGTT
TCACTCCTCACGTGACCATGTTCTCTTTGGACTGGCTTTTCCCCTTCCATTCCATATCTCTAAGTCCAGT
TTATCCTTTGAGTCCTGACTCACTCAAGCTTCTCCCCACTTCCTTCCTTGACCACTTGTTAAGTGTTTGC
ACCAGTAGTCTAGAAGTATAAAGGCATACACCTCATTATCACTACACACTGTTTCATAGGTAAGTCTTAA
TTTCAAATTAGTTCCTAACTCCTTGTTGACAATGACTATGACCAAGACGTCAATCCTCCTTTAGGGTCTA
AAACAGTACTGTGCATGTCAGAGGCATTCATTATATGCTTGTGAATTGACCAGAATAAATGACCGAATTG
AAGTCTGATTTTGGTCAATTCACCAGACAAAGGGCCACAGAAGCATTGCATCCTTTCATGACAGAATTAG
GGCATGATTCCATTTCTATATGAAATACTAGCTGTGTGTTGTTATTAAAATTATTTTCTGGCCCCACTGA
GAGTTGAAAGAAAAGTTGAAATTTAAAGGATTCCTTCTATTCATCATTTTTAAGAAATTTCATTTTTAGT
TATGAGGCAGTGTAAGGCATATTACCATTTTATTTGGACACTTATTTAGACCAGATTGCAAATTTTCTGT
TAATTGAGTGCTGAATTGGCCAACACACCATTGTAGATGTACTCCATAGCTTCTGCACATGGAATTTATT
ACAGACTTTTCATCCACATCTGCTCCTTTACTTTGCTTAATATACTATAGTATTTCTCTTAGATACAGGG
GAAGACAAAGAATATCAAATGACAAGTGAAGAAAAATTAACTTAGAATTATTCACAGTTAAATTGTATCT
ATACTCAGGTTATCTAATAAAATATTAAATAAACTATGTATCTTTTTTGTTGTCCTGTTATATACTTTCC
ATGTTTCTCATTACACAAATACACATTTAGAAAGCTGAAAATATGTAGATGAGCAAATACGAGGTTACCT
AAAATCCTATCATCCAGAGATGATAACTATTGACATTCTGCTATATATACTTCTAAACATTTTCTTGTAA
AACATAATTTTTAAATAGATTATATTCGTATTTTTGAGGAAAATATTTTTCAGTTAACAAGTTGCTATTT
CAAAATTAACACTGTAAATTCCTTTCTATGACAGTAAATACACATGTATATTTCATGTGAAATAGTTGTA
GCAACTTTTACTGTATAAACCCATGTAACATATTTAGCAAATCAATACATATTGTAGGAAACTTAGTTTC
CAATTTTCAATATGTTAAAATTTTGACAAGAATATTTTTGTGTAAACATTTTTATAAGTATATAATGCTT
CCTGAGGATAAATTCTAAAAAGAGGTCTTACTAAGTCAATATATATTTTTTCCTATAATTTTGACATGAT
TTGCGAATTTGCCCTTCAAAAAACTTATACCCATTTATACTCTGTTTCACCCTTCCTAAAAAGTACTAGT
TATCATCCTTCACTTTTCATGTTTAGAATCGGTTAGGTGAAAAATAGCATTTCCATTTACTATTTTTTAT
TATTTTCAACTACTGTTGAGGTTAAATATCTTTCTATGTGGTTTTGGGCCAATTTTATTTTTCTACTAGA
ATGATAATCTTTTATTGGTTTTTCAGACTATTACCTATATTCCTGAGTCTGACATATCAAAGTTTTTAAT
CCAATAAATAAAATATCATTTTAATAAAAAAAAAAAAA

RNA

>LINC01538

GGGAUGCCCCGCAGGCCUGGACUGAUCCACCAGGAGAACUUCAGUGCAUCAUGGGUCCUGCUAUUGCUCA
UCCUCUUCCCUGGAAAUGUGCGGUUUCACAGGAUGGUAAACAUCUUUCCCUGGAACAGAGCCUUGAGGAU
GCUAUGGUGCAGUAUGAUGACCACCAUCCCAGUCUGCCAAGGAAUGAGUAACUUCCUGCAAUUUAGGACU
UUCGGCCCUAAAACCAGUACAGUCCUGGACACACUAAGAUUGUUGGUCACCCUAGAAGAAUGGAGAUCUG
GCGGCUACCACUCACAUAUACUCGUAGAACAUUGAUUGCAAGGUAACUUACUGGCUGAAGCAGGUGAUGC
AUUGCUUCGGUGGGGCUGAUGUCGAAAACCUUCAGCUUCCCAAGACAACACUGAUCAGGCCUCCAGGAGC
AUUUCUCCUGCUCAGUGUCACAGUGCCACUGCAGAGCGCAAUUCCUGGGGCACGUCGGACAUUCAGUAAA
UACGUUUAUUGAAUGAAUAAAACAUAACACAGUAAGUAAUUCAUCUUAAAGUCAACCAUUUACAUUACUU
UUUAUCCCUGAAUACCCUUCCUCUACCUGUCAAUUCAGCAGUCCAAGGGAAAAACCAGCUUGGUCAACAU
UAAAUUUGGAGCUCCCACAUCAUAUAUUUUCAUAGAACUAAACUAAGACAUAGUUUAAAGAGAAAAUGUU
UCACUCCUCACGUGACCAUGUUCUCUUUGGACUGGCUUUUCCCCUUCCAUUCCAUAUCUCUAAGUCCAGU
UUAUCCUUUGAGUCCUGACUCACUCAAGCUUCUCCCCACUUCCUUCCUUGACCACUUGUUAAGUGUUUGC
ACCAGUAGUCUAGAAGUAUAAAGGCAUACACCUCAUUAUCACUACACACUGUUUCAUAGGUAAGUCUUAA
UUUCAAAUUAGUUCCUAACUCCUUGUUGACAAUGACUAUGACCAAGACGUCAAUCCUCCUUUAGGGUCUA
AAACAGUACUGUGCAUGUCAGAGGCAUUCAUUAUAUGCUUGUGAAUUGACCAGAAUAAAUGACCGAAUUG
AAGUCUGAUUUUGGUCAAUUCACCAGACAAAGGGCCACAGAAGCAUUGCAUCCUUUCAUGACAGAAUUAG
GGCAUGAUUCCAUUUCUAUAUGAAAUACUAGCUGUGUGUUGUUAUUAAAAUUAUUUUCUGGCCCCACUGA
GAGUUGAAAGAAAAGUUGAAAUUUAAAGGAUUCCUUCUAUUCAUCAUUUUUAAGAAAUUUCAUUUUUAGU
UAUGAGGCAGUGUAAGGCAUAUUACCAUUUUAUUUGGACACUUAUUUAGACCAGAUUGCAAAUUUUCUGU
UAAUUGAGUGCUGAAUUGGCCAACACACCAUUGUAGAUGUACUCCAUAGCUUCUGCACAUGGAAUUUAUU
ACAGACUUUUCAUCCACAUCUGCUCCUUUACUUUGCUUAAUAUACUAUAGUAUUUCUCUUAGAUACAGGG
GAAGACAAAGAAUAUCAAAUGACAAGUGAAGAAAAAUUAACUUAGAAUUAUUCACAGUUAAAUUGUAUCU
AUACUCAGGUUAUCUAAUAAAAUAUUAAAUAAACUAUGUAUCUUUUUUGUUGUCCUGUUAUAUACUUUCC
AUGUUUCUCAUUACACAAAUACACAUUUAGAAAGCUGAAAAUAUGUAGAUGAGCAAAUACGAGGUUACCU

AAAAUCCUAUCAUCCAGAGAUGAUAACUAUUGACAUUCUGCUAUAUAUACUUCUAAACAUUUUCUUGUAA
AACAUAAUUUUUAAAUAGAUUAUAUUCGUAUUUUUGAGGAAAAUAUUUUUCAGUUAACAAGUUGCUAUUU
CAAAAUUAACACUGUAAAUUCCUUUCUAUGACAGUAAAUACACAUGUAUAUUUCAUGUGAAAUAGUUGUA
GCAACUUUUACUGUAUAAACCCAUGUAACAUAUUUAGCAAAUCAAUACAUAUUGUAGGAAACUUAGUUUC
CAAUUUUCAAUAUGUUAAAAUUUUGACAAGAAUAUUUUUGUGUAAACAUUUUUAUAAGUAUAUAAUGCUU
CCUGAGGAUAAAUUCUAAAAAGAGGUCUUACUAAGUCAAUAUAUAUUUUUUCCUAUAAUUUUGACAUGAU
UUGCGAAUUUGCCCUUCAAAAAACUUAUACCCAUUUAUACUCUGUUUCACCCUUCCUAAAAAGUACUAGU
UAUCAUCCUUCACUUUUCAUGUUUAGAAUCGGUUAGGUGAAAAAUAGCAUUUCCAUUUACUAUUUUUUAU
UAUUUUCAACUACUGUUGAGGUUAAAUAUCUUUCUAUGUGGUUUUGGGCCAAUUUUAUUUUUCUACUAGA
AUGAUAAUCUUUUAUUGGUUUUUCAGACUAUUACCUAUAUUCCUGAGUCUGACAUAUCAAAGUUUUUAAU
CCAAUAAAUAAAAUAUCAUUUUAAUAAAAAAAAAAAAA

>NR_040025.1 Homo sapiens long intergenic non-protein coding RNA 1539 (LINC01539), transcript variant 1, long non-coding RNA
ATAGATGAATAGAATGAGCAAAAGTTGCAGCACAGCGTGTCACCAGAGAGGAAAAGAATATCCTTGCTGT
GCCAAGAAAGAAAAGACCATGGATCTACTAGTTGCAGATTTCAGCAGTAATGCCAAGGGGGCAAGAAGTG
ACCAGCAGAACAATTAGGCCATGGCAACAGAAGCCAACTACAACCCATGTTCCAATCTATGACCTCCCTC
TCCTGCTGGTACAAGGATATGAATGTGGCTGTAGCATCATTTTTGGAAGCCAGAATGGTAATACTGAAAA
TCACTGCTTGTCTCAAGATCCAACTTCTGGAAATCTCAACCATTCTGAATACAGCCATCATTCTTAAAAT
TGAAGAGAACTGTTTTACAAGATCTTGACTTGTAAAGAAACCAAGTTTATTAAGGGCAAGAGCTGATAAT
CAACTGACTAATGAGGATTGACTTCTACAATTGATGAGCATGGAATTGGATGTGCCTAAATCAACAGAAG
AGGGATTTTGATGTCATCACACTGTAGCCCCAGAAGACAGAACGCTCAAAGGTGTCAATGGGCAGAATTT
GTAGATGACATACAGCATCCAATGGATTACTCCTATTCAGGATAATGGAAGAAAAAGGTTCTTACCCTTC
AGCAGATAGCATGCTGAGCCCTGAACAAATTTTATGACTTCTGGTGCAAAGCAGACCCAGTCAGCTTGCC
TCTAGCAACTGGTTAATACTACTCCAAGGAAACCACACTTAAAATTCTTGGAAGTTCTTTTACCATCACA
TCAAGTTGCATTTTGAAGCTGAAGCCCTTCGAATCCATTTATACCTTGTGATATTCCCTTGCCAGATTGT
TGTTTTGTTTGCTAGACATGATTTTAGGATTTTGTCAAGGAAGCCATCTATGACCCCAATCAATCAAAAC
CATAAGGATCAAGGCTGGAAGAACCTATTCCTTAGAGCAAAGGATGCAATGTCAAGCAAGTTGTGATACC
TCAACTATCTACAATTTAACACCAAGGAAAGAACACTGTTTGGAGAAATGAGAAAGATTATCATCAGTGT
CTTTCAGAAGTCATCAAAAGAAGAAAAGGAGAAAAGAGAGGAGAAAGCAGAAAGATCTGGAGTCTGGGAA
GTTCAAGAGCATGGCACCAACATCTGGCAAGGGCTTTCATTCTGCATCATCTCATGGAGGAAAATAAAAA
GATTTCAGGTAAATCCAGATTTGGAGCTTAAATGACTCAAATGCTTTGCCTACATTGATCCTACATTCAG
GCTGCTTACATATCTCTTGGCTTCTCTGAGAACCAGATTGTCACCACTTAAAGTGTCTTCCAGAGTCCTC
ACGAGTATCAGAGAAGGCATAATCTGTACACGTGGCTTCATTTAAAATGAAAATATCAAGCAGTGTCATT
TTCACAAGTATCCCCACACTGTCTGAGGCTGAAAAGAAGAGTGTAGCTATGCACACTGCAGTGAGAAGAA
AATTTCAGCTCAATTGGAGGATGTAAAGATTGAAATACCACTGCTCCTAGTTCTGATGCAAAATTGAAAA
GAGTGCACTGGGTCTTCAAATGGCATATTGACAAGGAGAAAGAGGAAAGGATGCCCCAGGACAGCCTGGG
GGTCAAGGCTGGGCTCAGGCATGCCTGGACAAGCCATGCAAGTTGTCTCCGGACTTGAACCACGTATCAG
AGGAGCTATTTGAGGAGCCACATCAGCATACCTGCTTCTGTGGGCCCCCAGGCTGTGGTGCTCAGCCCTT
GCACTATGAAACATTTTTTTTTTCCAGAAGCCAAAGCTTTGAGCATCTCCTCCCATGTAGAGAGTGTATA
TGTTTACGTCCATGTCTTTCGCCATGTGAACGCATGCAACCACCACCTCAAATATCATAAATCCTCTCTC
TCCCCCTTTCTGTCTGCCTGTTTATCTACAATCTGTCTATTTAAATTCTTTTTAAACAAGAAGGGAATAT
CTTTATGCCTGTTTGTAGGGATATTCTACATCCATCTGTTATTTACATCATTCGTTTTCCTGAGGTGTCT
CCAACAAATCCTGGCTGGATGCACTGTATGTGTTATTAATAAAACAGCTGTAAAAATATTAAC

RNA
>LINC01539
AUAGAUGAAUAGAAUGAGCAAAAGUUGCAGCACAGCGUGUCACCAGAGAGGAAAAGAAUAUCCUUGCUGU
GCCAAGAAAGAAAAGACCAUGGAUCUACUAGUUGCAGAUUUCAGCAGUAAUGCCAAGGGGGCAAGAAGUG
ACCAGCAGAACAAUUAGGCCAUGGCAACAGAAGCCAACUACAACCCAUGUUCCAAUCUAUGACCUCCCUC
UCCUGCUGGUACAAGGAUAUGAAUGUGGCUGUAGCAUCAUUUUUGGAAGCCAGAAUGGUAAUACUGAAAA
UCACUGCUUGUCUCAAGAUCCAACUUCUGGAAAUCUCAACCAUUCUGAAUACAGCCAUCAUUCUUAAAAU
UGAAGAGAACUGUUUUACAAGAUCUUGACUUGUAAAGAAACCAAGUUUAUUAAGGGCAAGAGCUGAUAAU
CAACUGACUAAUGAGGAUUGACUUCUACAAUUGAUGAGCAUGGAAUUGGAUGUGCCUAAAUCAACAGAAG
AGGGAUUUUGAUGUCAUCACACUGUAGCCCCAGAAGACAGAACGCUCAAAGGUGUCAAUGGGCAGAAUUU
GUAGAUGACAUACAGCAUCCAAUGGAUUACUCCUAUUCAGGAUAAUGGAAGAAAAAGGUUCUUACCCUUC
AGCAGAUAGCAUGCUGAGCCCUGAACAAAUUUUAUGACUUCUGGUGCAAAGCAGACCCAGUCAGCUUGCC
UCUAGCAACUGGUUAAUACUACUCCAAGGAAACCACACUUAAAAUUCUUGGAAGUUCUUUUACCAUCACA
UCAAGUUGCAUUUUGAAGCUGAAGCCCUUCGAAUCCAUUUAUACCUUGUGAUAUUCCCUUGCCAGAUUGU
UGUUUUGUUUGCUAGACAUGAUUUUAGGAUUUUGUCAAGGAAGCCAUCUAUGACCCCAAUCAAUCAAAAC
CAUAAGGAUCAAGGCUGGAAGAACCUAUUCCUUAGAGCAAAGGAUGCAAUGUCAAGCAAGUUGUGAUACC
UCAACUAUCUACAAUUUAACACCAAGGAAAGAACACUGUUUGGAGAAAUGAGAAAGAUUAUCAUCAGUGU
CUUUCAGAAGUCAUCAAAAGAAGAAAAGGAGAAAAGAGAGGAGAAAGCAGAAAGAUCUGGAGUCUGGGAA
GUUCAAGAGCAUGGCACCAACAUCUGGCAAGGGCUUUCAUUCUGCAUCAUCUCAUGGAGGAAAAUAAAAA
GAUUUCAGGUAAAUCCAGAUUUGGAGCUUAAAUGACUCAAAUGCUUUGCCUACAUUGAUCCUACAUUCAG
GCUGCUUACAUAUCUCUUGGCUUCUCUGAGAACCAGAUUGUCACCACUUAAAGUGUCUUCCAGAGUCCUC
ACGAGUAUCAGAGAAGGCAUAAUCUGUACACGUGGCUUCAUUUAAAAUGAAAAUAUCAAGCAGUGUCAUU
UUCACAAGUAUCCCCACACUGUCUGAGGCUGAAAAGAAGAGUGUAGCUAUGCACACUGCAGUGAGAAGAA
AAUUUCAGCUCAAUUGGAGGAUGUAAAGAUUGAAAUACCACUGCUCCUAGUUCUGAUGCAAAAUUGAAAA
GAGUGCACUGGGUCUUCAAAUGGCAUAUUGACAAGGAGAAAGAGGAAAGGAUGCCCCAGGACAGCCUGGG
GGUCAAGGCUGGGCUCAGGCAUGCCUGGACAAGCCAUGCAAGUUGUCUCCGGACUUGAACCACGUAUCAG
AGGAGCUAUUUGAGGAGCCACAUCAGCAUACCUGCUUCUGUGGGCCCCCAGGCUGUGGUGCUCAGCCCUU
GCACUAUGAAACAUUUUUUUUUUCCAGAAGCCAAAGCUUUGAGCAUCUCCUCCCAUGUAGAGAGUGUAUA
UGUUUACGUCCAUGUCUUUCGCCAUGUGAACGCAUGCAACCACCACCUCAAAUAUCAUAAAUCCUCUCUC
UCCCCCUUUCUGUCUGCCUGUUUAUCUACAAUCUGUCUAUUUAAAUUCUUUUUAAACAAGAAGGGAAUAU
CUUUAUGCCUGUUUGUAGGGAUAUUCUACAUCCAUCUGUUAUUUACAUCAUUCGUUUUCCUGAGGUGUCU

CCAACAAAUCCUGGCUGGAUGCACUGUAUGUGUUAUUAAUAAAACAGCUGUAAAAAUAUUAAC

>NR_038325.1 Homo sapiens long intergenic non-protein coding RNA 1541 (LINC01541), transcript variant 1, long non-coding RNA
ACAGAAAGAGCCAGGATCCCAGACTGTGCTGAAGCTTCATGAGACCTCTCCTCGTCTGTGCACGAAAGAG
GAGCCGGACACAGAGTGTCCAGCCGACTCTGGAGCCCAGGCTGTTGCTTCCCGGTCTGGTGGTGAGTCCT
CCATTGTCTGATGTGGGTAAGTCCAAGACCAAGGAGCCAGCAGATTCAGAGTCCAGGGAGGGCTGTTCCT
GATTTGCAGATGGCGCCTTCTCTTTATGTCTTCACAAGATTGTGAAGCCTGTAGCTTGGAAGATACAGGA
CATAGGTGAAAGTGTTTGTACCATTCATTTAAAGGCATAAATGTCCAATGGGTCATAAAGGTCAAAGCTA
ATAGACAAAAAAAGCTTCCAGCATGGATGATGAGCAAATTAGATGCCTCTCTGCTCAGCATGAGGTTTTT
TCAGCTGCAAAAGTAAAATGAATCAACCATGGAATTACCCCAATGCACTAATAGCTACCGCTGTAGTGGT
AGATCGTTATATATCTGGGCAATTAAATTTTAAATGCTAACTGTGAGACCAAGTCAGCAAATTCTAATTG
AGACACGTTCTACAAAACACCTGACCACTACTCCTGAAACAGTCAAGGTCTTCAGAAATAAAGAAGGTTT
AAAAAAATTATTACAGTCAAGATACACGGCAACTAAATGTGATGTGGTATACCGTATGGGATACAGAAAC
TGAAAAAGGACATTAGGGCCATCTTGTGATACATGCACTGTCACAGGAAAAGTTTCCAGGAAAAGTCAAG
CCAGGGCTTCACAGAAGATCAAAAGAAAGAGACAAGGAGAAGCTAAGTGTATTAGTCAGGATTCTCTAAG
GGACAGAACTAATAGAATATATATACACACACAAACACACACACACACACATATATATACATATAAATAA
AAGGGAGTTTATTAAGGAGTATTAACTCACACAATCACAAGGTCCCACAACAGGCCGGGCCATCTGTAAG
CTGAGGAACAAGGAAGCCAGTCTGAATCCCAAAGATGACAAACTTGGAGTTCAATGTTCAAGGGCAGGAA
GCATCCAGCACAGGAGATAGATGTAGGCTGGGAGGCTAAGCCAGTCAAGACTTTTCACGTTTTTCTGCCT
GTTTCATATCCTAGCTGCAATGGCAGCTGATTAGATGGTGCCCACCCAGATTAAGGGTGGGTCTGCCTTT
CCCAGCACACTGACTCAAATGTTTATCTCCTTTGGCAACACCCTCACAGACACACTCAGGATTAATATTT
TGCTTCCTTCCATCCAATCAAGTTGACACTCAGTATTAACCATCACACTAAGCTTGTTATTTTATAGAAA
TGCTTGAAAACCAGAGATAAATGCAGCTACAAATGAACGATGACTCTGCGTTTTCCTTGTATATATAAAG
ATCAACAATAACACTTAGGTCATGAAACCCACCTACTTTACAGATCATTATGGAAATAATACACTGCTTC
AGCAATGGAGAAACAATCATCCATATTTATGCAAGAACTAAATTCAAGTTTGAAAGCTTGCTTTGAGCTT
GAAGATATAAAACATGATTCTGTGACAGTAAACAATGTATAACAAATCTTACAGAAATTTATGTGATTAC
TAAAAAAAAATCAATTCATCAATTCAATAAACTGAGTACCGTATTACTTTTCTATTGCTTCTTTACCAAA
TAAATACAAGCTTTGCAGTTTAAAACAACAGAAATTTAATTTTTTATAGTTCTGAGGGTCAGTAACCTAA
AACTGATCTTATGAAATAAAGTTAAATCTTGGGGATATGGGTGTGGACAGGGCTGGTTTCTTCTGGGGCT
CTGATGGTAAAATTTGCTTCCTTGCCTTTTCTCCTTCTAGGGAGCCTGCATTCCCTGCCTCCTAGTGGCT
TCCTCCCATCACTCGAACCTCTGTTTCCAGCCTCAGGATTCCTAATGTGACTCTGACGTTTCATCTCCTG
TTTATGAGGACGCTTCTGATTGCTAAGATACATACGGTGACACTATGGAAGTTAACTTGAAGATTAAAAC
CCTGAAAAATAGAAAGAATCTTCTATGGTATATCACAGAGATTACAAGGGCCTGAATTAGAGTGGTGACA
TTTTCATCCCTTGATAATGGATTAAAAACAATTGAATGAAGCTTTGGGGTGAACTTACAGGACTTTATGG
CAAACTTCATATGGGAGACTTACATGAAGGGAGGAAGAAATGCGTATGTGTACAAAATCATGTGGATAGA
TGTGCATTTGTGGGTATCTGTGAATACAAATACACAATTTTCAGACAGAAGTAGTATAAGAAAAGGAAAT
CAAACAAAGTCAAACATTTTAAGTGTAGCTCTTGGTGCACTTTAAAAAATTATTAAATTGAAGCTGTAGT
TTATTCTCATCATTGTCATCCTATATTAGTGATTTTCTTTAGCTTTTGAAATTAAATTCATAATATTACA
ATTCTGAATTCCTATGTATGTATATGTTAAGAATCCTTTAATGGGACTGACTTTCATATGTGTGTGGCTG
CATTCTGAAATAGTTGTCTAGTTGATACATAAAGCAGTGAGTGCTATTTGTAAGATATATTTATATTTAA
AGTCTTCATACAGTTATTAACTAGCTCCAATATTACTGAAATGATTTCTCCAAATATCCTATATTTAATT
ATCATGCTGGCAAATTTATTATAACTTTGGTTTGAAAAAATATATTATGATACATATCCACTGACTAATG
CCACCTATCTGCTTTCCTGAATTCAAAGTTTATGATTATTTCCATAAATGTATTTATCAGTATAATCAAA
AATAAAAATAACATAAATAAAATTTCTGTGAGCTTTACACATCTAATGTGCAATGATTATAAATCTGGTC
TTATACACATGGTAAAAAACATTTAAGTCACAAAAGTATACTTCAGGTTTAAGTATAAATCTGAAATTAA
ATTCTCAGATTTAAGTATAATTTTTAAAAGCTTTGGTAATTGTGTCCCTACTGTTAAATTTATTGTATAG
TTTTCACTATATCATCTGACCTTTTAATGTTTCTAGAAACTTGTGTGAGGTCTCATACTTCATGGCTAAT
AAAGTATTTATTTTTCTATTCTTAAAAAAAAAAAAAAAAAAA

RNA
>LINC01541
ACAGAAAGAGCCAGGAUCCCAGACUGUGCUGAAGCUUCAUGAGACCUCUCCUCGUCUGUGCACGAAAGAG
GAGCCGGACACAGAGUGUCCAGCCGACUCUGGAGCCCAGGCUGUUGCUUCCCGGUCUGGUGGUGAGUCCU
CCAUUGUCUGAUGUGGGUAAGUCCAAGACCAAGGAGCCAGCAGAUUCAGAGUCCAGGGAGGGCUGUUCCU
GAUUUGCAGAUGGCGCCUUCUCUUUAUGUCUUCACAAGAUUGUGAAGCCUGUAGCUUGGAAGAUACAGGA
CAUAGGUGAAAGUGUUUGUACCAUUCAUUUAAAGGCAUAAAUGUCCAAUGGGUCAUAAAGGUCAAAGCUA
AUAGACAAAAAAAGCUUCCAGCAUGGAUGAUGAGCAAAUUAGAUGCCUCUCUGCUCAGCAUGAGGUUUUU
UCAGCUGCAAAAGUAAAAUGAAUCAACCAUGGAAUUACCCCAAUGCACUAAUAGCUACCGCUGUAGUGGU
AGAUCGUUAUAUAUCUGGGCAAUUAAAUUUUAAAUGCUAACUGUGAGACCAAGUCAGCAAAUUCUAAUUG
AGACACGUUCUACAAAACACCUGACCACUACUCCUGAAACAGUCAAGGUCUUCAGAAAUAAAGAAGGUUU
AAAAAAAUUAUUACAGUCAAGAUACACGGCAACUAAAUGUGAUGUGGUAUACCGUAUGGGAUACAGAAAC
UGAAAAAGGACAUUAGGGCCAUCUUGUGAUACAUGCACUGUCACAGGAAAAGUUUCCAGGAAAAGUCAAG
CCAGGGCUUCACAGAAGAUCAAAAGAAAGAGACAAGGAGAAGCUAAGUGUAUUAGUCAGGAUUCUCUAAG
GGACAGAACUAAUAGAAUAUAUAUACACACACAAACACACACACACACACAUAUAUAUACAUAUAAAUAA
AAGGGAGUUUAUUAAGGAGUAUUAACUCACACAAUCACAAGGUCCCACAACAGGCCGGGCCAUCUGUAAG

CUGAGGAACAAGGAAGCCAGUCUGAAUCCCAAAGAUGACAAACUUGGAGUUCAAUGUUCAAGGGCAGGAA
GCAUCCAGCACAGGAGAUAGAUGUAGGCUGGGAGGCUAAGCCAGUCAAGACUUUUCACGUUUUUCUGCCU
GUUUCAUAUCCUAGCUGCAAUGGCAGCUGAUUAGAUGGUGCCCACCCAGAUUAAGGGUGGGUCUGCCUUU
CCCAGCACACUGACUCAAAUGUUUAUCUCCUUUGGCAACACCCUCACAGACACACUCAGGAUUAAUAUUU
UGCUUCCUUCCAUCCAAUCAAGUUGACACUCAGUAUUAACCAUCACACUAAGCUUGUUAUUUUAUAGAAA
UGCUUGAAAACCAGAGAUAAAUGCAGCUACAAAUGAACGAUGACUCUGCGUUUUCCUUGUAUAUAUAAAG
AUCAACAAUAACACUUAGGUCAUGAAACCCACCUACUUUACAGAUCAUUAUGGAAAUAAUACACUGCUUC
AGCAAUGGAGAAACAAUCAUCCAUAUUUAUGCAAGAACUAAAUUCAAGUUUGAAAGCUUGCUUUGAGCUU
GAAGAUAUAAAACAUGAUUCUGUGACAGUAAACAAUGUAUAACAAAUCUUACAGAAAUUUAUGUGAUUAC
UAAAAAAAAAUCAAUUCAUCAAUUCAAUAAACUGAGUACCGUAUUACUUUUCUAUUGCUUCUUUACCAAA
UAAAUACAAGCUUUGCAGUUUAAAACAACAGAAAUUUAAUUUUUUAUAGUUCUGAGGGUCAGUAACCUAA
AACUGAUCUUAUGAAAUAAAGUUAAAUCUUGGGGAUAUGGGUGUGGACAGGGCUGGUUUCUUCUGGGGCU
CUGAUGGUAAAAUUUGCUUCCUUGCCUUUUCUCCUUCUAGGGAGCCUGCAUUCCCUGCCUCCUAGUGGCU
UCCUCCCAUCACUCGAACCUCUGUUUCCAGCCUCAGGAUUCCUAAUGUGACUCUGACGUUUCAUCUCCUG
UUUAUGAGGACGCUUCUGAUUGCUAAGAUACAUACGGUGACACUAUGGAAGUUAACUUGAAGAUUAAAAC
CCUGAAAAAUAGAAAGAAUCUUCUAUGGUAUAUCACAGAGAUUACAAGGGCCUGAAUUAGAGUGGUGACA
UUUUCAUCCCUUGAUAAUGGAUUAAAAACAAUUGAAUGAAGCUUUGGGGUGAACUUACAGGACUUUAUGG
CAAACUUCAUAUGGGAGACUUACAUGAAGGGAGGAAGAAAUGCGUAUGUGUACAAAAUCAUGUGGAUAGA
UGUGCAUUUGUGGGUAUCUGUGAAUACAAAUACACAAUUUUCAGACAGAAGUAGUAUAAGAAAAGGAAAU
CAAACAAAGUCAAACAUUUUAAGUGUAGCUCUUGGUGCACUUUAAAAAAUUAUUAAAUUGAAGCUGUAGU
UUAUUCUCAUCAUUGUCAUCCUAUAUUAGUGAUUUUCUUUAGCUUUUGAAAUUAAAUUCAUAAUAUUACA
AUUCUGAAUUCCUAUGUAUGUAUAUGUUAAGAAUCCUUUAAUGGGACUGACUUUCAUAUGUGUGUGGCUG
CAUUCUGAAAUAGUUGUCUAGUUGAUACAUAAAGCAGUGAGUGCUAUUUGUAAGAUAUAUUUAUAUUUAA
AGUCUUCAUACAGUUAUUAACUAGCUCCAAUAUUACUGAAAUGAUUUCUCCAAAUAUCCUAUAUUUAAUU
AUCAUGCUGGCAAAUUUAUUAUAACUUUGGUUUGAAAAAAUAUAUUAUGAUACAUAUCCACUGACUAAUG
CCACCUAUCUGCUUUCCUGAAUUCAAAGUUUAUGAUUAUUUCCAUAAAUGUAUUUAUCAGUAUAAUCAAA
AAUAAAAAUAACAUAAAUAAAAUUUCUGUGAGCUUUACACAUCUAAUGUGCAAUGAUUAUAAAUCUGGUC
UUAUACACAUGGUAAAAAACAUUUAAGUCACAAAAGUAUACUUCAGGUUUAAGUAUAAAUCUGAAAUUAA
AUUCUCAGAUUUAAGUAUAAUUUUUAAAAGCUUUGGUAAUUGUGUCCCUACUGUUAAAUUUAUUGUAUAG
UUUUCACUAUAUCAUCUGACCUUUUAAUGUUUCUAGAAACUUGUGUGAGGUCUCAUACUUCAUGGCUAAU
AAAGUAUUUAUUUUUCUAUUCUUAAAAAAAAAAAAAAAAAAAA

>NR_110762.1 Homo sapiens long intergenic non-protein coding RNA 1543 (LINC01543), long non-coding RNA

GAGCAGTTGGTTTCGTTGTCTTAATTTGGGGCAGTAATGATTTCTGAGAAGCAGGTGATTGATAACGCGC
AAATTCAAAGCCCCTTGGGGGCTGCATTGTAGCATGGTGTGGGGATGGGGAGACAGAGGGCTGGTAGGAT
CCCGAATTCCTAGTTTTTCTCTCCCTCTCCCTTGCTTCTTCATTTTCTTCCTTCAGGCTTTCTTCCATTT
CTTTCTTCCATCAAATATTTGTGGAGCTGGAACTGGGCTTCACTATCATATTCCAAGGCCCTGTACTGGG
AACAGACAAAGAAGCTCCTCCCAGAGACTGGAAGAATGGTACAAGGTGTGAAAACCAAGGCTTAAAGCAT
GCAAGTTATTTGCCCGAGGTTTTTCAGCCACAAATGAAGAAGCTGGGTCTGCTCCACACCTGTGAACCAG
GCTATTTCCACCATAACCTGAACTCAGATGAGCACCAGCCCATGGAGAATAGTCCAGAAGGCTGCCCATA
GCCTTAAAGGACAGTCAGCCTCACAAAATGAGTGTTATATTCTGGATTATCAGAAGAGAAATGTGAGATG
CAGGCAGGTTGAGAAAATTGCTCCAAGTCACTTCGGGAATGTGTGCATGGACAAGGAAGCAGGAAGATCC
CAAGGAAAAAGAAGCAGCATGAAAAATGACAAGAAGACTACAACAGAAGTTTCTACGAACTTTGCACACA
CAGAAGAGGGTTCTTTTAGTTTTAGTCAGGATAGGAAAAGGACAAAATAAAAGTGAACATTTATTGAGCC

RNA

>LINC01543

GAGCAGUUGGUUUCGUUGUCUUAAUUUGGGGCAGUAAUGAUUUCUGAGAAGCAGGUGAUUGAUAACGCGC
AAAUUCAAAGCCCCUUGGGGGCUGCAUUGUAGCAUGGUGUGGGGAUGGGGAGACAGAGGGCUGGUAGGAU
CCCGAAUUCCUAGUUUUUCUCUCCCUCUCCCUUGCUUCUUCAUUUUCUUCCUUCAGGCUUUCUUCCAUUU
CUUUCUUCCAUCAAAUAUUUGUGGAGCUGGAACUGGGCUUCACUAUCAUAUUCCAAGGCCCUGUACUGGG
AACAGACAAAGAAGCUCCUCCCAGAGACUGGAAGAAUGGUACAAGGUGUGAAAACCAAGGCUUAAAGCAU
GCAAGUUAUUUGCCCGAGGUUUUUCAGCCACAAAUGAAGAAGCUGGGUCUGCUCCACACCUGUGAACCAG
GCUAUUUCCACCAUAACCUGAACUCAGAUGAGCACCAGCCCAUGGAGAAUAGUCCAGAAGGCUGCCCAUA
GCCUUAAAGGACAGUCAGCCUCACAAAAUGAGUGUUAUAUUCUGGAUUAUCAGAAGAGAAAUGUGAGAUG
CAGGCAGGUUGAGAAAAUUGCUCCAAGUCACUUCGGGAAUGUGUGCAUGGACAAGGAAGCAGGAAGAUCC
CAAGGAAAAAGAAGCAGCAUGAAAAAUGACAAGAAGACUACAACAGAAGUUUCUACGAACUUUGCACACA
CAGAAGAGGGUUCUUUUAGUUUUAGUCAGGAUAGGAAAAGGACAAAAUAAAAGUGAACAUUUAUUGAGCC

>NR_103449.1 Homo sapiens long intergenic non-protein coding RNA 1544 (LINC01544), long non-coding RNA
ATTCTCTAGGCGAAGGAGAACAATAAGAGGCAGGTAAAGCTGGAGCCCGTAACAGGAGGAAACGTGAGCA
TTGAAAACCCGCCCTTTGGATGAACTGACCACAGCTGGACAACACCAGTGCCAGGCCAGGGTTCCAAGCA
CTGCAGAGATGAAGCACCTTCTTCCAGGAGTTCCTGGAGTTCTCAGAAGCCAGAGTGGACTTGTTCCTCC
GTTTTATTTGATGAGGTTCTAAATATAGCTGGGCAAGGCTTCGGATTTTAGTGGGAGATGGCCAGAGGCG
AAGCAACTGATAAAAGTAGCTGAATAGCTCTCCCTCTCTGAGCTAGGTGCAAGCTCCTGACGTAGGAAGC
GATCCCTCCTTGCCACAGATAACCCACGTAGCCAGGTGCTGCCAAGCAGCTTCCACCTGGAGGAGGGCAG
CTGCTCTGCGCATGACCAGTAGCTGTGGATGGCATCGTCGAGGCATCTGGAGGAAGCTGCAGCCCCACAA
GGCCCCTGACCCCTCTGCCTTTCCCTCTGCTGGCTCCTCTTCCTCCCTCTTTCCCTGCTGTCCCCTGTAG
AGAACTTTCTACCAGTCCACACACCAACCTAGGTGCAGCCCCCCTGCGTGACTCCCTCCCTGACTATCAT
AACCATGGCCAGCAGTATCTCCACATCTCTGCCCAGATCCCCCTGGGAAGGCTTATCCCAGGCCCCAGGT
TTGGGGCAATGCCCAGTGGCATCGGATGGGGTCCCCCTTGAAAGGAAGCACAGAAAGACTGAGCCATCAA
CGTAGTCCCCTCCATCACCTGCCACTGTCCCCAAAATCTCCCACACCGGAAGTGTTTAGATTGTCTAAGG
ACAATATTGTCTAGGGTTTGAGGACCACCCCACAGCAAAAACACTAGAAAGCTAGTTCCAAAATGCAAAT
CCCAGGCCAAGCCCCTCACCTCCTGAGGCAGCATCCCTGGGTATAGATCTCAGAAATCTGCAGTTTTAAT
ACAAAGGCCCCCTAAACTTAAAGAAACAGCCTGAATTCACATGGGCACACCTTACCTGAGCAGGCAATAC
AGTGACAAGGGTGGAAACAGAGGGGACGAGACCTGACACTGGGAAGGCCTGACAGAGGGAAGGAAGGAAA
GCTCTCAGGCTAAGGGCGCTGGGAACCCCACTCTTCCCTCTAGGGAGTTGCCGTTCCCTGAGGGGCCTGA
GGAGCCTCCTCCTGAAGCCCCAGCTATTCAAATTACACCTTTTCTTATTAGGACATCAGGAGGTGTTGCC
CCTTAGCTTTCCATTCTAACCTCCTCAATGTAGGGGCCTTGCTGGTGTCGAGGACAGAGCGGAGGCCTCA
AGGGTATGTGCAGTCTAGGTGGCCTTGGTGGAGGGGCAGGATTCTACATTTGGGAGGGGACTTCTTGCTG
AACTCCTTCCAGATGGGTCAAATGGGGTAGACTGAGGGAGTGGTGTGGGAGGTGGTAAAGAACCAAGTAG
GGACTGGATGGATCCTGGGAGTTTACAGGGCTGCCTCGGAGAGGGAGGGTGCTGAGAGAGAGGAGACCAG
GGTGGGCACCAACGCGATCCCTGCAGGCTATGAGTGTAGGATCAGAGAGGGTTAAGGGCCAGAAGCCCCT
GTGTGTTTAGGACTGTGGCACCATGGGTGCATCCCCTGAGAAGCGAAGCTCAGGCAGGAGCAGCAGTGGG
CAAAGAGCCTGGTGGTGCAGGTGCAGGCCCTGTAGAGGAGGCAGTGACAACACCCAGCCCAGGCCGACTC
TCTCTGCCTGTCCCCTGAGTCCCCTGGCACCTCTCCACATCTGCTCAGCCCTCCCAGTTAACCTAGCACT
GGTAGATGGTTGATATGATTTGGATCTGTGTCCCCAAATCTCATGTTGAATTCTAATCCCCAGTGTTGGA
GGTGGGGCCTGGTGGGAGGTGATTGGATCATGGGGGTGAATCCTTCATGAATGGCTTAGCTTTATCCCCT
TAATGCTGTTCTTGTGGTAGTGAGGGAATTCTTGCAATATCTGGTTGTTTAAAAGTGTGTAGCACTTCCC
CCCCAACACTCTTTTGTTCCTGTTCCAACTGTGTGAAGTGCTCACTCCCCCTTTGCCTTCTGCTATGACT
GTTAAGTTTCCTGAGGCCTTCCCAGAAGCTGAGCAGGTGTCAGCATCATGCTTTCTGTATAGCCTGAGGA
ACCATAAGCCAATTAAACCTCTTTTCTTTAAAAATTACCCAGTCTGAGATATTTCTTTTCTTTTCTTTGA
GACAGGGTCTGGCTCTGTCACCCAGGCTGGAGCGTAGCTGCACCTCTGCCTCCAGGGCTCAAGTGATCCT
CCCACCTCAGCCTCACAAGTCACTGGGACCACAGCACGTGCCACCACACCCAGCTAATGTTTGCTTTCTT
TGTAGAGACAGGGTTTCACCATGTTGCACAGGCTGGTCTCAAACTCCTGAGCTCATGCGATTCTCCTGTC
TTGGCCTCCCAAAGTGATGGGATTACAGGTTTGAGCCACTGCACTCATCCTAGGTATTTCTCTATAGCAA
TGCAAGAAAAACTAATACGAAGGTGAATCCATCTGCAGATCCAGACTCCACAGGAAGCCAGCAAGTGTGA
TTGCGGGCTCTGCTGCATTTCAGGGGCGGTTCTATACTTGGCACGTGCATGCTTTCCCCAGCCTAGCATA
TTAGCCTGCTGGGGCTACTATAACAAAATACCACAAACTGAGTGGCTTAATACAACTGAAATGCATTCTC
TCACACTTCTAGTGGCCAAAGTCCAAGATCAAAGTTTTGGCAGGGTTGGCTCCTTCTGGACACTCAGAGG
GAGAGTCTGTCCTGTGTCTCTCCCCTGGCTTCTCCTGGTTGCTGGCAACCCTTGGCATTTTTTGCTTATA
AATGCATCCCTCTTATCTCAGCCTGTACTGTCACGTGACCTTCTTCCCTGTGCATCTGTCTCTTGTCCTC
TTCTTCTAAGGACAGCAGTCATATTGGATTAAGGGCCCAGCCACTCCAGCATGACCTCCTCTTAACTTGA
TGGCATTCAAAGCCATCAGGTTTGGAAACAAGGTCACATTACCAGGGACCGGAGGCTAGGACTTGAAGAT
ACCCTTTGGGAGGAGACACCTGCAACGTTTGGATTGGAGAAGACTCAGTGACCTTCTGGGATGTCGTTTA
CCCTCCCTGGGCTGCAGCTTCCTTGTTTGTAGAGGAAGAGGTTAAATTAAACAATCTCTTCCAGGTC

RNA
>LINC01544
AUUCUCUAGGCGAAGGAGAACAAUAAGAGGCAGGUAAAGCUGGAGCCCGUAACAGGAGGAAACGUGAGCA
UUGAAAACCCGCCCUUUGGAUGAACUGACCACAGCUGGACAACACCAGUGCCAGGCCAGGGUUCCAAGCA
CUGCAGAGAUGAAGCACCUUCUUCCAGGAGUUCCUGGAGUUCUCAGAAGCCAGAGUGGACUUGUUCCUCC
GUUUUAUUUGAUGAGGUUCUAAAUAUAGCUGGGCAAGGCUUCGGAUUUUAGUGGGAGAUGGCCAGAGGCG
AAGCAACUGAUAAAAGUAGCUGAAUAGCUCUCCCUCUCUGAGCUAGGUGCAAGCUCCUGACGUAGGAAGC
GAUCCCUCCUUGCCACAGAUAACCCACGUAGCCAGGUGCUGCCAAGCAGCUUCCACCUGGAGGAGGGCAG
CUGCUCUGCGCAUGACCAGUAGCUGUGGAUGGCAUCGUCGAGGCAUCUGGAGGAAGCUGCAGCCCCACAA
GGCCCCUGACCCCUCUGCCUUUCCCUCUGCUGGCUCCUCUUCCUCCCUCUUUCCCUGCUGUCCCCUGUAG
AGAACUUUCUACCAGUCCACACACCAACCUAGGUGCAGCCCCCCUGCGUGACUCCCUCCCUGACUAUCAU
AACCAUGGCCAGCAGUAUCUCCACAUCUCUGCCCAGAUCCCCCUGGGAAGGCUUAUCCCAGGCCCCAGGU
UUGGGGCAAUGCCCAGUGGCAUCGGAUGGGGUCCCCCUUGAAAGGAAGCACAGAAAGACUGAGCCAUCAA
CGUAGUCCCCUCCAUCACCUGCCACUGUCCCCAAAAUCUCCCACACCGGAAGUGUUUAGAUUGUCUAAGG
ACAAUAUUGUCUAGGGUUUGAGGACCACCCCACAGCAAAAACACUAGAAAGCUAGUUCCAAAAUGCAAAU

CCCAGGCCAAGCCCCUCACCUCCUGAGGCAGCAUCCCUGGGUAUAGAUCUCAGAAAUCUGCAGUUUUAAU
ACAAAGGCCCCCUAAACUUAAAGAAACAGCCUGAAUUCACAUGGGCACACCUUACCUGAGCAGGCAAUAC
AGUGACAAGGGUGGAAACAGAGGGGACGAGACCUGACACUGGGAAGGCCUGACAGAGGGAAGGAAGGAAA
GCUCUCAGGCUAAGGGCGCUGGGAACCCCACUCUUCCCUCUAGGGAGUUGCCGUUCCCUGAGGGGCCUGA
GGAGCCUCCUCCUGAAGCCCCAGCUAUUCAAAUUACACCUUUUCUUAUUAGGACAUCAGGAGGUGUUGCC
CCUUAGCUUUCCAUUCUAACCUCCUCAAUGUAGGGGCCUUGCUGGUGUCGAGGACAGAGCGGAGGCCUCA
AGGGUAUGUGCAGUCUAGGUGGCCUUGGUGGAGGGGCAGGAUUCUACAUUUGGGAGGGGACUUCUUGCUG
AACUCCUUCCAGAUGGGUCAAAUGGGGUAGACUGAGGGAGUGGUGUGGGAGGUGGUAAAGAACCAAGUAG
GGACUGGAUGGAUCCUGGGAGUUUACAGGGCUGCCUCGGAGAGGGAGGGUGCUGAGAGAGAGGAGACCAG
GGUGGGCACCAACGCGAUCCCUGCAGGCUAUGAGUGUAGGAUCAGAGAGGGUUAAGGGCCAGAAGCCCCU
GUGUGUUUAGGACUGUGGCACCAUGGGUGCAUCCCCUGAGAAGCGAAGCUCAGGCAGGAGCAGCAGUGGG
CAAAGAGCCUGGUGGUGCAGGUGCAGGCCCUGUAGAGGAGGCAGUGACAACACCCAGCCCAGGCCGACUC
UCUCUGCCUGUCCCCUGAGUCCCCUGGCACCUCUCCACAUCUGCUCAGCCCUCCCAGUUAACCUAGCACU
GGUAGAUGGUUGAUAUGAUUUGGAUCUGUGUCCCAAAUCUCAUGUUGAAUUCUAAUCCCCAGUGUUGGA
GGUGGGGCCUGGUGGGAGGUGAUUGGAUCAUGGGGGUGAAUCCUUCAUGAAUGGCUUAGCUUUAUCCCCU
UAAUGCUGUUCUUGUGGUAGUGAGGGAAUUCUUGCAAUAUCUGGUUGUUUAAAAGUGUGUAGCACUUCCC
CCCCAACACUCUUUUGUUCCUGUUCCAACUGUGUGAAGUGCUCACUCCCCCUUUGCCUUCUGCUAUGACU
GUUAAGUUUCCUGAGGCCUUCCCAGAAGCUGAGCAGGUGUCAGCAUCAUGCUUUCUGUAUAGCCUGAGGA
ACCAUAAGCCAAUUAAACCUCUUUUCUUUAAAAAUUACCCAGUCUGAGAUAUUUCUUUUCUUUUCUUUGA
GACAGGGUCUGGCUCUGUCACCCAGGCUGGAGCGUAGCUGCACCUCUGCCUCCAGGGCUCAAGUGAUCCU
CCCACCUCAGCCUCACAAGUCACUGGGACCACAGCACGUGCCACCACACCCAGCUAAUGUUUGCUUUCUU
UGUAGAGACAGGGUUUCACCAUGUUGCACAGGCUGGUCUCAAACUCCUGAGCUCAUGCGAUUCUCCUGUC
UUGGCCUCCCAAAGUGAUGGGAUUACAGGUUUGAGCCACUGCACUCAUCCUAGGUAUUUCUCUAUAGCAA
UGCAAGAAAAACUAAUACGAAGGUGAAUCCAUCUGCAGAUCCAGACUCCACAGGAAGCCAGCAAGUGUGA
UUGCGGGCUCUGCUGCAUUUCAGGGGCGGUUCUAUACUUGGCACGUGCAUGCUUUCCCCAGCCUAGCAUA
UUAGCCUGCUGGGGCUACUAUAACAAAAUACCACAAACUGAGUGGCUUAAUACAACUGAAAUGCAUUCUC
UCACACUUCUAGUGGCCAAAGUCCAAGAUCAAAGUUUUGGCAGGGUUGGCUCCUUCUGGACACUCAGAGG
GAGAGUCUGUCCUGUGUCUCUCCCCUGGCUUCUCCUGGUUGCUGGCAACCCUUGGCAUUUUUUGCUUAUA
AAUGCAUCCCUCUUAUCUCAGCCUGUACUGUCACGUGACCUUCUUCCCUGUGCAUCUGUCUCUUGUCCUC
UUCUUCUAAGGACAGCAGUCAUAUUGGAUUAAGGGCCCAGCCACUCCAGCAUGACCUCCUCUUAACUUGA
UGGCAUUCAAAGCCAUCAGGUUUGGAAACAAGGUCACAUUACCAGGGACCGGAGGCUAGGACUUGAAGAU
ACCCUUUGGGAGGAGACACCUGCAACGUUUGGAUUGGAGAAGACUCAGUGACCUUCUGGGAUGUCGUUUA
CCCUCCCUGGGCUGCAGCUUCCUUGUUUGUAGAGGAAGAGGUUAAAUUAAACAAUCUCUUCCAGGUC

>NR_131768.1 Homo sapiens long intergenic non-protein coding RNA 1601 (LINC01601), long non-coding RNA
TCCAAGCACCTCTGCAAATCCACTTCTCACAAACATGATAATTATGATGTTATCTGTGGTCATTTGACTG
AAATCTAAGCCAATGGAATGGGAATAGAGATATCTGTGCCACCTACACCTTCACACCTGGTCAATGAAAA
CTGCCTGCATGTTCTCCCACCTTTCAACTGATGGCAACACTAGAACTGTCTTAGCAGCCTACAGTGTAAA
TCTTGGCCAAAAGAAAGAACCAGAACTTGAAGATGTAATCAAAGACTTTCATTCAGGAGTATCAAGGAAT
TGTGACCCGTCTCTATAGCCTCCAGACCTTGACAAAGAAGACAGAATTGCTTCTCAGTTCCTCAGAGCAG
AACTGTGCATGAAATGAGACCCATCAATAATTTTCTGGAGCCCATCTCACAGCTGTGTGTTCCAACAGCA
GGCTTATACCCCTGGCGTTTTCAACAACATGGCCTAAGGCACAGCCTTATAGGCTTTCTAGAAGAAAAAC
TTCTTTGGGAGCCCTTTCCTGCTCACTTACCAGCCCTGTGATTCATGAAGATAAAGATTATATGAGATAA
TATATGCAAAATACTGTGAAAACTACTAGGGCAAGTATAAGGAATTATCATCTAATTTAGCTGGCTACAG
ATACCCAATGGTTCATCCAGAAGTTTGTTCCAGCTAACAGCAGTTGTGCCTCTTTCATGAGACTTATCAT
CCTTTGGTGGACTAGACTAGACTTATTTGGAACATGGAGACCTAAGAGTTTCAAGACTGAAAGCAAATGA
TTAGGACATTTCCCTAAGGTCTAACCCCAGAAGTCATATAGCTTTGCTGTGCTGTATTCTTTGGGTCAAA
GAAAATCACAAGGTCAGGCCAGGTTCAAGGGAAAGGGAAAATATGCTCCACCTTGTGATGGCAGATTTGG
AAATAATCACATACAAATAAAATAAAAATTAAAACATGAAAAAAA

RNA
>LINC01601
UCCAAGCACCUCUGCAAAUCCACUUCUCACAAACAUGAUAAUUAUGAUGUUAUCUGUGGUCAUUUGACUG
AAAUCUAAGCCAAUGGAAUGGGAAUAGAGAUAUCUGUGCCACCUACACCUUCACACCUGGUCAAUGAAAA
CUGCCUGCAUGUUCUCCCACCUUUCAACUGAUGGCAACACUAGAACUGUCUUAGCAGCCUACAGUGUAAA
UCUUGGCCAAAAGAAAGAACCAGAACUUGAAGAUGUAAUCAAAGACUUUCAUUCAGGAGUAUCAAGGAAU
UGUGACCCGUCUCUAUAGCCUCCAGACCUUGACAAAGAAGACAGAAUUGCUUCUCAGUUCCUCAGAGCAG
AACUGUGCAUGAAAUGAGACCCAUCAAUAAUUUUCUGGAGCCCAUCUCACAGCUGUGUGUUCCAACAGCA
GGCUUAUACCCCUGGCGUUUUCAACAACAUGGCCUAAGGCACAGCCUUAUAGGCUUUCUAGAAGAAAAAC
UUCUUUGGGAGCCCUUUCCUGCUCACUUACCAGCCCUGUGAUUCAUGAAGAUAAAGAUUAUAUGAGAUAA
UAUAUGCAAAAUACUGUGAAAACUACUAGGGCAAGUAUAAGGAAUUAUCAUCUAAUUUAGCUGGCUACAG
AUACCCAAUGGUUCAUCCAGAAGUUUGUUCCAGCUAACAGCAGUUGUGCCUCUUUCAUGAGACUUAUCAU
CCUUUGGUGGACUAGACUAGACUUAUUUGGAACAUGGAGACCUAAGAGUUUCAAGACUGAAAGCAAAUGA
UUAGGACAUUUCCCUAAGGUCUAACCCCAGAAGUCAUAUAGCUUUGCUGUGCUGUAUUCUUUGGGUCAAA
GAAAAUCACAAGGUCAGGCCAGGUUCAAGGGAAAGGGAAAAUAUGCUCCACCUUGUGAUGGCAGAUUUGG
AAAUAAUCACAUACAAAUAAAAUAAAAAUUAAAACAUGAAAAAAA

>NR_040074.1 Homo sapiens long intergenic non-protein coding RNA 1630 (LINC01630), transcript variant 1, long non-coding RNA

AATTGGATATGGGTATTGTAGCTGAAACGTCTTGCGAGCTGAGTCAGGGTCGGGGAAGCTGTGATGGCAA
TGAGAAGTCAACACACTGAGGATGGAGAGAAAGAAGATGAGTCTGGGAGAGAAAGAGAATGATTCTTCTG
GCCGAGGGAAAGCAAGAATATCTGTTCCTGCAACTGCACTGATTCTAGAAACACTCTCTATTCGAACTCT
CATGAGGTGCTGACCTGCTTGGTGAATGCTGGGATCTCTGGAAGAGGGAGGCAGACATCACACACCAGCT
GGCAAGTGGGAGCTGGCATGCAACATGCCCCAATACCACTCCCCTCCTGCCATCCCTATCTCCTAATGGT
GCCTCCTTTTGGCCAAACCCACCTTTGGCTGGAGGGCAACAGAGCCCATTGACGTGGTCCCCTTGCATCT
GCTACCTGGGGAGAAAAGCAGGATGGACACTGAATCTAGAGAAATGAAGAAACCCATCCAGAAGAATACT
GCTTCTTTATGTTTTTGTAGGTGTACAAATCAGGCCTCCCCATGATATTCTAAACTCCTTGAGGAAAGAA
GTGGAGTCCTGGGCTTCTGATGTAGCCTCCACAGTGCTCAGGGGATAGCGACACTGGCACCCTATAGATA
TTTAATTACATGTGGACTGGAAGAGACCAAACAGCTTCCTGGTATTTCAATCAAACCCGGTGCTCTAGAC
CTGTGGCTCACTCTGAGCAAACTATAATGAGTTGAACTTGAATCTGATTCCTGCTAAGAAAGGTTTCATT
TTAATAGGCAGTAAGGCTATGAGCTAACACCAGAATTCATTCGAGTTACTAGACTCACTGAAACTCCTCA
CAGGATCCATCTTTCATGTTTTCTAGATTTTGGTATTCTTATCTAAACTGCAGACCCAGAAACCCTATGG
ACCCTACCTTTCCAATTATATTTCTGGGAAGAGTCATTCTCTGCAGAGCTGAAGCATGTAACAGAAAGTT
CTCAACCTGAAAAATATACCTTCTGCGCCCAAGCCCCACTTTCTGCCACTATTTGGTAATCTCAAGTGGG
TGAGGTGGTCTCTGAACTTCAGCGCTCTCATGGTCAGAGCCACAAGGGGTTACTGGGAGGCTAAAGGGAA
GTGGCAAACGTGGCTGAGATTCAGAAAGAAAAGCAGCAAGTGTAGAGAGGCAAGGACTTTTCCATCTTTT
AAATTCCTTAAAGGAAGCCAAAGTTCATTTTCCAGTCTCCTTCAGATGCCTCTCCAATCCTTCACAGCAG
CGCACACATACCGCAGAAGGAACTCACCATCACAAAAAGATGACAGAGCACTGGGGGCTGGGGTGCCACT
TGTGTTGGCTGAGAGCCTGGATGCATGTGAGGTCAAGCCTGACCTTGGGAAAGCTGCTCTCTGGACATTA
CTTGCTGTGCCACATACACAGCTTGGGTTTCTGAGGAAACAACACAGGCTCTAAGGAGGAACTGGAACGG
GGCCCAGGGTATATCTTTTACAGCCCGTACTTTTGAGGAAAATAGAAGACAAAAGGTTTTTAAAAAACAC
AAAGTGTATACACGTATACACAAAGTTTACAAGTTTGTGTGTTTTAACATCTACTCTCCTTAAGAACAAG
TAGACAGGAAAGATATTACCTGATATGGGTTAAATTGTGTCCCCAAAAACATGTTTTGAAGTGCTAACCT
TTGGTACCTGTGGATATGACCTTACTTGGAAATAGGTTTTTGCAGACGTAACCAAGTTAAGATGAGGTCA
TATTTGATTAGGCTGAACCCTGAATCCAGTATGACTGGGGTCCTTATAT

RNA

>LINC01630

AAUUGGAUAUGGGUAUUGUAGCUGAAACGUCUUGCGAGCUGAGUCAGGGUCGGGGAAGCUGUGAUGGCAA
UGAGAAGUCAACACACUGAGGAUGGAGAGAAAGAAGAUGAGUCUGGGAGAGAAAGAGAAUGAUUCUUCUG
GCCGAGGGAAAGCAAGAAUAUCUGUUCCUGCAACUGCACUGAUUCUAGAAACACUCUCUAUUCGAACUCU
CAUGAGGUGCUGACCUGCUUGGUGAAUGCUGGGAUCUCUGGAAGAGGGAGGCAGACAUCACACACCAGCU
GGCAAGUGGGAGCUGGCAUGCAACAUGCCCCAAUACCACUCCCCUCCUGCCAUCCCUAUCUCCUAAUGGU
GCCUCCUUUUGGCCAAACCCACCUUUGGCUGGAGGGCAACAGAGCCCAUUGACGUGGUCCCCUUGCAUCU
GCUACCUGGGGAGAAAAGCAGGAUGGACACUGAAUCUAGAGAAAUGAAGAAACCCAUCCAGAAGAAUACU
GCUUCUUUAUGUUUUUGUAGGUGUACAAAUCAGGCCUCCCCAUGAUAUUCUAAACUCCUUGAGGAAAGAA
GUGGAGUCCUGGGCUUCUGAUGUAGCCUCCACAGUGCUCAGGGGAUAGCGACACUGGCACCCUAUAGAUA
UUUAAUUACAUGUGGACUGGAAGAGACCAAACAGCUUCCUGGUAUUUCAAUCAAACCCGGUGCUCUAGAC
CUGUGGCUCACUCUGAGCAAACUAUAAUGAGUUGAACUUGAAUCUGAUUCCUGCUAAGAAAGGUUUCAUU
UUAAUAGGCAGUAAGGCUAUGAGCUAACACCAGAAUUCAUUCGAGUUACUAGACUCACUGAAACUCCUCA
CAGGAUCCAUCUUUCAUGUUUUCUAGAUUUUGGUAUUCUUAUCUAAACUGCAGACCCAGAAACCCUAUGG
ACCCUACCUUUCCAAUUAUAUUUCUGGGAAGAGUCAUUCUCUGCAGAGCUGAAGCAUGUAACAGAAAGUU
CUCAACCUGAAAAAUAUACCUUCUGCGCCCAAGCCCCACUUUCUGCCACUAUUUGGUAAUCUCAAGUGGG
UGAGGUGGUCUCUGAACUUCAGCGCUCUCAUGGUCAGAGCCACAAGGGGUUACUGGGAGGCUAAAGGGAA
GUGGCAAACGUGGCUGAGAUUCAGAAAGAAAAGCAGCAAGUGUAGAGAGGCAAGGACUUUUCCAUCUUUU
AAAUUCCUUAAAGGAAGCCAAAGUUCAUUUUCCAGUCUCCUUCAGAUGCCUCUCCAAUCCUUCACAGCAG
CGCACACAUACCGCAGAAGGAACUCACCAUCACAAAAAGAUGACAGAGCACUGGGGGCUGGGGUGCCACU
UGUGUUGGCUGAGAGCCUGGAUGCAUGUGAGGUCAAGCCUGACCUUGGGAAAGCUGCUCUCUGGACAUUA
CUUGCUGUGCCACAUACACAGCUUGGGUUUCUGAGGAAACAACACAGGCUCUAAGGAGGAACUGGAACGG
GGCCCAGGGUAUAUCUUUUACAGCCCGUACUUUUGAGGAAAAUAGAAGACAAAAGGUUUUUAAAAAACAC
AAAGUGUAUACACGUAUACACAAAGUUUACAAGUUUGUGUGUUUUAACAUCUACUCUCCUUAAGAACAAG
UAGACAGGAAAGAUAUUACCUGAUAUGGGUUAAAUUGUGUCCCCAAAAACAUGUUUUGAAGUGCUAACCU
UUGGUACCUGUGGAUAUGACCUUACUUGGAAAUAGGUUUUUGCAGACGUAACCAAGUUAAGAUGAGGUCA
UAUUUGAUUAGGCUGAACCCUGAAUCCAGUAUGACUGGGGUCCUUAUAU

>NR_110763.1 Homo sapiens long intergenic non-protein coding RNA 1882 (LINC01882), long non-coding RNA
GCGACCTTGAAGCGGCATCCGAGGAGATGTGGCCACGGGGCAGGCGACCGACACCAGCGAGTCCAGAGGG
CCAGCGTGTGCACCACTGTGTGTCTCCAGAGACTTCAGGAAGCAGCCACCACGCCCGAGGAATGCAGGAA
GATGGACACACGGCTGGGGAAGTACAATGAAAGGCCAAGTAGGCAGCCTGTTCTCCTCAGATCAGTCCCC
CACGAACACTCATTCCCGAGGACTCATCCAATACTAATAAGAGAATGCTCTTGTTTTTGAAGAATTTTCT
GAAAGCCATCCTGACAAATTAAGTAGAGTATGCTGAAGATAGTCAGACTTTGTTTTTAAGAATTGAATAT
TCTGGAAGAGGCTCTTCAGTCCAATCTTTAGTTCTCTCCACAGAGCAAACGAAGTGAAGTGCTGAAGGCC
TGGAGCCCGAGCTGTTCCCACACGGACTCCAGGACAGTTAAGGCAGGGTTGCCTTAACTAAATCTCTGAC
AACTGTTTCTTCTGTCTTTCTCCTAAAAATGGAATGCAGGCCATCTATTCTAGGGAAATAAAGGATTCTA
GTTATGTGAATCCA

RNA
>LINC01882
GCGACCUUGAAGCGGCAUCCGAGGAGAUGUGGCCACGGGGCAGGCGACCGACACCAGCGAGUCCAGAGGG
CCAGCGUGUGCACCACUGUGUGUCUCCAGAGACUUCAGGAAGCAGCCACCACGCCCGAGGAAUGCAGGAA
GAUGGACACACGGCUGGGGAAGUACAAUGAAAGGCCAAGUAGGCAGCCUGUUCUCCUCAGAUCAGUCCCC
CACGAACACUCAUUCCCGAGGACUCAUCCAAUACUAAUAAGAGAAUGCUCUUGUUUUUGAAGAAUUUUCU
GAAAGCCAUCCUGACAAAUUAAGUAGAGUAUGCUGAAGAUAGUCAGACUUUGUUUUUAAGAAUUGAAUAU
UCUGGAAGAGGCUCUUCAGUCCAAUCUUUAGUUCUCUCCACAGAGCAAACGAAGUGAAGUGCUGAAGGCC
UGGAGCCCGAGCUGUUCCCACACGGACUCCAGGACAGUUAAGGCAGGGUUGCCUUAACUAAAUCUCUGAC
AACUGUUUCUUCUGUCUUUCUCCUAAAAAUGGAAUGCAGGCCAUCUAUUCUAGGGAAAUAAAGGAUUCUA
GUUAUGUGAAUCCA

>NR_146509.1 Homo sapiens long intergenic non-protein coding RNA 1887 (LINC01887), long non-coding RNA
GAAAACTAAGTGGCTTCCCTTGAGGCAGCCCGAGGGCTCCACTGGACCCAGAACTCTGAGTTCTGATGCT
GCCTATCATTTGCTTTTCCTCATGAGCTCCTGGACCACCTCATATCACCAGCACCCTCTACCCAAGGTCC
TGGAGCCAACACTGGAGTAAAAGAGCACAAGAGAACTCCTTAGAGGTGGAAGGATACCCTCTGAGGTCAA
ATCATTGGGCTTGTGGCCATGAGACCACAGACTTTGCCTGGCTTCCAGTGACTGTCCGAGATGACCTGTG
GAGTGCACCGCCTGTACTTGGACATCACTGTCTTTTCGGATCCATACCCGTCATCCTTGGAATTTCCCCT
GCATCCAAACCTGTTCCTGATACAGGTGTGAGTAATGAATAACCCTAAGGAATATCTTGGATGTCTTCAC
GGGGCTTATGACTTATTCTGAGGTTTACAAAATAGAGGCCCCCAGCTGAGCAAAGAGAGGGCTGCAGCGC
CGAGGGAACAGCTGCCGGACTCACCGGAGAGGCCTCCTGGAGCACGCCGGGACCCCCTCCACCCCCTGGC
TTTGGCTGCATCAATGGCTAGTTTGCCTGAACTATATCTGAGCCACTGAGAGTATTTATTAAGCAGAGAA
TAATTTTGAGTTTCACTTTTATAACTTTTGTTTCAAAAGTTGTTTTGGATAATTTAATGAAATGACTGTA
AACCAGAATTCCCCTTTTCATTATTTCTTTGTGTCAATATAAAAAGAGTTGATGATTTTAAAAGCCAGTG
AGATGTCCCTGTCTGTGGATGTCAGCGGGAGGGTGGGGTCTGACGCCTTTCTCTGGTGGCTGTCCAGGAA
GATTGCCCCAGCTTGCACTCCCTTTTGGCGGGTTCCAGAGCCTTCCATTTGAAAGGAGCATCTCTCACCA
CTCTGCCCCACCCAGCACCAGCTCTTCTGGAACCCACTGACTTCCTAAACACTTTCTTCCTCCTTCACCT
CATTGAATTCAAGACTTTAGAAAACAGCCAAAAACTCTAGAGGTGGGGGTGGGTTCGATCTGGAAATCCC
TGCTGTAATCTTTTTAAGGGAATCCACTGGAAATTATTAATTTATGTTTTAAATAAAACCATTCCTTACT
TAACAGAATATTAAGCCACTCATTTTTTTAACAGAACTTTCGAGATATAATTAAAATGCCATAAATTTCA
CCCCCACACAGTGTACAATTCCAAGGTTTCCAATATAGTCACAGAAGTCTCTGCAACAATTACCACAATT
TTAGACATGAAGACCATTTGTTGGCAGCCCTACCTGCCTCTATGTGTATGTATATAATTTTAATATTGGA
TTGGATACTATAGTAATATTGTACTGTTACCTATTTGTGTTTTTATATTGCTGTTTTATTTTATTCAGAT
GAATAATAATTATCTCCATATAGTCATACATTTATATAGGGTTAAACACACCTTTTTACAATTCAATAAA
TTAAATTGGTCAGGTATGGTGGCTCACGCCTGTAATCCCAGCACTTTGGGAGGCCAAGGCGGGTGGATCA
CCTGAGGTTGGGAGTTTGAGACCAGCGTGATCAACATGGAGAAACCCTGTCTGTACTAAAAATACAAAAA
ATTAGCCAGGCATGGTGGCACATGCCTGTAATCCCAGCTACTTGGGAGGCTGAGGCAGGAGAATCACTCG
AACCAAGGAGGCGGACATTGCGGTGAGCCAGGATCGCACCATTGCACTCCAGCCTGGGCAACAAGAGCAA
AACTCCATCTCAAGAAGAAAAAAAAAAAAAAAAGTAAATTGTTTTTAATTACATAAGAAACACGTGTTATT
GTTATAGAAATTCACATAGTGCTTCGGTCTATAGAATAAATGTATCCCCACCACCTCCCAGCCCACCCAC
TCGTCTGCATTCACCTACACACAGTTAAACATGCACATATGTTTCCGTGACACGATCTTGCTTTGCATAT
TGTTCACTGACTGGCTTTTCCATATAAGATTTAATTTGGTGATTATTGCTTATCAGTACCTATAGGTCTC
TCTCATTATTATTTATAATTACATCATTATTCCATAGGGTATTTTACCACAATTTGGTCACTTTTTCACT
GAGGAATTTTTATGTCAGTTTCAGTTATTTGTTATTGTTACCAATGCTGTACTGGGCATCCCTGTATATC
CACCTTTTTGTTGCATTAGTTAAGTGGTTTTTTCCTTTTATGATTGATTTCTAAAAATAAAATACTGCAG
CAAA

RNA
>LINC01887
GAAAACUAAGUGGCUUCCCUUGAGGCAGCCCGAGGGCUCCACUGGACCCAGAACUCUGAGUUCUGAUGCU
GCCUAUCAUUUGCUUUUCCUCAUGAGCUCCUGGACCACCUCAUAUCACCAGCACCCUCUACCCAAGGUCC
UGGAGCCAACACUGGAGUAAAAGAGCACAAGAGAACUCCUUAGAGGUGGAAGGAUACCCUCUGAGGUCAA
AUCAUUGGGCUUGUGGCCAUGAGACCACAGACUUUGCCUGGCUUCCAGUGACUGUCCGAGAUGACCUGUG
GAGUGCACCGCCUGUACUUGGACAUCACUGUCUUUUCGGAUCCAUACCCGUCAUCCUUGGAAUUUCCCCU
GCAUCCAAACCUGUUCCUGAUACAGGUGUGAGUAAUGAAUAACCCUAAGGAAUAUCUUGGAUGUCUUCAC
GGGGCUUAUGACUUAUUCUGAGGUUUACAAAAUAGAGGCCCCCAGCUGAGCAAAGAGAGGGCUGCAGCGC
CGAGGGAACAGCUGCCGGACUCACCGGAGAGGCCUCCUGGAGCACGCCGGGACCCCCUCCACCCCCUGGC
UUUGGCUGCAUCAAUGGCUAGUUUGCCUGAACUAUAUCUGAGCCACUGAGAGUAUUUAUUAAGCAGAGAA
UAAUUUUGAGUUUCACUUUUAUAACUUUUGUUUCAAAAGUUGUUUUGGAUAAUUUAAUGAAAUGACUGUA
AACCAGAAUUCCCCUUUUCAUUAUUUCUUUGUGUCAAUAUAAAAAGAGUUGAUGAUUUUAAAAGCCAGUG
AGAUGUCCCUGUCUGUGGAUGUCAGCGGGAGGGUGGGGUCUGACGCCUUUCUCUGGUGGCUGUCCAGGAA
GAUUGCCCCAGCUUGCACUCCCUUUUGGCGGGUUCCAGAGCCUUCCAUUUGAAAGGAGCAUCUCUCACCA
CUCUGCCCCACCCAGCACCAGCUCUUCUGGAACCCACUGACUUCCUAAACACUUUCUUCCUCCUUCACCU
CAUUGAAUUCAAGACUUUAGAAAACAGCCAAAAACUCUAGAGGUGGGGGUGGGUUCGAUCUGGAAAUCCC
UGCUGUAAUCUUUUUAAGGGAAUCCACUGGAAAUUAUUAAUUUAUGUUUUAAAUAAAACCAUUCCUUACU
UAACAGAAUAUUAAGCCACUCAUUUUUUUAACAGAACUUUCGAGAUAUAAUUAAAAUGCCAUAAAUUUCA
CCCCCACACAGUGUACAAUUCCAAGGUUUCCAAUAUAGUCACAGAAGUCUCUGCAACAAUUACCACAAUU
UUAGACAUGAAGACCAUUUGUUGGCAGCCCUACCUGCCUCUAUGUGUAUGUAUAUAAUUUUAAUAUUGGA
UUGGAUACUAUAGUAAUAUUGUACUGUUACCUAUUUGUGUUUUUAUAUUGCUGUUUUAUUUUAUUCAGAU
GAAUAAUAAUUAUCUCCAUAUAGUCAUACAUUUAUAUAGGGUUAAACACACCUUUUUACAAUUCAAUAAA
UUAAAUUGGUCAGGUAUGGUGGCUCACGCCUGUAAUCCCAGCACUUUGGGAGGCCAAGGCGGGUGGAUCA
CCUGAGGUUGGGAGUUUGAGACCAGCGUGAUCAACAUGGAGAAACCCUGUCUGUACUAAAAAUACAAAAA
AUUAGCCAGGCAUGGUGGCACAUGCCUGUAAUCCCAGCUACUUGGGAGGCUGAGGCAGGAGAAUCACUCG
AACCAAGGAGGCGGACAUUGCGGUGAGCCAGGAUCGCACCAUUGCACUCCAGCCUGGGCAACAAGAGCAA
AACUCCAUCUCAAGAAGAAAAAAAAAAAAAAAAGUAAAUUGUUUUUAAUUACAUAAGAAACACGUGUUAUU

GUUAUAGAAAUUCACAUAGUGCUUCGGUCUAUAGAAUAAAUGUAUCCCCACCACCUCCCAGCCCACCCAC
UCGUCUGCAUUCACCUACACACAGUUAAACAUGCACAUAUGUUUCCGUGACACGAUCUUGCUUUGCAUAU
UGUUCACUGACUGGCUUUUCCAUAUAAGAUUUAAUUUGGUGAUUAUUGCUUAUCAGUACCUAUAGGUCUC
UCUCAUUAUUAUUUAUAAUUACAUCAUUAUUCCAUAGGGUAUUUUACCACAAUUUGGUCACUUUUUCACU
GAGGAAUUUUUAUGUCAGUUUCAGUUAUUUGUUAUUGUUACCAAUGCUGUACUGGGCAUCCCUGUAUAUC
CACCUUUUUGUUGCAUUAGUUAAGUGGUUUUUUCCUUUUAUGAUUGAUUUCUAAAAAUAAAAUACUGCAG
CAAA

>NR_146900.1 Homo sapiens long intergenic non-protein coding RNA 1892 (LINC01892), long non-coding RNA

AAGAAGTTGGCCTTAGCCAGAATGGAGATACCTGATCATCAGAGGCTGGAGGAAAGCAGGTTCCTCAAAA
AACAAACAACAACAAAAAAACAACAAAACATGAAGAAGCCAAATCTGGAGGATGAGTTTTCCAAATTAAA
CACTGAATATTGTTGTAGAGTAAAATGATACTGTTTGTAAAGAGATGTTTTTACTAGACATCCTCTTCCT
AGCCACCTATTAAGGAATAACTAGAATATATATTAACAGCAAGACATCGGAAATTATCTTTGCACCTGAC
AAAATGTTAAGATTCAGAATTTGGCTGGATGCTCCTAAAGAGCTACTGAAATAGACTCAATGGCAGCTAC
GATGCCCAACAGAAGCAGCGATGCCTGGTGCACATAGCAGGTTGCTAGCAGGAGTCATCTGGGGTGATTC
ACCTGTCTTGTTGGTGAGGATATGGACATCATCTGGGGACTCTCAGAAATCCCTGAAGTGATATAGAGTC
CCACATGAGCTTGCCTGTGGTGTTTTACAAACCAATAACGTAGTGTTCATAAACCAAAGTCACTTGGAGC
TTCAGAATTGTAAAATATTCTCAAGAAGGTTTTCAGGCCGGCTTATACCTGGAAAAACTTACAGATCTAC
TTGAAGCCTCTCTAAGCTCCCGGACTAGTAAATTTCCAAAAAATCAGCTTTCAAGATGATTTTCCGTTGG
GTCTGATGAAGATTCTACCCTTCACTAGACTCTACCCAGGCTCCTCTGAGCCCTCTTCTCAACTAGATCT
CAACTGTGGTCTATAAAGGCTTAACACTAACATAGTTTCCAACAGCTCAACCCCACATCCCAAAGGTGTT
CTTCACTAGCCTTAAAGTGCTGGCCTGAGAAAACTCAAGACTGCCAAATGAATCTGCGTTTTTTCCAGGC
ATCACCTGAAGATAAGGCCCCTGTCTCCTAGTCTCTGTGGGAAGGCGAGAACCTAGCTTGGATAAGCGCC
AGTCAAAAAACCCACATGGTTTCCCATGGACCAACTCCACTTTACCACTTTTGGGAATTTTTCACTTTCC
TAACTGAGCCCCTACTTTCCCCTCTCCCTAATTCCTTCTTTTATCCTTAAAATGCCCAGTCACTTCTGTA
CAACTCGAAGTTGAATTTTGTTCACACTGGACTCTTTTCCCTATTGAAATAGTTATCACTGATTAAAATC
TGTCCTTACCACTTTAA

RNA

>LINC01892

AAGAAGUUGGCCUUAGCCAGAAUGGAGAUACCUGAUCAUCAGAGGCUGGAGGAAAGCAGGUUCCUCAAAA
AACAAACAACAACAAAAAAACAACAAAACAUGAAGAAGCCAAAUCUGGAGGAUGAGUUUUCCAAAUUAAA
CACUGAAUAUUGUUGUAGAGUAAAAUGAUACUGUUUGUAAAGAGAUGUUUUUACUAGACAUCCUCUUCCU
AGCCACCUAUUAAGGAAUAACUAGAAUAUAUAUUAACAGCAAGACAUCGGAAAUUAUCUUUGCACCUGAC
AAAAUGUUAAGAUUCAGAAUUUGGCUGGAUGCUCCUAAAGAGCUACUGAAAUAGACUCAAUGGCAGCUAC
GAUGCCCAACAGAAGCAGCGAUGCCUGGUGCACAUAGCAGGUUGCUAGCAGGAGUCAUCUGGGGUGAUUC
ACCUGUCUUGUUGGUGAGGAUAUGGACAUCAUCUGGGGACUCUCAGAAAUCCCUGAAGUGAUAUAGAGUC
CCACAUGAGCUUGCCUGUGGUGUUUUACAAACCAAUAACGUAGUGUUCAUAAACCAAAGUCACUUGGAGC
UUCAGAAUUGUAAAAUAUUCUCAAGAAGGUUUUCAGGCCGGCUUAUACCUGGAAAAACUUACAGAUCUAC
UUGAAGCCUCUCUAAGCUCCCGGACUAGUAAAUUUCCAAAAAAUCAGCUUUCAAGAUGAUUUUCCGUUGG
GUCUGAUGAAGAUUCUACCCUUCACUAGACUCUACCCAGGCUCCUCUGAGCCCUCUUCUCAACUAGAUCU
CAACUGUGGUCUAUAAAGGCUUAACACUAACAUAGUUUCCAACAGCUCAACCCCACAUCCCAAAGGUGUU
CUUCACUAGCCUUAAAGUGCUGGCCUGAGAAAACUCAAGACUGCCAAAUGAAUCUGCGUUUUUUCCAGGC
AUCACCUGAAGAUAAGGCCCCUGUCUCCUAGUCUCUGUGGGAAGGCGAGAACCUAGCUUGGAUAAGCGCC
AGUCAAAAAACCCACAUGGUUUCCCAUGGACCAACUCCACUUUACCACUUUUGGGAAUUUUUCACUUUCC
UAACUGAGCCCCUACUUUCCCCUCUCCCUAAUUCCUUCUUUUAUCCUUAAAAUGCCCAGUCACUUCUGUA
CAACUCGAAGUUGAAUUUUGUUCACACUGGACUCUUUUCCCUAUUGAAAUAGUUAUCACUGAUUAAAAUC
UGUCCUUACCACUUUAA

>NR_146903.1 Homo sapiens long intergenic non-protein coding RNA 1894 (LINC01894), long non-coding RNA

ATTCTCAAGTGAGCCACAGGAAGAGTCCTGTTTTGAGGGCAAGGCTTTTCTTGAAAACTACACTCTCTAT
GCAGCTCATTCCTGAAGAAGGGCTTTGGCATGTGGAGATTATGCCCTCTTGCTAGGGTACTGGAAGGGAT
CCATGTGGTATTGTCCCAGCAATAACTCTGCCACAGAGATATGCGATCTCCAGCAGCTGTCGGCAGGCTT
CACTCCCATTCCATCCTCCTTCCTGCATCCAGTAGTCTCCCAGCCCCGCCTCACCCTCCCACTGCTCCAG
AAGAATGACAGAACTCAGAGCTGCAAGAGAAAGAAGACTGAATTCAGTCTCTCTTGCAGAAAAATTGTTT
TCCGTCAAGTTGATAATAATGATCACACTGAGAATGAATGACTCAAGATGAATATCCAAAGATATATCAA
AACCTGGTCAGCAGTCGTGGGGAAAGGAAGAAAGCCTGAGTTGAGTTTATGAAAGTTTTCCACTAAAGTC
AGCAACAGCTTAAATTCAAACCAGGCACAAGACGCTTGAATCTCCATATGAGTTGTTCATCAAAGGGCTG
AAAGAAAAGAAATCTGAGCCCGAGCACGGTCTGGCCTAAAAGCTTTCAAGTGACTCCTTTCTGTTGCAAA
TGAAAGAAGTCCTGCTACTCTAAAGAATAACTAAACCAGACTGGAGTCACTGAAACTGATGGAAGAACAT
CTCAGGCATGAGTGCCATGGTGACGTCATCAGCTCCGTTCACAACCACTCACCATGAGGCCATTGCTCCT
GTGGAGGACACTGGCTTAGGACTCATGAAGTAGTACCTCAGGAAGGTGTCTCTGTAAGTGTTCCTTCCAC
CAGCTCCAAGACACAATGAGATGGAGAGAACTGGAACAATTCCCTATGATTGCAGCTGGATATTTTCACA
TGAACTATCTAGTAATGAAGGCAATGGCATGTGGTGTGGCAAAGACTGGCCCCAGATTACATTTCCAGTC
CTTTTGTACTGAGGAGGGGCTTTGGGACCAAGACAGTAGGAGTGAGGGTACAGGTTCCACATTCTATTTC
TCTCTTAGTTTTCTGTGGTTATCTTGGTGACTGCATGGTAAAGATGGAGGTGACAAAAGATGGAAGGAGC
CTGGATCCCTGAATGACTTCATGGTGCAGAGCCTTACACTACCCCCAACTCATACTAAAACAGGGGTGGG
AGGAACAATAGACCATTATTCTGTTAAGGTGCTGAAATTGTAGAGCTGTTAATTTGAACAGTTAGAGTTT
CTTATACTGATACAGTATCCTTACCCTGGATTGTTGAAATAATTAGTCCAAATAGTTTCTTTGCCTAAAA
CTGTGGACAGAGCCTAAGCAATATTGAATCCAAGACAAGGTATATTTATCATCAAATGCTACTCACTATA
CAATACAGATGAATGTGGCACTTACTTCCCGTGTTGTTACCCAGCCTGCTTCTAGTATTCTTTACACAAA
ATAACATTCATGAGCAACCTGGGCACA

RNA

>LINC01894

AUUCUCAAGUGAGCCACAGGAAGAGUCCUGUUUUGAGGGCAAGGCUUUUCUUGAAAACUACACUCUCUAU
GCAGCUCAUUCCUGAAGAAGGGCUUUGGCAUGUGGAGAUUAUGCCCUCUUGCUAGGGUACUGGAAGGGAU
CCAUGUGGUAUUGUCCCAGCAAUAACUCUGCCACAGAGAUAUGCGAUCUCCAGCAGCUGUCGGCAGGCUU
CACUCCCAUUCCAUCCUCCUUCCUGCAUCCAGUAGUCUCCCAGCCCCGCCUCACCCUCCCACUGCUCCAG
AAGAAUGACAGAACUCAGAGCUGCAAGAGAAAGAAGACUGAAUUCAGUCUCUCUUGCAGAAAAAUUGUUU
UCCGUCAAGUUGAUAAUAAUGAUCACACUGAGAAUGAAUGACUCAAGAUGAAUAUCCAAAGAUAUAUCAA
AACCUGGUCAGCAGUCGUGGGGAAAGGAAGAAAGCCUGAGUUGAGUUUAUGAAAGUUUUCCACUAAAGUC
AGCAACAGCUUAAAUUCAAACCAGGCACAAGACGCUUGAAUCUCCAUAUGAGUUGUUCAUCAAAGGGCUG
AAAGAAAAGAAAUCUGAGCCCGAGCACGGUCUGGCCUAAAAGCUUUCAAGUGACUCCUUUCUGUUGCAAA
UGAAAGAAGUCCUGCUACUCUAAAGAAUAACUAAACCAGACUGGAGUCACUGAAACUGAUGGAAGAACAU
CUCAGGCAUGAGUGCCAUGGUGACGUCAUCAGCUCCGUUCACAACCACUCACCAUGAGGCCAUUGCUCCU
GUGGAGGACACUGGCUUAGGACUCAUGAAGUAGUACCUCAGGAAGGUGUCUCUGUAAGUGUUCCUUCCAC
CAGCUCCAAGACACAAUGAGAUGGAGAGAACUGGAACAAUUCCCUAUGAUUGCAGCUGGAUAUUUUCACA
UGAACUAUCUAGUAAUGAAGGCAAUGGCAUGUGGUGUGGCAAAGACUGGCCCCAGAUUACAUUUCCAGUC
CUUUUGUACUGAGGAGGGGCUUUGGGACCAAGACAGUAGGAGUGAGGGUACAGGUUCCACAUUCUAUUUC
UCUCUUAGUUUUCUGUGGUUAUCUUGGUGACUGCAUGGUAAAGAUGGAGGUGACAAAAGAUGGAAGGAGC
CUGGAUCCCUGAAUGACUUCAUGGUGCAGAGCCUUACACUACCCCCAACUCAUACUAAAACAGGGGUGGG
AGGAACAAUAGACCAUUAUUCUGUUAAGGUGCUGAAAUUGUAGAGCUGUUAAUUUGAACAGUUAGAGUUU
CUUAUACUGAUACAGUAUCCUUACCCUGGAUUGUUGAAAUAAUUAGUCCAAAUAGUUUCUUUGCCUAAAA
CUGUGGACAGAGCCUAAGCAAUAUUGAAUCCAAGACAAGGUAUAUUUAUCAUCAAAUGCUACUCACUAUA
CAAUACAGAUGAAUGUGGCACUUACUUCCCGUGUUGUUACCCAGCCUGCUUCUAGUAUUCUUUACACAAA
AUAACAUUCAUGAGCAACCUGGGCACA

>NR_146506.1 Homo sapiens long intergenic non-protein coding RNA 1895 (LINC01895), long non-coding RNA

CATGCGCGGAGCTCACTAGTGGCAGAGCCCACGACACCCGCTCCTCTCCAGCTCTCTCTTTAGGGACAGA
GAGGAATCTACTTACCAAGATGGCAGGAGCAAGTGGTTATCAGAGCCTGCTCTCTAGAAATACAATTAAA
CAAGCAGCCCCTGTCTCTTTTTCTCCACTTTCAGTCTTGGGGAAGGGATTGGAATATGTGAGCAGGAGAG
AAGGGTCATGTTGGAGCAGCCAGGACTCAGAGCCTTTCCGCAGACTGAGTCTGGATCCTTTCCTTCAGCC
TCCAGGAATACTCCCGGCTCCACACCCAGAATGGTAATCCAGGCAAGGGCTCTGGTGTGTGGCCACACTG
CCACCTATTACCTGCGGGTCCAAATGCAGTGACTCAACTTCTCTGTGCCTCTCTTTCCACATGTATAAAA
TGAGGATAATGTTGCTATTGTAAGAATTGCATCAATTAATAAACAGCACTCAGTGAAAAAA

RNA

>LINC01895

CAUGCGCGGAGCUCACUAGUGGCAGAGCCCACGACACCCGCUCCUCUCCAGCUCUCUCUUUAGGGACAGA
GAGGAAUCUACUUACCAAGAUGGCAGGAGCAAGUGGUUAUCAGAGCCUGCUCUCUAGAAAUACAAUUAAA
CAAGCAGCCCCUGUCUCUUUUUCUCCACUUUCAGUCUUGGGGAAGGGAUUGGAAUAUGUGAGCAGGAGAG
AAGGGUCAUGUUGGAGCAGCCAGGACUCAGAGCCUUUCCGCAGACUGAGUCUGGAUCCUUUCCUUCAGCC
UCCAGGAAUACUCCCGGCUCCACACCCAGAAUGGUAAUCCAGGCAAGGGCUCUGGUGUGUGGCCACACUG
CCACCUAUUACCUGCGGGUCCAAAUGCAGUGACUCAACUUCUCUGUGCCUCUCUUUCCACAUGUAUAAAA
UGAGGAUAAUGUUGCUAUUGUAAGAAUUGCAUCAAUUAAUAAACAGCACUCAGUGAAAAAA

>XR_007066389.1 PREDICTED: Homo sapiens long intergenic non-protein coding RNA 1897 (LINC01897), ncRNA
CAGTTGACTTCACCACAGGCCCAGGTCTTGTATTGAGGACAAGGAAATACTGCCCCATAATGCTGTTGAG
AATATTTAAGAGCAGTCAGGAAACCCTTTGTAGCTTGGCTTTATTGCAGCAAGTGTCAAAGAGGAGGACC
TGAACTTCCAAGGCCAACTTGACTGGAAACCCTCTAGTCTTTAATCCATAGAAACCTGACTTCTGTGCCC
CCCATGCCTAGAAAACTACTTTTAAAAAGAACATTAGTGGTCACCACGTTGGTAAAGTATTAATAAATAA
ACATGTTTTAGTTCTAA

RNA
>LINC01897
CAGUUGACUUCACCACAGGCCCAGGUCUUGUAUUGAGGACAAGGAAAUACUGCCCCAUAAUGCUGUUGAG
AAUAUUUAAGAGCAGUCAGGAAACCCUUUGUAGCUUGGCUUUAUUGCAGCAAGUGUCAAAGAGGAGGACC
UGAACUUCCAAGGCCAACUUGACUGGAAACCCUCUAGUCUUUAAUCCAUAGAAACCUGACUUCUGUGCCC
CCCAUGCCUAGAAAACUACUUUUAAAAAGAACAUUAGUGGUCACCACGUUGGUAAAGUAUUAAUAAAUAA
ACAUGUUUUAGUUCUAA

>NR_126324.1 Homo sapiens long intergenic non-protein coding RNA 1899 (LINC01899), long non-coding RNA
GACTCCTTCATCATCAGGATGCCAGATAATATTTTAAAAGCTGACTCCACCAGAGACCCGATTCTTGAAC
TTTTAATGCACATTTGGAAATCCCGTGTTTAGTTGGCTTAGATTATAAACTGCAGGAGAACAAGAAGTGT
ATCAATTATGTTCCAGTTTCTAGGATGTACTCTTCTAGTGGATCCACAAGCAAAAGGGAAGAGGGGAGTG
TGAAGGCAGAGAAAATAAAGCACAGCCTATGAAATTATAGCTTGCAGTTTTGCCAGAATGATGCTCTATT
AATGCTCAGTGCCTCTTAACTTCTCACTTAAAACCCAATTGGGAAAAATCACCAACTAATAGAGGCTATT
CCGTATACTTACTAGAATATATGAGTTTTACAAATTGGAATGATAGGCATTGATATTGACACATCTGGAG
AAGGAGTGATCAAAGAGAAGTGGCCCTGGAGCTCTCCCCATGAAAAGATTAAATTTTTAAGAAGATGCTG
CTCACCTAAAACTTATCAATAAAGTCAACTATAGTTTCCAATTGGTAAATCATTGTATGAAAAAACAATA
AAACTATGAAATAAA

RNA
>LINC01899
GACUCCUUCAUCAUCAGGAUGCCAGAUAAUAUUUUAAAAGCUGACUCCACCAGAGACCCGAUUCUUGAAC
UUUUAAUGCACAUUUGGAAAUCCCGUGUUUAGUUGGCUUAGAUUAUAAACUGCAGGAGAACAAGAAGUGU
AUCAAUUAUGUUCCAGUUUCUAGGAUGUACUCUUCUAGUGGAUCCACAAGCAAAAGGGAAGAGGGGAGUG
UGAAGGCAGAGAAAAUAAAGCACAGCCUAUGAAAUUAUAGCUUGCAGUUUUGCCAGAAUGAUGCUCUAUU
AAUGCUCAGUGCCUCUUAACUUCUCACUUAAAACCCAAUUGGGAAAAAUCACCAACUAAUAGAGGCUAUU
CCGUAUACUUACUAGAAUAUAUGAGUUUUACAAAUUGGAAUGAUAGGCAUUGAUAUUGACACAUCUGGAG
AAGGAGUGAUCAAAGAGAAGUGGCCCUGGAGCUCUCCCCAUGAAAAGAUUAAAUUUUUAAGAAGAUGCUG
CUCACCUAAAACUUAUCAAUAAAGUCAACUAUAGUUUCCAAUUGGUAAAUCAUUGUAUGAAAAAACAAUA
AAACUAUGAAAUAAA

>XR_001753529.2 PREDICTED: Homo sapiens long intergenic non-protein coding RNA 1900 (LINC01900), transcript variant X1, ncRNA
GGCCTACTCATCAGAGCCTCTGGAAGTGGATTGGTATCCCCTCTGCTCCCAGGAATCCAGTAAAAGTTTG
CAGAGGATGCAGCAGGTGAGTCCTGTAGACATCAGAGGGGCTTCGACGCTGCACAGCTGCCACTCAGAGG
GGACACTGCTGGCCTCGATCACATCATGGAGACCACAAGGATGCTGGGAATGCACTTCCCATTGGCCGAG
CTGAGGATATGAGCAGGCCATGGGCTGGATGGCTCACGCGTTCAGAGGAGGGACAGCGCTTGTTGCAGAG
AATGAGATTTAGTGTGATGTGTATAAAGGTGTTTCATGGCTAAAGACCAAACAGAGACTAGTGTCTCCCA
CTCAGGAAGAATAATCACCATGCTAATGGATTAAAATTCTGTTACATAGAA

RNA
>LINC01900
GGCCUACUCAUCAGAGCCUCUGGAAGUGGAUUGGUAUCCCCUCUGCUCCCAGGAAUCCAGUAAAAGUUUG
CAGAGGAUGCAGCAGGUGAGUCCUGUAGACAUCAGAGGGGCUUCGACGCUGCACAGCUGCCACUCAGAGG
GGACACUGCUGGCCUCGAUCACAUCAUGGAGACCACAAGGAUGCUGGGAAUGCACUUCCCAUUGGCCGAG
CUGAGGAUAUGAGCAGGCCAUGGGCUGGAUGGCUCACGCGUUCAGAGGAGGGACAGCGCUUGUUGCAGAG
AAUGAGAUUUAGUGUGAUGUGUAUAAAGGUGUUUCAUGGCUAAAGACCAAACAGAGACUAGUGUCUCCCA
CUCAGGAAGAAUAAUCACCAUGCUAAUGGAUUAAAAUUCUGUUACAUAGAA

>NR_187474.1 Homo sapiens long intergenic non-protein coding RNA 1901 (LINC01901), long non-coding RNA
ACCTAATCAAGCCTGGGCAATGGCGGGCGCCCCTCCCCCAGCCTTGCTGCCGCCTTGCAGTTTGATCTCAGACTGCT
GTGCTAGCAATCAGCGAGACTCCGTGGGTGTAGGACCCTCTGAGCCAGGTGTGGGATATAATCTCGTGGTGCGCCGT
TTTTTAAGCCCGTCGGAAAAGCGCAGTATTCAGGTATCACCAGCTGTTTATTTCTTCAGACATAGTTAAAATCTAAA
AGAAAAAAGAAAAAAAAGTCCAACATTCAGAATCCCCAAGATGCATACGTAAATCCTAATAGAAAATGCTCAGTTCA
AGAAGTTCCATTGAGGTGAAGCAATGATGCAAGGCTTTTGGCAACCGTTCCCCAGCTCCCAGCCACACATCGCTCCA
CTTACCTTGAGTGACAGCCTCCAAAGAAAAGTTTCCACCGCGCCAGAGTCTTGACCCTGAGTGGCATCGGCAGGGTC
TCCTGCGGCAGACCAGGGGTGTTGCGGGCTGCGCTGGGTGGGGGACCGCGCGCTGCACGTGCAGTGCCGCCCGGACG
GCGGCGAAAGGACAGCGGTGCACCCCGCCCGAGGCCCCAGCGATCCATCAGGGCGGCTGTACGCGGGAAGACGCTTC
ATCTCATCCCTAGATGGCAGCAGGATTCTGAAGCTGGCGCAGCCCTGGGCAGCCCTTTCTGGTCTATTTTCCTGAAC
AGATTGCCAGGGGATACGGCGGCTAAACAGCCAGGCGCCTCCCCGGAGCGCAGCTCAGACAGAAGAGCAAGCCGGAC
AGACGCCGCTGGCGAGGCTTCGGGGGTCAGATCCTGCAGCTTGGACACCACATTCTCTCTTTTTGCACACAGATGTG
CTACTGTGTAAAAATAAGCAAACAAACAAAAACGGGAGTTCAGCTTCTTGGAGAATGTGTCATGGTCAAGACCAGTG
ATTCTGGACGGTGACAAAAGTCCTGGAGGCCTGAGTGCACAGAGGACACACGCACAGATGCACATGAACTCATGGAG
AAACACTGGTCTTCTCTGCCAGGGACCAGGCTGGCCATGCAGGAAGGGAGCAGGATGTGAAGATTCAGACTTTAGCT
CCAGCTTCACTACCGTCCTGCCGTGCACTTTGGGGGA

RNA
>LINC01901
ACCUAAUCAAGCCUGGGCAAUGGCGGGCGCCCCUCCCCCAGCCUUGCUGCCGCCUUGCAGUUUGAUCUCAGACUGCU
GUGCUAGCAAUCAGCGAGACUCCGUGGGUGUAGGACCCUCUGAGCCAGGUGUGGGAUAUAAUCUCGUGGUGCGCCGU
UUUUUAAGCCCGUCGGAAAAGCGCAGUAUUCAGGUAUCACCAGCUGUUUAUUUCUUCAGACAUAGUUAAAAUCUAAA
AGAAAAAAGAAAAAAAAGUCCAACAUUCAGAAUCCCCAAGAUGCAUACGUAAAUCCUAAUAGAAAAUGCUCAGUUCA
AGAAGUUCCAUUGAGGUGAAGCAAUGAUGCAAGGCUUUUGGCAACCGUUCCCCAGCUCCCAGCCACACAUCGCUCCA
CUUACCUUGAGUGACAGCCUCCAAAGAAAAGUUUCCACCGCGCCAGAGUCUUGACCCUGAGUGGCAUCGGCAGGGUC
UCCUGCGGCAGACCAGGGGUGUUGCGGGCUGCGCUGGGUGGGGGACCGCGCGCUGCACGUGCAGUGCCGCCCGGACG
GCGGCGAAAGGACAGCGGUGCACCCCGCCCGAGGCCCCAGCGAUCCAUCAGGGCGGCUGUACGCGGGAAGACGCUUC
AUCUCAUCCCUAGAUGGCAGCAGGAUUCUGAAGCUGGCGCAGCCCUGGGCAGCCCUUUCUGGUCUAUUUUCCUGAAC
AGAUUGCCAGGGGAUACGGCGGCUAAACAGCCAGGCGCCUCCCCGGAGCGCAGCUCAGACAGAAGAGCAAGCCGGAC
AGACGCCGCUGGCGAGGCUUCGGGGGUCAGAUCCUGCAGCUUGGACACCACAUUCUCUCUUUUUGCACACAGAUGUG
CUACUGUGUAAAAAUAAGCAAACAAACAAAAACGGGAGUUCAGCUUCUUGGAGAAUGUGUCAUGGUCAAGACCAGUG
AUUCUGGACGGUGACAAAAGUCCUGGAGGCCUGAGUGCACAGAGGACACACGCACAGAUGCACAUGAACUCAUGGAG
AAACACUGGUCUUCUCUGCCAGGGACCAGGCUGGCCAUGCAGGAAGGGAGCAGGAUGUGAAGAUUCAGACUUUAGCU
CCAGCUUCACUACCGUCCUGCCGUGCACUUUGGGGGA

>NR_151703.1 Homo sapiens long intergenic non-protein coding RNA 1902 (LINC01902), long non-coding RNA
GCTGGGGCCTCGGGCGGGGTGCACCGCTGTCCTTTCGCCGCCGTCCGGGCGGCACTGCACGTGCAGCGCG
CGGTCCCCCACCCAGCGCAGCCCGCAACACCCCTGGTCTGCCGCAGGAGACCCTGCCGATGCCACTCAGG
GTCAAGACTCTGGCGCGGTGGAAACTGCAGAGCAAAAGGAAACACAGACAAAAACAGTGAGAAGGAGAGA
GAGCATGTGGGGCTTTGCCAGAGAGGAACTTGGATGGCAATCACCTAAGAACTCCAAACTCAGTGCCAGG
GCAGACTGGTGTCTATTTTGCAATGTCTTCCAGTAGTTGATAACTTTCTACTTTTAAAATAATTGGAAGA
ATGTAAGCTCTCAACTAGTCCACTAGTCCCGAAACAATAGATACCAGCTGGCAGAGATAATAGCTGTCTG
GAAGAGAGAGATACATACCACATCAAAAAACAACAAAAAAAGGATGGCTCAAGTACTCAAAAGGCAGCAC
TAAAATAAGGCTATGTCTCTTCCTGAGAATACTCTGGACTTCATTCTCTGATTAAAGATCACAACTGCTT
CTTCGTTGAGGAAATCATGTATTTCAAAGCCACCAATGAACAAGGTAATGGGATGTATTGGTCCAGCACT
GATAACAGGTTATCCTACAATCAAGTATACTCTTTCCAGAAAGCTGAGTTAGGTATGCAACTGCCTTTCT
AAGCCATGACTACATTGCTAAATGAATCATTGATTTGGAAAATCTGAAGTGACCTTCAGCTTGTTCTCCA
GCTCTTCCTCAACAGAAAAGAGAGGCAAAATCAGCAAAATATTGGAAAGAGATTTTAACCTGAATGGAAA
AGACAATATGAGGGTGATGAAAGACTCTAGATGGTCCGTCTTGGAGAGAAGGTTTTGCCGTCTTGCCCCA
GTTGGTCTCGAACTCCCGGGCTCAAGCAATCCACCTGCCTTGGCCTCCCAAAGTGCTGGGATTACAGACA
CCAGTAATGAGGTGTCTCCTGGCACACAATCACAATGCACTGACAAACCATCTTGGTGTGTGAAAAAGAC
TCAGAAAAATCCAAGCAATGTGACACTACTAGCAAGCATAGTTCCACTTCACCAGAGACTTCAGGTCATA
GTTGGGCCTAAGGCAGAGTCGCTTGGCTGGAGAAGTCATTATTTATAGGACTATAGACCAAGTTTCATGT
CTGAGAGGCTCTTACGTTATTACATAGAAACAAAATTTTGTTATCTATAAGAATGATTTGCGTCTTTCTC
CTTACGTTAGTAATCCTAAGAAAATATTGAAAATGAATCATTCTTTAAAACAATTAAACAATTTACTGAT
TATTTTCACCACTATATGATGCACATGTGCCTACCAAGAAATCAGATATTTTCTTGTGTGTAGCTTCATT
CAATAGAGTAAAATAGAAAACTTTTATTATTAATAAAAATGTGAAATTCCATGTCATATACTATGATGTA
TAATCTTCTATGCTTCATTGTGTCTATCCTTTAGCATCACCATTGACTGACTTCAAGAGTACACTTTCCC
CTTACATAGCTGTGCTAGTTGCTACAGGTAATATTTAAAAGCATAAAATACAGTCTCTGACTCTGTGCTT
TAACTTAATCCAGTTGGAGAAACAGAATATGTGTAAAAAGATGAAAAATTATGGAATTTAATAAAATCTA
ATTCCAAATGGAAAATATAGGCAAGAAATATGATAAGACATCAATACCTGAGAACAAGATTACCATTGGA
GAGGGTAGACCAGGTGCAGTTTTTCTATGACTAGTAGACTCCGAAAGAAGGGAATTCTAAACAAGAAGGT
ATGTGGAAGAAGCAGTATATTTTAAACCCTCAACTCTTGTTAGAAAAAACAAACATCCTCTGAGGACTTC
CTCATAGGTAGCAATATGATAAGCAATAATGCTCTGCATCAGGAATGAAATTAGGTATTAAAGGAGGGAA
GATCTAACAAGTAGAAGTTATAATTACAGACCTTGAAACTCATGTTCTAGATCAGCGGTCTCCACCCTTT
TTGGCACTAGGGACCAGTTTTGTGGAAGACAGTTTTTCCACGGACCAGGGTAGGAGATGCTTTTGGGATG
ATTCAGGCACATTACATTTATTGTGCACTTTATTTCTATTATTATTACATTGTAATATATAATGAAATAA
TTATACAACTCACTATAAAATAGAATCACTGGGAGCCCTGAGCTTGTTTTCCTGCAACTAGATGGTCCCA
TATGGGGATGATGGGAGACAGTGACAGATCATCAAGCATTAGATGCTCATAAGAAGCAAGGAACTGAGAT
CCTTGCAACCTAGATCCATTGCATGTGTGGTTCACAATAGGTTTTGCACTCCTGTGGGAATCTAATGCCA
CTGCTGATCTGACAAGAGGCGGAGCTTAGGTGGTAATGTGTCTGGAAATACAGATGACGCTTTGCTCACT
CACCTGCTGCTTACCTCCTGCTGCGTAGCCTGGTTATAAACAGGCCATGAACTGGTACTGGTCTGTGGCC
TAGGGGTTGGGGGCTCCTGTTCTAGATTTTTTAGAGAAGACTACCATGTAAAATGATAGTGAATAATTAT
TAATATTAATAATATAATAGGTCTAATTGCTAAATCATATATCATAAAATCTAGCCTTTATGATAATTTA
CCTTTTAAACAGTCAATAAAAACTGGAACAATCTAGTAGGGTATCACAGCGGTGATAGGTCTTAACTTGC
ATAATATTTGAATTTATAGAGAGAATAAATACCACATTGAAGTAAAGCACAAAATAAGCCATGAAGTCAG
TACTCGGTATTGCATGTTCGTGAGACAGTGAAGAATTTAATTCAATTACAGACATGCCATAGGGAATAAT
GATTTACCAAACTTTATCGAGTTAAGTGATAAAAAGTGTTATGTTAGGTAGATTAATCAAGGGTAGTGGT
TCCATTCTTTGGTTTTTAGATGCTAGGAAGGCTGTGGAAATAAAGTTGCATGTAGCTCCACATACACACT
GAAACATGTATCTACCAAGCAATAAACATACTTTCAACATGTTTCCTTTAAATATGTATAAATAGGCCAG
GCGTGGTGGCTCACACCTGTAATCCCAATAATTTGGGAGGATGAGGCTGGTGAATCACTTGAATTGAGGA
GTTTAAGAGCAGTCTGGGCAACATGGTGAAATCCTGTTTCTACAAAAAAATACCCCCCAAAATGCTTATA
AATATGACTACTACAATAAAAATAAATTTAATGAATATAATTTATTGTAATAGGCCATTCTATATTTTTT
CAGTCAACAGATGTGATTATTATCTTACACTGGGAGATGCCTACCTGTCTCGTCTCCTCTCCTTTCCTCT
CCTCTCCTCTCCTCTCCTTTCCCTTTTCCCTTCTTTTCTTCCCTCCTCTCCCCTCTCCTTCCCCTTCTCC
TTCCCTTCCCTTCCCCTTCCCTACTCCTTCCCTTCTTTGCCTAAGCTGGAGTGCAGTGGCATGATCTCAG
CTCACTGCAACATCCACCTCCCTGGTCCAAGCAATTCTCCCACCTCAGCCTCCCTAGTAGCTGGGATTAC
AGGCGCACCACCACATCTGGCTAATTTTTGTATTTTTTAGTAGAGACAGGGTTTCACCATGTTGGCCAGG
CTGGTCCCGAACTCCTGATCTCAGGTGATCTACCCTCTTTGGCCTCCCAAAGTGCTAGGATTACAGGCGT
GAGCCACCACGCCCCACTTTAATTTTGTATTTTTAGTAGAGACGGGGTTTCACCATGTTGGCAAGGCTGA
TCTCTAACTCCTGACCTTGTGATCTGCCCATCTATGCCTCCCAAAGTGTTGAGATCACAGGCGCGAGCCA
CCGCGCCCAGCCTCCTCTCCTCTCCTCTCCTTTCCTTTTCGCTTTTCCCTTTCCTTCTTCCTTCCCCTCT
CCTCCCTCCCCTTCCCCTTTCCATCCCCCTCCTGTTCCCTTCTTTCCTTTGGCCTTGTGAAGTATCTATA
TATTATTTTCATTAAAGTGCTGTCAGGGCTGGGCGAGATGGATCATGCCTATAATCCCAGCACTTTGTTA
GGCAGAATCAGGCAGCTCACCTGAGGTCAAAAGTTCGAGACCAGCCTGGCCAACATTGCAAAACCCCGTC
TCTACTAAAGAATACAAAAATTAGTGGAGTGTGGTGGCATGTGACTGTAATCTCAGCTACTCAGGAGGCT
GAGGTAGGAGAATTGCTTGAACCCAGGAGGCGAAGTCTGCAGTGAGCCGAGAACTCGCCACTGTACTCCA
GCCTGGGAGACAGAGTGAGACTCCATCTCAAAAACAAAACAAAACAAAACAAAACAAAACAAAACAAAAC

AAACAAAAAAATTGCTGCTAGCCAGGCATAGTGACATGCACCTGTAGTTGTAGCTATTTGGAGGCTGAGG
TCAGAGGAAAGCTTGAGGCCAGAAGTTTGAGACCAATCTGGGCAACAAAGCAAGATCCCATCTCTAAAAT
ACAAAAACAAACAAACAAACAACCATGCTGCCAACTTACATTGACATATGCTTTTTAATTATCAAATATA
AAATGTACAGACTAAATGCTCAATATATTTTCATTTGTGTTGTTTGTTATTGGTTTACTGAGTTATGCTA
A

RNA
>LINC01902
GCUGGGGCCUCGGGCGGGGUGCACCGCUGUCCUUUCGCCGCCGUCCGGGCGGCACUGCACGUGCAGCGCG
CGGUCCCCCACCCAGCGCAGCCCGCAACACCCCUGGUCUGCCGCAGGAGACCCUGCCGAUGCCACUCAGG
GUCAAGACUCUGGCGCGGUGGAAACUGCAGAGCAAAAGGAAACACAGACAAAAACAGUGAGAAGGAGAGA
GAGCAUGUGGGGCUUUGCCAGAGAGGAACUUGGAUGGCAAUCACCUAAGAACUCCAAACUCAGUGCCAGG
GCAGACUGGUGUCUAUUUUGCAAUGUCUUCCAGUAGUUGAUAACUUUCUACUUUUAAAAUAAUUGGAAGA
AUGUAAGCUCUCAACUAGUCCACUAGUCCCGAAACAAUAGAUACCAGCUGGCAGAGAUAAUAGCUGUCUG
GAAGAGAGAGAUACAUACCACAUCAAAAAACAACAAAAAAAGGAUGGCUCAAGUACUCAAAAGGCAGCAC
UAAAAUAAGGCUAUGUCUCUUCCUGAGAAUACUCUGGACUUCAUUCUCUGAUUAAAGAUCACAACUGCUU
CUUCGUUGAGGAAAUCAUGUAUUUCAAAGCCACCAAUGAACAAGGUAAUGGGAUGUAUUGGUCCAGCACU
GAUAACAGGUUAUCCUACAAUCAAGUAUACUCUUUCCAGAAAGCUGAGUUAGGUAUGCAACUGCCUUUCU
AAGCCAUGACUACAUUGCUAAAUGAAUCAUUGAUUUGGAAAAUCUGAAGUGACCUUCAGCUUGUUCUCCA
GCUCUUCCUCAACAGAAAAGAGAGGCAAAAUCAGCAAAAUAUUGGAAAGAGAUUUUAACCUGAAUGGAAA
AGACAAUAUGAGGGUGAUGAAAGACUCUAGAUGGUCCGUCUUGGAGAGAAGGUUUUGCCGUCUUGCCCCA
GUUGGUCUCGAACUCCCGGGCUCAAGCAAUCCACCUGCCUUGGCCUCCCAAAGUGCUGGGAUUACAGACA
CCAGUAAUGAGGUGUCUCCUGGCACACAAUCACAAUGCACUGACAAACCAUCUUGGUGUGUGAAAAAGAC
UCAGAAAAAUCCAAGCAAUGUGACACUACUAGCAAGCAUAGUUCCACUUCACCAGAGACUUCAGGUCAUA
GUUGGGCCUAAGGCAGAGUCGCUUGGCUGGAGAAGUCAUUAUUUAUAGGACUAUAGACCAAGUUUCAUGU
CUGAGAGGCUCUUACGUUAUUACAUAGAAACAAAAUUUUGUUAUCUAUAAGAAUGAUUUGCGUCUUUCUC
CUUACGUUAGUAAUCCUAAGAAAAUAUUGAAAAUGAAUCAUUCUUUAAAACAAUUAAACAAUUUACUGAU
UAUUUUCACCACUAUAUGAUGCACAUGUGCCUACCAAGAAAUCAGAUAUUUUCUUGUGUGUAGCUUCAUU
CAAUAGAGUAAAAUAGAAAACUUUUAUUAUUAAUAAAAAUGUGAAAUUCCAUGUCAUAUACUAUGAUGUA
UAAUCUUCUAUGCUUCAUUGUGUCUAUCCUUUAGCAUCACCAUUGACUGACUUCAAGAGUACACUUUCCC
CUUACAUAGCUGUGCUAGUUGCUACAGGUAAUAUUUAAAAGCAUAAAAUACAGUCUCUGACUCUGUGCUU
UAACUUAAUCCAGUUGGAGAAACAGAAUAUGUGUAAAAAGAUGAAAAAUUAUGGAAUUUAAUAAAAUCUA
AUUCCAAAUGGAAAAUAUAGGCAAGAAAUAUGAUAAGACAUCAAUACCUGAGAACAAGAUUACCAUUGGA
GAGGGUAGACCAGGUGCAGUUUUUCUAUGACUAGUAGACUCCGAAAGAAGGGAAUUCUAAACAAGAAGGU
AUGUGGAAGAAGCAGUAUAUUUUAAACCCUCAACUCUUGUUAGAAAAAACAAACAUCCUCUGAGGACUUC
CUCAUAGGUAGCAAUAUGAUAAGCAAUAAUGCUCUGCAUCAGGAAUGAAAUUAGGUAUUAAAGGAGGGAA
GAUCUAACAAGUAGAAGUUAUAAUUACAGACCUUGAAACUCAUGUUCUAGAUCAGCGGUCUCCACCCUUU
UUGGCACUAGGGACCAGUUUUGUGGAAGACAGUUUUUCCACGGACCAGGGUAGGAGAUGCUUUUGGGAUG
AUUCAGGCACAUUACAUUUAUUGUGCACUUUAUUUCUAUUAUUAUUACAUUGUAAUAUAUAAUGAAAUAA
UUAUACAACUCACUAUAAAAUAGAAUCACUGGGAGCCCUGAGCUUGUUUUCCUGCAACUAGAUGGUCCCA
UAUGGGGAUGAUGGGAGACAGUGACAGAUCAUCAAGCAUUAGAUGCUCAUAAGAAGCAAGGAACUGAGAU
CCUUGCAACCUAGAUCCAUUGCAUGUGUGGUUCACAAUAGGUUUUGCACUCCUGUGGGAAUCUAAUGCCA
CUGCUGAUCUGACAAGAGGCGGAGCUUAGGUGGUAAUGUGUCUGGAAAUACAGAUGACGCUUUGCUCACU
CACCUGCUGCUUACCUCCUGCUGCGUAGCCUGGUUAUAAACAGGCCAUGAACUGGUACUGGUCUGUGGCC
UAGGGGUUGGGGGCUCCUGUUCUAGAUUUUUUAGAGAAGACUACCAUGUAAAAUGAUAGUGAAUAAUUAU
UAAUAUUAAUAAUAUAAUAGGUCUAAUUGCUAAAUCAUAUAUCAUAAAAUCUAGCCUUUAUGAUAAUUUA
CCUUUUAAACAGUCAAUAAAAACUGGAACAAUCUAGUAGGGUAUCACAGCGGUGAUAGGUCUUAACUUGC
AUAAUAUUUGAAUUUAUAGAGAGAAUAAAUACCACAUUGAAGUAAAGCACAAAAUAAGCCAUGAAGUCAG
UACUCGGUAUUGCAUGUUCGUGAGACAGUGAAGAAUUUAAUUCAAUUACAGACAUGCCAUAGGGAAUAAU
GAUUUACCAAACUUUAUCGAGUUAAGUGAUAAAAAGUGUUAUGUUAGGUAGAUUAAUCAAGGGUAGUGGU
UCCAUUCUUUGGUUUUUAGAUGCUAGGAAGGCUGUGGAAAUAAAGUUGCAUGUAGCUCCACAUACACACU
GAAACAUGUAUCUACCAAGCAAUAAACAUACUUUCAACAUGUUUCCUUUAAAUAUGUAUAAAUAGGCCAG
GCGUGGUGGCUCACACCUGUAAUCCCAAUAAUUUGGGAGGAUGAGGCUGGUGAAUCACUUGAAUUGAGGA
GUUUAAGAGCAGUCUGGGCAACAUGGUGAAAUCCUGUUUCUACAAAAAAAUACCCCCCAAAAUGCUUAUA
AAUAUGACUACUACAAUAAAAAUAAAUUUAAUGAAUAUAAUUUAUUGUAAUAGGCCAUUCUAUAUUUUUU
CAGUCAACAGAUGUGAUUAUUAUCUUACACUGGGAGAUGCCUACCUGUCUCGUCUCCUCUCCUUUCCUCU
CCUCUCCUCUCCUCUCCUUUCCCUUUUCCCUUCUUUUCUUCCCUCCUCUCCCCUCUCCUUCCCCUUCUCC
UUCCCUUCCCUUCCCCUUCCCUACUCCUUCCCUUCUUUGCCUAAGCUGGAGUGCAGUGGCAUGAUCUCAG
CUCACUGCAACAUCCACCUCCCUGGUCCAAGCAAUUCUCCCACCUCAGCCUCCCUAGUAGCUGGGAUUAC
AGGCGCACCACCACAUCUGGCUAAUUUUUGUAUUUUUUAGUAGAGACAGGGUUUCACCAUGUUGGCCAGG
CUGGUCCCGAACUCCUGAUCUCAGGUGAUCUACCCUCUUUGGCCUCCCAAAGUGCUAGGAUUACAGGCGU
GAGCCACCACGCCCCACUUUAAUUUUGUAUUUUUAGUAGAGACGGGGUUUCACCAUGUUGGCAAGGCUGA
UCUCUAACUCCUGACCUUGUGAUCUGCCCAUCUAUGCCUCCCAAAGUGUUGAGAUCACAGGCGCGAGCCA
CCGCGCCCAGCCUCCUCUCCUCUCCUCUCCUUUCCUUUUCGCUUUUCCCUUUCCUUCUUCCUUCCCCUCU

CCUCCCUCCCCUUCCCCUUUCCAUCCCCCUCCUGUUCCCUUCUUUCCUUUGGCCUUGUGAAGUAUCUAUA
UAUUAUUUUCAUUAAAGUGCUGUCAGGGCUGGGCGAGAUGGAUCAUGCCUAUAAUCCCAGCACUUUGUUA
GGCAGAAUCAGGCAGCUCACCUGAGGUCAAAAGUUCGAGACCAGCCUGGCCAACAUUGCAAAACCCCGUC
UCUACUAAAGAAUACAAAAAUUAGUGGAGUGUGGUGGCAUGUGACUGUAAUCUCAGCUACUCAGGAGGCU
GAGGUAGGAGAAUUGCUUGAACCCAGGAGGCGAAGUCUGCAGUGAGCCGAGAACUCGCCACUGUACUCCA
GCCUGGGAGACAGAGUGAGACUCCAUCUCAAAAACAAAACAAAACAAAACAAAACAAAACAAAACAAAAC
AAACAAAAAAAUUGCUGCUAGCCAGGCAUAGUGACAUGCACCUGUAGUUGUAGCUAUUUGGAGGCUGAGG
UCAGAGGAAAGCUUGAGGCCAGAAGUUUGAGACCAAUCUGGGCAACAAAGCAAGAUCCCAUCUCUAAAAU
ACAAAAACAAACAAACAAACAACCAUGCUGCCAACUUACAUUGACAUAUGCUUUUUAAUUAUCAAAUAUA
AAAUGUACAGACUAAAUGCUCAAUAUAUUUUCAUUUGUGUUGUUUGUUAUUGGUUUACUGAGUUAUGCUA
A

>NR_146471.1 Homo sapiens long intergenic non-protein coding RNA 1903 (LINC01903), long non-coding RNA
GGTTCACGCTCCTGCCTCAGCCTCCCGAGTAGCTGGAACTACAGGCGCCCGCCACCACGCCCAGCTAATT
TTTGTATTTTCAGTAGAGACAGGATTTCATTGTGTTAGCCGGGATGGTCTCGATCTCCTGACCTCGTGAT
CCACCCGCCTCGGCCTCCCAAAGTGCTGGGATTACAGGCGTGAGCCACCACACCCCGCCACTTCAAATAC
TCTTTAACTTTACACTTAAGCAAAGAAATATTACATATTTCAGTCCTGAAAATATTATGATAAATCAAAT
AGACCCTATGACATAGGATATATTAAATATTTAAGTTTTGGAAATGTCCTTTATATACAAAATATTTCCA
TTGTTAACAAGTGCTTTTCAAAAAATATTTTTATTATACCTTAGAAAGAGCCAATTTAGGGTCTACACTA
TCTTTGTTCATCTCGTTTTCTTTTTTGCACATATTCCTTTACTAATGTAGATTAGCACTACAAGATAAAA
AGTATCCAGCATCATAACATTGTAATTAATTCCCCCATATCAAATTATATGAAAAATCCCACCTCAGACA
CTCTGCCAAAGGGTATGGAACATTTAATCAAGATGAAGTGCTTCAATTGAAGCAGTATATGCTCACCTCT
ATCAGTAATTTTGTCTCACTTTATTTCAAAGTATTATTATTTTTCGTCCCAAGATTTTAGTAACACGTTG
TGTTAAATTCTTGTCATATTTAAAATTAAACCCTCTTATTCGTATTATATTTAAAAGCAAATTTTCATTG
TTTCAATGAAATCGTTCAGTATACTCGGCAAAGTTTATAGTAAAGTTCTGAACAATAAATACATTCTGAA
ATGTCCTGTGCTTTCAAAAGTACATATTAGAGCTGATGGCTTAAGCACCATTATTTAGTGGCATTTCTAA
TTATTACTTTCCTTTATGTGGTTTTTTTTAAGAGGCACCAAAGTTCCTTTTGTCACATGGCTCGTGTTGC
CTTCAGTAACAGCCACTTGAATTTGCCCTTAGTTGCAAACTCAGATCATGACACAAAATATACTCATAAA
AGAACTTTATTCTGGACATTCTTCCAGACTATGGAAATGATGTTAGACAAAAAGCAAACTTGAGCGATTT
TCTCATTCGAGTTCAAAATGGGTCATAAAGCAGTGGAGACAATTCGCAATATGAACAACACATTTGGCCC
AGGGACTGTTAATGAAGGTACAGTGCGGGGGCGGTTCGAGAAGTTTTGCAAAAGAGATGAGACCCTTGAA
GACGAGGAGCATAATGGTTGGTCACAGGAAGTTGACAACAACCGAGAGCAATCATTGAAGCTGATCCTCT
CACAACACCACAAGAAGTTGCAGAACTCATTGTTGACCATTCCACAGTCATTCGGCCTTTGAAGCAAATT
GGGAAGGTGAGAACTCTAGATAAGTGGGATGCCCCATGAGCTGAGCCAAAATAAAATAAAAAAGTCATTT
TGAAGTGTCATCTTCTCTTATTCTATGCAACAACAATGAAACACTTTTCTATTGGATTGTGATGTGCGAC
GAAATGTAGATTTTATAGGACAACCAGTGATGACCAGCTGAATAACTGGATCCAGAAGAAGCTCCAAAGC
ACTTCCCAAAGCCAAACTTGCACCAAATAAAAGGTCATCGTCATTGTTGGGTGCTCTGCTGCCAGCCTAA
TTTACTACAGCTTTCTGAATCCTAATGAAACTATTACATCTGAGACGTATGCTCAGCAAATCGATGAGAT
GTCCTGAAAACTGCAATGCCTGCAGCCGACACTGGTCCACAGAAAAAGCCCAATTATTCCCCACAACTAC
ACGTGACCGCATGTAGCACAACCAATGCATCAAAAGTCGAACAAATTGGGCTACAAATTTTTGCCTCGTC
CACTTTATTCACCTGACCTCTCACCAACTGACCTCCCAACTCTCTGCCACTTCTTCAAGCATCTCGCCAA
CTTTTTGCAAGGAAAAAGCTTCCACAACTAGTAGGAGGCAGAAAATGCTTTCCAAGAGTTCATCGAATCC
TGAAGCACGTATTTTTTTTTTTATGCCATGGAAATAAACAAACTTATTTTCTTTCTTTGGCAAAAATGTG
TTGATTGTAATGGTTCCTATTTTGATTAATAAAGATGTATTTGTGCC

RNA
>LINC01903
GGUUCACGCUCCUGCCUCAGCCUCCCGAGUAGCUGGAACUACAGGCGCCCGCCACCACGCCCAGCUAAUU
UUUGUAUUUUCAGUAGAGACAGGAUUUCAUUGUGUUAGCCGGGAUGGUCUCGAUCUCCUGACCUCGUGAU
CCACCCGCCUCGGCCUCCCAAAGUGCUGGGAUUACAGGCGUGAGCCACCACACCCCGCCACUUCAAAUAC
UCUUUAACUUUACACUUAAGCAAAGAAAUAUUACAUAUUUCAGUCCUGAAAAUAUUAUGAUAAAUCAAAU
AGACCCUAUGACAUAGGAUAUAUUAAAUAUUUAAGUUUUGGAAAUGUCCUUUAUAUACAAAAUAUUUCCA
UUGUUAACAAGUGCUUUUCAAAAAAUAUUUUUAUUAUACCUUAGAAAGAGCCAAUUUAGGGUCUACACUA
UCUUUGUUCAUCUCGUUUUCUUUUUUGCACAUAUUCCUUUACUAAUGUAGAUUAGCACUACAAGAUAAAA
AGUAUCCAGCAUCAUAACAUUGUAAUUAAUUCCCCCAUAUCAAAUUAUAUGAAAAAUCCCACCUCAGACA
CUCUGCCAAAGGGUAUGGAACAUUUAAUCAAGAUGAAGUGCUUCAAUUGAAGCAGUAUAUGCUCACCUCU
AUCAGUAAUUUUGUCUCACUUUAUUUCAAAGUAUUAUUAUUUUUCGUCCCAAGAUUUUAGUAACACGUUG
UGUUAAAUUCUUGUCAUAUUUAAAAUUAAACCCUCUUAUUCGUAUUAUAUUUAAAAGCAAAUUUUCAUUG
UUUCAAUGAAAUCGUUCAGUAUACUCGGCAAAGUUUAUAGUAAAGUUCUGAACAAUAAAUACAUUCUGAA
AUGUCCUGUGCUUUCAAAAGUACAUAUUAGAGCUGAUGGCUUAAGCACCAUUAUUUAGUGGCAUUUCUAA
UUAUUACUUUCCUUUAUGUGGUUUUUUUUAAGAGGCACCAAAGUUCCUUUUGUCACAUGGCUCGUGUUGC
CUUCAGUAACAGCCACUUGAAUUUGCCCUUAGUUGCAAACUCAGAUCAUGACACAAAAUAUACUCAUAAA
AGAACUUUAUUCUGGACAUUCUUCCAGACUAUGGAAAUGAUGUUAGACAAAAAGCAAACUUGAGCGAUUU
UCUCAUUCGAGUUCAAAAUGGGUCAUAAAGCAGUGGAGACAAUUCGCAAUAUGAACAACACAUUUGGCCC
AGGGACUGUUAAUGAAGGUACAGUGCGGGGGCGGUUCGAGAAGUUUUGCAAAAGAGAUGAGACCCUUGAA
GACGAGGAGCAUAAUGGUUGGUCACAGGAAGUUGACAACAACCGAGAGCAAUCAUUGAAGCUGAUCCUCU
CACAACACCACAAGAAGUUGCAGAACUCAUUGUUGACCAUUCCACAGUCAUUCGGCCUUUGAAGCAAAUU
GGGAAGGUGAGAACUCUAGAUAAGUGGGAUGCCCCAUGAGCUGAGCCAAAAUAAAAUAAAAAAGUCAUUU
UGAAGUGUCAUCUUCUCUUAUUCUAUGCAACAACAAUGAAACACUUUUCUAUUGGAUUGUGAUGUGCGAC
GAAAUGUAGAUUUUAUAGGACAACCAGUGAUGACCAGCUGAAUAACUGGAUCCAGAAGAAGCUCCAAAGC
ACUUCCCAAAGCCAAACUUGCACCAAAUAAAAGGUCAUCGUCAUUGUUGGGUGCUCUGCUGCCAGCCUAA
UUUACUACAGCUUUCUGAAUCCUAAUGAAACUAUUACAUCUGAGACGUAUGCUCAGCAAAUCGAUGAGAU
GUCCUGAAAACUGCAAUGCCUGCAGCCGACACUGGUCCACAGAAAAAGCCCAAUUAUUCCCCACAACUAC
ACGUGACCGCAUGUAGCACAACCAAUGCAUCAAAAGUCGAACAAAUUGGGCUACAAAUUUUUGCCUCGUC
CACUUUAUUCACCUGACCUCUCACCAACUGACCUCCCAACUCUCUGCCACUUCUUCAAGCAUCUCGCCAA

CUUUUUGCAAGGAAAAAGCUUCCACAACUAGUAGGAGGCAGAAAAUGCUUUCCAAGAGUUCAUCGAAUCC
UGAAGCACGUAUUUUUUUUUUUAUGCCAUGGAAAUAAACAAACUUAUUUUCUUUCUUUGGCAAAAAUGUG
UUGAUUGUAAUGGUUCCUAUUUUGAUUAAUAAAGAUGUAUUUGUGCC

>NR_146724.1 Homo sapiens long intergenic non-protein coding RNA 1904 (LINC01904), long non-coding RNA
AACATGTGAGCTCCTTGAGGGTAATCCCTCTCTCCCTGGTAGCCCTAGTACTGCCTTCAGGCAGATATCC
ACAAAAGCACCTTGCACAGAAACATCTTTGAGCTATTTTAAGAGGCAGCATTATTTTACTGACAAATGTT
CAGAATTGAGCTCTCTGACTTGCCCCCAGCCCAACCTCTATGGATTGACCATTCTCCTCAGAGAGACTGT
GTATGCCTGTGCAGGCACACATGTTGAGGGGAAGACAGGAAGACCTACCCTCCCCCAGGCCCTGGCTTCT
CTTCAGACAGTGCCATTCACACTGAGCCTCACAGGCAAGTACTGCCTGGTGTAACTTGGGGAGTGATGGG
GCCTGCTGACCAGAATCACTCCTCCAAAAGCTTCACACACAAGCCTGAGTTTCCAGTCATCACCAGCAGC
ACCTTCAGTCTTTGAGAGCCTTCTTAATGAACACACATATTTTACCTGGATCAAGACACTGTGTTATCTG
GCAAAGGTTGCCCATTTAAAATGAGTGACTGGAAAGGTTCGTTTATCTGGTCAACCCTCTACTGCCCCCC
TATTGTCACCCCTTTGGGATTAGAATCCTGAAAGAATGATGAGAAGAAAGGCTACATATGGAGAGACCTG
AGAAACCCCGAATCTTTCTTGAGAGGAGGAAATCAGACACCTGCACAGTCCAGCAGCCTGATCCCAGGAG
CCAGGGAGCAGGGAGCAGAGGAGAGTTCCAGGGACCAGAAGCCAGGAAGTGGGATTAAAGGCTAACACTG
GCCACACAAGACGGACCGCTGCACAGGAATTTACAGTTCACAGAGCACTTTTTCATCTCCTTAAAATCTC
ATAAAATCGCAGTGAATTGAGAAAAAAAAAAAAAAAAAAAAAAAAAAAAAA

RNA
>LINC01904
AACAUGUGAGCUCCUUGAGGGUAAUCCCUCUCUCCCUGGUAGCCCUAGUACUGCCUUCAGGCAGAUAUCC
ACAAAAGCACCUUGCACAGAAACAUCUUUGAGCUAUUUUAAGAGGCAGCAUUAUUUUACUGACAAAUGUU
CAGAAUUGAGCUCUCUGACUUGCCCCCAGCCCAACCUCUAUGGAUUGACCAUUCUCCUCAGAGAGACUGU
GUAUGCCUGUGCAGGCACACAUGUUGAGGGGAAGACAGGAAGACCUACCCUCCCCCAGGCCCUGGCUUCU
CUUCAGACAGUGCCAUUCACACUGAGCCUCACAGGCAAGUACUGCCUGGUGUAACUUGGGGAGUGAUGGG
GCCUGCUGACCAGAAUCACUCCUCCAAAAGCUUCACACACAAGCCUGAGUUUCCAGUCAUCACCAGCAGC
ACCUUCAGUCUUUGAGAGCCUUCUUAAUGAACACACAUAUUUUACCUGGAUCAAGACACUGUGUUAUCUG
GCAAAGGUUGCCCAUUUAAAAUGAGUGACUGGAAAGGUUCGUUUAUCUGGUCAACCCUCUACUGCCCCCC
UAUUGUCACCCCUUUGGGAUUAGAAUCCUGAAAGAAUGAUGAGAAGAAAGGCUACAUAUGGAGAGACCUG
AGAAACCCCGAAUCUUUCUUGAGAGGAGGAAAUCAGACACCUGCACAGUCCAGCAGCCUGAUCCCAGGAG
CCAGGGAGCAGGGAGCAGAGGAGAGUUCCAGGGACCAGAAGCCAGGAAGUGGGAUUAAAGGCUAACACUG
GCCACACAAGACGGACCGCUGCACAGGAAUUUACAGUUCACAGAGCACUUUUUCAUCUCCUUAAAAUCUC
AUAAAAUCGCAGUGAAUUGAGAAAAAAAAAAAAAAAAAAAAAAAAAAAAAA

>NR_146510.1 Homo sapiens long intergenic non-protein coding RNA 1905 (LINC01905), transcript variant 1, long non-coding RNA
TGATGCAGCAAGAAGGCCCTTGCCAGATGCCAGCAACTTGATATTGAACTTCTCATCCTCCAGAATGGTT
TGTTTTTCCTCTCTGTCATACCTCAAGAGGAACACAAAGAGTGATGGCCTGTCACTCTGATGCCTCTCTC
TACTCAAATGGCAGAAACCAACCAGGTTCTGTCAATAGAAGATACTAAAGAAAGAAAGCCAGGAGAAGAA
AAGGGACTTCTTTCTTCAAGGTCTAAGCTGTTCTAATAGCATTATTCTGGGAATGGCATCTTCCACTCCT
CCTAGTACAAAGAACCTGCTTCCTCATACCCCGTCAAGGAACTGCAGCATCCAGGTGATGGCCACAGTGG
CAGAACCTAGGAAGCCAAGAAGAAACCAGAGAATGGAGACACATGGCAGCAATGGCAAGTCATCAGACCC
AGGATTTCCCAAAGAACACTGGTCCAGGAAGATGCTCTGTGAAAAAGTGTTCCCTGGTCGGAGAAATTTG
TGAAACACTTTGCATAATACTTAAAAGAATGTATGGCACATCAAAGGTAATATGCAATAAAAGAATTCAT
TTTTTTAAAACCAAG

RNA
>LINC01905
UGAUGCAGCAAGAAGGCCCUUGCCAGAUGCCAGCAACUUGAUAUUGAACUUCUCAUCCUCCAGAAUGGUU
UGUUUUUCCUCUCUGUCAUACCUCAAGAGGAACACAAAGAGUGAUGGCCUGUCACUCUGAUGCCUCUCUC
UACUCAAAUGGCAGAAACCAACCAGGUUCUGUCAAUAGAAGAUACUAAAGAAAGAAAGCCAGGAGAAGAA
AAGGGACUUCUUUCUUCAAGGUCUAAGCUGUUCUAAUAGCAUUAUUCUGGGAAUGGCAUCUUCCACUCCU
CCUAGUACAAAGAACCUGCUUCCUCAUACCCCGUCAAGGAACUGCAGCAUCCAGGUGAUGGCCACAGUGG
CAGAACCUAGGAAGCCAAGAAGAAACCAGAGAAUGGAGACACAUGGCAGCAAUGGCAAGUCAUCAGACCC
AGGAUUUCCCAAAGAACACUGGUCCAGGAAGAUGCUCUGUGAAAAAGUGUUCCCUGGUCGGAGAAAUUUG
UGAAACACUUUGCAUAAUACUUAAAAGAAUGUAUGGCACAUCAAAGGUAAUAUGCAAUAAAAGAAUUCAU
UUUUUUAAAACCAAG

>NR_170236.1 Homo sapiens long intergenic non-protein coding RNA 1906 (LINC01906), long non-coding RNA
AGGAATATGTGGAAATTCTCCTGGGTTTTCCAGCTCCTGAGAGAGCCCTGGCTTTGTGGGGTGAAGTCCT
TTGATTGCCTCAGGTTCCTGATCTGTTCGTGACCCTGTTAGCCCAAAAGCTGGCAGAGCTCCATTCTTTT
TAGGTCTTATTCTCAGACGGTCTTGGGTAAATGGTGGCTGATTGCCTTCAGCATCTAGAACAAAACACTC
TATGGCCCAGAGGAATTCCAGCTGGAATCTATGATCTTATTCTCTTGAGGAATGGACCATGCAGTATCCT
TCAAGGAAGAATCAAGAATGTTTCCACAGAGAATATACATATTTCATCTCTTTCTCTGCTGAATGGATCC
CCACTGAAAGCCATGAATTTCTCTTATTAGACCCTGTAGAATCCCAGTTTATGGCAGTGACATTAAAAAC
ATCTAGTTTATGACTTGGATTAGGCAGCCTGTAAAATGGCTTCCAGTGATCCCTGCT

RNA
>LINC01906
AGGAAUAUGUGGAAAUUCUCCUGGGUUUUCCAGCUCCUGAGAGAGCCCUGGCUUUGUGGGGUGAAGUCCU
UUGAUUGCCUCAGGUUCCUGAUCUGUUCGUGACCCUGUUAGCCCAAAAGCUGGCAGAGCUCCAUUCUUUU
UAGGUCUUAUUCUCAGACGGUCUUGGGUAAAUGGUGGCUGAUUGCCUUCAGCAUCUAGAACAAAACACUC
UAUGGCCCAGAGGAAUUCCAGCUGGAAUCUAUGAUCUUAUUCUCUUGAGGAAUGGACCAUGCAGUAUCCU
UCAAGGAAGAAUCAAGAAUGUUUCCACAGAGAAUAUACAUAUUUCAUCUCUUUCUCUGCUGAAUGGAUCC
CCACUGAAAGCCAUGAAUUUCUCUUAUUAGACCCUGUAGAAUCCCAGUUUAUGGCAGUGACAUUAAAAAC
AUCUAGUUUAUGACUUGGAUUAGGCAGCCUGUAAAAUGGCUUCCAGUGAUCCCUGCU

>XR_002958212.2 PREDICTED: Homo sapiens long intergenic non-protein coding RNA 1908 (LINC01908), ncRNA
ATTCCCACTGTGCCCCCACAACAGCACCGAAGCACCGAGTTTATTTCCAGGCAGGCAGTGAGCAGGGCTG
AGAAATTGCCCCAGGCTACAAGCCTCCCAGCTGAGAAAGCAAGCAGACTCACAGTTCCTTGGCTGTCCCA
TGCAGCCTGCAGCAGCAATCCACCCCCTTTAAAGTGTCTGTGGATTCTTTCAGCTTTTTTGGTTCAAGCA
ATTCTCCTGTCTCAGCCTCCCGAGTAGCTGGGATTACAGTCATGTGCCCCTGTGTCCAGAATTGGTTCCT
TCTGGTGGGTTCTTGGTCTTGCTGTCTTCAAGAATGAAGCCGTGGACCCTCGTCATGAATGTTACAGTTC
TTAAAGATGAAGAGAACAGCATCAGGGCACATTGAGGCTCAGAAATTTATATTCCAAGAAGAGGTGCACA
GGAACAGAGCAGATCCAAAGCAGACAACCACCTGGAAGCAGTCATAACAACAGCAATAGCAAATCAATGC
TGATAAAAGAAATCAAGTACAGAGAGTTTTAAGGACATTGTCCAAAGTTGTACAAGGGCACCATTTTTAA
AAGTTGACAACCTCCTGCAGGGATGTTCTGAAAGACAGAGACATCTTGTCGGAATTATGAATGTCCTTGG
TGGATGAGGCAGAAAGAGTTGACTGTGTAGGACATTCCTGACAAGTCCTCAGGATTTGAAATGACTCCAA
CACTAAGCATTCACCTGGAGATAACCTACAGAGATACTCTGTGTTACCCACAACTAGTCTGTTGACTAAG
TTGGTGAATATATTTGGGAAAAGCCCATGTGGAAGAATGGAAGCCTGCATTATCTTTCTCAGAGACAGAC
AGGAAGGTGACTCTATGGAAGACATCAGCGATAAACTAGGAAGGAGTGACACGGTAAGTGAAAATAGAGA
TATGCAGTGTG

RNA
>LINC01908
AUUCCCACUGUGCCCCCACAACAGCACCGAAGCACCGAGUUUAUUUCCAGGCAGGCAGUGAGCAGGGCUG
AGAAAUUGCCCCAGGCUACAAGCCUCCCAGCUGAGAAAGCAAGCAGACUCACAGUUCCUUGGCUGUCCCA
UGCAGCCUGCAGCAGCAAUCCACCCCCUUUAAAGUGUCUGUGGAUUCUUUCAGCUUUUUUGGUUCAAGCA
AUUCUCCUGUCUCAGCCUCCCGAGUAGCUGGGAUUACAGUCAUGUGCCCCUGUGUCCAGAAUUGGUUCCU
UCUGGUGGGUUCUUGGUCUUGCUGUCUUCAAGAAUGAAGCCGUGGACCCUCGUCAUGAAUGUUACAGUUC
UUAAAGAUGAAGAGAACAGCAUCAGGGCACAUUGAGGCUCAGAAAUUUAUAUUCCAAGAAGAGGUGCACA
GGAACAGAGCAGAUCCAAAGCAGACAACCACCUGGAAGCAGUCAUAACAACAGCAAUAGCAAAUCAAUGC
UGAUAAAAGAAAUCAAGUACAGAGAGUUUUAAGGACAUUGUCCAAAGUUGUACAAGGGCACCAUUUUUAA
AAGUUGACAACCUCCUGCAGGGAUGUUCUGAAAGACAGAGACAUCUUGUCGGAAUUAUGAAUGUCCUUGG
UGGAUGAGGCAGAAAGAGUUGACUGUGUAGGACAUUCCUGACAAGUCCUCAGGAUUUGAAAUGACUCCAA
CACUAAGCAUUCACCUGGAGAUAACCUACAGAGAUACUCUGUGUUACCCACAACUAGUCUGUUGACUAAG
UUGGUGAAUAUAUUUGGGAAAAGCCCAUGUGGAAGAAUGGAAGCCUGCAUUAUCUUUCUCAGAGACAGAC
AGGAAGGUGACUCUAUGGAAGACAUCAGCGAUAAACUAGGAAGGAGUGACACGGUAAGUGAAAAUAGAGA
UAUGCAGUGUG

>NR_126336.1 Homo sapiens long intergenic non-protein coding RNA 1909 (LINC01909), long non-coding RNA
TTGGGTCCTGTCTTGGTGATAAAGATAAAACTAAAGGACAATCAAATCTCATCAAACTTCTCTCTGAGGA
GCTGATGACGTTTCAGAAACAGTGACAAAGTTCAGAATCCCATTCTTCCATCCTAGATGGGAAACATCAA
CTTTCCTGATGATGCTGAAAAAGCAAGATACTCATTTTTTCCAGCCACCCTTGCAGAGCCAGAAAGCCCA
TGAGATTCAATAGATGTAAGACTGAGAGAGATTTTCATTCCTGGATGAAAGGAGACAGGCCTGGGAGAAG
AGTTTTCTCTTGCCATCTTTGCAGTCTCTCCACTCCTGACGTGTACAGGGTCAGGAAAGGCCATGTGAAA
ACATGGCGAGAAGACAGTCATCTGCAACCCAAGGAGAGAGACTTCACCAGACACTGACCCTGCTGGCACC
TTGATCTTGGACTTCCAGTGTTCAGAACTATAAGAAATAAATTCCTGTTATGTAAGGTGCCCAGTCCATG
GTATTTTGTTGTAACAGCCTGAGCTGTTGAAGATGGATTTTCCTTTCAAGATCAGATCTTGGGCCTCAGA
GTCAGCCTCACTATAGAAAGCAAGGGCTAGTCTCAGCTTCCCACAGAAGACTTGGTTTTATTGGAGGCAA
GTTTTGTCTTGCTTACAATGAGGTTATACAAAGCATAACAAAATTCACATTTTGTTAAGTGACTACCATA
TGATCACTCTGAATTTTATTTTACCTGTCATTTCTCGGTATCAATAGGTTTTACTTCCCCTTGTTCTGGA
TTTCAGTGATTGATGACTGAATACAATAAATATTTATTGAGCGCC

RNA
>LINC01909
UUGGGUCCUGUCUUGGUGAUAAAGAUAAAACUAAAGGACAAUCAAAUCUCAUCAAACUUCUCUCUGAGGA
GCUGAUGACGUUUCAGAAACAGUGACAAAGUUCAGAAUCCCAUUCUUCCAUCCUAGAUGGGAAACAUCAA
CUUUCCUGAUGAUGCUGAAAAAGCAAGAUACUCAUUUUUUCCAGCCACCCUUGCAGAGCCAGAAAGCCCA
UGAGAUUCAAUAGAUGUAAGACUGAGAGAGAUUUUCAUUCCUGGAUGAAAGGAGACAGGCCUGGGAGAAG
AGUUUUCUCUUGCCAUCUUUGCAGUCUCUCCACUCCUGACGUGUACAGGGUCAGGAAAGGCCAUGUGAAA
ACAUGGCGAGAAGACAGUCAUCUGCAACCCAAGGAGAGAGACUUCACCAGACACUGACCCUGCUGGCACC
UUGAUCUUGGACUUCCAGUGUUCAGAACUAUAAGAAAUAAAUUCCUGUUAUGUAAGGUGCCCAGUCCAUG
GUAUUUUGUUGUAACAGCCUGAGCUGUUGAAGAUGGAUUUUCCUUUCAAGAUCAGAUCUUGGGCCUCAGA
GUCAGCCUCACUAUAGAAAGCAAGGGCUAGUCUCAGCUUCCCACAGAAGACUUGGUUUUAUUGGAGGCAA
GUUUUGUCUUGCUUACAAUGAGGUUAUACAAAGCAUAACAAAAUUCACAUUUUGUUAAGUGACUACCAUA
UGAUCACUCUGAAUUUUAUUUUACCUGUCAUUUCUCGGUAUCAAUAGGUUUUACUUCCCCUUGUUCUGGA
UUUCAGUGAUUGAUGACUGAAUACAAUAAAUAUUUAUUGAGCGCC

>NR_110764.1 Homo sapiens long intergenic non-protein coding RNA 1910 (LINC01910), long non-coding RNA
AGACTGATCTGAGTAATAATAACACTCCAGTCTTCTGCACAGCCAGCTCTGCATGAATTACTCTTTCTCT
ATTGCAATTCCCCTGGCTTGATGAATCAGCTCTGTCTAGGCAGCGGGCAAGAGAAATCCTGAGGCTCAAA
GACACTGAATGACCTGCTGCTTCACACACAGGTGGTCAGTGTTGCCAAAGACCAGGACGAGCAGTCCAGG
CAGCTGTCACATTTCCAACATGTGGAAAGCCTCGCCAAAGCATGAGCTCTGCTGTCTCCCTTCCATGCAG
CGAGGCCACACGAGGCGGACGCAGATTTGTTCCAGCAGCTTCACCAGGGAATAAACAGCAGGCATAGATT
ACACACTCTTTCTATATTACTATGAAGCTTCAAGATATTTTATGCGGCTTTTGTAAGAAACATAATCCTA
CTAACATTGACTGAGTACCAGGCCCTCTGCTAGGAGGCAGGGAAACTGCATACAGCAACTCCAGCACCAT
CCAGCCTCGAGGGCGAGGCAGACTCGTAAGTGGGTGATCATAACAGCGAGTGCTAAATGATATATTGGGA
AAATCACAGAATACTGTCGCAACACCCAGGGTGTGCTCTCACCCATCTTGAGAATGAGGAAGAGTTAACC
CAGAACAAAGAAAGGGTAGTTGATGTTTAGTGTTTGGGCAGATGTATTCCAAGCAAAGGGAACATTATTG
GTAAAAGAAGAAAGGGGATTGAGAGCCCAGAGTCTTCGGAGAGCTGTAAATATTCTGTCAAGGCACAGAA
GCGAAGTTTGGAGTTTATGCTACCGACAATGGGAATCTCTAAAAGGAGTTCAGAAAGATCTGAAAATAGA
CTCAAAGATTTCTTCAACAAAAGGAGATGGGTGTCTGAAAATGATGAAGATGTGGTCCCTGACTCCAAAG
AGCTCATCACCTACTGGGAGAGACAGATATGTAAACAAATCATTACAGAACAATTTGTTTCATTCTAAAT
GTAGCACACAAAAAGAGCTGGGGACAGTGAAGAGGAAGCCGTCAGCGCTGCCTGCGGAGAAGAAAGGAAG
GCTTTGTGTAGGTGACAACTGAGCAAGATGACAGATGAGTGGGCGGAAACCAGTCCTAGCAGAAGGGGTG
GACCCTTGGGGCGGGACCAGGAAGGGCACCTCCAATTCATTACCCAAGATTAAATGAATGGAACATTCGC
CGTACTCTTTAAATTTCAAGAATTTCTTTTCTTTCTTTCTTTCTTTTTTGAGACGGAATTTTGCTCTTGT
CGCCCAGGCTGGAGTGCAATGGCGCAATCTCGGCTCACCGCAACCTCCGCCTCCTGGTTCAAGCTATTCT
TCTGCCTCAGTCTCCCGAGTAGCTGGGATTGCAGGCATGAGCCACCACGCCCGGCTAATTTTGTATTTTT
AGTAGAGATGAGGTTTCTCCATGTTGGTCAGGCTGGTCTCAAACTCCTGAACTCAGGTGATCCGCCCGCC
TTAGTCTCCCAAAGTGCTGGGATTACAGGCGTGAACCACCATGCCCGACCTAAATTTCAAGAATTTCTAT
TGCAACTCCTTTGCCTCAGGAATTTGTCCCCTTCCTAGTGGAGATAGTGAATGTTAAACTGTAACCTACA
TATCTCTTCTCAAGGATTTTGAATGTTTGTTTGTACAGAGAACACTCCATCTGGTATATCCCAGAAACAA
AAGGAGCTTTTCAGTAAGATTTTCAGTGCACGGAAGCCGTGTTCATTTCTAGAGACTGCATTCTCAGAAG
CTTTTCGCCCTAAGCACAAAATCAGAGGCAAGTAGGAGGAGTGGGGCTGCACCTGAGTGCAGCATTGCTG
TACCCATCCTGTTTCCCAGACAAGCAGATTCAAAAATCAAGTGTCTTATTCACTGGGGTTAGAAAGAATC
TATTTTTTTCCTTTGGCAGCATCAAACATCAGAAATAATTCCACTGAAAAGAATATTATACCTAATGTAT
TCTACTTAAAGTCCAACAAATACATTAAGAGCAGAAAATTGTAGGATACAAGATTCTCAAATTTTCAGTA
ATACAGCTCAATATCAGTACACAAGGTGAGGCCTTATGGACTGAAGGGTTGTGTCCACCCAAAATTCATA
TGTTGAAGCCCTAACCCCCCACTGCGATGGTATGAGGAGGTGAAGCCCCTGGAAGATGATTAGGTCATGA
GAGAGAAGCCCTTGTCAATGGGACTAGTGACCTTGCCAAAGGGACCCTAAAGAGCTCTTATGCCCTCCTT
CCTCTTGTGAGTGCACAACTAGCCGGCAAGCCAGAAGAGGACCCTTATCAGAACCTGACATTGCTGCACC
CCAATCTCAGAATTCCAGCCTCCAAAATCGGAGAAATATATTTCTATTGTTTATAAACCACGCTGGGGTA
CTTTGTTGTAGCAGCCAAAACTGACTAAGATGAGAGGCCAAAAAAGGTGCTTTCCCTATTAACAGCACGA
CTCTGCCCGTGCTTACAAAGAACTGTGGTTGAAATGGATTCAGCTTTACAACATCTGCAGAAGGCTAAGT
TACGTGCGGCAGTAGTGGTGGAAATGTTGAACTAGGAACTGTATTAAGGGGTTGAAAGTAAAGGATGAG
CCATAATTTATTCAGGAAAACGCAAATAAAAGTAGTGGAAATAGCAA

RNA
>LINC01910
AGACUGAUCUGAGUAAUAAUAACACUCCAGUCUUCUGCACAGCCAGCUCUGCAUGAAUUACUCUUUCUCU
AUUGCAAUUCCCCUGGCUUGAUGAAUCAGCUCUGUCUAGGCAGCGGGCAAGAGAAAUCCUGAGGCUCAAA
GACACUGAAUGACCUGCUGCUUCACACACAGGUGGUCAGUGUUGCCAAAGACCAGGACGAGCAGUCCAGG
CAGCUGUCACAUUUCCAACAUGUGGAAAGCCUCGCCAAAGCAUGAGCUCUGCUGUCUCCCUUCCAUGCAG
CGAGGCCACACGAGGCGGACGCAGAUUUGUUCCAGCAGCUUCACCAGGGAAUAAACAGCAGGCAUAGAUU
ACACACUCUUUCUAUAUUACUAUGAAGCUUCAAGAUAUUUUAUGCGGCUUUUGUAAGAAACAUAAUCCUA
CUAACAUUGACUGAGUACCAGGCCCUCUGCUAGGAGGCAGGGAAACUGCAUACAGCAACUCCAGCACCAU
CCAGCCUCGAGGGCGAGGCAGACUCGUAAGUGGGUGAUCAUAACAGCGAGUGCUAAAUGAUAUAUUGGGA
AAAUCACAGAAUACUGUCGCAACACCCAGGGUGUGCUCUCACCCAUCUUGAGAAUGAGGAAGAGUUAACC
CAGAACAAAGAAAGGGUAGUUGAUGUUUAGUGUUUGGGCAGAUGUAUUCCAAGCAAAGGGAACAUUAUUG
GUAAAAGAAGAAAGGGGAUUGAGAGCCCAGAGUCUUCGGAGAGCUGUAAAUAUUCUGUCAAGGCACAGAA
GCGAAGUUUGGAGUUUAUGCUACCGACAAUGGGAAUCUCUAAAAGGAGUUCAGAAAGAUCUGAAAAUAGA
CUCAAAGAUUUCUUCAACAAAAGGAGAUGGGUGUCUGAAAAUGAUGAAGAUGUGGUCCCUGACUCCAAAG
AGCUCAUCACCUACUGGGAGAGACAGAUAUGUAAACAAAUCAUUACAGAACAAUUUGUUUCAUUCUAAAU
GUAGCACACAAAAAGAGCUGGGGACAGUGAAGAGGAAGCCGUCAGCGCUGCCUGCGGAGAAGAAAGGAAG
GCUUUGUGUAGGUGACAACUGAGCAAGAUGACAGAUGAGUGGGCGGAAACCAGUCCUAGCAGAAGGGGUG
GACCCUUGGGGCGGGACCAGGAAGGGCACCUCCAAUUCAUUACCCAAGAUUAAAUGAAUGGAACAUUCGC
CGUACUCUUUAAAUUUCAAGAAUUUCUUUUCUUUCUUUCUUUCUUUUUUGAGACGGAAUUUUGCUCUUGU
CGCCCAGGCUGGAGUGCAAUGGCGCAAUCUCGGCUCACCGCAACCUCCGCCUCCUGGUUCAAGCUAUUCU
UCUGCCUCAGUCUCCCGAGUAGCUGGGAUUGCAGGCAUGAGCCACCACGCCCGGCUAAUUUUGUAUUUUU
AGUAGAGAUGAGGUUUCUCCAUGUUGGUCAGGCUGGUCUCAAACUCCUGAACUCAGGUGAUCCGCCCGCC

UUAGUCUCCCAAAGUGCUGGGAUUACAGGCGUGAACCACCAUGCCCGACCUAAAUUUCAAGAAUUUCUAU
UGCAACUCCUUUGCCUCAGGAAUUUGUCCCCUUCCUAGUGGAGAUAGUGAAUGUUAAACUGUAACCUACA
UAUCUCUUCUCAAGGAUUUUGAAUGUUUGUUUGUACAGAGAACACUCCAUCUGGUAUAUCCCAGAAACAA
AAGGAGCUUUUCAGUAAGAUUUUCAGUGCACGGAAGCCGUGUUCAUUUCUAGAGACUGCAUUCUCAGAAG
CUUUUCGCCCUAAGCACAAAAUCAGAGGCAAGUAGGAGGAGUGGGGCUGCACCUGAGUGCAGCAUUGCUG
UACCCAUCCUGUUUCCCAGACAAGCAGAUUCAAAAAUCAAGUGUCUUAUUCACUGGGGUUAGAAAGAAUC
UAUUUUUUUCCUUUGGCAGCAUCAAACAUCAGAAAUAAUUCCACUGAAAAGAAUAUUAUACCUAAUGUAU
UCUACUUAAAGUCCAACAAAUACAUUAAGAGCAGAAAAUUGUAGGAUACAAGAUUCUCAAAUUUUCAGUA
AUACAGCUCAAUAUCAGUACACAAGGUGAGGCCUUAUGGACUGAAGGGUUGUGUCCACCCAAAAUUCAUA
UGUUGAAGCCCUAACCCCCCACUGCGAUGGUAUGAGGAGGUGAAGCCCCUGGAAGAUGAUUAGGUCAUGA
GAGAGAAGCCCUUGUCAAUGGGACUAGUGACCUUGCCAAAGGGACCCUAAAGAGCUCUUAUGCCCUCCUU
CCUCUUGUGAGUGCACAACUAGCCGGCAAGCCAGAAGAGGACCCUUAUCAGAACCUGACAUUGCUGCACC
CCAAUCUCAGAAUUCCAGCCUCCAAAAUCGGAGAAAUAUAUUUCUAUUGUUUAUAAACCACGCUGGGGUA
CUUUGUUGUAGCAGCCAAAACUGACUAAGAUGAGAGGCCAAAAAAGGUGCUUUCCCUAUUAACAGCACGA
CUCUGCCCGUGCUUACAAAGAACUGUGGUUGAAAUGGAUUCAGCUUUACAACAUCUGCAGAAGGCUAAGU
UACGUGCGGCAGUAGUGGUGGAAAUGUUGAACUAGGAACUGUAUUAAGGGGUUGAAAGUAAAGGAUGAG
CCAUAAUUUAUUCAGGAAAACGCAAAUAAAAGUAGUGGAAAUAGCAA

>NR_183524.1 Homo sapiens long intergenic non-protein coding RNA 1912 (LINC01912), long non-coding RNA
GTTAGGATTGAGATGTCTATTTGACCTTCAAATTGAAATGTAAAGTAGGAATTGGATATTATAGTCTGGG
GAATCCAGAGAAGTGCAGGACCAAAATAACAATTTAGGGGTCATTTGTGTACAATAACTAGAAGAAGAAA
ATAACAGAGTTCCCAGAATATATGGCCAGTTGGATATCAAGACTAAAAATATTAAAAATGAAAATATGAT
CAATGGCATCAAGCACCGCTGAAAGGTCAAGCAAATAAGAATTGAGGGCTGACTACAGGTTTTGAACATA
TGGAAGACACTGGTGACCTTAGGGGAGACACAAGAGATAATTTGGAAACAGTGGACAAAAACAATTTTGA
GATATTTTTCTGTAAAGGGAAACAGAGCAATATGATGGTTGTTGGAAAAAGAAGTGGAGATTGAGGTTAT
GGTTATTATTTTTATTATTATTTAGGGAGGCCTGACAAGCTGTTTGTTGACATAAGGGAAAGATACATTA
CAGATGAAGGAGAGATGAAGGAGAGAGAAAAGATAATTGCTAAGAATTATCCTGGACTATGTGAGACAGA
AACAGAATATATGAATGGGAGGTCGATTTTCACTAAATTTACTCACGATTTATTCAGAGTAATAGGAAAA
ATGTCAAATATAAGTGTATCAATATTGAGTGTAATTTTGGTACACTTATACATTTTTCCTAGTACTATTT
CCATAAGTGATAGAAAAAATGTCAAATATAAGTGTATCAATTTGAGTGTAAGAGCTTGTGAGATCTTCAA
TTTTTCGATTTTTATGCGATAGGACTATTTGGAAAAGATTTTGGTGGCTGGGACTGTAGTTGTTTTGACA
TTAATGGTGATGAGGTATAAGATACAGGCATCAAAGTGACAGTTAAGGAGGACTGTTTCCTATTTCCTCA
GTTTAATCTTCTCAGGTGAGTTTGAAGGTGCCAAATAACATAGATTCCAGGATTTGGAAAGGCCACACAG
GTGTACAGTGAAATAATGAAGGATTTAAAAGTTGTGTTGGAGAGAAATAGAGTGAGCACAGACATAAGAT
TTCAAGGAATAAGGAGGAGTAAACCACAGTCAGTGCTACTGGCAACAAGAAAAGATTGACTCATGATATG
AAAGTCCAAGCTGAAGGCGGAGAGGGAAGGCTCTAGAAAAAGCAATGCAGAACGAAGAAGTCACATCCGT
CACTTTCAAGCCTAGGTGTATGAGGTGTGGGGGAGGAAAGCACAGGCATCCCTGAGAAGCCTGTTGGAGA
ATCAGCATCCTCAGGGGAGATTCGAGTTTCAATCGGAGCAAGAAGCATCCCAGAGGCACTTTAACCAAAC
CCTGGAGAGAGAAGAAATAAGTATATTACTGTGTCGTCTTTGCACAGCAAGAACTTAGCATCATCCTCTT
GTATTTTGGATTTTGTTTCCTATTTCCTCAGTTTAATCTTCTCAGCCATAGGTCATAAAGATGGCGATGG
TTATTTCCTTCCTCATACCCATGGTTTGTTACAATGTGACTTTGCGGTTCTTCCCATCAGTAAACAGAAG
CCATTTTCTTACTCCTTGCCTCTGGATGGGCCTTGTGGCTTGCTTTGAGCAATAGAACGCATCAGAAGGT
GACCTCATGCCAGTTTCATGCCTTGGCTTCAGGAGGCCTTGAATTCTCTGCTGCTCCTTAGGAATCTTCT
GAGCCACTGCTTAACAAGCTCCAACCTCTCCAGCTGGAAGATGAGGCATAATACAGAGGAGAGGCAAACT
GTCCTAAAGAGACTACCCTAAGCCAACCAGTCCAGATGACAGGAGGTTGACCTTGACACATGTGTAAGCC
CAGCCAAGGTCAGCCAAACCTAGTACAGATCAGAAAAACCATCTATCAATGCTCAGCCCAAATTTCCAAC
CCACAGAAATATGAATTAAATAAATGATTGTTGTTTAAACCA

RNA
>LINC01912
GUUAGGAUUGAGAUGUCUAUUUGACCUUCAAAUUGAAAUGUAAAGUAGGAAUUGGAUAUUAUAGUCUGGG
GAAUCCAGAGAAGUGCAGGACCAAAAUAACAAUUUAGGGGUCAUUUGUGUACAAUAACUAGAAGAAGAAA
AUAACAGAGUUCCCAGAAUAUAUGGCCAGUUGGAUAUCAAGACUAAAAAUAUUAAAAAUGAAAAUAUGAU
CAAUGGCAUCAAGCACCGCUGAAAGGUCAAGCAAAUAAGAAUUGAGGGCUGACUACAGGUUUUGAACAUA
UGGAAGACACUGGUGACCUUAGGGGAGACACAAGAGAUAAUUUGGAAACAGUGGACAAAAACAAUUUUGA
GAUAUUUUUCUGUAAAGGGAAACAGAGCAAUAUGAUGGUUGUUGGAAAAAGAAGUGGAGAUUGAGGUUAU
GGUUAUUAUUUUUAUUAUUAUUUAGGGAGGCCUGACAAGCUGUUUGUUGACAUAAGGGAAAGAUACAUUA
CAGAUGAAGGAGAGAUGAAGGAGAGAGAAAAGAUAAUUGCUAAGAAUUAUCCUGGACUAUGUGAGACAGA
AACAGAAUAUAUGAAUGGGAGGUCGAUUUUCACUAAAUUUACUCACGAUUUAUUCAGAGUAAUAGGAAAA
AUGUCAAAUAUAAGUGUAUCAAUAUUGAGUGUAAUUUUGGUACACUUAUACAUUUUUCCUAGUACUAUUU
CCAUAAGUGAUAGAAAAAAUGUCAAAUAUAAGUGUAUCAAUUUGAGUGUAAGAGCUUGUGAGAUCUUCAA
UUUUUCGAUUUUUAUGCGAUAGGACUAUUUGGAAAAGAUUUUGGUGGCUGGGACUGUAGUUGUUUUGACA
UUAAUGGUGAUGAGGUAUAAGAUACAGGCAUCAAAGUGACAGUUAAGGAGGACUGUUUCCUAUUUCCUCA
GUUUAAUCUUCUCAGGUGAGUUUGAAGGUGCCAAAUAACAUAGAUUCCAGGAUUUGGAAAGGCCACACAG
GUGUACAGUGAAAUAAUGAAGGAUUUAAAAGUUGUGUUGGAGAGAAAUAGAGUGAGCACAGACAUAAGAU
UUCAAGGAAUAAGGAGGAGUAAACCACAGUCAGUGCUACUGGCAACAAGAAAAGAUUGACUCAUGAUAUG
AAAGUCCAAGCUGAAGGCGGAGAGGGAAGGCUCUAGAAAAAGCAAUGCAGAACGAAGAAGUCACAUCCGU
CACUUUCAAGCCUAGGUGUAUGAGGUGUGGGGGAGGAAAGCACAGGCAUCCCUGAGAAGCCUGUUGGAGA
AUCAGCAUCCUCAGGGGAGAUUCGAGUUUCAAUCGGAGCAAGAAGCAUCCCAGAGGCACUUUAACCAAAC
CCUGGAGAGAGAAGAAAUAAGUAUAUUACUGUGUCGUCUUUGCACAGCAAGAACUUAGCAUCAUCCUCUU
GUAUUUUGGAUUUUGUUUCCUAUUUCCUCAGUUUAAUCUUCUCAGCCAUAGGUCAUAAAGAUGGCGAUGG
UUAUUUCCUUCCUCAUACCCAUGGUUUGUUACAAUGUGACUUUGCGGUUCUUCCCAUCAGUAAACAGAAG
CCAUUUUCUUACUCCUUGCCUCUGGAUGGGCCUUGUGGCUUGCUUUGAGCAAUAGAACGCAUCAGAAGGU
GACCUCAUGCCAGUUUCAUGCCUUGGCUUCAGGAGGCCUUGAAUUCUCUGCUGCUCCUUAGGAAUCUUCU
GAGCCACUGCUUAACAAGCUCCAACCUCUCCAGCUGGAAGAUGAGGCAUAAUACAGAGGAGAGGCAAACU
GUCCUAAAGAGACUACCCUAAGCCAACCAGUCCAGAUGACAGGAGGUUGACCUUGACACAUGUGUAAGCC
CAGCCAAGGUCAGCCAAACCUAGUACAGAUCAGAAAAACCAUCUAUCAAUGCUCAGCCCAAAUUUCCAAC
CCACAGAAAUAUGAAUUAAAUAAAUGAUUGUUGUUUAAACCA

>NR_040033.1 Homo sapiens long intergenic non-protein coding RNA 1915 (LINC01915), long non-coding RNA
AATCACTAATGACCTCATCTTCAAATGGTCAGTGATAATAAACAAGAAGGAGGGATTTCCAAGGGAGCAG
AGCATTAATAAGCTACTTGTGCAAGAAGCTACAATGAGAAAAAAAAATAGCAAGAAATAGATGAGGGAAGG
AGGTTCTGTAGAGGGAAAAACAAAGAGAGAAAAGGAGAATGAATTGTGCTGAGAAAGAGATGCCTGGATG
AAGAAGCTGGCCCTAGCACCCATAAAGCTGGAAAGAAAGTGGGGCCTTGGAAACTTCTGGAAGGATGCGA
GGAAAGAAAAATGCAAGGAAATGAGATGGAGAGGAGAAAGAGAAACCAGGAAGCACTTCGGAAGCATGTA
AATTACAAGGAAATGAGGAACAGCCTTTATTGAAACAGTCTGTGAAGTCTGTGCTAGCGCTGAGAGCTTC
ATTTTACTGTCCTGGCAAGGAGGTGTGGCCCCTCTCCAGTGCCCAGCACCTCATCGCTGTGGTAACAGAA
GACACGCTTATCTTCCGGATTTTGCCTCAGAGACTTGGGCTGTACAAGCTGCTTTCCTGGGGTGGCTTGT
CCTTTTGCAGTTAACCGAGATGCCTGGGGAGAAGAGCTTCCCCCAACAGGATGGCAGGCGATGCTTCCAG
ATGCGGTCACTTGGCACGTCTGAGCCGAATGTCTCCATAAAAATATGATGTAATTAACAGAGCCGCAGCA
GCTGGAACAATTCAGCACCTCACATTGATTTTCCATGTCTCCTATTTACTTTCAGTTTTTAAACTGTCTG
CAGTGACAGGAAATCAAAGAATTATAATGGTGTGTGTGAGGGCCCAATGCTGGGTCGGCATTTCCTTGTG
AAATCCTGAGATTAATCAGTTTCCTATCACTCAATCATCCCTTTTCACTTCTAAAGGAAAACTGTCCTCC
CAAATGCTACGAAATAGCAGTCTATTTTTGGATATTAAAAAAATCAATTGTATTATTTTAAAATTTGAGA
GGAGTGTTTTTAAAAAAAGTCAGCCCGAAGCAATATTCACTTATCTGTTTTCCCATTCAAGTTTAATAGG
AGTTGGCAAAGGCATTTGGGTGCAGTGAGACACAAGTACTATATTCACCCTTCCCAAGTTTTGAGAGCTT
GGAAAAGTTACTTGGTGGAACCAAAGAGAGGAACTGCATTCCAAGTTTTTTTGATCACTGAAATGAAGGA
AAATAAGTTTCCCCATATGTGGTTATTCATGAGCCTTCATCTGAAGCTGGTGGTTTTCTCATAGTTGGTC
ACTGTATTTCCAGCTCTGTCAGGTGAAAGTCAGAATCTTGGGTTAATTTCACTTTGGGTTCCTTAAAATG
CTGGAGAAAATAGGCAGCTGCGGTTGTAGTGGGGAAACAGGCCTCTGTCTGAAGTCAGCTTGCTCTAGAT
TGTGTTTGAAAACATGCCTCAGATTTAAGTGGTCTTGCTTTTTTTCCAGACTCCCTTGCCTTGGCAGATT
CTGTGTCTTGGCCATACTGTAAGAACTCACTTTTCTTTGTCTTTTTATTGATTCAAGCAATATAACCATT
TCATAAATAATTAGGAAAATAAAGAAGAAAATTTCTCATAATTCTACTGCTCAAACGCAATAGCTATGAT
TGCTTTGGAGCAATTGCTTTGGGTCATTTTCAAATGCTGAGTTTTGGTATGATTCTAAGCATAATGTACA
AACAACTTTACATGTTGCTTTTTCACTTAATCTATGTCAGAAGCATCTCCCCAAGTTATGATTAGCTTCA
GTAATTATCTTTTTAATTGCTGCCCAAACAGCTGGAACAATTCAGTCCCTCTTATTGACTTTCCATGTGT
CCTATGGAAGTAGAGACAGTCTATTAACTATTTCTGTATTTTGGGACAATAGATCAAATTCATTTTTCAC
TGTAATTAATCCCATGATAAGTATCTTTATAAATAAGCCTTTTCTATATATAAGATAATTTCTTTAGGAT
AAATTCCTAGAAATTACAGGGTCAAAGTGGATACATATTTTATGACTCTTGATATTTCTTATTGACCTTT
AAAGCTATTATGTGACTTTATATTATCACCTAAAATGTTTGAGGAATTCATGCTTTACGAGCGTATTTTA
TTGACTAAATATGCAGTACTGAGAGAGCTTTATGTGTGCAGTTTGATGATGCTGACAGATATCCCGGAGA
TAAACATTTTTTAATTTAAAGATGTTCTGTTTATGTTTAACATACAGAAGTTAATGTTAAACATGATGCC
TATTTTGTTTAACATACAGAAGTGACTGTATTAAAGCCATTTCTTCTAAATGTGCAATAAACTATGAATT
CTTTGCCTGGT

RNA
>LINC01915
AAUCACUAAUGACCUCAUCUUCAAAUGGUCAGUGAUAAUAAACAAGAAGGAGGGAUUUCCAAGGGAGCAG
AGCAUUAAUAAGCUACUUGUGCAAGAAGCUACAAUGAGAAAAAAAAAUAGCAAGAAAUAGAUGAGGGAAGG
AGGUUCUGUAGAGGGAAAAACAAAGAGAGAAAAGGAGAAUGAAUUGUGCUGAGAAAGAGAUGCCUGGAUG
AAGAAGCUGGCCCUAGCACCCAUAAAGCUGGAAAGAAAGUGGGGCCUUGGAAACUUCUGGAAGGAUGCGA
GGAAAGAAAAAUGCAAGGAAAUGAGAUGGAGAGGAGAAAGAGAAACCAGGAAGCACUUCGGAAGCAUGUA
AAUUACAAGGAAAUGAGGAACAGCCUUUAUUGAAACAGUCUGUGAAGUCUGUGCUAGCGCUGAGAGCUUC
AUUUUACUGUCCUGGCAAGGAGGUGUGGCCCCUCUCCAGUGCCCAGCACCUCAUCGCUGUGGUAACAGAA
GACACGCUUAUCUUCCGGAUUUUGCCUCAGAGACUUGGGCUGUACAAGCUGCUUUCCUGGGGUGGCUUGU
CCUUUUGCAGUUAACCGAGAUGCCUGGGGAGAAGAGCUUCCCCCAACAGGAUGGCAGGCGAUGCUUCCAG
AUGCGGUCACUUGGCACGUCUGAGCCGAAUGUCUCCAUAAAAAUAUGAUGUAAUUAACAGAGCCGCAGCA
GCUGGAACAAUUCAGCACCUCACAUUGAUUUUCCAUGUCUCCUAUUUACUUUCAGUUUUUAAACUGUCUG
CAGUGACAGGAAAUCAAAGAAUUAUAAUGGUGUGUGUGAGGGCCCAAUGCUGGGUCGGCAUUUCCUUGUG
AAAUCCUGAGAUUAAUCAGUUUCCUAUCACUCAAUCAUCCCUUUUCACUUCUAAAGGAAAACUGUCCUCC
CAAAUGCUACGAAAUAGCAGUCUAUUUUUGGAUAUUAAAAAAAUCAAUUGUAUUAUUUUAAAAUUUGAGA
GGAGUGUUUUUAAAAAAAGUCAGCCCGAAGCAAUAUUCACUUAUCUGUUUUCCCAUUCAAGUUUAAUAGG
AGUUGGCAAAGGCAUUUGGGUGCAGUGAGACACAAGUACUAUAUUCACCCUUCCCAAGUUUUGAGAGCUU
GGAAAAGUUACUUGGUGGAACCAAAGAGAGGAACUGCAUUCCAAGUUUUUUUGAUCACUGAAAUGAAGGA
AAAUAAGUUUCCCCAUAUGUGGUUAUUCAUGAGCCUUCAUCUGAAGCUGGUGGUUUUCUCAUAGUUGGUC
ACUGUAUUUCCAGCUCUGUCAGGUGAAAGUCAGAAUCUUGGGUUAAUUUCACUUUGGGUUCCUUAAAAUG
CUGGAGAAAAUAGGCAGCUGCGGUUGUAGUGGGGAAACAGGCCUCUGUCUGAAGUCAGCUUGCUCUAGAU
UGUGUUUGAAAACAUGCCUCAGAUUUAAGUGGUCUUGCUUUUUUUCCAGACUCCCUUGCCUUGGCAGAUU
CUGUGUCUUGGCCAUACUGUAAGAACUCACUUUUCUUUGUCUUUUUAUUGAUUCAAGCAAUAUAACCAUU
UCAUAAAUAAUUAGGAAAAUAAAGAAGAAAAUUUCUCAUAAUUCUACUGCUCAAACGCAAUAGCUAUGAU
UGCUUUGGAGCAAUUGCUUUGGGUCAUUUUCAAAUGCUGAGUUUUGGUAUGAUUCUAAGCAUAAUGUACA
AACAACUUUACAUGUUGCUUUUUCACUUAAUCUAUGUCAGAAGCAUCUCCCCAAGUUAUGAUUAGCUUCA

GUAAUUAUCUUUUUAAUUGCUGCCCAAACAGCUGGAACAAUUCAGUCCCUCUUAUUGACUUUCCAUGUGU
CCUAUGGAAGUAGAGACAGUCUAUUAACUAUUUCUGUAUUUUGGGACAAUAGAUCAAAUUCAUUUUUCAC
UGUAAUUAAUCCCAUGAUAAGUAUCUUUAUAAAUAAGCCUUUUCUAUAUAUAAGAUAAUUUCUUUAGGAU
AAAUUCCUAGAAAUUACAGGGUCAAAGUGGAUACAUAUUUUAUGACUCUUGAUAUUUCUUAUUGACCUUU
AAAGCUAUUAUGUGACUUUAUAUUAUCACCUAAAAUGUUUGAGGAAUUCAUGCUUUACGAGCGUAUUUUA
UUGACUAAAUAUGCAGUACUGAGAGAGCUUUAUGUGUGCAGUUUGAUGAUGCUGACAGAUAUCCCGGAGA
UAAACAUUUUUUAAUUUAAAGAUGUUCUGUUUAUGUUUAACAUACAGAAGUUAAUGUUAAACAUGAUGCC
UAUUUUGUUUAACAUACAGAAGUGACUGUAUUAAAGCCAUUUCUUCUAAAUGUGCAAUAAACUAUGAAUU
CUUUGCCUGGU

>XR_001753544.3 PREDICTED: Homo sapiens long intergenic non-protein coding RNA 1916 (LINC01916), transcript variant X1, ncRNA
ATACTTCTTCCAGCTGCCCTGTGAAGAAAGACGTGTTTGCTTTTCCTTCAGCCATGATTAGTCCTGTGAT
GTTATGAGAAAAATGATTTGAAAATGAGCTTGAGGTTTCAGCCCTGGAGGCCTGAACAGACGAGCTGGCT
GTGGCTAGTCACTCCCCCACCAGCCTTGGCCGTGCACTCATGCCATTCAGCAGATTTGATGAAGCCAGCT
TTCATGTTACGAGCTACCTCTGTGGAGAAGCCCACATGTCAAGAGAGTGGATGACCCTCTTGCCCACAGC
CAGTGAGGAATGGAGGCCCTGAGGCCAATGGGCTACAAAGAACTAAATCCTGTCAACATTCATATGCATG
ACCTTGGAATCAGATCCCACCCCCATCAAGGCCTGACATGACTGCAATCCCAGCTGACATTTTGATTGTA
GTCATGTAAGAGTCCCAGAGGACCCAGTTAAGCTAGGCACAGTCTCTTAATGCGGGGAAATTGTGAAGTA
ATTAGTACTGTTGTTTTGGGATAATGTGTTACGTAACAAGAGACAGGTGATACAACATCTCTGAGATTCT
CAACATGTTTGGTAAAAATAATTAAACTGCTTTTAATGTATATGTCATGATTAATGTTATCTATAATGAG
TGTATTAGGGATACATTAATATTCACAAAGACTATATAGCTATTAACTAATTGTTCTGTGGCTTATAGAC
ATAATTTTGGATGATTCGTTCTTTTTTTGCCACTAATAAATATACAAAAACCTTTCATGTTTTAAAATGA
ATAAAAAGTTATAATTCTGAAGAGGTCCTTTATTTAAATGAAACACACTAAAACCATTGAATTTTAATAT
TTATATTATGAAATGATCTGCCACTGTTTTTTGTAAGTGTACCTGCTATTATGACTATATTTTCAAGTTA
AAGATTCATGACGTTTTTGTGAGAAAATATTTTAAAAACTTGTTAATTTTTTCAGATTAACTACAAAAAA
TCTATTGTAAAAATTTATAAAATACTAATAATTAAGTCTCTTTATTTTCATGTCAGTGCTCTTAAAATAT
AGGTTGTTTCAAAGACAGTAGCAATAAAAATGTCAATTCAGAATTCCTTCAGGGATGACCCTAAGTCTAA
CAGGTATATCCTTTGAAACAAGAAAAATATCTAGCACCTTGGGAAATGAAAATAGTACATTTTTATTTTC
TCTTAATAAAATTAACTCAGATTGTCAATTGTTTATTGTTTCTGTTTGATACCATTGAGGTATTTGTTCC
AGAGATAAAAATATGTA

RNA
>LINC01916
AUACUUCUUCCAGCUGCCCUGUGAAGAAAGACGUGUUUGCUUUUCCUUCAGCCAUGAUUAGUCCUGUGAU
GUUAUGAGAAAAAUGAUUUGAAAAUGAGCUUGAGGUUUCAGCCCUGGAGGCCUGAACAGACGAGCUGGCU
GUGGCUAGUCACUCCCCCACCAGCCUUGGCCGUGCACUCAUGCCAUUCAGCAGAUUUGAUGAAGCCAGCU
UUCAUGUUACGAGCUACCUCUGUGGAGAAGCCCACAUGUCAAGAGAGUGGAUGACCCUCUUGCCCACAGC
CAGUGAGGAAUGGAGGCCCUGAGGCCAAUGGGCUACAAAGAACUAAAUCCUGUCAACAUUCAUAUGCAUG
ACCUUGGAAUCAGAUCCCACCCCCAUCAAGGCCUGACAUGACUGCAAUCCCAGCUGACAUUUUGAUUGUA
GUCAUGUAAGAGUCCCAGAGGACCCAGUUAAGCUAGGCACAGUCUCUUAAUGCGGGGAAAUUGUGAAGUA
AUUAGUACUGUUGUUUUGGGAUAAUGUGUUACGUAACAAGAGACAGGUGAUACAACAUCUCUGAGAUUCU
CAACAUGUUUGGUAAAAAUAAUUAAACUGCUUUUAAUGUAUAUGUCAUGAUUAAUGUUAUCUAUAAUGAG
UGUAUUAGGGAUACAUUAAUAUUCACAAAGACUAUAUAGCUAUUAACUAAUUGUUCUGUGGCUUAUAGAC
AUAAUUUUGGAUGAUUCGUUCUUUUUUUGCCACUAAUAAAUAUACAAAAACCUUUCAUGUUUUAAAAUGA
AUAAAAAGUUAUAAUUCUGAAGAGGUCCUUUAUUUAAAUGAAACACACUAAAACCAUUGAAUUUUAAUAU
UUAUAUUAUGAAAUGAUCUGCCACUGUUUUUUGUAAGUGUACCUGCUAUUAUGACUAUAUUUUCAAGUUA
AAGAUUCAUGACGUUUUUGUGAGAAAAUAUUUUAAAAACUUGUUAAUUUUUUCAGAUUAACUACAAAAAA
UCUAUUGUAAAAAUUUAUAAAAUACUAAUAAUUAAGUCUCUUUAUUUUCAUGUCAGUGCUCUUAAAAUAU
AGGUUGUUUCAAAGACAGUAGCAAUAAAAAUGUCAAUUCAGAAUUCCUUCAGGGAUGACCCUAAGUCUAA
CAGGUAUAUCCUUUGAAACAAGAAAAAUAUCUAGCACCUUGGGAAAUGAAAAUAGUACAUUUUUAUUUUC
UCUUAAUAAAAUUAACUCAGAUUGUCAAUUGUUUAUUGUUUCUGUUUGAUACCAUUGAGGUAUUUGUUCC
AGAGAUAAAAAUAUGUA

>NR_110800.1 Homo sapiens long intergenic non-protein coding RNA 1917 (LINC01917), long non-coding RNA
TCAAGGTTCTATCAGTGATAAATAAGGGAGGCTAAGGTATTGAGGAACCATATAATGTTATTTACACTAC
CTACTTTCTAATCTGCCTCATCTACTCACCTTCCTCTTTCTCTTCCTGTAATCTTATTGCATGGAAGAAA
TTTTTGGTGAAGACAAGTTGTCTGTTTTTCTTAAAATCATTTTTTTAGCTAGTGGTATGTCTCGAACACT
TTCCTAATAGTTCATTGAGATAAAGTCATAACATTTTTTTTTTCTTTCTTCGCTACCCTCACTCATTGCC
CCATTGGAATTGACTGCAAATTGCAATTTTCTAGAGAAAGAATCAGTAATCCTTTCTACACAGTGGCCTT
GGTGATTTCAAAACTTTAAGAATATTTAAAATATTCAAGTTCTTTCTCCAAAAACTGGTGACTTTCTGCT
GCTGAGCTCCTGAAAGTTTTTGAGAAGAAAGTTGAAAGGTTGAAAATGGTGCTGTGTTTATTTGAATATC
TGAAGGGTAAACTCCAAGCTACTTATCTGAAAACAAATATTCTCTGAAACCTGCAGAGTAAAGAAGTATA
ACTTGACATTTCTTCTGCAAGAAAAAAACAAGAAGATGGTGACTTTCAAGACCTCAGTCAATGACTAAAG
TTCTGTGACCACAGTGAGCATATTACTGCGGTAACCAGGGCAGTAATGTCAAGACAAAGGTCAGGGTTGG
CTTTAAGAAAGAAGCCTGAGAACCGGAACTACATAGAGGCCAGAAAATCAGTGTCTACATGCTAAAGGCA
ATTTTCAGGAACAAGTACCAGGAGCAGATCAACAAAACAAAGAAGATCAACAAAACAAATAAGGAAGGTG
GTTTTATGAACCCAGAGGGGCAGTGGTTATGAGTTGTGGTGCCACTTTATTCAACCTTATCTGCTTGGAA
GTTTTTCTCCTACACCATTAAACCCCTCACTGAACCAAACTTTGTACAGACTGCAGCCAAGAAGGGCTGT
ATTATGGCTTTCTTGGACCTCTTCATCCACAAAAATATTAAAAATTGTATTTCATGA

RNA
>LINC01917
UCAAGGUUCUAUCAGUGAUAAAUAAGGGAGGCUAAGGUAUUGAGGAACCAUAUAAUGUUAUUUACACUAC
CUACUUUCUAAUCUGCCUCAUCUACUCACCUUCCUCUUUCUCUUCCUGUAAUCUUAUUGCAUGGAAGAAA
UUUUUGGUGAAGACAAGUUGUCUGUUUUUCUUAAAAUCAUUUUUUUAGCUAGUGGUAUGUCUCGAACACU
UUCCUAAUAGUUCAUUGAGAUAAAGUCAUAACAUUUUUUUUUUCUUUCUUCGCUACCCUCACUCAUUGCC
CCAUUGGAAUUGACUGCAAAUUGCAAUUUUCUAGAGAAAGAAUCAGUAAUCCUUUCUACACAGUGGCCUU
GGUGAUUUCAAAACUUUAAGAAUAUUUAAAAUAUUCAAGUUCUUUCUCCAAAAACUGGUGACUUUCUGCU
GCUGAGCUCCUGAAAGUUUUUGAGAAGAAAGUUGAAAGGUUGAAAAUGGUGCUGUGUUUAUUUGAAUAUC
UGAAGGGUAAACUCCAAGCUACUUAUCUGAAAACAAAUAUUCUCUGAAACCUGCAGAGUAAAGAAGUAUA
ACUUGACAUUUCUUCUGCAAGAAAAAAACAAGAAGAUGGUGACUUUCAAGACCUCAGUCAAUGACUAAAG
UUCUGUGACCACAGUGAGCAUAUUACUGCGGUAACCAGGGCAGUAAUGUCAAGACAAAGGUCAGGGUUGG
CUUUAAGAAAGAAGCCUGAGAACCGGAACUACAUAGAGGCCAGAAAAUCAGUGUCUACAUGCUAAAGGCA
AUUUUCAGGAACAAGUACCAGGAGCAGAUCAACAAAACAAAGAAGAUCAACAAAACAAAUAAGGAAGGUG
GUUUUAUGAACCCAGAGGGGCAGUGGUUAUGAGUUGUGGUGCCACUUUAUUCAACCUUAUCUGCUUGGAA
GUUUUUCUCCUACACCAUUAAACCCCUCACUGAACCAAACUUUGUACAGACUGCAGCCAAGAAGGGCUGU
AUUAUGGCUUUCUUGGACCUCUUCAUCCACAAAAAUAUUAAAAAUUGUAUUUCAUGA

>NR_110798.1 Homo sapiens long intergenic non-protein coding RNA 1919 (LINC01919), long non-coding RNA
GGCTCTCAAGCAAGGTCCACTTGCAGTGAAATAAACCAGAGGTTTGAATCATGGCTTGATACGAAATTAT
TGGAAAGAATATGGCGATGACATCCACAATGCCGGATAAAGAAAATATTTAGAAGATGAGATTCCTGTGT
TTCTGGCTTTCATAACTTTTTAGATTTCCTTTCTACTGGAAAACTTTTAACTTTGAAAACCGCAAGTAAT
ATTTTTCTTCCTGAAACCTCTGCAGTGGTAGCAATGCACTGAAGTTTGATTAAAAGTTTACTGGTCATTC
CTGCCATGAGAAGATGATAGAGAATATCACCACAGTGTGACTTGCTTCCAAGGCAGCATGGTTTCATGCA
GAGGATCTAAACTGGGGACCATGTGCTGGGCTGCAAAAGGGCTGTGCAGTGCCTAAAATGTTATGCAAAA
TTTTATAAAGGTAGGTCATGTATTGCTCCATTGATGCTGCCCTTATTCATGATGATTAAAGCCTTTGGGA
GGTATTCCTTCAGAATAATGGACAACTTCGATAAGTACTGGTAAGAAAATAAGTAGAAACTCTAAATTTG
AAGTCTTC

RNA
>LINC01919
GGCUCUCAAGCAAGGUCCACUUGCAGUGAAAUAAACCAGAGGUUUGAAUCAUGGCUUGAUACGAAAUUAU
UGGAAAGAAUAUGGCGAUGACAUCCACAAUGCCGGAUAAAGAAAAUAUUUAGAAGAUGAGAUUCCUGUGU
UUCUGGCUUUCAUAACUUUUUAGAUUUCCUUUCUACUGGAAAACUUUUAACUUUGAAAACCGCAAGUAAU
AUUUUUCUUCCUGAAACCUCUGCAGUGGUAGCAAUGCACUGAAGUUUGAUUAAAAGUUUACUGGUCAUUC
CUGCCAUGAGAAGAUGAUAGAGAAUAUCACCACAGUGUGACUUGCUUCCAAGGCAGCAUGGUUUCAUGCA
GAGGAUCUAAACUGGGGACCAUGUGCUGGGCUGCAAAAGGGCUGUGCAGUGCCUAAAAUGUUAUGCAAAA
UUUUAUAAAGGUAGGUCAUGUAUUGCUCCAUUGAUGCUGCCCUUAUUCAUGAUGAUUAAAGCCUUUGGGA
GGUAUUCCUUCAGAAUAAUGGACAACUUCGAUAAGUACUGGUAAGAAAAUAAGUAGAAACUCUAAAUUUG
AAGUCUUC

>NR_131977.1 Homo sapiens long intergenic non-protein coding RNA 1922 (LINC01922), long non-coding RNA
GCCTCCCGGGTTCACGCCATTCTCCTGCCTCAGCGTCCTGAGTAGCTGGGACCACAGGTGCCCGCCACCA
CGCCTGCTCAATGCGATGGAATACATTCATGCCATTGTGCAACCTTCACCACCGCCCATCTCCAGAACTC
TCTTCATCCTGCAAAACTGAAACTCGGCCCCGGGTGCCCATCCCCCTCCCCGCCCTGGCATCCACCCTCT
ACTTTCTGTCTCTCTGATCTTGACTGGAAGTAGGTATGTACTTAGTCCGCATTTTAAAGTGCAGAGCCTG
GGACCCAGTGAGACGGAGTCACGTGCCTGTGGTCACTCGATAGAAAATGGCAAAGCTGGGCATCCCACCC
GAGGTCTGTGTCCACAGCCCCTGCCTTGGCGGCCACTACACTGTGGCTTCTTTCAAGTGAGTGCTGAGGA
GGAAAGGGGATTACGCTGCACAACCTCAGTGGTGAGATGAGATGAACGGGTGGAAACTAAACGTAAGGAA
TGTAAGGGATGGGTGAAAAGACGCCAGTGAGCCTCCTGGAAGGGCAGCAAGTTCCCAGACATTCGAAGAA
TCAAGGTCCTGAAAGGATGACCCAGCAGGGACAGGGCAGAGGGGACCCTGCCACAGCTGAGCGATGGCCT
AGATGCTCGTTCTGGGTGCTGTCCCTTCTGGGACTCTAGCGAAACTGTTTTCCTCTCTATTCAGAGTCTG
CCATTCATTGGACTATAATGCCCCATTTCATCTGATTGATGACCCAAATAGCTTCAAGTAGCTCTCCGCG
GGTGGCCAGCCGCCCTGGAGGGTGTCCAGTTACAGGAGCTGAGCACACTGGACGCCTTTTCTCTTCCACA
GTGGGCAATGCACGGTGGGTCCCCCCTTGTCAGGATTAATGAGAGCTTTGGTTGCAGGGCCCCCCCGCCT
GTTGCCACGTGACAGTCTCACAAGCTCACCCGGGAAGAGAGGAGAGAAGCCCATCAGGTCACTGAAGTGG
GCAGTAGAACCCAGGAGAGGAAGAAAGAAAGGAGAGAGGGGGGAAGTGGATGCAGAGACAGAGCCATTGA
AAGTGTTTTCCAACAATGGACTTGGATCGGCTTCCCCTGCCCCTGCAGCAGGGAGCTCGCTGTGCCTGCA
CTGCTGCAGTACTGACAAATTCATCCCTGTCCAAGGAGAGTGAAGTGAAGGTCCGTGAGGCGAGACCTGC
TGGCACTCAGGGTACGTGGGGCCTGGCCCCATGTCCAAGTGCTCCCTCCTTACTGAGGAGTTGGGTTGCC
AGGTGCAGCGGCAGTTTCTCTAAGCCACGTTCAGAGCTGAGTTACTGGGTTCTGTGTGAGAAGGGAGCCC
ACGCCCGAGCCTGTGGAACAGGTCTCGGCTGGAGCAGGTGGTCTGTGCCCCTCCTCACCCCATCTGTCCT
GGCTTGCTCTCACCTCGGCTTCCTGGCCTTCCCAGGGCCCTGGTCTCATCCTCACCCTAAAGCAGGCCCC
TCCACCTGAGGCGCCTTCGCACTGAGATGCCCGCTGATCTTCCCAGGACCCTCATCTCCGTGGCTCTAAG
CTGTTCACCTTCACCCTGGACCCCCAGGAGGGCAGTGGATGAAGGACAAAGAGCAACAACTCTGCTGCCA
GCACCAAAAAGCCCTCTGCTGACTCCCTCAAGTCCTCTTTTGCAGATACCTACAAAGGGCCTTGAAAGAA
AAAAGAACTAGAATTACGAACAAAAAAAAAAAAA

RNA
>LINC01922
GCCUCCCGGGUUCACGCCAUUCUCCUGCCUCAGCGUCCUGAGUAGCUGGGACCACAGGUGCCCGCCACCA
CGCCUGCUCAAUGCGAUGGAAUACAUUCAUGCCAUUGUGCAACCUUCACCACCGCCCAUCUCCAGAACUC
UCUUCAUCCUGCAAAACUGAAACUCGGCCCCGGGUGCCCAUCCCCCUCCCCGCCCUGGCAUCCACCCUCU
ACUUUCUGUCUCUCUGAUCUUGACUGGAAGUAGGUAUGUACUUAGUCCGCAUUUUAAAGUGCAGAGCCUG
GGACCCAGUGAGACGGAGUCACGUGCCUGUGGUCACUCGAUAGAAAAUGGCAAAGCUGGGCAUCCCACCC
GAGGUCUGUGUCCACAGCCCCUGCCUUGGCGGCCACUACACUGUGGCUUCUUUCAAGUGAGUGCUGAGGA
GGAAAGGGGAUUACGCUGCACAACCUCAGUGGUGAGAUGAGAUGAACGGGUGGAAACUAAACGUAAGGAA
UGUAAGGGAUGGGUGAAAAGACGCCAGUGAGCCUCCUGGAAGGGCAGCAAGUUCCCAGACAUUCGAAGAA
UCAAGGUCCUGAAAGGAUGACCCAGCAGGGACAGGGCAGAGGGGACCCUGCCACAGCUGAGCGAUGGCCU
AGAUGCUCGUUCUGGGUGCUGUCCCUUCUGGGACUCUAGCGAAACUGUUUUCCUCUCUAUUCAGAGUCUG
CCAUUCAUUGGACUAUAAUGCCCCAUUUCAUCUGAUUGAUGACCCAAAUAGCUUCAAGUAGCUCUCCGCG
GGUGGCCAGCCGCCCUGGAGGGUGUCCAGUUACAGGAGCUGAGCACACUGGACGCCUUUUCUCUUCCACA
GUGGGCAAUGCACGGUGGGUCCCCCCUUGUCAGGAUUAAUGAGAGCUUUGGUUGCAGGGCCCCCCCGCCU
GUUGCCACGUGACAGUCUCACAAGCUCACCCGGGAAGAGAGGAGAGAAGCCCAUCAGGUCACUGAAGUGG
GCAGUAGAACCCAGGAGAGGAAGAAAGAAAGGAGAGAGGGGGGAAGUGGAUGCAGAGACAGAGCCAUUGA
AAGUGUUUUCCAACAAUGGACUUGGAUCGGCUUCCCCUGCCCCUGCAGCAGGGAGCUCGCUGUGCCUGCA
CUGCUGCAGUACUGACAAAUUCAUCCCUGUCCAAGGAGAGUGAAGUGAAGGUCCGUGAGGCGAGACCUGC
UGGCACUCAGGGUACGUGGGGCCUGGCCCCAUGUCCAAGUGCUCCCUCCUUACUGAGGAGUUGGGUUGCC
AGGUGCAGCGGCAGUUUCUCUAAGCCACGUUCAGAGCUGAGUUACUGGGUUCUGUGUGAGAAGGGAGCCC
ACGCCCGAGCCUGUGGAACAGGUCUCGGCUGGAGCAGGUGGUCUGUGCCCCUCCUCACCCCAUCUGUCCU
GGCUUGCUCUCACCUCGGCUUCCUGGCCUUCCCAGGGCCCUGGUCUCAUCCUCACCCUAAAGCAGGCCCC
UCCACCUGAGGCGCCUUCGCACUGAGAUGCCCGCUGAUCUUCCCAGGACCCUCAUCUCCGUGGCUCUAAG
CUGUUCACCUUCACCCUGGACCCCCAGGAGGGCAGUGGAUGAAGGACAAAGAGCAACAACUCUGCUGCCA
GCACCAAAAAGCCCUCUGCUGACUCCCUCAAGUCCUCUUUUGCAGAUACCUACAAAGGGCCUUGAAAGAA
AAAAGAACUAGAAUUACGAACAAAAAAAAAAAAA

>NR_033881.1 Homo sapiens long intergenic non-protein coding RNA 1924 (LINC01924), long non-coding RNA
GGATATAGGTGGTACCTACATGGGAACACATTGTCTAATACCACCCAGAATGGGTAGCAAAGTTCATAGT
GAAGTTTCCACTGTGAGCGGCAGGTGGCGGGCTCTTCTGAGAAAGAAGCTTCTAGATGCATATTGTGGGG
AGCAGATGAAACTCTGAGGCTTTCAGGGACGTGAGGTACGGTGCCAAGGCCACACCACTGAGACAGGGGT
CTCACTATGTTGCCCAAACTGGCCTTGATCTTCTGGGCTCAAGCAATCTTCCCACCTCAGCCTCCGAGTC
GCTGGGATTGCAGATAAGGAAATTGAGGCTCAGTGTTGGCAGGTAACATGTTCAAGATCTTGAAACTAGC
AGGTGGCAGAGCTGGGGTTCATCTGTCTCCATGTGCTCAGAATTCTGAGAGGAATTGCTGAAAAGGATGG
GGACTCTGAGCTACATATGCATAGCATTGATTTCAGTGAGAAAACTACATATTTTTGACCATATGAAAAA
AAATCTGTTCTTGAATTTTGATGATATTTGCATAGTTTTTAAATTACCTCTGGCAGAAAAATTATGTCAA
AGATGAACCTAAACAAAAGGGAATGACATGTTGTTTGGATTACTGAGCCAGAGAAGCCGCTGGACCATTG
AGATTTAGTAGGCAATATGTTTGCCAGCCTTTTTGAAAATTGGAATGTTATTTTTGAACTTTATCGAAGA
ACATGCTGCATCTCTAAGGCCTGCAAGATTATTCTGTGCCCATCCCATTTATGGACTGCAAACTTACTTG
ATTCCTGATAGCAGAGTTGAGATATAGGGTTCATCCATGTTGCTGCATGTGTCAGAATTTCTTTCCGTCT
TCAGGCTGAATAGTACTCCGTTGTATGTATATACCACATTTGGTCTATTCATTTGTCTGTCGATGGACAC
GTAGACTGCTTCTTCCTTTTGGCAACTGTGATAACCCTGCTATGGACATGGATGTGCAAATAACAAGTAG
CCCCTGTGTCTGCTGTTCTCCTCTATGTGTTCTTGTGAAGAGAATGCTTCTCTAGCACGAGTTCAATATG
ATCTGGGAATTTGAACTGGATCGGCTAAATACTTTATCAGTAGGAAGAAAACTCTCTTTACAGACTGAAA
ATCATTCTGCACAGAAGCACATATGCGCATACTCACACACACACAGATGCCTTCAAGTGAGGATGCCTGA
AGTAATTAAAGGTAATGTGTATCAAGCGTTAATTACAAAATCACTGTGCTCTTCTGAATTGTACACAATT
ATCAGAGAAGCAGCACAAATATCATCATGCCACAAGGCTGCATTTCCTACCATGAGCTTTAACAATTTAT
TTTCAGAACTTCACTTATCCATTAGTTATGCATGCATCTCTACACATTCCCTATATGCCTGGATTATTAG
GTATACGCCATATCATGAAGGCTGTGAAAGTGATTACAAGAAAGTCAAAGATACCCAGATAGGATCTTTC
CTCAGAACCATTTACTTATGCACATTCACCCACATTTGTAAACATAATATTGCAGCAAAAGGCTTGGGCT
CCAGTCATTCAGAGTAGCCACACTGCCCAGGAATATATAACTTTATGTACTTTTTTTTTTTATCATGGAA
CATAAGGGACTATTCATTCTGTAAATCCAGATCTTGTAATTTCAGTGTCTGATATAAGTCAGACTACTGG
AAAGAAAGAAATTTAATTAAAATTAGAAAGGAAAAAAAAAAA

RNA
>LINC01924
GGAUAUAGGUGGUACCUACAUGGGAACACAUUGUCUAAUACCACCCAGAAUGGGUAGCAAAGUUCAUAGU
GAAGUUUCCACUGUGAGCGGCAGGUGGCGGGCUCUUCUGAGAAAGAAGCUUCUAGAUGCAUAUUGUGGGG
AGCAGAUGAAACUCUGAGGCUUUCAGGGACGUGAGGUACGGUGCCAAGGCCACACCACUGAGACAGGGGU
CUCACUAUGUUGCCCAAACUGGCCUUGAUCUUCUGGGCUCAAGCAAUCUUCCCACCUCAGCCUCCGAGUC
GCUGGGAUUGCAGAUAAGGAAAUUGAGGCUCAGUGUUGGCAGGUAACAUGUUCAAGAUCUUGAAACUAGC
AGGUGGCAGAGCUGGGGUUCAUCUGUCUCCAUGUGCUCAGAAUUCUGAGAGGAAUUGCUGAAAAGGAUGG
GGACUCUGAGCUACAUAUGCAUAGCAUUGAUUUCAGUGAGAAAACUACAUAUUUUUGACCAUAUGAAAAA
AAAUCUGUUCUUGAAUUUUGAUGAUAUUUGCAUAGUUUUUAAAUUACCUCUGGCAGAAAAAUUAUGUCAA
AGAUGAACCUAAACAAAAGGGAAUGACAUGUUGUUUGGAUUACUGAGCCAGAGAAGCCGCUGGACCAUUG
AGAUUUAGUAGGCAAUAUGUUUGCCAGCCUUUUUGAAAAUUGGAAUGUUAUUUUUGAACUUUAUCGAAGA
ACAUGCUGCAUCUCUAAGGCCUGCAAGAUUAUUCUGUGCCCAUCCCAUUUAUGGACUGCAAACUUACUUG
AUUCCUGAUAGCAGAGUUGAGAUAUAGGGUUCAUCCAUGUUGCUGCAUGUGUCAGAAUUUCUUUCCGUCU
UCAGGCUGAAUAGUACUCCGUUGUAUGUAUAUACCACAUUUGGUCUAUUCAUUUGUCUGUCGAUGGACAC
GUAGACUGCUUCUUCCUUUUGGCAACUGUGAUAACCCUGCUAUGGACAUGGAUGUGCAAAUAACAAGUAG
CCCCUGUGUCUGCUGUUCUCCUCUAUGUGUUCUUGUGAAGAGAAUGCUUCUCUAGCACGAGUUCAAUAUG
AUCUGGGAAUUUGAACUGGAUCGGCUAAAUACUUUAUCAGUAGGAAGAAAACUCUCUUUACAGACUGAAA
AUCAUUCUGCACAGAAGCACAUAUGCGCAUACUCACACACACACAGAUGCCUUCAAGUGAGGAUGCCUGA
AGUAAUUAAAGGUAAUGUGUAUCAAGCGUUAAUUACAAAAUCACUGUGCUCUUCUGAAUUGUACACAAUU
AUCAGAGAAGCAGCACAAAUAUCAUCAUGCCACAAGGCUGCAUUUCCUACCAUGAGCUUUAACAAUUUAU
UUUCAGAACUUCACUUAUCCAUUAGUUAUGCAUGCAUCUCUACACAUUCCCUAUAUGCCUGGAUUAUUAG
GUAUACGCCAUAUCAUGAAGGCUGUGAAAGUGAUUACAAGAAAGUCAAAGAUACCCAGAUAGGAUCUUUC
CUCAGAACCAUUUACUUAUGCACAUUCACCCACAUUUGUAAACAUAAUAUUGCAGCAAAAGGCUUGGGCU
CCAGUCAUUCAGAGUAGCCACACUGCCCAGGAAUAUAUAACUUUAUGUACUUUUUUUUUUUAUCAUGGAA
CAUAAGGGACUAUUCAUUCUGUAAAUCCAGAUCUUGUAAUUUCAGUGUCUGAUAUAAGUCAGACUACUGG
AAAGAAAGAAAUUUAAUUAAAAUUAGAAAGGAAAAAAAAAAA

>NR_136505.1 Homo sapiens long intergenic non-protein coding RNA 1925 (LINC01925), long non-coding RNA
GGGCGCGTGGCCGAGGTCCGAGGAGGGCCCGGAGGTCTGGCCGAGGTCCGAGGAGGGGCCCGGAGGTCTG
GCCGAGGTCCGAGGAGGGGCCCGGAGGTCTGGCCGAGGTCCGAGGAGGGGCCCGGAGGTCTGGCCGAGGT
CCGAGGAGGGGCCCGGAGGTCTGGCCGAGGTCCGAGGAGGGGTCCGGAGGTCTGGCCGAGGTCCGAGGAG
GGCCCGGAGGTCTGGCCGAGGTCCGAGGAGGGCCCGGAGGTCTGGCCGAGGTGATAATAGTTAAGCCTAG
AGCCATGAGAGGCCTGTGCTCCGTGGGACAGGAGGCTAAGAGCGGAACAGGGCCCACTGTTGGATGCTAC
TGATCACCAGACTGTGGCCTTTATACACATCAACTTGCTTCATCTCCACCAGAACAAATGAATGACAAAA
CCCGATGAAAGACAAAGCCAGAGTAAATCCCTTCTCATCTATGTCACGGCAAAAGATATAGTAACAGCAA
AAACAAGCTC

RNA
>LINC01925
GGGCGCGUGGCCGAGGUCCGAGGAGGGCCCGGAGGUCUGGCCGAGGUCCGAGGAGGGGCCCGGAGGUCUG
GCCGAGGUCCGAGGAGGGGCCCGGAGGUCUGGCCGAGGUCCGAGGAGGGGCCCGGAGGUCUGGCCGAGGU
CCGAGGAGGGGCCCGGAGGUCUGGCCGAGGUCCGAGGAGGGGUCCGGAGGUCUGGCCGAGGUCCGAGGAG
GGCCCGGAGGUCUGGCCGAGGUCCGAGGAGGGCCCGGAGGUCUGGCCGAGGUGAUAAUAGUUAAGCCUAG
AGCCAUGAGAGGCCUGUGCUCCGUGGGACAGGAGGCUAAGAGCGGAACAGGGCCCACUGUUGGAUGCUAC
UGAUCACCAGACUGUGGCCUUUAUACACAUCAACUUGCUUCAUCUCCACCAGAACAAAUGAAUGACAAAA
CCCGAUGAAAGACAAAGCCAGAGUAAAUCCCUUCUCAUCUAUGUCACGGCAAAAGAUAUAGUAACAGCAA
AAACAAGCUC

>NR_146904.1 Homo sapiens long intergenic non-protein coding RNA 1926 (LINC01926), long non-coding RNA
CATCACAGTCCCAAGAATAACACAAGAAATTCTCCAAGTTTCCAAGAAGGGAAGATGGAGTGGAGGGCGC
CCACCCTATCTCCAGTGGACATCCCCTGGGCTACTCCTCTCCTGATGAGAAAATGCTCACTTCAGCCAAG
TGCAGAATGGTCCCCTTTGTTTTCGTGGGTATCAGTGGAGAATGAGATCCTTCATATGGAATCTCCCTGT
GTTGCTCAGGCTGATCTCGAACTCCTGAGCCCAAGCATGCATCAGCCTCTCAGTAGCTGTGATTACAGGC
ATTGAGTCACCACTCCTGAGAAATAACAGGACTTTGGTGCTATTCTCCAAGCTGAAAGCCATCCTCTTCG
GCCCTGAGGACACAGCTTTTTCTGCCATAACTCACTGTCTCCACTGGCTCAATTAGCCAATTAGTGGCAT
TGTAGAGGGCTGAGATGTTACTTTTTAGTTTCCAATAAAATTCTCAAGTTGGTTTCAAAAAAAAAAAAAA
A

RNA
>LINC01926
CAUCACAGUCCCAAGAAUAACACAAGAAAUUCUCCAAGUUUCCAAGAAGGGAAGAUGGAGUGGAGGGCGC
CCACCCUAUCUCCAGUGGACAUCCCCUGGGCUACUCCUCUCCUGAUGAGAAAAUGCUCACUUCAGCCAAG
UGCAGAAUGGUCCCCUUUGUUUUCGUGGGUAUCAGUGGAGAAUGAGAUCCUUCAUAUGGAAUCUCCCUGU
GUUGCUCAGGCUGAUCUCGAACUCCUGAGCCCAAGCAUGCAUCAGCCUCUCAGUAGCUGUGAUUACAGGC
AUUGAGUCACCACUCCUGAGAAAUAACAGGACUUUGGUGCUAUUCUCCAAGCUGAAAGCCAUCCUCUUCG
GCCCUGAGGACACAGCUUUUUCUGCCAUAACUCACUGUCUCCACUGGCUCAAUUAGCCAAUUAGUGGCAU
UGUAGAGGGCUGAGAUGUUACUUUUUAGUUUCCAAUAAAAUUCUCAAGUUGGUUUCAAAAAAAAAAAAAA
A

>NR_146901.1 Homo sapiens long intergenic non-protein coding RNA 1928 (LINC01928), long non-coding RNA
AATTGGCAAACATTTTCTCTAAAGCTCAACACAGTAAATATTTTAAGCTTTACAGGGCCATACCAAACAG
GTGCTGGACCAGACCTGGACTCCCTGGGGCCACAGAGCTTCATTTGAACTAAGTAGAAGACTGCTCTGGT
TAGGGAGGCAATCGGTAAAGAAAATGAAGCATCCATCTAGCATCATCCCACTGGGAGGAAAGAAGGAGAC
CAAAAGAAGGTAAACAATAGCAAAAAAATCTCTGAAGGAGTAAATCAAGATGGAGTTGATGGATAAGGAG
GAATAAAACTACTCCAATTATCTGGAACTTACACTAAATGAAAAGCAAGTCTCCAAGATAGAAGCTGAGC
ATGACAAACAAATGCTGACAATATCAGGTAGTGCTTCAGTTCAACTATGAGGGATTCTTTCTCCCACAAC
TTCCCTACTCCAGGAACTTGGAAAGTGGATCATCTCTGACAGCTAAATTCCAAAACAAACACTTAGCAGA
TTGGAACATAATTA

RNA
>LINC01928
AAUUGGCAAACAUUUUCUCUAAAGCUCAACACAGUAAAUAUUUUAAGCUUUACAGGGCCAUACCAAACAG
GUGCUGGACCAGACCUGGACUCCCUGGGGCCACAGAGCUUCAUUUGAACUAAGUAGAAGACUGCUCUGGU
UAGGGAGGCAAUCGGUAAAGAAAAUGAAGCAUCCAUCUAGCAUCAUCCCACUGGGAGGAAAGAAGGAGAC
CAAAAGAAGGUAAACAAUAGCAAAAAAAUCUCUGAAGGAGUAAAUCAAGAUGGAGUUGAUGGAUAAGGAG
GAAUAAAACUACUCCAAUUAUCUGGAACUUACACUAAAUGAAAAGCAAGUCUCCAAGAUAGAAGCUGAGC
AUGACAAACAAAUGCUGACAAUAUCAGGUAGUGCUUCAGUUCAACUAUGAGGGAUUCUUUCUCCCACAAC
UUCCCUACUCCAGGAACUUGGAAAGUGGAUCAUCUCUGACAGCUAAAUUCCAAAACAAACACUUAGCAGA
UUGGAACAUAAUUA

>NR_110743.1 Homo sapiens long intergenic non-protein coding RNA 1929 (LINC01929), long non-coding RNA
GTGTTTGTCTCCTGGGATGCTGTGGGGTTGACCCTGGATAGCATTCACGGTCATTAGCTCATGGTGTTCT
TGGGAGACATTTGAAGTCAGCTGGTGTCCTGCTGATGGAGCTGCTGAGTTCAGCATCACATCGACATAAT
TTCATGATGCGTTTGCATGAAGGGCTTTGGTGCCATTTCTGAGTGAGACTTCAGAAGCCTCCTGAATCCT
ATTCTGGCCCATCGTGGCATGGTCAGCAGATGAGAAACAGATGGACTGGTGTGTGGTGGGAGGAAGCTGT
CCTGGTCGAGCTCTGCTTCAGGCCAGTCCTTTCTGAGATTGGAGAAGTGAGAAGAGGAGCAGAGAGACCA
GCTGGGAGCTGCTGTAGTAACCCAGGTGAGGGAGATGGTGGTCTGGACCAGTGCTTCGCATGGAGGTGGA
CAGAAGTGATATAACTGATTTAAGTCAACCAGCTGCTTGATCCAAATAATGCCAAATGAGTTTATTGCCA
TCTCTTCATATTTTACAACCAGATCACTTCTGCATAAAACCACATGGAAGAATAAGAAGGGAAAAACAAT
CAAGCAATAAACTGTTCATCTGAAAATAGTTAAATTCTCCAACAGTTGATTTTAAAATATTTTCCTAAAA
TCTATTAAGATTTGTTCAAAGTAATTCTTCTATGGAGGCACATCTCTGGACTGAAACAACCAGCCTTTGC
CTTTGGTGTGGTCCTGTTTCAGTCAAAATGGCTAACAGGCTATGAAGAAAATCAAATTCCAAGTTACTCA
AATGTGGAGAAAATCTGGAGGTCTGGTATGGGCATCTTTTCAAAATGTTTTAATTACACATTGATTTACA
AATGACATTGTCTAGTATGGTAGTTTTAAAAAAATCATCATTTTTAAACATCTGCCAAATTTTAAATGGT
AGTTTTTCATGAAAGTACAAAGAAAACTTCATCCACGATCACGAAATTTTCCAACTCATATATCCTGATC
AAAACAAAGACTTTCTATGGACAATTGTTGAACCACGCCTGACCTTGGACCCTGTGCTATCTACTCTTCT
CAGTGTCATTCTCTATCTAGTTTGGCTTTTCCAAGTTTAATCACTAAGATATTCAGATATTATTACAAAA
TATTATTACAAGCCCTTTAATTACCTTGATAATATTACTGGCAATATTAAAAACTTTTAGCAGTATCTTT
CATGGTGTGTGCTTTGCCTGAAAAGGAAAGATATCTCTGATTAAAGTTTGAAACCATTAATAATATAGAA
CACCATACAAATGACACTTTTCCAAATTAGAAATTTTAAGATCAAAGATTATTTACGGGCTTATATTTGT
ATCTAATTTTAACATTTTTATGTAGAGAAGAGGTCTCGCTGTACTGCACAGGCCAATCTCAAACTCCTGA
CTTCAAGCTATCCTCCTGCCTCAATCTTTTATTTACTTTTAATGCTATTCTTTGTTCACTTTGCTGAGAT
ATCTAGGTCAAAGCCTAAAGGCTATGTATTTTAATTTCAAGGCTGCATGTTTATATCCTCTCATACCACT
AACATCCTGGCTAATGTCTTAATGACTAATTTTTTCCAGTTTTACCACGGAATTGTCTTGAAGTTGGTGC
ATATTTCACAGACTCCAATCTGAAGTGTGGATCACAGCATTTGTTAGAAACTAGTTGACTTTGACTCTTT
TTCACTCTGAGATAGAATCTAATTGGACCTGTTATCTTGATTTTTATTGGTCAAAGCTTTCATTTCAGGC
CTCCTAATAGGATGCTGACCAGCCAATGGAATGTTTGCCTTTGGTCAGGTGTTCACCCTCTGGTCCAATT
AGCCGAGGCCAAAATGTAGTAGGGAATTGTAGCCAAAGAATGGATAAGGAATTTCCTAAGGCAGTCTTGC
TCAGCAGGTGCTATGTCAGTTTTTAGAAGAGTTTGTGGTTGTGATAATTGCTCTAAGGGTTGGCCCATAC
TGCGTATGATCTAAGGATCTAAGAATAATTTATTACCAAAGTAGCAAAAGAACTTCTTGTAAATAACCTC
TGACAAATATTTTTTATTGGCGTGTACCCTAAAGATAGCAGTACAGTGAAGCTCCAGTAACTGAAAAGAT
CCTGGATTGGTATTAAGAAATTAGAGTTAAAGGCTTATGTAACTGTCTTATAGATAATTTTAGGCAAAGC
AGTCTTGAAATAAATTCTGCTAATCAGTTAAAGAAACAGTTGAATATTTTTTAATAGCAAGAGCACTAGT
CTTAGAATCAGGAACCATGGGTTGGCTCTGAGACTCTCTGTCTCATCTTGGGGAAATCATTTCAGCTTTC
TGGCCCCACCCGGTGTTTTCCCGGAGCCCATAAGATGAATGTTGTAATAGGCTACTTCTACTTCTAAAGC
ATCCTTGATCTGAAATTTGAGAAACAGAACTGAGAATCAAAATGAAATAAAAGCTTGAATAGATGTTAGG
AGACCAAAAAAAAAAAAAAAAGCCAGAGAGAAATTATTTATAGAGCATTCACTATGATATCCCGTGCGCTT
TACACATGTTGACTTCTCATTCCTCAGAGAAAGGCTCTGAGGTTGTATCCAGTTTACAGTAGAAAACAGG
TAAGATACCTTGCCCCAGCCATACAAACTGTAAACAGTGGAGTCAACCCAATTTTGTTTAGCGAAAAGAA
TAATAAAGTGCTTTCCATCATACCAAATTCTTCCTTAAATGCAGGGAGAAACAATGCTTATGGAAAAATA
GAGGCAGGTAAAAAAAACAGTAATGTTTTACGAAGAAATAACAGCATTTTCACTAAATGATGTGGAGCTT
GGGTTGAGAATGCTGATATATGACTCAGAATTCAAACCTTTTGCACTGATTGTTTTCACTTAGATTTTAT
GAAAGCTGTAGGTGTGTGAGTTAACGAGGAATGCTTCAGCACTTCCCTGGTCATGCATAAACATCTACAT
GCAAAACAGCAAGGAGTGCTCTGTTTATCACAGCTGCAGTCAAGCCAACAAAACTGTTTCTACCACTGAG
GATGAAATTTGTAAAATTTTCTCTCAAAAAATAAAATAAGCCTCTTGTTTGATTGCC

RNA
>LINC01929
GUGUUUGUCUCCUGGGAUGCUGUGGGGUUGACCCUGGAUAGCAUUCACGGUCAUUAGCUCAUGGUGUUCU
UGGGAGACAUUUGAAGUCAGCUGGUGUCCUGCUGAUGGAGCUGCUGAGUUCAGCAUCACAUCGACAUAAU
UUCAUGAUGCGUUUGCAUGAAGGGCUUUGGUGCCAUUUCUGAGUGAGACUUCAGAAGCCUCCUGAAUCCU
AUUCUGGCCCAUCGUGGCAUGGUCAGCAGAUGAGAAACAGAUGGACUGGUGUGUGGUGGGAGGAAGCUGU
CCUGGUCGAGCUCUGCUUCAGGCCAGUCCUUUCUGAGAUUGGAGAAGUGAGAAGAGGAGCAGAGAGACCA
GCUGGGAGCUGCUGUAGUAACCCAGGUGAGGGAGAUGGUGGUCUGGACCAGUGCUUCGCAUGGAGGUGGA
CAGAAGUGAUAUAACUGAUUUAAGUCAACCAGCUGCUUGAUCCAAAUAAUGCCAAAUGAGUUUAUUGCCA
UCUCUUCAUAUUUUACAACCAGAUCACUUCUGCAUAAAACCACAUGGAAGAAUAAGAAGGGAAAAACAAU
CAAGCAAUAAACUGUUCAUCUGAAAAUAGUUAAAUUCUCCAACAGUUGAUUUUAAAAUAUUUUCCUAAAA
UCUAUUAAGAUUUGUUCAAAGUAAUUCUUCUAUGGAGGCACAUCUCUGGACUGAAACAACCAGCCUUUGC
CUUUGGUGUGGUCCUGUUUCAGUCAAAAUGGCUAACAGGCUAUGAAGAAAAUCAAAUUCCAAGUUACUCA
AAUGUGGAGAAAAUCUGGAGGUCUGGUAUGGGCAUCUUUUCAAAAUGUUUUAAUUACACAUUGAUUUACA
AAUGACAUUGUCUAGUAUGGUAGUUUUAAAAAAAUCAUCAUUUUUAAACAUCUGCCAAAUUUUAAAUGGU
AGUUUUUCAUGAAAGUACAAAGAAAACUUCAUCCACGAUCACGAAAUUUUCCAACUCAUAUAUCCUGAUC
AAAACAAAGACUUUCUAUGGACAAUUGUUGAACCACGCCUGACCUUGGACCCUGUGCUAUCUACUCUUCU

CAGUGUCAUUCUCUAUCUAGUUUGGCUUUUCCAAGUUUAAUCACUAAGAUAUUCAGAUAUUAUUACAAAA
UAUUAUUACAAGCCCUUUAAUUACCUUGAUAAUAUUACUGGCAAUAUUAAAAACUUUUAGCAGUAUCUUU
CAUGGUGUGUGCUUUGCCUGAAAAGGAAAGAUAUCUCUGAUUAAAGUUUGAAACCAUUAAUAAUAUAGAA
CACCAUACAAAUGACACUUUUCCAAAUUAGAAAUUUUAAGAUCAAAGAUUAUUUACGGGCUUAUAUUUGU
AUCUAAUUUUAACAUUUUUAUGUAGAGAAGAGGUCUCGCUGUACUGCACAGGCCAAUCUCAAACUCCUGA
CUUCAAGCUAUCCUCCUGCCUCAAUCUUUUAUUUACUUUUAAUGCUAUUCUUUGUUCACUUUGCUGAGAU
AUCUAGGUCAAAGCCUAAAGGCUAUGUAUUUUAAUUUCAAGGCUGCAUGUUUAUAUCCUCUCAUACCACU
AACAUCCUGGCUAAUGUCUUAAUGACUAAUUUUUUCCAGUUUUACCACGGAAUUGUCUUGAAGUUGGUGC
AUAUUUCACAGACUCCAAUCUGAAGUGUGGAUCACAGCAUUUGUUAGAAACUAGUUGACUUUGACUCUUU
UUCACUCUGAGAUAGAAUCUAAUUGGACCUGUUAUCUUGAUUUUUAUUGGUCAAAGCUUUCAUUUCAGGC
CUCCUAAUAGGAUGCUGACCAGCCAAUGGAAUGUUUGCCUUUGGUCAGGUGUUCACCCUCUGGUCCAAUU
AGCCGAGGCCAAAAUGUAGUAGGGAAUUGUAGCCAAAGAAUGGAUAAGGAAUUUCCUAAGGCAGUCUUGC
UCAGCAGGUGCUAUGUCAGUUUUUAGAAGAGUUUGUGGUUGUGAUAAUUGCUCUAAGGGUUGGCCCAUAC
UGCGUAUGAUCUAAGGAUCUAAGAAUAAUUUAUUACCAAAGUAGCAAAAGAACUUCUUGUAAAUAACCUC
UGACAAAUAUUUUUUAUUGGCGUGUACCCUAAAGAUAGCAGUACAGUGAAGCUCCAGUAACUGAAAAGAU
CCUGGAUUGGUAUUAAGAAAUUAGAGUUAAAGGCUUAUGUAACUGUCUUAUAGAUAAUUUUAGGCAAAGC
AGUCUUGAAAUAAAUUCUGCUAAUCAGUUAAAGAAACAGUUGAAUAUUUUUUAAUAGCAAGAGCACUAGU
CUUAGAAUCAGGAACCAUGGGUUGGCUCUGAGACUCUCUGUCUCAUCUUGGGGAAAUCAUUUCAGCUUUC
UGGCCCCACCCGGUGUUUUCCCGGAGCCCAUAAGAUGAAUGUUGUAAUAGGCUACUUCUACUUCUAAAGC
AUCCUUGAUCUGAAAUUUGAGAAACAGAACUGAGAAUCAAAAUGAAAUAAAAGCUUGAAUAGAUGUUAGG
AGACCAAAAAAAAAAAAAAAAGCCAGAGAGAAAUUAUUUAUAGAGCAUUCACUAUGAUAUCCCGUGCGCUU
UACACAUGUUGACUUCUCAUUCCUCAGAGAAAGGCUCUGAGGUUGUAUCCAGUUUACAGUAGAAAACAGG
UAAGAUACCUUGCCCCAGCCAUACAAACUGUAAACAGUGGAGUCAACCCAAUUUUGUUUAGCGAAAAGAA
UAAUAAAGUGCUUUCCAUCAUACCAAAUUCUUCCUUAAAUGCAGGGAGAAACAAUGCUUAUGGAAAAAUA
GAGGCAGGUAAAAAAAACAGUAAUGUUUUACGAAGAAAUAACAGCAUUUUCACUAAAUGAUGUGGAGCUU
GGGUUGAGAAUGCUGAUAUAUGACUCAGAAUUCAAACCUUUUGCACUGAUUGUUUUCACUUAGAUUUUAU
GAAAGCUGUAGGUGUGUGAGUUAACGAGGAAUGCUUCAGCACUUCCCUGGUCAUGCAUAAACAUCUACAU
GCAAAACAGCAAGGAGUGCUCUGUUUAUCACAGCUGCAGUCAAGCCAACAAAACUGUUUCUACCACUGAG
GAUGAAAUUUGUAAAAUUUUCUCUCAAAAAAUAAAAUAAGCCUCUUGUUUGAUUGCC

>NR_149137.1 Homo sapiens long intergenic non-protein coding RNA 2564 (LINC02564), transcript variant 1, long non-coding RNA
GGGCGATGCTCCCCGGGTAGGACAAACCGGTCACCTGGGCTGCGAGGGCGGCTTAGGGGCAGAAGCGGCG
GTCCAGGGCCGCCTGGCGCAGCAGCCTGTCCCAGCCGCGGTCCCTGCAGTCCCTCCCTGGCGGCTGCGCA
GCCGTCCCACGACAGGGGCCATAAACTCTCCAGAGCGGAAAGCCGCACCCTGGTGGCCCGGCCCCGCGCC
CAGACCTGGCGGCCGCTGGCACCTGACCCGCTGCATGGGTCTCCAGGGAGCTCGCTGCCCACCCGGCGCT
GCAGGCTCGGCTCCCTCGTACACTCTCTGGCAAGCCCAAGAAAGTCAGGGGCCTATGTGAGCCAAAGAGG
AGAGAAGGTGATGCCTCAGCCCAGTGTTTCTGCCCCACCTCGCTTGTGGCCTTCGGAACTTGATTTGCAC
CGCAGGAAAATGGGCAATGAAAACCCCTCCCTAACTGGCTTCTCAGTCCACTCTGACCAGCCCACTGCAC
AGCGCCCACCCTGCAGCTCCAGATGAGGCCTCACTCTGTCACCCAGGTTGGGGTGGAGTGGCACAGTCAC
AGCTCACTATAACCTCAAGCTCCTGGGCTCAAGTGATCCTGCCACCTCAGCCTCCTAAGTAGCTGGAACT
ACAGATGTGCACTGCCATGCCAGGCTTGTCTAACATTTTTATGTGTTGCTTCATCCAGTTTGCTAGAGTT
TTTGGAGATTTCTGTCTTCATTCATGAGGGATAATAGTCTGCACTTTTATTTTCTTGTGATACTTTTGTC
TGATTTGTTATCTGGGTAATACTGGCCTTGAAAATGAATTGATGTTTTCCTGCTTC

RNA
>LINC02564
GGGCGAUGCUCCCCGGGUAGGACAAACCGGUCACCUGGGCUGCGAGGGCGGCUUAGGGGCAGAAGCGGCG
GUCCAGGGCCGCCUGGCGCAGCAGCCUGUCCCAGCCGCGGUCCCUGCAGUCCCUCCCUGGCGGCUGCGCA
GCCGUCCCACGACAGGGGCCAUAAACUCUCCAGAGCGGAAAGCCGCACCCUGGUGGCCCGGCCCCGCGCC
CAGACCUGGCGGCCGCUGGCACCUGACCCGCUGCAUGGGUCUCCAGGGAGCUCGCUGCCCACCCGGCGCU
GCAGGCUCGGCUCCCUCGUACACUCUCUGGCAAGCCCAAGAAAGUCAGGGGCCUAUGUGAGCCAAAGAGG
AGAGAAGGUGAUGCCUCAGCCCAGUGUUUCUGCCCCACCUCGCUUGUGGCCUUCGGAACUUGAUUUGCAC
CGCAGGAAAAUGGGCAAUGAAAACCCCUCCCUAACUGGCUUCUCAGUCCACUCUGACCAGCCCACUGCAC
AGCGCCCACCCUGCAGCUCCAGAUGAGGCCUCACUCUGUCACCCAGGUUGGGGUGGAGUGGCACAGUCAC
AGCUCACUAUAACCUCAAGCUCCUGGGCUCAAGUGAUCCUGCCACCUCAGCCUCCUAAGUAGCUGGAACU
ACAGAUGUGCACUGCCAUGCCAGGCUUGUCUAACAUUUUUAUGUGUUGCUUCAUCCAGUUUGCUAGAGUU
UUUGGAGAUUUCUGUCUUCAUUCAUGAGGGAUAAUAGUCUGCACUUUUAUUUUCUUGUGAUACUUUUGUC
UGAUUUGUUAUCUGGGUAAUACUGGCCUUGAAAAUGAAUUGAUGUUUUCCUGCUUC

>NR_149129.1 Homo sapiens long intergenic non-protein coding RNA 2565 (LINC02565), long non-coding RNA

GCCTCCAGAGCTATCTTTGCTTGAAAGAATGAAACTTCAGGAATGAAAGTTTGCACATAAAGCAAGCCCA
TGAGAAAGACATTTCGGGAAAACTAGAATCCTGTTAAGTTATGGGATTTTTTTCAAGCCACATGGTTGAT
AAGTAGTCTGACCAGGATCCTGGAGCAACACAAGGAAGACAAGGACCCTTGAAGACAAGGCAGCCTTGAA
ACCAAATGAAGCCCTGGGCCAGTGAGCATGGCAAGAGGAAGACTGTTAATTGCGAGAAAGCTGGGTCTTG
AAGATGATTCCATATCAACATACAGGGAGTTCCACCACTGAATGGTCTATCGGTTGGGAATGCCATCATT
TATTTTCACCAGTGGGGAAGAATTGGTATTAATATCAAAACCACAGTACTGTCATACCCAGCTATAATTT
GCAATGTTTATATGCCTGTACGAATGAATAATTAACAGTTAAATGAATCAATATATAT

RNA

>LINC02565

GCCUCCAGAGCUAUCUUUGCUUGAAAGAAUGAAACUUCAGGAAUGAAAGUUUGCACAUAAAGCAAGCCCA
UGAGAAAGACAUUUCGGGAAAACUAGAAUCCUGUUAAGUUAUGGGAUUUUUUUCAAGCCACAUGGUUGAU
AAGUAGUCUGACCAGGAUCCUGGAGCAACACAAGGAAGACAAGGACCCUUGAAGACAAGGCAGCCUUGAA
ACCAAAUGAAGCCCUGGGCCAGUGAGCAUGGCAAGAGGAAGACUGUUAAUUGCGAGAAAGCUGGGUCUUG
AAGAUGAUUCCAUAUCAACAUACAGGGAGUUCCACCACUGAAUGGUCUAUCGGUUGGGAAUGCCAUCAUU
UAUUUUCACCAGUGGGGAAGAAUUGGUAUUAAUAUCAAAACCACAGUACUGUCAUACCCAGCUAUAAUUU
GCAAUGUUUAUAUGCCUGUACGAAUGAAUAAUUAACAGUUAAAUGAAUCAAUAUAUAU

>NR_038340.1 Homo sapiens long intergenic non-protein coding RNA 2582 (LINC02582), long non-coding RNA
GCTGACTCTCTTTTCGGACTCAGCCCGCCTGCACCCAGGTGAAATAAACAGCCATGTTGCTCACACAAAG
CCTGTTTGGTGGTCTCTTCACAGGGACACGGATGAAATTTGGTGCCGTGACTCGGATCGGGGAACCTCCC
TTAGGAGATCAATCCCCTGTACTCCTTTTCTTTGCCCTGTGAGAAAGATCCACCTATGACCTCAGGTCCT
CAGACCGACCAGCCCAAGGAACATCTCACCAATTTTAAATCAGACCTTGAAGATTTGTTGTTCAAGGAGA
AACTGAAGAGCAAGAAGGAAAGTGAGAGCCAGCAATACCAGCAGAGCCAGATCTGAGCTGGGAGAAGGGG
AGAAAGTTTGTGAAGAGGAGATCGGTGACCTGGGCTCCTTATGTGCCTGAAAGAGTTTGAGTTTCCTGTT
AACTCCAAATCAACAGTATTTTCAACAAGAAATGTGCAATTGAAATCAAGTGCTGTTTAAGTGCAGCTAG
GATTTCCACAGGAAGACACTTGCAGTGAACAGAGTTATGGAGCAGCAAAAACACAGATCTATTTGGAAAA
AGAGAAAACATATGCGTTGTATTTTGCTTCAATTATAAAATACCATCCTCTCAAAGGTGGTTCTAAATTA
CAAAGGACTTTGATTTCTAGGTAGATTCTGGGTAGAGACTTCCTTTCATATTGAGGCATTAATGACACCT
TTTAACCTGGGAAGCAATATGACTGGAGTTGTACTTTGAGAAGATTAATCAGGTTTGGTTGCAGAATGAA
AGAGAAGATGAAGTCAAGAGATTGGTTTAGAGGCTCTAGCAGAAGCTTAGTCATATTTCAAAATGATCAA
ATATCAAGAAAAATTCTGAGCTGCATAACTTGTATAAAGTAATTTTCAGTGATTTTTTTCATGGTTATGA
TAAAAGAACTGGATTAGCAGAAACTTTTACCCTGAATCAAGATTTAATTTTTCTTTGAGCTCATCTTAAG
GATATCGGAACATAGGGAGCAAACGATGGTGTGGCTGCCTCAGTGCTTGATTTTTAACGGTTTTGAAGAG
AATAGTTACATTTCTTCTCCTAGTAAGAACTAATAAATACATTAACAGAAATGAATTCCCTATCCCTTTG
TACACTGGTCTATTTCTTCAAAACATTAAATACTATTGATAAGATATCACCTTTTAAAAAA

RNA
>LINC02582
GCUGACUCUCUUUUCGGACUCAGCCCGCCUGCACCCAGGUGAAAUAAACAGCCAUGUUGCUCACACAAAG
CCUGUUUGGUGGUCUCUUCACAGGGACACGGAUGAAAUUUGGUGCCGUGACUCGGAUCGGGGAACCUCCC
UUAGGAGAUCAAUCCCCUGUACUCCUUUUCUUUGCCCUGUGAGAAAGAUCCACCUAUGACCUCAGGUCCU
CAGACCGACCAGCCCAAGGAACAUCUCACCAAUUUUAAAUCAGACCUUGAAGAUUUGUUGUUCAAGGAGA
AACUGAAGAGCAAGAAGGAAAGUGAGAGCCAGCAAUACCAGCAGAGCCAGAUCUGAGCUGGGAGAAGGGG
AGAAAGUUUGUGAAGAGGAGAUCGGUGACCUGGGCUCCUUAUGUGCCUGAAAGAGUUUGAGUUUCCUGUU
AACUCCAAAUCAACAGUAUUUUCAACAAGAAAUGUGCAAUUGAAAUCAAGUGCUGUUUAAGUGCAGCUAG
GAUUUCCACAGGAAGACACUUGCAGUGAACAGAGUUAUGGAGCAGCAAAAACACAGAUCUAUUUGGAAAA
AGAGAAAACAUAUGCGUUGUAUUUUGCUUCAAUUAUAAAAUACCAUCCUCUCAAAGGUGGUUCUAAAUUA
CAAAGGACUUUGAUUUCUAGGUAGAUUCUGGGUAGAGACUUCCUUUCAUAUUGAGGCAUUAAUGACACCU
UUUAACCUGGGAAGCAAUAUGACUGGAGUUGUACUUUGAGAAGAUUAAUCAGGUUUGGUUGCAGAAUGAA
AGAGAAGAUGAAGUCAAGAGAUUGGUUUAGAGGCUCUAGCAGAAGCUUAGUCAUAUUUCAAAAUGAUCAA
AUAUCAAGAAAAAUUCUGAGCUGCAUAACUUGUAUAAAGUAAUUUUCAGUGAUUUUUUUCAUGGUUAUGA
UAAAAGAACUGGAUUAGCAGAAACUUUUACCCUGAAUCAAGAUUUAAUUUUUCUUUGAGCUCAUCUUAAG
GAUAUCGGAACAUAGGGAGCAAACGAUGGUGUGGCUGCCUCAGUGCUUGAUUUUUAACGGUUUUGAAGAG
AAUAGUUACAUUUCUUCUCCUAGUAAGAACUAAUAAAUACAUUAACAGAAAUGAAUUCCCUAUCCCUUUG
UACACUGGUCUAUUUCUUCAAAACAUUAAAUACUAUUGAUAAGAUAUCACCUUUUAAAAAA

>NR_183516.1 Homo sapiens long intergenic non-protein coding RNA 2837 (LINC02837), transcript variant 1, long non-coding RNA
ATTCCTGGGAGGTGTCTGGGAAGCACATCGCAGTGTGCACCGCAGCAGGGCTTGATCCACCACATCACAT
TCCGCGCTCGCTGCTGCCTGAACTCATCAAAGTGGACCCAGTGAAAGCTGTGTTGAGGAGGACTATTCTG
GTGCTGAGGCTGGGTACATTCATGAGGCCAGGAACACGGGATTGAATTCCAAGCAGAGCACCAGGATACC
TGTAGCAGAAATCCAGGCAAGAGCCAAGAAGATGGGAACGGAATAGCAAGCGCTGAATTAATTAATTAAT
TAATTAATTTTTTGAGATGGAGTCTTGCTCTGTCGCCCAGGCTGGAGTGCAGTGGTGTAATGTTGGCTCA
CTGCAACCTCCCCCTCCGGGGTTCAAGCGATTCTCCTGCCTCAGCCTCCCAAGTAGCTAGGATTACAGGT
GCCCGCCACCATGCTCGGCTAATTTTTTGTATTTTTAGTAGAGACGAGGTTTCGCCATGTTGGCCAGGCT
GGTCTGGAACTCCTGACCTCAAGTGATTGGCCGGCCTTGGGCAAGTGCTGAATTTAAACACGGCTCTCCT
GAGGACTTCTATTGGGTTCTGCCTAGTGGTAACCGCCGAGGCTTCGCTTACTCTGTAGATCTCCCGGGAG
CAGCAGGAAACAAGAAACCCTTAGCCTTTTCAAAGTAATAGCCATTCTCCTCACTGACCTGACAAAGACG
GATGTCAAGTGGGCCCTGCAGGAGTTTGACAGTTTGTCCAAACAACTGTTTTTAGTTGAGGCTATAAATT
CTTAGGAAAGGGTTTGTCAGTGTGGCACATTAAATCAGTTGGCCAGTATGCATTACTCTGACCATCTTCT
TAGAAAGCAACGTCTTTGGGGATTTTCAGATGAGTGAATATTCAATGCTCTGTCTTTTAGCATTATCATC
CACTTCCCCTGCCCCTCATAGAATTCTCTTTAGAGGATTGGCTCCTGGGCCTCCAGGAGAGCGGGCAGTA
GGGTAGGGTGGTGGGAGGAGAGGAAAGTCTTAATCCCTTGCCAGGGATTTACTTTGTTCTTCTCAGTCGG
AGCCTCCCGTACAATGCTGTTTTGAGCTTCACTTAGAAATTGGCTTTATTAGCTAGGAAAGGGGAATTCT
TTTCACAACAATCGGAAAATGTAATCCCTTAGTCAGGCGGCTGGAGTCCTTTAATTCACCTCTCCGGACC
TTGGGCTGCTGGCTGCTCCTCTCTGCCGCCTGGCTGTCTCTCCCTTACCTCCCAGAGTTAGGGTAATAGC
TGACATTTTTCTTCACTAGGCTTTCCTAAGGGATTTCATTCCATTTGTGAGACACTTTAATTCCTGAAGC
TGCAAAGTTTGCTTTTTATTACTATAGCTCTACTTATTCTGCATAATTTTATTTATTATTTGTTTATGTA
TTTATTTTTTGAGACAGAGTCTCGCTGTGTTGCCCAGGCTGGAGTGCAGTGGCGCCATCTCAGCTCACTG
CAACCTCTGCCTCCTGGGTTCAAGCAATTCTCCTTCCTCAGTCTCCTGAGCAGCTGGGACTACAGGCACG
TGCTACCACGCCCAGCTAAGTTTTGTATTTTTAGTAGAGACGAGGTTTCATCATATTGGCCAGGCTGGTC
TCGAACTCCTGACCTTGTGATCCACCTGCCTCAGCCTCCCAAAGTGCTGGGATTACAGGCGTGAGCCACC
ACGCCTGGCTTATTCTGCATAATTTTTAAGAGTTGTGAATTGTTTTAAGATCAAATTTAAAAGTACATTT
TCTTTCTTATCTTGTCATTAACTGATGTTGGGTATGTGTGTGTGCTTGTGATTTCCCTGGCTGGACCAGT
GTATAACTTCAAAGAGGGCTGGTGCCTTGCCTTTAGTCTGCTGGATAGCACTCCTCCAGACCCTCCCTTT
GCTGAGCAGCTCCGTGGACCTGTGCAAATCTCTACCCAGCTTTGTTGGACAACAAAATACCCTGGAGAAT
ATCAAGCCCTTTAACATACCCAGTTTGTGCTCCCAGACGTAAGATCTCTTTGGCCATGTATGTGGGATTC
CCATGAAGGAGAAGCTTCATTTCCAGCAAGAATCTGGCTTCCACCTAGACTTGGGAAACAGATACTGAAA
AGCATATTGCTGATCAACTAGACAACCTACTTCCACTCTTGATTGCCCCTGTAATCCATCTAGAACAGCC
AGAGCAATTTATTTATTAAAAAATTTAAAATTG

RNA
>LINC02837
AUUCCUGGGAGGUGUCUGGGAAGCACAUCGCAGUGUGCACCGCAGCAGGGCUUGAUCCACCACAUCACAU
UCCGCGCUCGCUGCUGCCUGAACUCAUCAAAGUGGACCCAGUGAAAGCUGUGUUGAGGAGGACUAUUCUG
GUGCUGAGGCUGGGUACAUUCAUGAGGCCAGGAACACGGGAUUGAAUUCCAAGCAGAGCACCAGGAUACC
UGUAGCAGAAAUCCAGGCAAGAGCCAAGAAGAUGGGAACGGAAUAGCAAGCGCUGAAUUAAUUAAUUAAU
UAAUUAAUUUUUUGAGAUGGAGUCUUGCUCUGUCGCCCAGGCUGGAGUGCAGUGGUGUAAUGUUGGCUCA
CUGCAACCUCCCCCUCCGGGGUUCAAGCGAUUCUCCUGCCUCAGCCUCCCAAGUAGCUAGGAUUACAGGU
GCCCGCCACCAUGCUCGGCUAAUUUUUUGUAUUUUUAGUAGAGACGAGGUUUCGCCAUGUUGGCCAGGCU
GGUCUGGAACUCCUGACCUCAAGUGAUUGGCCGGCCUUGGGCAAGUGCUGAAUUUAAACACGGCUCUCCU
GAGGACUUCUAUUGGGUUCUGCCUAGUGGUAACCGCCGAGGCUUCGCUUACUCUGUAGAUCUCCCGGGAG
CAGCAGGAAACAAGAAACCCUUAGCCUUUUCAAAGUAAUAGCCAUUCUCCUCACUGACCUGACAAAGACG
GAUGUCAAGUGGGCCCUGCAGGAGUUUGACAGUUUGUCCAAACAACUGUUUUUAGUUGAGGCUAUAAAUU
CUUAGGAAAGGGUUUGUCAGUGUGGCACAUUAAAUCAGUUGGCCAGUAUGCAUUACUCUGACCAUCUUCU
UAGAAAGCAACGUCUUUGGGGAUUUUCAGAUGAGUGAAUAUUCAAUGCUCUGUCUUUUAGCAUUAUCAUC
CACUUCCCCUGCCCCUCAUAGAAUUCUCUUUAGAGGAUUGGCUCCUGGGCCUCCAGGAGAGCGGGCAGUA
GGGUAGGGUGGUGGGAGGAGAGGAAAGUCUUAAUCCCUUGCCAGGGAUUUACUUUGUUCUUCUCAGUCGG
AGCCUCCCGUACAAUGCUGUUUUGAGCUUCACUUAGAAAUUGGCUUUAUUAGCUAGGAAAGGGGAAUUCU
UUUCACAACAAUCGGAAAAUGUAAUCCCUUAGUCAGGCGGCUGGAGUCCUUUAAUUCACCUCUCCGGACC
UUGGGCUGCUGGCUGCUCCUCUCUGCCGCCUGGCUGUCUCUCCCUUACCUCCCAGAGUUAGGGUAAUAGC
UGACAUUUUUCUUCACUAGGCUUUCCUAAGGGAUUUCAUUCCAUUUGUGAGACACUUUAAUUCCUGAAGC
UGCAAAGUUUGCUUUUUAUUACUAUAGCUCUACUUAUUCUGCAUAAUUUUAUUUAUUAUUUGUUUAUGUA
UUUAUUUUUUGAGACAGAGUCUCGCUGUGUUGCCCAGGCUGGAGUGCAGUGGCGCCAUCUCAGCUCACUG
CAACCUCUGCCUCCUGGGUUCAAGCAAUUCUCCUUCCUCAGUCUCCUGAGCAGCUGGGACUACAGGCACG
UGCUACCACGCCCAGCUAAGUUUUGUAUUUUUAGUAGAGACGAGGUUUCAUCAUAUUGGCCAGGCUGGUC
UCGAACUCCUGACCUUGUGAUCCACCUGCCUCAGCCUCCCAAAGUGCUGGGAUUACAGGCGUGAGCCACC
ACGCCUGGCUUAUUCUGCAUAAUUUUUAAGAGUUGUGAAUUGUUUUAAGAUCAAAUUUAAAAGUACAUUU
UCUUUCUUAUCUUGUCAUUAACUGAUGUUGGGUAUGUGUGUGUGCUUGUGAUUUCCCUGGCUGGACCAGU
GUAUAACUUCAAAGAGGGCUGGUGCCUUGCCUUUAGUCUGCUGGAUAGCACUCCUCCAGACCCUCCCUUU

GCUGAGCAGCUCCGUGGACCUGUGCAAAUCUCUACCCAGCUUUGUUGGACAACAAAAUACCCUGGAGAAU
AUCAAGCCCUUUAACAUACCCAGUUUGUGCUCCCAGACGUAAGAUCUCUUUGGCCAUGUAUGUGGGAUUC
CCAUGAAGGAGAAGCUUCAUUUCCAGCAAGAAUCUGGCUUCCACCUAGACUUGGGAAACAGAUACUGAAA
AGCAUAUUGCUGAUCAACUAGACAACCUACUUCCACUCUUGAUUGCCCCUGUAAUCCAUCUAGAACAGCC
AGAGCAAUUUAUUUAUUAAAAAAAUUUAAAAUUG

>XR_002958226.1 PREDICTED: Homo sapiens long intergenic non-protein coding RNA 2856 (LINC02856), ncRNA

AGTCTCTCAAATAACAGATAAAGAAATTAGGCAAGGTACTGCTGTCTCCATCTCTGTAAAATAAAACCTA
GGCACTTTCACTGATAATAGACTAGCACCAACATGCAGTCAGTAACACGGGACACTAAAAACAAGCCATT
TGGATCACACAAAGAAGAATATTGACTCGTGCCACTTTAAAACATGTGATATTATTTGGCATTCACAATT
CTTTCCCTATTTGTTGTCTGTATTGAATTTTTCCTTTGACTTAGTGCACTTGCTCATGTATCTTGCCCTC
TTTTAAACCTTGGTTGGTGACGGTTCTTTGCACAATTGCGGAGGTCTTTTGCTACTCCAATTGCATAAAT
AACTGAAGTAAGGCTGCAAAAGTCAACCGAGCTTGGGCATCAAGGACAATGAAGTTGCTGAGTATCTCTT
GTGCCTCACTGACTCCTTTCCAGTCAGGAGACGACCAGTTTGAAGCAGAAGAATGTCCTGATAATGGCAT
AGAGCCAAAGCGATTCCATCCTCTGGACATGAGCTGTGTGGTGTCCCCGTCCTCATACCTATTCCAGAAC
CACACTGGTCCCTGCTCTCGTCTCCGAACTGTCGGAGGATGGACCTGCTTTTGCAAGGACCTGAACTCCC
TGTGTTGTTGCTTAAGATTTTTACCCAGGCATGAAAAGGAAATGAATTCTGCCAACTCATCGCTGTGTCT
GTGGGAACAGAAACTCAGGGCACCTATTCTCTGCAAGAAAAGCATCAATTCCCTGTAAGAAAAGTTTCCC
ACCTGAGACAATGACACAGACCAACATAAATGCTCTTTTGGTTTTATGATTTCTGATATTAGATTTTACT
TGATTTTTTTATTTTTAATTTTTTAAATTTCATTTTGAGAGTTAAAAGGGTTACTTCTTTTATTTCCAGC
AGTTCAAGGAATTTCAGAGCAATCTTGGTCCAGAGTTGGTAATAAAGACATTTAAATGAGATGGGAATAC
TGTCTGTTATGCTGACCAGTACTAAGTCTCCCGCAGTTCATTTTTCCTGAGAATAAATGCCTGTCTGATA
CATCAAGA

RNA

>LINC02856

AGUCUCUCAAAUAACAGAUAAAGAAAUUAGGCAAGGUACUGCUGUCUCCAUCUCUGUAAAAUAAAACCUA
GGCACUUUCACUGAUAAUAGACUAGCACCAACAUGCAGUCAGUAACACGGGACACUAAAAACAAGCCAUU
UGGAUCACACAAAGAAGAAUAUUGACUCGUGCCACUUUAAAACAUGUGAUAUUAUUUGGCAUUCACAAUU
CUUUCCCUAUUUGUUGUCUGUAUUGAAUUUUUCCUUUGACUUAGUGCACUUGCUCAUGUAUCUUGCCCUC
UUUUAAACCUUGGUUGGUGACGGUUCUUUGCACAAUUGCGGAGGUCUUUUGCUACUCCAAUUGCAUAAAU
AACUGAAGUAAGGCUGCAAAAGUCAACCGAGCUUGGGCAUCAAGGACAAUGAAGUUGCUGAGUAUCUCUU
GUGCCUCACUGACUCCUUUCCAGUCAGGAGACGACCAGUUUGAAGCAGAAGAAUGUCCUGAUAAUGGCAU
AGAGCCAAAGCGAUUCCAUCCUCUGGACAUGAGCUGUGUGGUGUCCCCGUCCUCAUACCUAUUCCAGAAC
CACACUGGUCCCUGCUCUCGUCUCCGAACUGUCGGAGGAUGGACCUGCUUUUGCAAGGACCUGAACUCCC
UGUGUUGUUGCUUAAGAUUUUUACCCAGGCAUGAAAAGGAAAUGAAUUCUGCCAACUCAUCGCUGUGUCU
GUGGGAACAGAAACUCAGGGCACCUAUUCUCUGCAAGAAAAGCAUCAAUUCCCUGUAAGAAAAGUUUCCC
ACCUGAGACAAUGACACAGACCAACAUAAAUGCUCUUUUGGUUUUAUGAUUUCUGAUAUUAGAUUUUACU
UGAUUUUUUUAUUUUUAAUUUUUUAAAUUUCAUUUUGAGAGUUAAAAGGGUUACUUCUUUUAUUUCCAGC
AGUUCAAGGAAUUUCAGAGCAAUCUUGGUCCAGAGUUGGUAAUAAAGACAUUUAAAUGAGAUGGGAAUAC
UGUCUGUUAUGCUGACCAGUACUAAGUCUCCCGCAGUUCAUUUUUCCUGAGAAUAAAUGCCUGUCUGAUA
CAUCAAGA

>NR_034133.1 Homo sapiens long intergenic non-protein coding RNA 2864 (LINC02864), long non-coding RNA

TTCTCCTGCCTCAGCCTCCCGAGTAGCTGGAACTACAGGCGCCCGCCATGATGCCCGGCTCATTTTCTGT
GTTTTCAGTAGAGACGGGGTTTCACTGTGTTAGCCAGGAAAGTCTTGATCTCCTGACCTCGTGATCCTGA
CCTCGGCCTCCCAAAGTGCTGGGATTACAGGCGCGAGCCACCGCGCCCGACAGCAGTTATGAAGTTCTAA
AAGCAAGTCTTAATCAGGAAGTGTCCTTGATCACCAACGGCTCGCCCAGGCATGCTGGCTCTCTTCCACT
TCCACCTTCCACCATGGGATGACGCAGTAAGAAGGCCATCAGTAGATGCCAGTCCCTCAACCTTGAACTT
TCCAGACGCAGAACTTTATGCCTCCATTTTCCTCTGCTGCATGGCCCCAGGAGAGATTTTAATTAGCTTT
CTAACCTTGGTCCAGATTGCACATGCAAATGGTAGAGGATGCAACACCCCCGCTTGTGGAGCTGCCGCTT
GTGTCTGGCATGAAAATTCACAAGAAGAGAGGAAATACTGAGGAGAAAATGGCAGATTGTGTTTGCTGAA
TTTGATTGACGAAGAAGTCACCATGAAAATCACAGTGAACCATTTGGAAAGCAAACTGCCAAAAAAATAAT
AGTTAGTCATGCTCTCAGGCTGGTTGTTTTGGCTGTTGTGGGTTTCTTGCATTTCCAGATGATTGCAAAG
AGCTGTTTCTCAATTTCTGCAACAAGTGCCAGCTGAAATTTTGGTACCAGTTTCATTAAATATGTAGAAC
AATTTGAGCCTGAGTGAGTTCTGTGGAGCCCATACTACCTGCGATTCAAGCCAGCAACCTTGGAATCTCC
CTGAACAATTTCTATGGTGTCCAATCACATTTGCAATCACTCCCCACACTCAGTAGGTTCTGCCCCCACA
TCATTTCTTTTCTCTATCCTCTTTCCCAGCTTTATAATATGACTTAGTTCAAGCCCTAATCACTGGATTA
TTCAAAGTCCCTTAACAGGTTAATTCCTACTGTGATCAACACTTTAAAGGAAGGCAATAGGGCCAGGCAT
GGTGGCTCACGGCTGTAATCCCAGCACTTTGGGAGGCTGAGGCGAGTGGATCACAAGGTCGGGAGTTTGA
GACCAGCCTGACTAACACAGTGAAACCCCGACTCTACTAAAAATACAAAAAATTAGCCAGGTGTGGTCGT
GGGTACCTGTAGTCCCAGCTACTAGGGAGGCTGAGGCAGGAGAATGGCGTGAACCCGAGAGGCGGAGCTT
GCAGTGAGCCAAGATTACGCCACTGCACTCCAGCCTGGGTGACGGAGCGAGACTCCATCTCAAAAAAAAT
AAAAAATAAATAAAACAGAAAGGCAATGGCTATCCCAAATTGCTGAAGCCTTTGCTAAAAGCACAGACAG
AAAACAACAATATGGTGATGTTTGTGTTAAACAAATGTTATTGATTCTAGGACACAGGTCAGATGGCAAA
TAGTCAAACTCAAATGTTGATCCCAGGGGTGGCTGTATCATCACTAAGGCCAAATCACAACTTTGACTGG
TCTCATGGGAACACAGATCTTGGGTTTGGAAAGAGCACAGGCCTGGCCATGGGAATGGACACAGGGTAAT
TTTAAGATGACTAGAGGTTCCTCAAACCCTAAAAGTACCACTGAACCTTGTTTAAAGCTAAAATGGCTCC
TAAATGATAGACACGTTTCTCAGTTATCTCTGCAAACTGAAGATCCTATCAGCCTGTTCAGGGTCTCACA
GGCCTCTGGGTTTGTACATGATGTCCCTACAGTGGCGATGGGGACTGATGAGAGCCACCGTTCCCATTGG
AGGAGGGCAAGCTGCCACTGAGTGTCAGCTGCTGGAGTAAACATCCAATCACATGTGTACCTGTATCCTA
TGGATATTGGCTTCCCCTCATACAACTTGTTGTTCCTTTGTTTTCCATTCCTGACTATATTTGAATCTTT
CTTGGATTTCCAGGAAACATCTGACTGCTTTCCTATGCCACTTCATACCCAAGAGAAGCTCAGGGAGATC
CTTTAAGGTCCTTTATGCCCATTATGATAGTACATGAATAGGTAAATGTACCACTGCTCCCTGTACTTCT
TGGATAATGCACAAATAGTAAGACAGTCTCGCAACCAGCAGCCTAGACAGCCCAAATATGTGAAACCAAA
ACAGCTTGATCTTCCAGACCTGTTCTGACTCCTTGAGTCTTAAATCTCCAAGCCTCTTGAATGTTTCACC
CACAGCCTTCCCTTTGGGATCTGGCATGGGACAGGAAGGGCAGAGAAGGGCCTGGGGCTTTTCCTCAGGG
ATCCACCACAGTCTAGGCCAGTGGGTGGCAAACTTTTTCTGCAAAGGATCAGCTGGGAGATAGTTTCAAC
TTTGTAGTCTCGTCGCAGCTACTCTACTCGACTCTGCCACTGGAGTGTGAATGCAGTCATGAATAAAAGA
ATGAAAGTTTTCCAGTGAAACTTTAGTCACAAAAACAGTCTGCAGGCTGTGTCTGGCCCGTGGGCTTTGG
TTTGCTGGCCTGCGGGCTCCTAGAAGGCTCTGACTTTATCCCTCTATGAGGGAAAGCAGGTTTTGGGGTA
TTCTCCCAAATCTTTATACCTTATGTCTGTGTCTCTTTTTAAATTAAAAGTAAAATTTAGGCCAAAAAAA
AAAAAAAAAAAAAA

RNA

>LINC02864

UUCUCCUGCCUCAGCCUCCCGAGUAGCUGGAACUACAGGCGCCCGCCAUGAUGCCCGGCUCAUUUUCUGU
GUUUUCAGUAGAGACGGGGUUUCACUGUGUUAGCCAGGAAAGUCUUGAUCUCCUGACCUCGUGAUCCUGA
CCUCGGCCUCCCAAAGUGCUGGGAUUACAGGCGCGAGCCACCGCGCCCGACAGCAGUUAUGAAGUUCUAA
AAGCAAGUCUUAAUCAGGAAGUGUCCUUGAUCACCAACGGCUCGCCCAGGCAUGCUGGCUCUCUUCCACU
UCCACCUUCCACCAUGGGAUGACGCAGUAAGAAGGCCAUCAGUAGAUGCCAGUCCCUCAACCUUGAACUU
UCCAGACGCAGAACUUUAUGCCUCCAUUUUCCUCUGCUGCAUGGCCCCAGGAGAGAUUUUAAUUAGCUUU
CUAACCUUGGUCCAGAUUGCACAUGCAAAUGGUAGAGGAUGCAACACCCCCGCUUGUGGAGCUGCCGCUU
GUGUCUGGCAUGAAAAUUCACAAGAAGAGAGGAAAUACUGAGGAGAAAAUGGCAGAUUGUGUUUGCUGAA
UUUGAUUGACGAAGAAGUCACCAUGAAAAUCACAGUGAACCAUUUGGAAAGCAAACUGCCAAAAAAAUAAU
AGUUAGUCAUGCUCUCAGGCUGGUUGUUUUGGCUGUUGUGGGUUUCUUGCAUUUCCAGAUGAUUGCAAAG
AGCUGUUUCUCAAUUUCUGCAACAAGUGCCAGCUGAAAUUUUGGUACCAGUUUCAUUAAAUAUGUAGAAC
AAUUUGAGCCUGAGUGAGUUCUGUGGAGCCCAUACUACCUGCGAUUCAAGCCAGCAACCUUGGAAUCUCC
CUGAACAAUUUCUAUGGUGUCCAAUCACAUUUGCAAUCACUCCCCACACUCAGUAGGUUCUGCCCCCACA
UCAUUUCUUUUCUCUAUCCUCUUUCCCAGCUUUAUAAUAUGACUUAGUUCAAGCCCUAAUCACUGGAUUA
UUCAAAGUCCCUUAACAGGUUAAUUCCUACUGUGAUCAACACUUUAAAGGAAGGCAAUAGGGCCAGGCAU
GGUGGCUCACGGCUGUAAUCCCAGCACUUUGGGAGGCUGAGGCGAGUGGAUCACAAGGUCGGGAGUUUGA
GACCAGCCUGACUAACACAGUGAAACCCCGACUCUACUAAAAAUACAAAAAAUUAGCCAGGUGUGGUCGU
GGGUACCUGUAGUCCCAGCUACUAGGGAGGCUGAGGCAGGAGAAUGGCGUGAACCCGAGAGGCGGAGCUU
GCAGUGAGCCAAGAUUACGCCACUGCACUCCAGCCUGGGUGACGGAGCGAGACUCCAUCUCAAAAAAAAU
AAAAAAUAAAUAAAACAGAAAGGCAAUGGCUAUCCCAAAUUGCUGAAGCCUUUGCUAAAAGCACAGACAG

AAAACAACAAUAUGGUGAUGUUUGUGUUAAACAAAUGUUAUUGAUUCUAGGACACAGGUCAGAUGGCAAA
UAGUCAAACUCAAAUGUUGAUCCCAGGGGUGGCUGUAUCAUCACUAAGGCCAAAUCACAACUUUGACUGG
UCUCAUGGGAACACAGAUCUUGGGUUUGGAAAGAGCACAGGCCUGGCCAUGGGAAUGGACACAGGGUAAU
UUUAAGAUGACUAGAGGUUCCUCAAACCCUAAAAGUACCACUGAACCUUGUUUAAAGCUAAAAUGGCUCC
UAAAUGAUAGACACGUUUCUCAGUUAUCUCUGCAAACUGAAGAUCCUAUCAGCCUGUUCAGGGUCUCACA
GGCCUCUGGGUUUGUACAUGAUGUCCCUACAGUGGCGAUGGGGACUGAUGAGAGCCACCGUUCCCAUUGG
AGGAGGGCAAGCUGCCACUGAGUGUCAGCUGCUGGAGUAAACAUCCAAUCACAUGUGUACCUGUAUCCUA
UGGAUAUUGGCUUCCCCUCAUACAACUUGUUGUUCCUUUGUUUUCCAUUCCUGACUAUAUUUGAAUCUUU
CUUGGAUUUCCAGGAAACAUCUGACUGCUUUCCUAUGCCACUUCAUACCCAAGAGAAGCUCAGGGAGAUC
CUUUAAGGUCCUUUAUGCCCAUUAUGAUAGUACAUGAAUAGGUAAAUGUACCACUGCUCCCUGUACUUCU
UGGAUAAUGCACAAAUAGUAAGACAGUCUCGCAACCAGCAGCCUAGACAGCCCAAAUAUGUGAAACCAAA
ACAGCUUGAUCUUCCAGACCUGUUCUGACUCCUUGAGUCUUAAAUCUCCAAGCCUCUUGAAUGUUUCACC
CACAGCCUUCCCUUUGGGAUCUGGCAUGGGACAGGAAGGGCAGAGAAGGGCCUGGGGCUUUUCCUCAGGG
AUCCACCACAGUCUAGGCCAGUGGGUGGCAAACUUUUUCUGCAAAGGAUCAGCUGGGAGAUAGUUUCAAC
UUUGUAGUCUCGUCGCAGCUACUCUACUCGACUCUGCCACUGGAGUGUGAAUGCAGUCAUGAAUAAAAGA
AUGAAAGUUUUCCAGUGAAACUUUAGUCACAAAAACAGUCUGCAGGCUGUGUCUGGCCCGUGGGCUUUGG
UUUGCUGGCCUGCGGGCUCCUAGAAGGCUCUGACUUUAUCCCUCUAUGAGGGAAAGCAGGUUUUGGGGUA
UUCUCCCAAAUCUUUAUACCUUAUGUCUGUGUCUCUUUUUAAAUUAAAAGUAAAAUUUAGGCCAAAAAAA
AAAAAAAAAAAAAA

>NR_186852.1 Homo sapiens long intergenic non-protein coding RNA 2879 (LINC02879), transcript variant 1, long non-coding RNA
GGACTTATTCAGCAGAACCCCCAAGAAGATGCAGACTAAGAAATGCCAATGCTTTGATGGGTACAAGGAA
GAAATAGGACTAAGACAAGACAAATAATGGAGGACTTACATGGCAGAGGTAAATCACCCACATCTCTTTC
TCCAACTCCTTATGCTCGATGCTCAGCATTGAAGCCCTTTACCCCAGGGACAAATACAAGTTTGACAAAG
CCATCTGAGGAAAATAAGGCAACAAGGAGAAGTGCAGAGACAAGGTGGGGAAAGCCCCTTACAAAACCAT
CAGATCTCGTGAGAACTCATCCACATCACAAGAACAGCATGAAGAAACGGAACAAGGGGAATGCAATCTC
ACAGGATGGAAATAACCTGTGGTGAATTGTTGCCATCCAGATCCACTTTTAAGTCCACATGGTTCATTCA
TTTTGGACTAGATCCTGGTACAGCCCAGTGAACTGATATTCTTGAAATCAGGCACAGAGGCTCTGAAGTA
ATGCATTACATTTGCATCCATGATTTGCTTAAAATGTTCCATTTAGCCTTTCCTCCCAGGAAACAAAGCC
AGCAGTATTTGATTATTGAATAGCTCGTTTTGGATGCTTAGTTTGAGAAGATTTTATTAAGATTATGGGA
AACTTGAATTTTACAAAATGAATCATGAGTTATATTTCAGTTTTGATTTGTCTCCAAGGGTTGAAATACA
TAGAAGTCTAGGATACATTATATATTAGCTCTATTTTAGCATGCATTTAATGCAATCACAGGCAAAAATC
AAATGCTTTAAAGAAAAAGATTGCAGTTTTAGAAATGAAGGATGTGAAAAAGGACGAATCTATATAAATA
AAAGTGAACTTAGAGTAATAACATGCAATATAGTCTATGTAGATATGCATATTTTAATTGTATATTACTA
TTTGTCCCATCACAATAATTGGTTTAAACAATGGTGTATTTATATGTGTAAAACAAAGTTTGGTATGTGT
GTGCACACATATACACACAGAAACACACACACACACTTTTCTAATCAACTAACATTTAAGAAATACCACT
GAAGGAAATATTAATGACAGAAAGAGCTTTTCTGCACACAGAAAAGCACGCACACTCCTATGCAGCATAC
AAATAGGTAAGAGTACATGTTTTCCCTGAAAGAAGAGCAAGAGACCAACGGAATAAGCCCCTTTACTGGG
TTCCCATATGGCTGAATGGTCTCAATATCTCAATCTTTAGAACATACCTCAGCATCTCCACATCTCCTCC
TACCTCTGTCCTGTGTTGCTGGGACACAGGGGCAGGAAATGGATGCATCCCTTCCCATGCACGCCCAAAC
AGCTCCTCAACTCAATCTCAGCCCCATGCCTCTTCTATTCTTCTGTGAGGAAGTGGCTTCTCATGACCTA
TCCGAGAGCTCAGCACATGTTAAGTTCTCACACAGGCTTAAGCCAACATAATCGTCAACATTAAAAAGTA
ACAACATAATATTTGGGCGGATGATGTTTTCTGTTCATAATCAACAGTGACTTCTCAGAGTGGCCTTTTC
TGACCTCTCTATCTAAATAGTACTTTACGTTTTCTCTATCCCCTCCCTAAATTACTCTGGTTCATTTTTT
GTCAAAGAATTTTTACTGCTGAATAGTGTCATAGACCTATTCATTTATTTATTTTCTCACTCTTCCCAGT
AAAATATAAGATGCGTGGAGAAGAAGTTTGTTTTGTTCCCCTCTGCCATATGGGAAAGAACAGTGGTGGA
ATTTAGTAGATGCTCAATAAATATTTGTTGATTTAATAAAAAAA

RNA
>LINC02879
GGACUUAUUCAGCAGAACCCCCAAGAAGAUGCAGACUAAGAAAUGCCAAUGCUUUGAUGGGUACAAGGAA
GAAAUAGGACUAAGACAAGACAAAUAAUGGAGGACUUACAUGGCAGAGGUAAAUCACCCACAUCUCUUUC
UCCAACUCCUUAUGCUCGAUGCUCAGCAUUGAAGCCCUUUACCCCAGGGACAAAUACAAGUUUGACAAAG
CCAUCUGAGGAAAAUAAGGCAACAAGGAGAAGUGCAGAGACAAGGUGGGGAAAGCCCCUUACAAAACCAU
CAGAUCUCGUGAGAACUCAUCCACAUCACAAGAACAGCAUGAAGAAACGGAACAAGGGGAAUGCAAUCUC
ACAGGAUGGAAAUAACCUGUGGUGAAUUGUUGCCAUCCAGAUCCACUUUUAAGUCCACAUGGUUCAUUCA
UUUUGGACUAGAUCCUGGUACAGCCCAGUGAACUGAUAUUCUUGAAAUCAGGCACAGAGGCUCUGAAGUA
AUGCAUUACAUUUGCAUCCAUGAUUUGCUUAAAAUGUUCCAUUUAGCCUUUCCUCCCAGGAAACAAAGCC
AGCAGUAUUUGAUUAUUGAAUAGCUCGUUUUGGAUGCUUAGUUUGAGAAGAUUUUAUUAAGAUUAUGGGA
AACUUGAAUUUUACAAAAUGAAUCAUGAGUUAUAUUUCAGUUUUGAUUUGUCUCCAAGGGUUGAAAUACA
UAGAAGUCUAGGAUACAUUAUAUAUUAGCUCUAUUUUAGCAUGCAUUUAAUGCAAUCACAGGCAAAAAUC
AAAUGCUUUAAAGAAAAAGAUUGCAGUUUUAGAAAUGAAGGAUGUGAAAAAGGACGAAUCUAUAUAAAUA
AAAGUGAACUUAGAGUAAUAACAUGCAAUAUAGUCUAUGUAGAUAUGCAUAUUUUAAUUGUAUAUUACUA
UUUGUCCCAUCACAAUAAUUGGUUUAAACAAUGGUGUAUUUAUAUGUGUAAAACAAAGUUUGGUAUGUGU
GUGCACACAUAUACACACAGAAACACACACACACACUUUUCUAAUCAACUAACAUUUAAGAAAUACCACU
GAAGGAAAUAUUAAUGACAGAAAGAGCUUUUCUGCACACAGAAAAGCACGCACACUCCUAUGCAGCAUAC
AAAUAGGUAAGAGUACAUGUUUUCCCUGAAAGAAGAGCAAGAGACCAACGGAAUAAGCCCCUUUACUGGG
UUCCCAUAUGGCUGAAUGGUCUCAAUAUCUCAAUCUUUAGAACAUACCUCAGCAUCUCCACAUCUCCUCC
UACCUCUGUCCUGUGUUGCUGGGACACAGGGGCAGGAAAUGGAUGCAUCCCUUCCCAUGCACGCCCAAAC
AGCUCCUCAACUCAAUCUCAGCCCCAUGCCUCUUCUAUUCUUCUGUGAGGAAGUGGCUUCUCAUGACCUA
UCCGAGAGCUCAGCACAUGUUAAGUUCUCACACAGGCUUAAGCCAACAUAAUCGUCAACAUUAAAAAGUA
ACAACAUAAUAUUUGGGCGGAUGAUGUUUUCUGUUCAUAAUCAACAGUGACUUCUCAGAGUGGCCUUUUC
UGACCUCUCUAUCUAAAUAGUACUUUACGUUUUCUCUAUCCCCUCCCUAAAUUACUCUGGUUCAUUUUUU
GUCAAAGAAUUUUUACUGCUGAAUAGUGUCAUAGACCUAUUCAUUUAUUUAUUUUCUCACUCUUCCCAGU
AAAAUAUAAGAUGCGUGGAGAAGAAGUUUGUUUUGUUCCCCUCUGCCAUAUGGGAAAGAACAGUGGUGGA
AUUUAGUAGAUGCUCAAUAAAUAUUUGUUGAUUUAAUAAAAAAA

>NR_186640.1 Homo sapiens long intergenic non-protein coding RNA 2958 (LINC02958), long non-coding RNA
ACATTTTCTGTAGCTTTGCCCGAAGAGAGAAGCTGTTTTCTCTCAGAGATGGAACATGTTAGCTGGTTGC
CATGATGTCTTCATTTGTCCATCGCTGCCCCTCTGTGAGCAGGCAGCCATCAGGACAGAGAGTGGCATGC
TGGACAGGCCGACTCTGACAAGGAGCAAGATCTCCACGCCACTGCACACCAGCCTGAGAGACAGAGCACG
ACTCCATCTCAAAGAAAAAACAAAAAAGAAAAGAAAATGCAGATCTCTGGGCCCCACCCATACCTGGGGA
CTCAGAATCACCAGGGCAAGGTCTCATCCTTATGCGTCACATGTGTTTTGAGATCTCATTATCTCCTGGG
AGGCTGGAGTGCAGTGGTGTGATCATAGCTCACTGCAGTCTTGAACTCATGGACTCAAGAGATCCTTCCA
CCTCAGCTTCCCGAGCAGCTGGGACTACAGCTACGCAGACATTGTGCTGGAATTTCTATGCTGAAGAATT
AAGCTATATTGTTTGGCAAAAGAAGAAACTGAAAACAAGAAATCCATCCCTTTCTACCACTGCTATTATA
TCATCCACGTTTAAAAAAAAAAAGTTTGTATGTAGTTATATACAACAAGAAGTTTCTGCTTCGGATTTCT
CTGTTGGGCAGGGTGGTGGGAGGGATGGGGTCTCGAGTTTTTCCCAAGTGAAATCCTGGTAATCCTGCCT
GAGAGAATTCTTATGAAAGTTTCACTCACAATGAAAGCAAACTTCCAAGGTGGAGGAGGGTGAAGCCGGC
TGGCTGGACCGATCACTGAGGCCCAGTGCTGCGAAGAGAGTTCAGCACATTATTCCAACCCTTTCTGGGA
GCACGGCCCAGGCTTGGCACCGGTTTGGTGTTCTCGAGTGTGATGCATTCTGAAAACCTGGCTGCGTCCG
TGGCCCAGCTCCAGTTCTGCAGGGCTCAGCATCAGGCTGCCCCCTCTCCTAAGGTGTTTTGCAGGATCGC
CCCTCAGAGCTAAACACCCACCAGCTGACCACCTGTTCCAGGTGATGGGATTTTGGCTCAGATCAGCCTG
GATCAGGGTGGACCTCTACAAGCTGGCTTGCATGAATGTAAATGTCTGCTGCCCATCTTGAAGACCATCG
GCAAGCTTTGGTGGGTTCATATTCAGTGGCATTAGGGATTAAGGCAAACTTAAAATGATTCTGAGTGTAA
ACAGATACAGATATAAACTTTTGGGGGAACAATTTGGTAATAGCTAATAACAATCGTTAAAATGTTTATA
TCCTTTGACCTGATAGTGTTAGTTCTAAGAATTTACCTTAAGAAAATAAATAAATTTATATCCCATGA

RNA
>LINC02958
ACAUUUUCUGUAGCUUUGCCCGAAGAGAGAAGCUGUUUUCUCUCAGAGAUGGAACAUGUUAGCUGGUUGC
CAUGAUGUCUUCAUUUGUCCAUCGCUGCCCCUCUGUGAGCAGGCAGCCAUCAGGACAGAGAGUGGCAUGC
UGGACAGGCCGACUCUGACAAGGAGCAAGAUCUCCACGCCACUGCACACCAGCCUGAGAGACAGAGCACG
ACUCCAUCUCAAAGAAAAAACAAAAAAGAAAAGAAAAUGCAGAUCUCUGGGCCCCACCCAUACCUGGGGA
CUCAGAAUCACCAGGGCAAGGUCUCAUCCUUAUGCGUCACAUGUGUUUUGAGAUCUCAUUAUCUCCUGGG
AGGCUGGAGUGCAGUGGUGUGAUCAUAGCUCACUGCAGUCUUGAACUCAUGGACUCAAGAGAUCCUUCCA
CCUCAGCUUCCCGAGCAGCUGGGACUACAGCUACGCAGACAUUGUGCUGGAAUUUCUAUGCUGAAGAAUU
AAGCUAUAUUGUUUGGCAAAAGAAGAAACUGAAAACAAGAAAUCCAUCCCUUUCUACCACUGCUAUUAUA
UCAUCCACGUUUAAAAAAAAAAAGUUUGUAUGUAGUUAUAUACAACAAGAAGUUUCUGCUUCGGAUUUCU
CUGUUGGGCAGGGUGGUGGGAGGGAUGGGGUCUCGAGUUUUUCCCAAGUGAAAUCCUGGUAAUCCUGCCU
GAGAGAAUUCUUAUGAAAGUUUCACUCACAAUGAAAGCAAACUUCCAAGGUGGAGGAGGGUGAAGCCGGC
UGGCUGGACCGAUCACUGAGGCCCAGUGCUGCGAAGAGAGUUCAGCACAUUAUUCCAACCCUUUCUGGGA
GCACGGCCCAGGCUUGGCACCGGUUUGGUGUUCUCGAGUGUGAUGCAUUCUGAAAACCUGGCUGCGUCCG
UGGCCCAGCUCCAGUUCUGCAGGGCUCAGCAUCAGGCUGCCCCCUCUCCUAAGGUGUUUUGCAGGAUCGC
CCCUCAGAGCUAAACACCCACCAGCUGACCACCUGUUCCAGGUGAUGGGAUUUUGGCUCAGAUCAGCCUG
GAUCAGGGUGGACCUCUACAAGCUGGCUUGCAUGAAUGUAAAUGUCUGCUGCCCAUCUUGAAGACCAUCG
GCAAGCUUUGGUGGGUUCAUAUUCAGUGGCAUUAGGGAUUAAGGCAAACUUAAAAUGAUUCUGAGUGUAA
ACAGAUACAGAUAUAAACUUUUGGGGGAACAAUUUGGUAAUAGCUAAUAACAAUCGUUAAAAUGUUUAUA
UCCUUUGACCUGAUAGUGUUAGUUCUAAGAAUUUACCUUAAGAAAAUAAAUAAAUUUAUAUCCCAUGA

>LINC02974
GTTTAGAAATATTGTTTCCATATTACAAGTTTAAGGAAAAGTTTATCTGCCAAAATAATAACATGGATTTCTAGAGC
ACCCCATACCATAAGGAGGAGCCAGAATATTTTCCAGCTGAATTCCAGTCTTACTCATTTTCCTTGAATGATACTGA
AATCTAAATTATCCTTGAACACAAACAATGACAAAGAAATACAAATGTTCATGCAGTGTAATTTTCAACAATCAAAT
TGGCCAATCTGATTCTAATTCTAAGCTTTGCTGAGGATGCCAAGCAGAAGCCACGTGAGCACACTGCTGAACACCCA
GGATCTTTCCATGTATGGCAGCCTTCATGTGAGTTCTCTTCTCACCATACAATGTTGTGGATACAGAAGGGAAGGAA
TTGGATTTGGATGAGTACTGAGCACCGATCACAGGACAGAGCCTCAAAGTTGCTTTTTGGAATAAATTTCTTATAGT
A

RNA
>LINC02974
GUUUAGAAAUAUUGUUUCCAUAUUACAAGUUUAAGGAAAAGUUUAUCUGCCAAAAUAAUAACAUGGAUUUCUAGAGC
ACCCCAUACCAUAAGGAGGAGCCAGAAUAUUUUCCAGCUGAAUUCCAGUCUUACUCAUUUUCCUUGAAUGAUACUGA
AAUCUAAAUUAUCCUUGAACACAAACAAUGACAAAGAAAUACAAAUGUUCAUGCAGUGUAAUUUUCAACAAUCAAAU
UGGCCAAUCUGAUUCUAAUUCUAAGCUUUGCUGAGGAUGCCAAGCAGAAGCCACGUGAGCACACUGCUGAACACCCA
GGAUCUUUCCAUGUAUGGCAGCCUUCAUGUGAGUUCUCUUCUCACCAUACAAUGUUGUGGAUACAGAAGGGAAGGAA
UUGGAUUUGGAUGAGUACUGAGCACCGAUCACAGGACAGAGCCUCAAAGUUGCUUUUUGGAAUAAAUUUCUUAUAGU
A

>LINC02979
TTTAAAAAATCCAAACTATTTATTGAAAAGCTCTACTAAACAAAATTAGTTCAGACAAAGCAATAAGATGACGAATC
AAGCTCTCTCCAGAGATGAAAGAAAAGCCAGTCTCAAAGATGACTATAACTAACGCTGTTGCTGGAAAGACAAGGGC
ACCGAGCCAGCCCTGGGAGAGGCTCCACTTCTTCAAAGACAAAACGATTCCAAACGTGGCAGGACACAGTGCTCACC
CTACAGTGAACCTCTGGAATATGTTATTTTTTTAAATTCAGTTAATCTTTCTTCTTTTTTTTTTTGAGACAGAGTCT
CCTGTCGCCCAGGCTGGAGTGCAGGGGTGCGATCTCAGCTCACTGCAACCTCCACCTCCCGGGTTCAAATGATTCTC
GGGCCTCAGCCTCACAAGTAGCTGGAATTACAGGCATGCGCCACCATACCCAGCTAATTTTTGTATTTTTAGTAGAG
ATAGGGTTTCATTATGTTGTCCAGGCTGGGAGACAGAGCAAGACCCCAACTCAAACAAAAACAAA

RNA
>LINC02979
UUUAAAAAAUCCAAACUAUUUAUUGAAAAGCUCUACUAAACAAAAUUAGUUCAGACAAAGCAAUAAGAUGACGAAUC
AAGCUCUCUCCAGAGAUGAAAGAAAAGCCAGUCUCAAAGAUGACUAUAACUAACGCUGUUGCUGGAAAGACAAGGGC
ACCGAGCCAGCCCUGGGAGAGGCUCCACUUCUUCAAAGACAAAACGAUUCCAAACGUGGCAGGACACAGUGCUCACC
CUACAGUGAACCUCUGGGAAUAUGUUAUUUUUUUAAAUUCAGUUAAUCUUUCUUCUUUUUUUUUUUGAGACAGAGUCU
CCUGUCGCCCAGGCUGGAGUGCAGGGGUGCGAUCUCAGCUCACUGCAACCUCCACCUCCCGGGUUCAAAUGAUUCUC
GGGCCUCAGCCUCACAAGUAGCUGGAAUUACAGGCAUGCGCCACCAUACCCAGCUAAUUUUUGUAUUUUUAGUAGAG
AUAGGGUUUCAUUAUGUUGUCCAGGCUGGGAGACAGAGCAAGACCCCAACUCAAACAAAAACAAA

>XR_935478.3 PREDICTED: Homo sapiens long intergenic non-protein coding RNA 3035 (LINC03035), ncRNA
CCACTCAACAATCCCACTGGACCACATGAAGGCTGCATCCTAACTAATGGACCTCTTGGAGAAGGATTTT
ATTTGATAACTTGGTTAAAGAGCCTGAAATGTTTCATTATCAATTGCTTCATTGTTGTTGGTATAGATTA
ATCACCAGTACTTTCATTCATTTATTTATCTTCTTGAAACTCTGATTTTCCAATAGGTACAACTCACTTT
TTCTGCTTTAATTTTCACTAGTTCTCCCAACCATTAGTTATAAAGAATCATCTAGTTATGGAGAAATCAC
TAACCCTTTGGGAATCCTGTCCCATAGCTGAAATCTACATTCCTCCAATTGTCTGGCAAGTTTTAAAGTT
GTTATTTCTTTGTTTGTTTCAACAGATTTGTCCCTAATCATGTGGAAAGGGCTGCTAAAATACAGAATGT
ACTTACTCCAAGTGAGATTCCCAGTTCACAGATACAGTAGTAAACCTAATTTGCAAATAAGCCACAGTCA
TACACATAATATTTCACACACATATTAATATTGTTAATATTAATATTAAGCAGTTATTTGCAAAACAAAT
TGGTACAATTTCTTTATAAGATTGTTTTACAGGATTTTATCAGCTGTTGCAAGAAAAGAACTGAGCTGCA
AAAGGGATTGGAGTTGTATCTCATTACTCTTCCTTAGGGAGGGTATTGAATTTGGTTAAAAATCAGTCCA
CTCCTTTAGAAAGTATGTTGCAGAGAGACAAATGCAAAATGCCAGATGGTGGAGTTGAAACAGAGACCTC
ATGGAAATCCGGACACAGACATGCGCAGAGGGAGAAGGCTATGTGAGGAAGAAGGCAGTGGTTGGAGTGA
TGATTCTCCAAGCCTAGGAAGGCCAGTGACTGGCAGCAAAGCACCAGAAACGAGGGGAAGAAACATGGAA
CCGATTCCTTCTCAGGCCTCAGGAGGAACCAACCCAGACGAACCTTGATCTCAGGCTCTAGCCTCTGTTG
CAATAAAACATTTTATTGTTGAA

RNA
>LINC03035
CCACUCAACAAUCCCACUGGACCACAUGAAGGCUGCAUCCUAACUAAUGGACCUCUUGGAGAAGGAUUUU
AUUUGAUAACUUGGUUAAAGAGCCUGAAAUGUUUCAUUAUCAAUUGCUUCAUUGUUGUUGGUAUAGAUUA
AUCACCAGUACUUUCAUUCAUUUAUUUAUCUUCUUGAAACUCUGAUUUUCCAAUAGGUACAACUCACUUU
UUCUGCUUUAAUUUUCACUAGUUCUCCCAACCAUUAGUUAUAAAGAAUCAUCUAGUUAUGGAGAAAUCAC
UAACCCUUUGGGAAUCCUGUCCCAUAGCUGAAAUCUACAUUCCUCCAAUUGUCUGGCAAGUUUUAAAGUU
GUUAUUUCUUUGUUUGUUUCAACAGAUUUGUCCCUAAUCAUGUGGAAAGGGCUGCUAAAAUACAGAAUGU
ACUUACUCCAAGUGAGAUUCCCAGUUCACAGAUACAGUAGUAAACCUAAUUUGCAAAUAAGCCACAGUCA
UACACAUAAUAUUUCACACACAUAUUAAUAUUGUUAAUAUUAAUAUUAAGCAGUUAUUUGCAAAACAAAU
UGGUACAAUUUCUUUAUAAGAUUGUUUUACAGGAUUUUAUCAGCUGUUGCAAGAAAAGAACUGAGCUGCA
AAAGGGAUUGGAGUUGUAUCUCAUUACUCUUCCUUAGGGAGGGUAUUGAAUUUGGUUAAAAAUCAGUCCA
CUCCUUUAGAAAGUAUGUUGCAGAGAGACAAAUGCAAAAUGCCAGAUGGUGGAGUUGAAACAGAGACCUC
AUGGAAAUCCGGACACAGACAUGCGCAGAGGGAGAAGGCUAUGUGAGGAAGAAGGCAGUGGUUGGAGUGA
UGAUUCUCCAAGCCUAGGAAGGCCAGUGACUGGCAGCAAAGCACCAGAAACGAGGGGAAGAAACAUGGAA
CCGAUUCCUUCUCAGGCCUCAGGAGGAACCAACCCAGACGAACCUUGAUCUCAGGCUCUAGCCUCUGUUG
CAAUAAAACAUUUUAUUGUUGAA

>NR_148972.1 Homo sapiens long intergenic non-protein coding RNA 3069 (LINC03069), long non-coding RNA

AATTAGCGTGGGAGGAAGAGACAGTGAAGCAGCCCCTTAAAAATGGTCTTTCGGGTGGAAACACTTTATT
TGGGAAGCAAAGTGCAAGATTCAGCAATAGAAACCACTCAGTGTCCTTGGTGAATGATTTGATCCTGGCC
ATCAGAACCTTTACTTCAGCCAGCAATAAACTCTTAGAAACAGAAGCTTCGAGGTATGTACTCCTGTTCT
GCACGAACAAGTTTTCTGGTTGAACATGCTGAACCTCTTAAAAAATTCAGAACAAGTTTTCTCAGAGTTT
ACAAAGGGATTTCAATCACATGAAGAGAGGAAAACACACTGAGTCGTAACAGTGACATCAAAATACTTTT
GAAGTTGACTGAAAATGTGGTTGCTTTTGTTTGAGAATGGCAAATTAAAGAATTGGATATTGGGTTCTTA
TCTCAGAAGAAACAAAGGAGGAGACCCTGAAAAACTGAACATGAAGAGGAAGAAGTGGAGTTATGCAACT
CTTCATAAGAAAATCTCCATCTGTTTGGAGAAGTACTGCGTCCTTCCGGATTATCTTCTACACTATGCCT
TATTGTTATCTGGCTGGTTCCACCACCGGTCAGAAGAATGAAAATTGCAGATACCTATTCTGTGTGTTTC
TCTCCAGAATGGTGGATGTTAGAGACCCTACTAAGGATTTGTGGACTCACTAACGTAGTCTCTAAAAGGT
TGGACATCCCTACCCAAGGCTCAAATGGTAACAGGTCCATGCCTTACTTGTGTCAGTTCTCTTATTGAAA
GAGCATCTCCTAACCATCCTACCAATAAGGGTCTTACTCTGTTGCCCTGGCTGGAGTGCAGTGGCCCAAT
CATAGCTCACTGCAGCCTTGACTTCCCAGCCTCAGTTGATCCTCCTACCTTAATCTCCCGAGTAGCTGGG
ACTGCAGGTTTACTTATACTAAGATTTTCTTCTGCCTCTGCCAACCCTGAGACAGCAAGACCAATTCCTC
CTCTCCCCTGTCCTCCTCCTCGCCTACTCCACATGAAGACAACGAGGATGAAGACCTTCATGATGATCCA
TTTCCACTTAATGAACAAACTTCAGGAAATTATTCAAGAAGAGAGGCATGGTGGTATGCTGGAAAGTCTG
CTGAGCTTGAAAGTAGGTAACCCAGGCTCCTGTTCCTGTTCTCATTGATGAGTTCCCTGACTTCAGGTGA
GTCACAGCTTCTCTGGACTTTCATGTCCTTTTGCAAAGTGAGTCTGATCATTTCTAGGATCTTTTCCAGC
CCTGAGATTGTATGAGCTGCAATAGCTAAGAGATATGGGAAAGTGGGTCATTGGAAAGTTGTTTTCCTAA
CTCCACAGGCAACAATGTGCCTCATTCTTAGTCATCAAAGGCAAATACGAAGGGATGGGAAGCACTCTCT
TCACTGTCCATTTGCAAGCAGAATCACTCTTCACTTGATTGTCCAGTGTCTGTATAGTCTCTCACAGAGC
CCAGCACATGTGGGCATTCTGTAAATGGCCATTGAGAACTGCCAGGTCTGTGGCTGCTACAGAGCACAAT
GATGATGATAATGGTGTTGATGATCATGATGATGATGGGCTGGTGTATCGAGGCACAGACCCAAGGAAGG
CTAATGAGAATGTTTCTAGGGAAGCTTCCTGCATGTCACTCTTCTTCCTTCTACTTCCTCTAGGTTCATC
TTCCCTGACTAACAGGGAGCCAGGGCTATAATAACGGGAAGGAAGTCATCATGTGATTCTACCTAGAAAC
GCAGGCATCAACTTGAAATGTTTTCTCTTTCTCTTGTAACTCTGGAATATGGCTCCTTGGATTGGAGGTG
GCAAATACCACGGGTTAAACAATGTCATTCTGAGCTCTTTTAGTACTGAATAATGCAATATATACCATAT
TCTACCCTCTTAAAGAAGGAGTGTATCAGCTATGTGCCTGATATGGTTTGGCTCTATGTCCCCACCCAAA
TCTCATCTTGTAGCACCCATAATTCCCAGGTTTTGTGGGAGGGACCCGGTGGGAGATGATTGAATCATGG
GGGCTGGTCTTTCCTGTGCTGGTCTTCTGATAGTAAATGGGTCTCACAAGATCTGATGGTTTTTGAAAAC
AGGAGTTTCTCTGCATGAGCTCTCTCTGCCTGCTGCCATCCACTTAAGATGTGACTTGCTCGTCCATCGC
CTTCTGCCATGATTGTGAGGCCTCCCCAGCCACGTGGAACTTAGCTCAGGACAGCAACCAGACCTGAGAT
TCTTTTCAACTTATACGCTGTTGATGAATGTGGTTTCTGGATGAAAAACTGAGAACAGACTGACCCTCTG
GCCACAGCTACTCGCGTCAGGGACGCATAGTTGCAGGTGCATGCTGTCTGGTTTGGTTTGCCAAGTCAAA
ATAGTTGTGTTTCCCGCTGTGGTGAGAGTTTCCAACTTCAGACGGGGCAAGAGAAATTCTAAACAGTTTG
CCCTTTGCAGATGTATTGAAATAGTACTGAAATTTACTGATTTTCCCTCATGTTTTTTTTCCGTGCTTTT
ATCACTACCCTGAAAATAAACAAGGAGAAAAGGCAAGC

RNA

>LINC03069

AAUUAGCGUGGGAGGAAGAGACAGUGAAGCAGCCCCUUAAAAAUGGUCUUUCGGGUGGAAACACUUUAUU
UGGGAAGCAAAGUGCAAGAUUCAGCAAUAGAAACCACUCAGUGUCCUUGGUGAAUGAUUUGAUCCUGGCC
AUCAGAACCUUUACUUCAGCCAGCAAUAAACUCUUAGAAACAGAAGCUUCGAGGUAUGUACUCCUGUUCU
GCACGAACAAGUUUUCUGGUUGAACAUGCUGAACCUCUUAAAAAAUUCAGAACAAGUUUUCUCAGAGUUU
ACAAAGGGAUUUCAAUCACAUGAAGAGAGGAAAACACACUGAGUCGUAACAGUGACAUCAAAAUACUUUU
GAAGUUGACUGAAAAUGUGGUUGCUUUUGUUUGAGAAUGGCAAAUUAAAGAAUUGGAUAUUGGGUUCUUA
UCUCAGAAGAAACAAAGGAGGAGACCCUGAAAAACUGAACAUGAAGAGGAAGAAGUGGAGUUAUGCAACU
CUUCAUAAGAAAAUCUCCAUCUGUUUGGAGAAGUACUGCGUCCUUCCGGAUUAUCUUCUACACUAUGCCU
UAUUGUUAUCUGGCUGGUUCCACCACCGGUCAGAAGAAUGAAAAUUGCAGAUACCUAUUCUGUGUGUUUC
UCUCCAGAAUGGUGGAUGUUAGAGACCCUACUAAGGAUUUGUGGACUCACUAACGUAGUCUCUAAAAGGU
UGGACAUCCCUACCCAAGGCUCAAAUGGUAACAGGUCCAUGCCUUACUUGUGUCAGUUCUCUUAUUGAAA
GAGCAUCUCCUAACCAUCCUACCAAUAAGGGUCUUACUCUGUUGCCCUGGCUGGAGUGCAGUGGCCCAAU
CAUAGCUCACUGCAGCCUUGACUUCCCAGCCUCAGUUGAUCCUCCUACCUUAAUCUCCCGAGUAGCUGGG
ACUGCAGGUUUACUUAUACUAAGAUUUUCUUCUGCCUCUGCCAACCCUGAGACAGCAAGACCAAUUCCUC
CUCUCCCCUGUCCUCCUCCUCGCCUACUCCACAUGAAGACAACGAGGAUGAAGACCUUCAUGAUGAUCCA
UUUCCACUUAAUGAACAAACUUCAGGAAAUUAUUCAAGAAGAGAGGCAUGGUGGUAUGCUGGAAAGUCUG
CUGAGCUUGAAAGUAGGUAACCCAGGCUCCUGUUCCUGUUCUCAUUGAUGAGUUCCCUGACUUCAGGUGA
GUCACAGCUUCUCUGGACUUUCAUGUCCUUUUGCAAAGUGAGUCUGAUCAUUUCUAGGAUCUUUUCCAGC
CCUGAGAUUGUAUGAGCUGCAAUAGCUAAGAGAUAUGGGAAAGUGGGUCAUUGGAAAGUUGUUUUCCUAA
CUCCACAGGCAACAAUGUGCCUCAUUCUUAGUCAUCAAAGGCAAAUACGAAGGGAUGGGAAGCACUCUCU

UCACUGUCCAUUUGCAAGCAGAAUCACUCUUCACUUGAUUGUCCAGUGUCUGUAUAGUCUCUCACAGAGC
CCAGCACAUGUGGGCAUUCUGUAAAUGGCCAUUGAGAACUGCCAGGUCUGUGGCUGCUACAGAGCACAAU
GAUGAUGAUAAUGGUGUUGAUGAUCAUGAUGAUGAUGGGCUGGUGUAUCGAGGCACAGACCCAAGGAAGG
CUAAUGAGAAUGUUUCUAGGGAAGCUUCCUGCAUGUCACUCUUCUUCCUUCUACUUCCUCUAGGUUCAUC
UUCCCUGACUAACAGGGAGCCAGGGCUAUAAUAACGGGAAGGAAGUCAUCAUGUGAUUCUACCUAGAAAC
GCAGGCAUCAACUUGAAAUGUUUUCUCUUUCUCUUGUAACUCUGGAAUAUGGCUCCUUGGAUUGGAGGUG
GCAAAUACCACGGGUUAAACAAUGUCAUUCUGAGCUCUUUUAGUACUGAAUAAUGCAAUAUAUACCAUAU
UCUACCCUCUUAAAGAAGGAGUGUAUCAGCUAUGUGCCUGAUAUGGUUUGGCUCUAUGUCCCCACCCAAA
UCUCAUCUUGUAGCACCCAUAAUUCCCAGGUUUUGUGGGAGGGACCCGGUGGGAGAUGAUUGAAUCAUGG
GGGCUGGUCUUUCCUGUGCUGGUCUUCUGAUAGUAAAUGGGUCUCACAAGAUCUGAUGGUUUUUGAAAAC
AGGAGUUUCUCUGCAUGAGCUCUCUCUGCCUGCUGCCAUCCACUUAAGAUGUGACUUGCUCGUCCAUCGC
CUUCUGCCAUGAUUGUGAGGCCUCCCCAGCCACGUGGAACUUAGCUCAGGACAGCAACCAGACCUGAGAU
UCUUUUCAACUUAUACGCUGUUGAUGAAUGUGGUUUCUGGAUGAAAAACUGAGAACAGACUGACCCUCUG
GCCACAGCUACUCGCGUCAGGGACGCAUAGUUGCAGGUGCAUGCUGUCUGGUUUGGUUUGCCAAGUCAAA
AUAGUUGUGUUUCCCGCUGUGGUGAGAGUUUCCAACUUCAGACGGGGCAAGAGAAAUUCUAAACAGUUUG
CCCUUUGCAGAUGUAUUGAAAUAGUACUGAAAUUUACUGAUUUUCCCUCAUGUUUUUUUCCGUGCUUUU
AUCACUACCCUGAAAAUAAACAAGGAGAAAAGGCAAGC

>XR_001753453.3 PREDICTED: Homo sapiens long intergenic non-protein coding RNA 3092 (LINC03092), transcript variant X1, ncRNA
CTCCTCTGTGTCGCCTCAGCAACTTGTCCATATCATTGTTACAGCACTTACCACACAGTTGTAAGACGCC
CACAACAGCCTATGAAGAATAACTAGAGGTCTTCATATCAGAGGCAGAAGGTACTGCAAGCTTACTTTCC
AACGTGGCTGCATCGTTTCACATTCCCACCAGCAGTATGAGGTTTCCAATTTCTCTGCATCCTCATCAAA
ACTTGTTATTATCTGTCTTTTTTATTTTAGACGTTCTAGAAGGTGTGGAGTGGTATCTCATTGTGGTATT
GATTTGCATTTCCTTAAGGACTAATGCTTTTGAACATTTTTTCATGTGCTTATTGGCCATTTGTATATCT
TCTCTGGAGAAATGTTTATTCAAATCCTTTGTCCATTTTTTAATTGGATTGTCTTTTTATTGTTGCATGG
TATAAGTTCTCTATATATTCTGGACACAAGTCTCTTATTAGTTATACCATTTGCAAATATTTTCTCCCAT
TCTGTGCGTTGTCTTTTAACCTTTCTAATGTGTCCATTGAAACACAAAAGTTTTTAATTTTCACAAAGTT
CAATTTATCTACTTTTTTCTTTTGTCACTTATTTGTATCATATCTGAGAAACCATTACCTAATCCAAGGT
CAGGAAGGATTACTCCTATGTTCTCTTTTGAGAGTTTTATTGTTTTTACTCTTATATTTAAGTTGAATAT
CCATTTTGAGTTAGTTAATTTTTGTATATAGTATGACATAGGATCGAGAGGATCTTGTTAATCACTTTTT
CCTGATATACATCAGCTTCAAATGTGAAGCTATAAATCTCCCTGTAGGTTTCCTGAAAGCCCTCATTATA
TAAAGCAACTTCTGTGACAAATTTGACTTGATTAATTAATTAATTAGAGACACAGTCTTGCTCTGTCCCC
CAGACTGGAGTGCAGTGGTGCCATCTTGGATCACAGCAACCTTCAACTCTCAGGTTCAAGCAATTCTAGT
GCCTCAGCTTCCCAAGTAGCTAGGATCACAGGTGTGCACCAGCATGCCTGGCTACTTTTTGTATTTTTAG
TAGAGGCATGTTTTTGCCATGTTAGCCAGACTGGTCTCTAACTCCTGGCCTCAAGTCTTCCCCTGCCTTG
GCCAACCAAAGTGCTGGGATTACTGGTGTGAACCACCGTGCCTAGTCATGATAATTTATTGAGTGGAGTT
CTGTTCAAGATGATGGAGGGCTAACAGCAGCAAAAGCCAGGCTCACTGGCTCTTGCCTGTAATCCTAGCA
CTTTGGGAGGCCTAGTTGGTTAGGTAGATCACCTGAGCTCAGGAGTTCGAGACCAGCCTAGCCAACATGG
CGAAACCCTGTCTCTACTAAAAATACAAAAATTAGCCGGGCATAGTGGTGGATGCTAATAATCCCAGCTA
CTCTGGAGGCTGAGACAGGAGAATTGCTTGAACCTGGGGGCGGAGGTTGCAGTGAGCTGAGATTGCGCCA
CTTCACTTCAGCCTGAGTGACAGAGTGAAATTCCATCTTAAAAGAAAAAAAAAAAAAAAAGGAAAGAAAG
CAGCAAAGAGAATTTTTTGGCAAGATTATGAGCCAATTTTAAGAGCTTTTATTACATTTCAAATGTGATT
TTAAATTCTCAAAGTTGGACAGATAGCAGGGAAAGAATGGTCACAGACTTTTCCCTAATATTTTAAGTTG
CTTCAAACCAGTTATTAAATCTTATATGTATTTTTTAAAGACTTATTCCACAGGGGTTTGATTAATTTAC
ACAGACTTCTTCCAAAACTGTATCTAAACCACATTGTTGATATCTAAGAAACTCTATGAGCTTCTTCAAA
AGCCCTCTTAAAAATACAACAAAATCATGTTAGCAACTCCAGATTCCAAGTTGAAATCCTCCCGCTTTAG
TATAAGAACCATCAGTTTTGAATGAGTCTGGATACGAAAACCTATTTTCCCATTTTGAGGAGAAAATCCC
TTTTTAGAGACTCTTTCTTGAATGGATATTTGTTATAACATATTTGGGCTTAGGGCTAAAACCTTTGGTT
TGTAAGCTGAGGTTTTCATTTGCACTTACCATCAACAGCGTATAAGTTGAAAAGAATCTCAGGTCTGGTT
GCTGTCCTGAGCTACTGCAAAGAAGAAACCACTGAGAATAAGAGCATTTCAATTATTGTACATCACATTT
GCTATTTGCTGCCGTTATTCTTCTATTAAAGGAGGATAATCAACTGATATGATATTGTAGATCTCAGTGC
CTAGTGCATTGTAGGTCCTCAATATATGTTGATTCTTCTTTTCTTTACATATTTTTGAGGTACAGAAAGA
AGTTCTTAAAATGGTTTTTAACTGTAATCATTTTGGAAAATTAGCTATGAAGATGGAAATTTGTGAACAA
AGGAGAACTTGCAAATGCCATTTTACTGAGCATGTTACTGAATGGTTATATAAGGAGTAAATATTCTCTC
AGCCTTAGTAAGTAAATATGAACCAATAAGAACACAGGTGAGGACTCTTTTCCTAACCAGAATAGATGGC
TAAGGTTTACAGGGCAATAAATTAGACTATTTGTATCCAAAGCAATATTGTTGTCACCACCAATTGTAAT
AAGCATTTTTATAATTTATGCCTTTAATGTGTTTATTATGAAAAAGCTTCTAAGTATATTGCACAAGTGG
TAATCTGCTCCAGAACTGGAAATTTGGGAGACTTGGTTTTTGGACTCAGCCTATTCACTATTATTTGGTT
GATAGGTTTATTATTATTATTATTATTATTTTTAAAGGCCTGAGACAGTAGAGACTTTCTAATAAAATA
TGCTGCAGTGATGAAAATTA

RNA
>LINC03092
CUCCUCUGUGUCGCCUCAGCAACUUGUCCAUAUCAUUGUUACAGCACUUACCACACAGUUGUAAGACGCC
CACAACAGCCUAUGAAGAAUAACUAGAGGUCUUCAUAUCAGAGGCAGAAGGUACUGCAAGCUUACUUUCC
AACGUGGCUGCAUCGUUUCACAUUCCCACCAGCAGUAUGAGGUUUCCAAUUUCUCUGCAUCCUCAUCAAA
ACUUGUUAUUAUCUGUCUUUUUUAUUUUAGACGUUCUAGAAGGUGUGGAGUGGUAUCUCAUUGUGGUAUU
GAUUUGCAUUUCCUUAAGGACUAAUGCUUUUGAACAUUUUUUCAUGUGCUUAUUGGCCAUUUGUAUAUCU
UCUCUGGAGAAAUGUUUAUUCAAAUCCUUUGUCCAUUUUUUAAUUGGAUUGUCUUUUUAUUGUUGCAUGG
UAUAAGUUCUCUAUAUAUUCUGGACACAAGUCUCUUAUUAGUUAUACCAUUUGCAAAUAUUUUCUCCCAU
UCUGUGCGUUGUCUUUUAACCUUUCUAAUGUGUCCAUUGAAACACAAAAGUUUUUAAUUUUCACAAAGUU
CAAUUUAUCUACUUUUUUCUUUUGUCACUUAUUUGUAUCAUAUCUGAGAAACCAUUACCUAAUCCAAGGU
CAGGAAGGAUUACUCCUAUGUUCUCUUUUGAGAGUUUUAUUGUUUUUACUCUUAUAUUUAAGUUGAAUAU
CCAUUUUGAGUUAGUUAAUUUUUGUAUAUAGUAUGACAUAGGAUCGAGAGGAUCUUGUUAAUCACUUUUU
CCUGAUAUACAUCAGCUUCAAAUGUGAAGCUAUAAAUCUCCCUGUAGGUUUCCUGAAAGCCCUCAUUAUA
UAAAGCAACUUCUGUGACAAAUUUGACUUGAUUAAUUAAUUAAUUAGAGACACAGUCUUGCUCUGUCCCC
CAGACUGGAGUGCAGUGGUGCCAUCUUGGAUCACAGCAACCUUCAACUCUCAGGUUCAAGCAAUUCUAGU
GCCUCAGCUUCCCAAGUAGCUAGGAUCACAGGUGUGCACCAGCAUGCCUGGCUACUUUUUGUAUUUUUAG
UAGAGGCAUGUUUUUGCCAUGUUAGCCAGACUGGUCUCUAACUCCUGGCCUCAAGUCUUCCCCUGCCUUG
GCCAACCAAAGUGCUGGGAUUACUGGUGUGAACCACCGUGCCUAGUCAUGAUAAUUUAUUGAGUGGAGUU
CUGUUCAAGAUGAUGGAGGGCUAACAGCAGCAAAAGCCAGGCUCACUGGCUCUUGCCUGUAAUCCUAGCA

CUUUGGGAGGCCUAGUUGGUUAGGUAGAUCACCUGAGCUCAGGAGUUCGAGACCAGCCUAGCCAACAUGG
CGAAACCCUGUCUCUACUAAAAAUACAAAAAUUAGCCGGGCAUAGUGGUGGAUGCUAAUAAUCCCAGCUA
CUCUGGAGGCUGAGACAGGAGAAUUGCUUGAACCUGGGGGCGGAGGUUGCAGUGAGCUGAGAUUGCGCCA
CUUCACUUCAGCCUGAGUGACAGAGUGAAAUUCCAUCUUAAAAGAAAAAAAAAAAAAAAAAGGAAAGAAAG
CAGCAAAGAGAAUUUUUUGGCAAGAUUAUGAGCCAAUUUUAAGAGCUUUUAUUACAUUUCAAAUGUGAUU
UUAAAUUCUCAAAGUUGGACAGAUAGCAGGGAAAGAAUGGUCACAGACUUUUCCCUAAUAUUUUAAGUUG
CUUCAAACCAGUUAUUAAAUCUUAUAUGUAUUUUUUAAAGACUUAUUCCACAGGGGUUUGAUUAAUUUAC
ACAGACUUCUUCCAAAACUGUAUCUAAACCACAUUGUUGAUAUCUAAGAAACUCUAUGAGCUUCUUCAAA
AGCCCUCUUAAAAAUACAACAAAAUCAUGUUAGCAACUCCAGAUUCCAAGUUGAAAUCCUCCCGCUUUAG
UAUAAGAACCAUCAGUUUUGAAUGAGUCUGGAUACGAAAACCUAUUUUCCCAUUUUGAGGAGAAAAUCCC
UUUUUAGAGACUCUUUCUUGAAUGGAUAUUUGUUAUAACAUAUUUGGGCUUAGGGCUAAAACCUUUGGUU
UGUAAGCUGAGGUUUUCAUUUGCACUUACCAUCAACAGCGUAUAAGUUGAAAAGAAUCUCAGGUCUGGUU
GCUGUCCUGAGCUACUGCAAAGAAGAAACCACUGAGAAUAAGAGCAUUUCAAUUAUUGUACAUCACAUUU
GCUAUUUGCUGCCGUUAUUCUUCUAUUAAAGGAGGAUAAUCAACUGAUAUGAUAUUGUAGAUCUCAGUGC
CUAGUGCAUUGUAGGUCCUCAAUAUAUGUUGAUUCUUCUUUUCUUUACAUAUUUUUGAGGUACAGAAAGA
AGUUCUUAAAAUGGUUUUUAACUGUAAUCAUUUUGGAAAAUUAGCUAUGAAGAUGGAAAUUUGUGAACAA
AGGAGAACUUGCAAAUGCCAUUUUACUGAGCAUGUUACUGAAUGGUUAUAUAAGGAGUAAAUAUUCUCUC
AGCCUUAGUAAGUAAAUAUGAACCAAUAAGAACACAGGUGAGGACUCUUUUCCUAACCAGAAUAGAUGGC
UAAGGUUUACAGGGCAAUAAAUUAGACUAUUUGUAUCCAAAGCAAUAUUGUUGUCACCACCAAUUGUAAU
AAGCAUUUUUAUAAUUUAUGCCUUUAAUGUGUUUAUUAUGAAAAAGCUUCUAAGUAUAUUGCACAAGUGG
UAAUCUGCUCCAGAACUGGAAAUUUGGGAGACUUGGUUUUUGGACUCAGCCUAUUCACUAUUAUUUGGUU
GAUAGGUUUAUUAUUAUUAUUAUUAUUAUUUUUUAAAGGCCUGAGACAGUAGAGACUUUCUAAUAAAAUA
UGCUGCAGUGAUGAAAAUUA

>NR_187441.1 Homo sapiens long intergenic non-protein coding RNA 3110 (LINC03110), transcript variant 1, long non-coding RNA
TACAGTGTACCACACCAAGGAACTCCCACACTCTGAATGAACAGCCCAGTGATCAGGAGCTTTGAATCAATTCTTGG
CACCTAACAGTTTGTGAAGAGTCTGCTAAACTCCCTCTATCAGAAATCCCAGAGGACTGAGAACAGGTCATCCTTGG
AGCTGTATCTGAATGGAGTCAGGATCTGGAAGCCCAAGTCTCCTTGTCAATGTCTGCTTGGTTCCCAAGGACACCTG
TAAATGGAAGAGGGGCAT

RNA
>LINC03110
UACAGUGUACCACACCAAGGAACUCCCACACUCUGAAUGAACAGCCCAGUGAUCAGGAGCUUUGAAUCAAUUCUUGG
CACCUAACAGUUUGUGAAGAGUCUGCUAAACUCCCUCUAUCAGAAAUCCCAGAGGACUGAGAACAGGUCAUCCUUGG
AGCUGUAUCUGAAUGGAGUCAGGAUCUGGAAGCCCAAGUCUCCUUGUCAAUGUCUGCUUGGUUCCCAAGGACACCUG
UAAAUGGAAGAGGGGCAU

>NR_186639.1 Homo sapiens long intergenic non-protein coding RNA 3111 (LINC03111), long non-coding RNA
GCTAGCACTTGTTTGCTGAACTGTGGAAGAACTGCAAGGTCACGCACATTATTCATGATAAAGCAACTGA
GCTGACAAGTCTGTGCTTAATTCAAGAATAACCAGTAGGAGAGAGAGAAAACACAAGGAGTTAACCACCA
TCGCCGGTGCTCTCTCAAAGGAAGAGGGGTTAACAGATGAATTCGCAAAGACAGCTCTTCTGGCCAAGAA
TATGGATCATTTGCAGCACAGAGGCAAGTTGATATATGTAGCATCATGACAGTGAAGCAAATTCCACAAA
AACTGCTAATGGACGGAGAAATTTCATGGAGAAAAGCTAGCAGACAGAGCTGGAATAAAACAATGGCAGT
GTTCCTTGCTCAAAGAAGCAGTCCCGGGCTGTTCTTTGCCCAGGGATATCTTCCCAAGATGACCAGAAAT
GGCTTCATGACCTAAGGGTGGACAGCAGTGTTCTATTGTACCCACATCATCATCGTCTCACACGGAAGAG
ATGAGGCCTGTCATCAATCCATCAGATTACTCTTTTGATAGTGCAAATTGGAATTACAGAAAGGTTTCTT
TTTGGAGAAGG

RNA
>LINC03111
GCUAGCACUUGUUUGCUGAACUGUGGAAGAACUGCAAGGUCACGCACAUUAUUCAUGAUAAAGCAACUGA
GCUGACAAGUCUGUGCUUAAUUCAAGAAUAACCAGUAGGAGAGAGAGAAAACACAAGGAGUUAACCACCA
UCGCCGGUGCUCUCUCAAAGGAAGAGGGGUUAACAGAUGAAUUCGCAAAGACAGCUCUUCUGGCCAAGAA
UAUGGAUCAUUUGCAGCACAGAGGCAAGUUGAUAUAUGUAGCAUCAUGACAGUGAAGCAAAUUCCACAAA
AACUGCUAAUGGACGGAGAAAUUUCAUGGAGAAAAGCUAGCAGACAGAGCUGGAAUAAAACAAUGGCAGU
GUUCCUUGCUCAAAGAAGCAGUCCCGGGCUGUUCUUUGCCCAGGGAUAUCUUCCCAAGAUGACCAGAAAU
GGCUUCAUGACCUAAGGGUGGACAGCAGUGUUCUAUUGUACCCACAUCAUCAUCGUCUCACACGGAAGAG
AUGAGGCCUGUCAUCAAUCCAUCAGAUUACUCUUUUGAUAGUGCAAAUUGGAAUUACAGAAAGGUUUCUU
UUUGGAGAAGG
