# Supplementary material for: Long Intergenic Non-Coding RNAs of Human Chromosome 18: Focus on Cancers
Source: Biomedicines. 2024 Feb 28;12(3):544. doi: 10.3390/biomedicines12030544 (PMC10968066; doi:10.3390/biomedicines12030544)

Table S22. Predicted secondary structures of lincRNAs of human chromosome 18

Base-pair probabilities

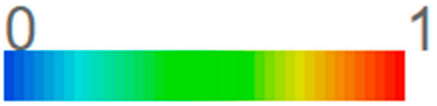

Prediction of secondary structures of full-length lincRNAs were performed using the 'RNAfold Server' web-based tool (http://rna.tbi.univie.ac.at//cgi-bin/RNAWebSuite/RNAfold.cgi) on the site of the Institute for Theoretical Chemistry University of Vienna.

Reference.

Reuter, J.S., Mathews, D.H. RNAstructure: software for RNA secondary structure prediction and analysis. BMC Bioinformatics 2010, 11, 129, doi: 10.1186/1471-2105-11-129.

LINC00305

Results for minimum free energy prediction

The minimum free energy of -223.50 kcal/mol

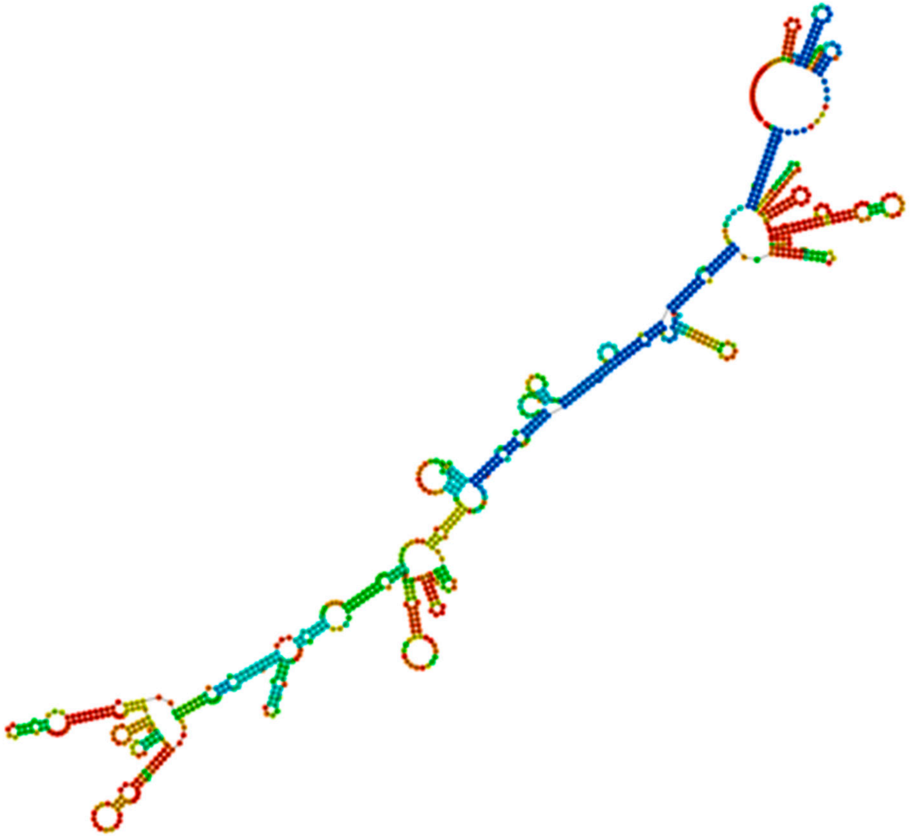

Results for thermodynamic ensemble prediction

The free energy of the thermodynamic ensemble is -241.63 kcal/mol.

The ensemble diversity is 220.42.

The minimum free energy of -157.32 kcal/mol

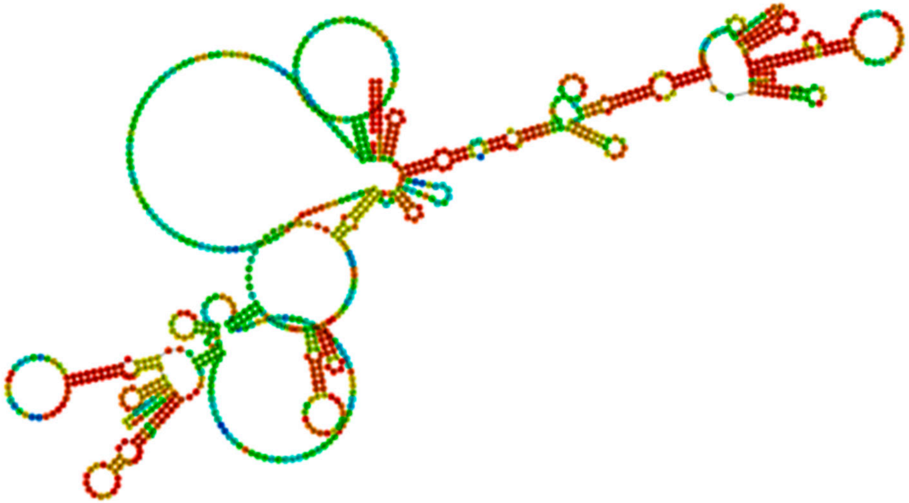

LINC00470

Results for minimum free energy prediction

The minimum free energy of **-565.90** kcal/mol

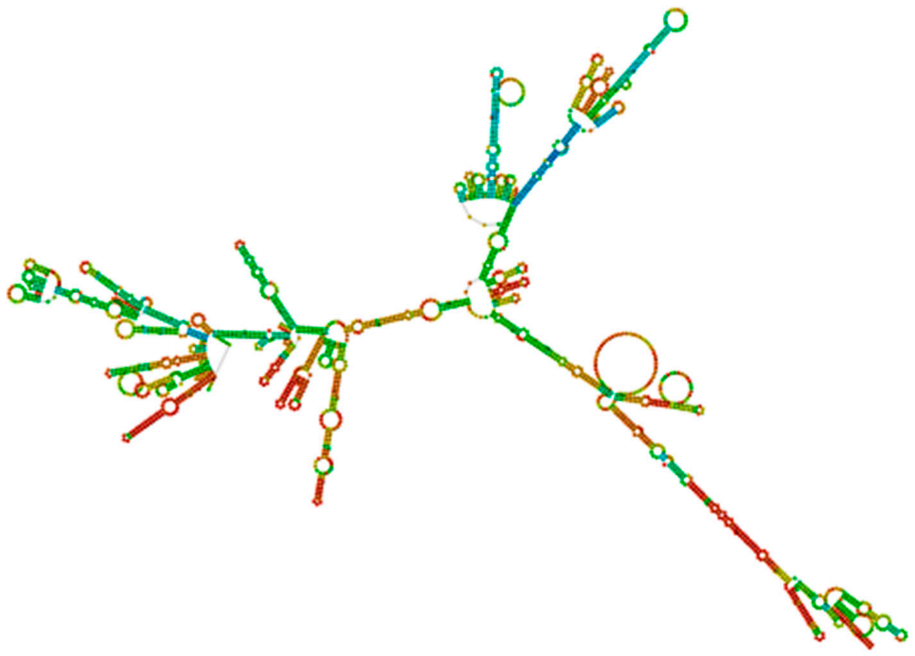

Results for thermodynamic ensemble prediction

The free energy of the thermodynamic ensemble is **-565.90** kcal/mol.

The ensemble diversity is **635.89**.

The minimum free energy of **-369.54** kcal/mol

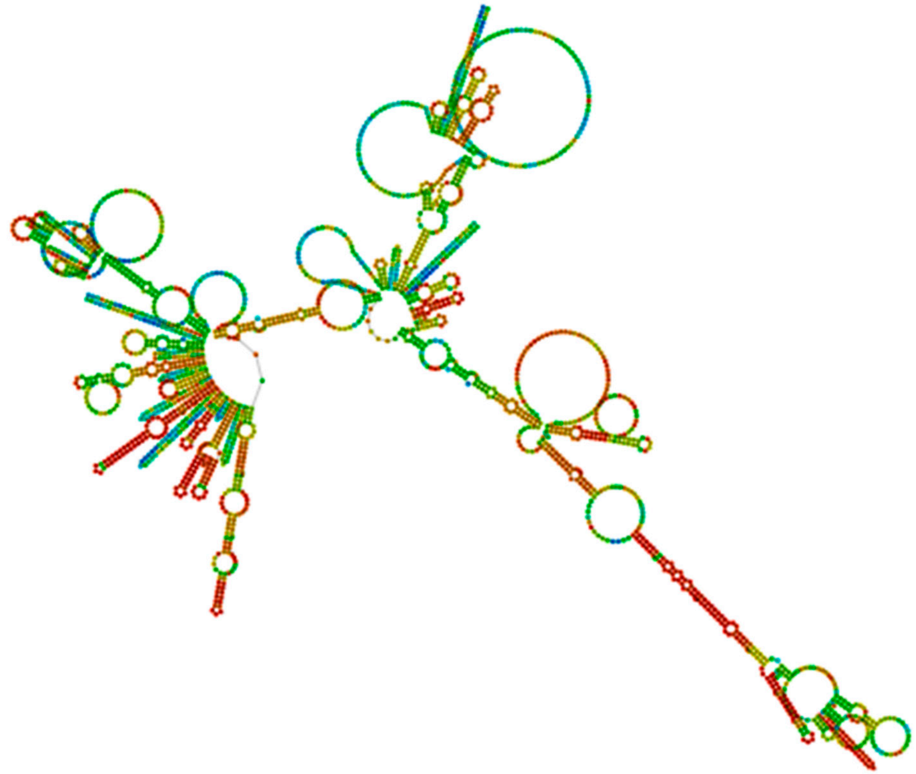

LINC00526

Results for minimum free energy prediction

The minimum free energy of **-421.10** kcal/mol

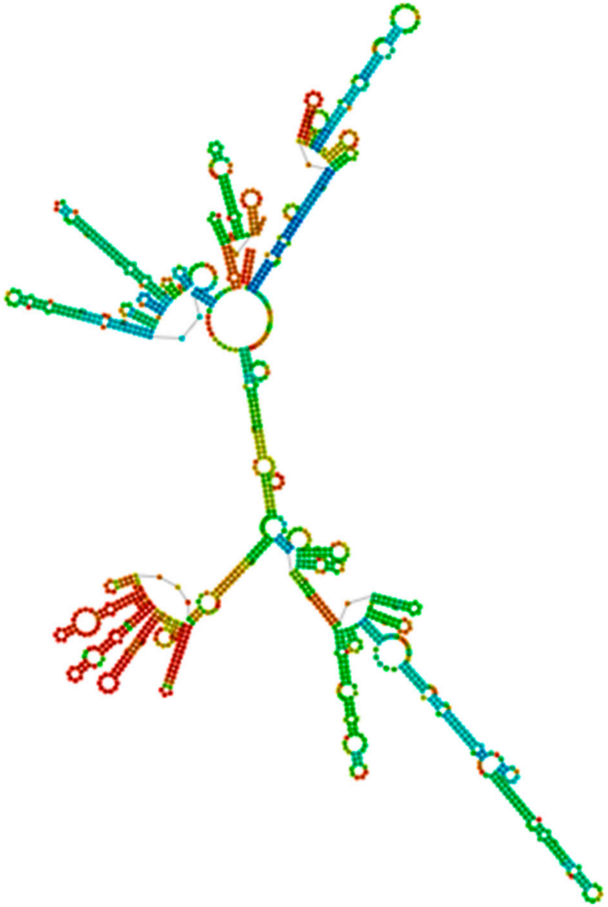

Results for thermodynamic ensemble prediction

The free energy of the thermodynamic ensemble is **-443.63** kcal/mol.

The ensemble diversity is **429.41**.

The minimum free energy of **-244.77** kcal/mol

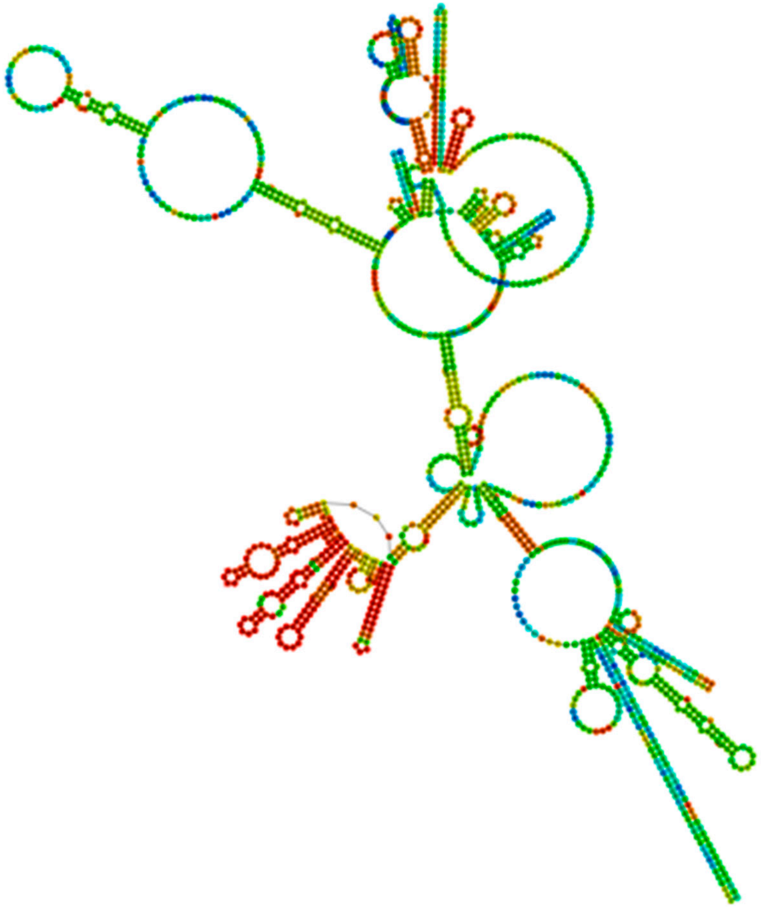

LINC00667

Results for minimum free energy prediction

The minimum free energy of **-1233.40** kcal/mol

Results for thermodynamic ensemble prediction

The free energy of the thermodynamic ensemble is **-1302.93** kcal/mol.

The ensemble diversity is **1082.07**.

The minimum free energy of **-880.24** kcal/mol

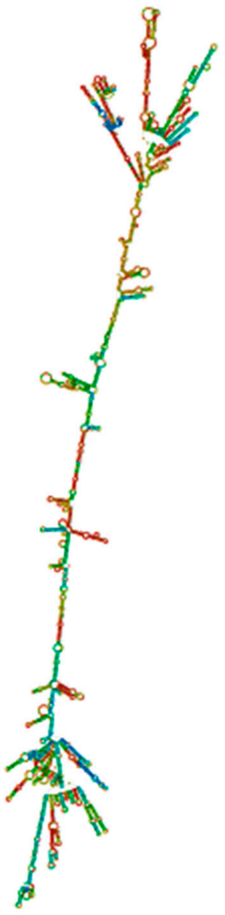

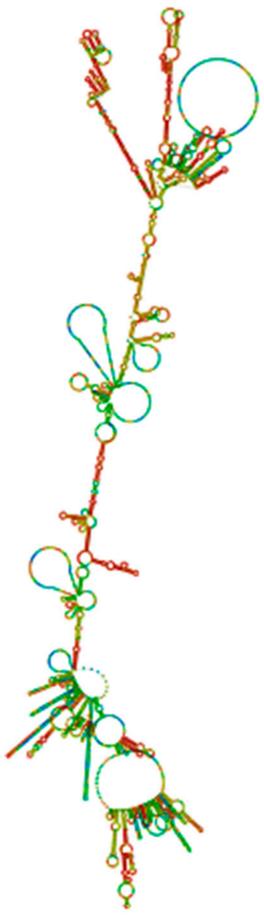

LINC00668

Results for minimum free energy prediction

The minimum free energy of **-575.90** kcal/mol

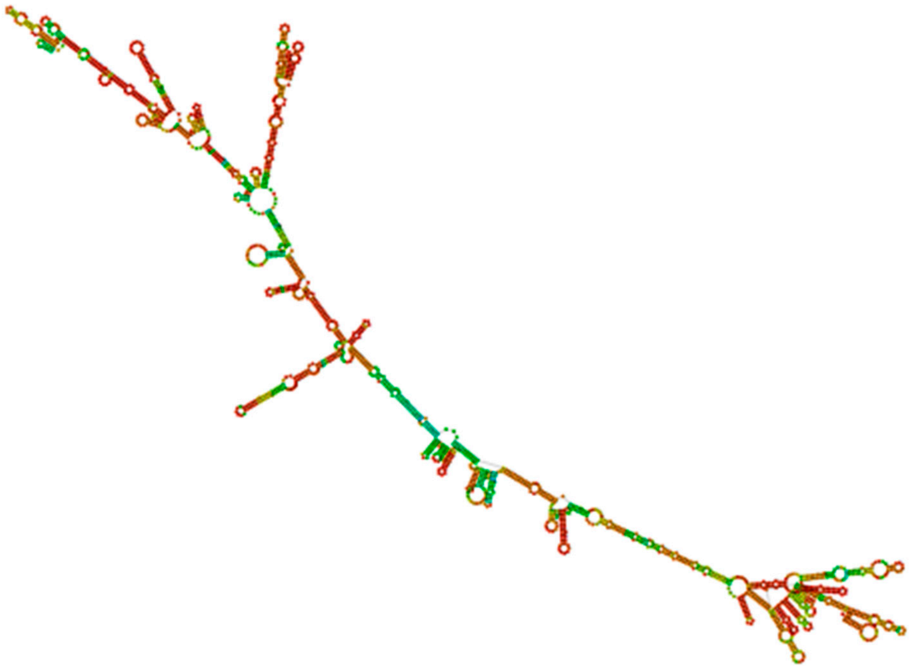

Results for thermodynamic ensemble prediction

The free energy of the thermodynamic ensemble is **-604.52** kcal/mol.

The ensemble diversity is **331.91**.

The minimum free energy of **-492.86** kcal/mol

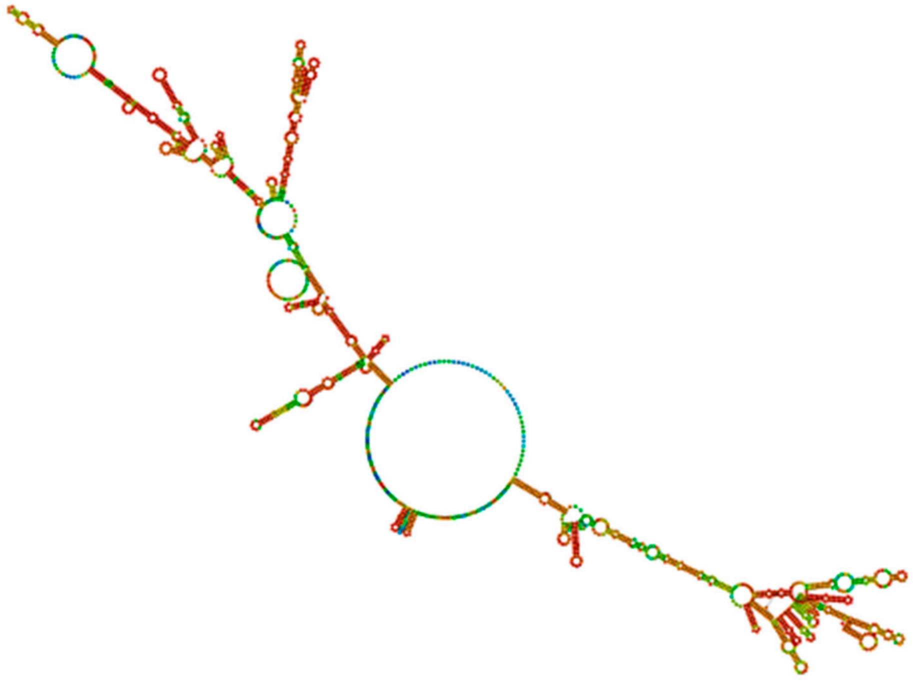

LINC00907

Results for minimum free energy prediction

The minimum free energy of **-578.50** kcal/mol

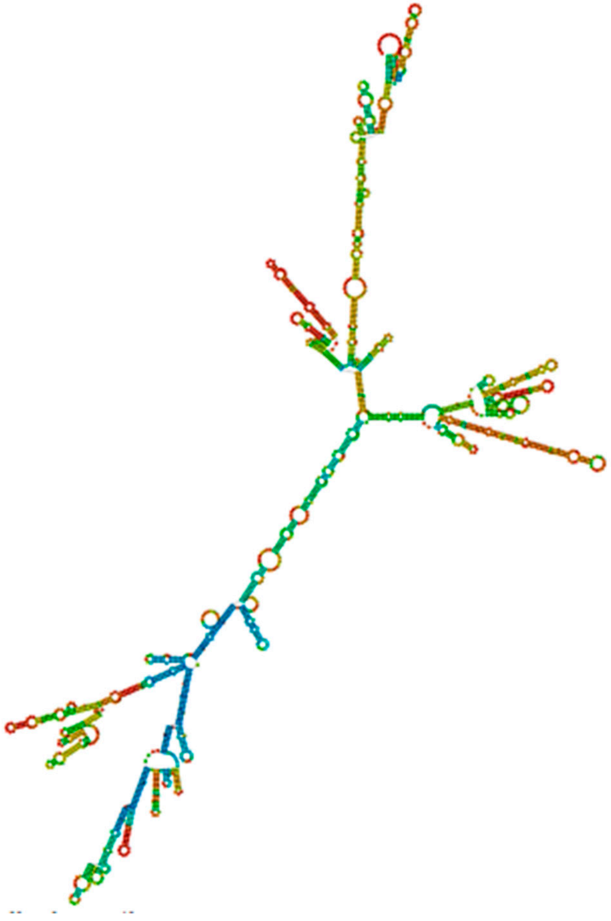

Results for thermodynamic ensemble prediction

The free energy of the thermodynamic ensemble is **-612.32** kcal/mol.

The ensemble diversity is **504.83**.

The minimum free energy of **-425.90** kcal/mol

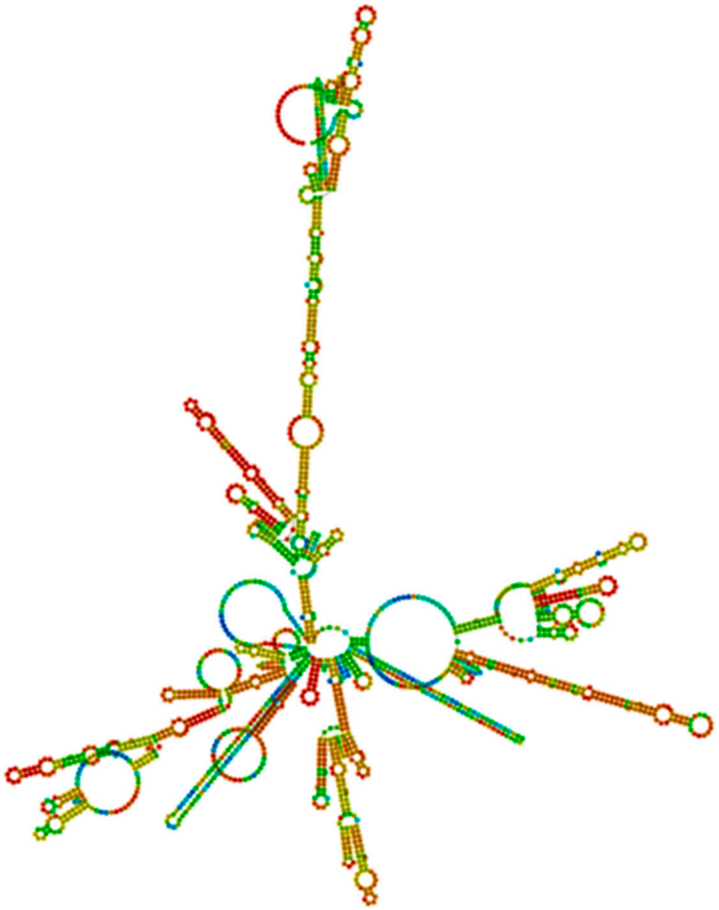

LINC01254

Results for minimum free energy prediction

The minimum free energy of **-629.40** kcal/mol

Results for thermodynamic ensemble prediction

The free energy of the thermodynamic ensemble is **-663.82** kcal/mol.

The ensemble diversity is **400.74**.

The minimum free energy of **-543.20** kcal/mol

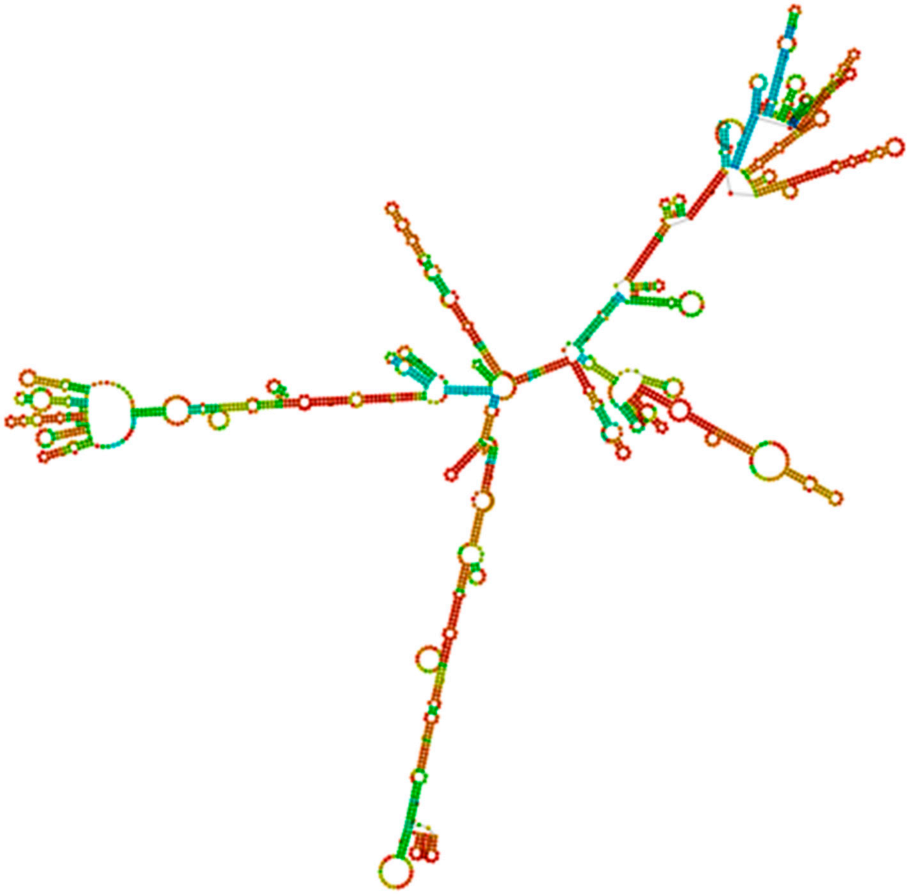

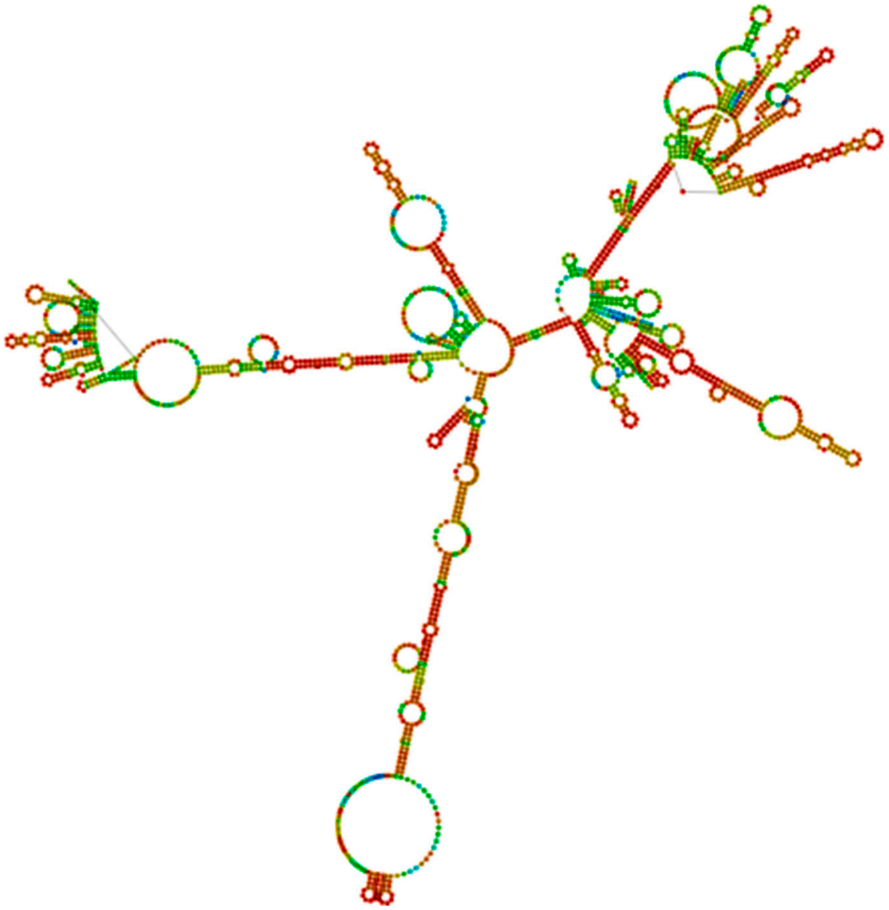

LINC01255

Results for minimum free energy prediction

The minimum free energy of **-134.20** kcal/mol

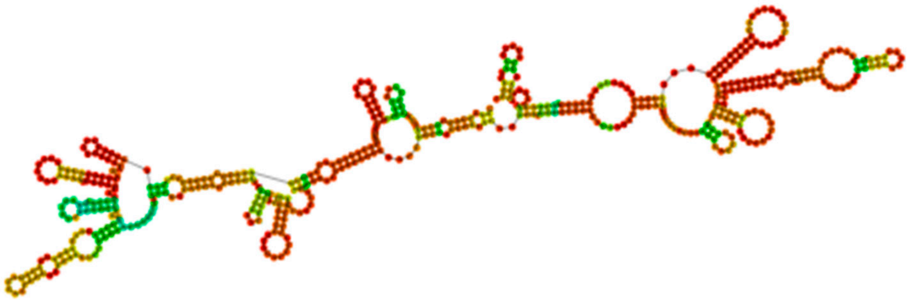

Results for thermodynamic ensemble prediction

The free energy of the thermodynamic ensemble is **-141.51** kcal/mol.

The ensemble diversity is **78.24**.

The minimum free energy of **-125.20** kcal/mol

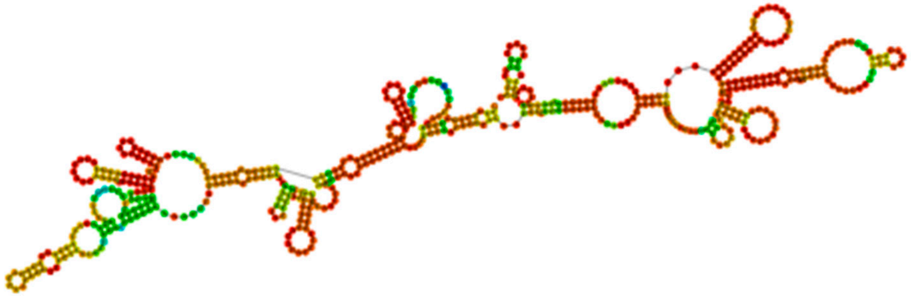

LINC01387

Results for minimum free energy prediction

The minimum free energy of **-201.70** kcal/mol

Results for thermodynamic ensemble prediction

The free energy of the thermodynamic ensemble is **-212.37** kcal/mol.

The ensemble diversity is **239.37**.

The minimum free energy of **-109.50** kcal/mol

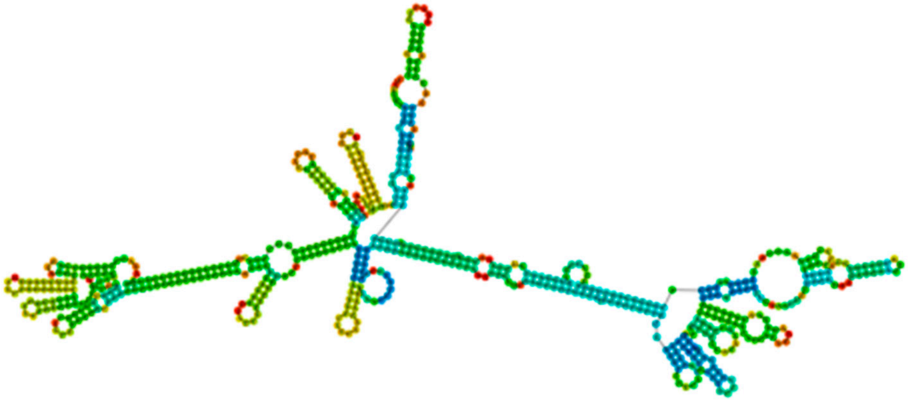

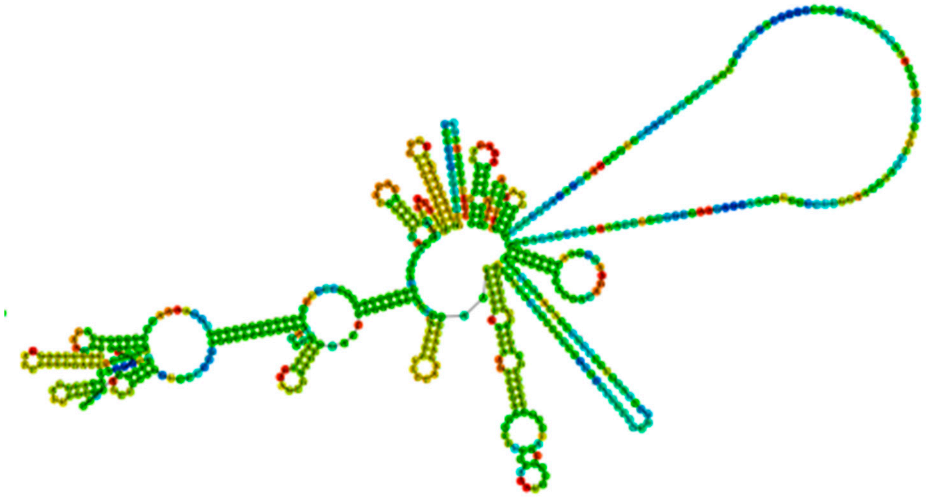

LINC01415

Results for minimum free energy prediction

The minimum free energy of **-1608.30** kcal/mol

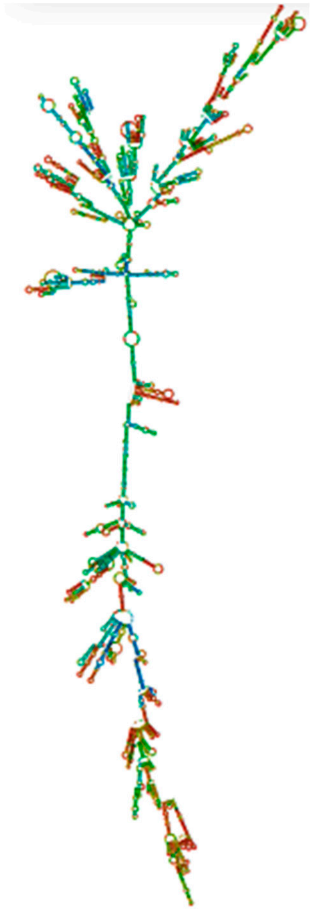

Results for thermodynamic ensemble prediction

The free energy of the thermodynamic ensemble is **-1723.47** kcal/mol.

The ensemble diversity is **1756.46**.

The minimum free energy of **-1045.79** kcal/mol

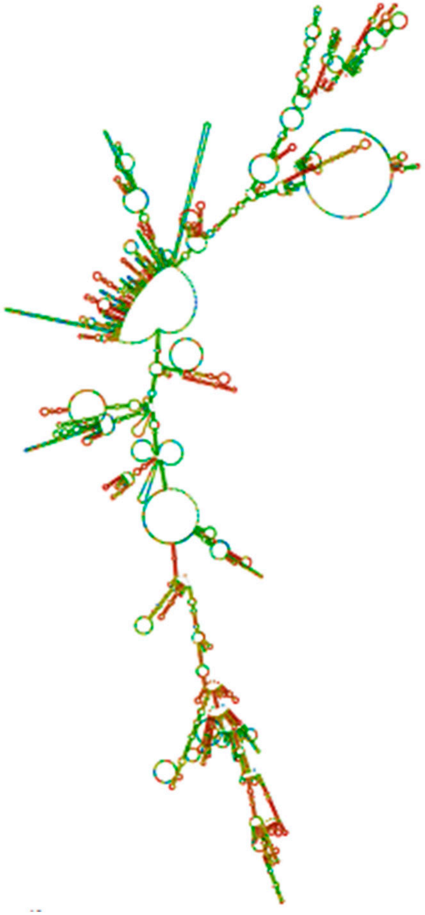

LINC01416

Results for minimum free energy prediction

The minimum free energy of **-566.50** kcal/mol

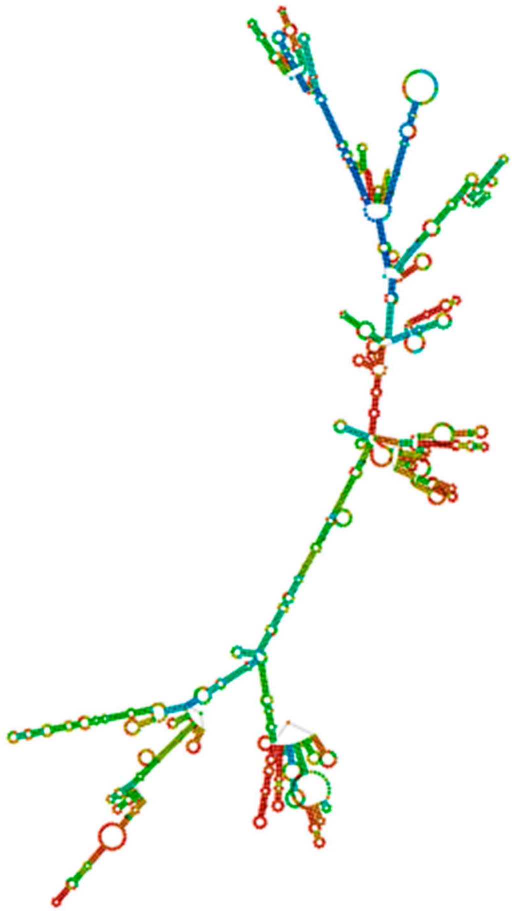

Results for thermodynamic ensemble prediction

The free energy of the thermodynamic ensemble is **-608.26** kcal/mol.

The ensemble diversity is **646.93**.

The minimum free energy of **-379.61** kcal/mol

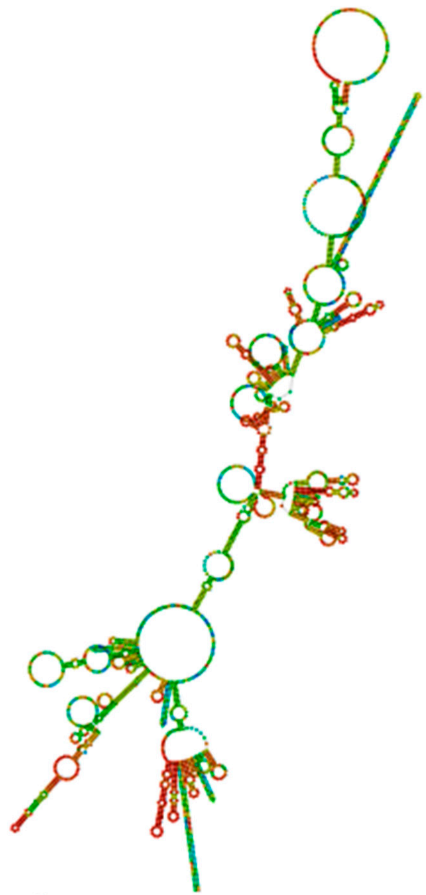

LINC01443

Results for minimum free energy prediction

The minimum free energy of **-600.60** kcal/mol

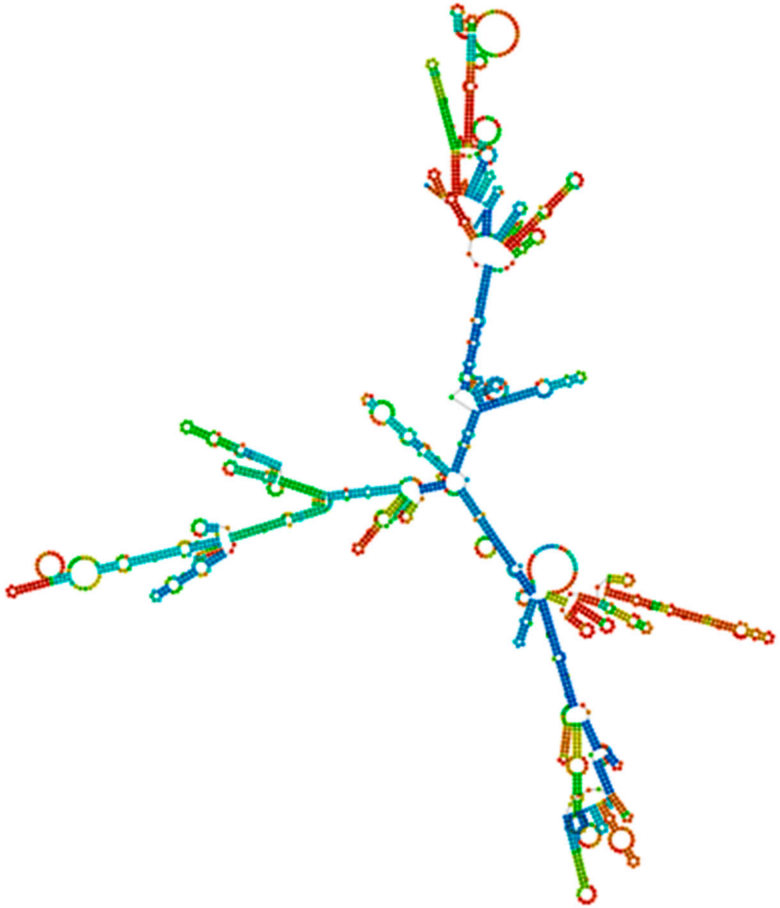

Results for thermodynamic ensemble prediction

The free energy of the thermodynamic ensemble is **-628.43** kcal/mol.

The ensemble diversity is **510.01**.

The minimum free energy of **-392.57** kcal/mol

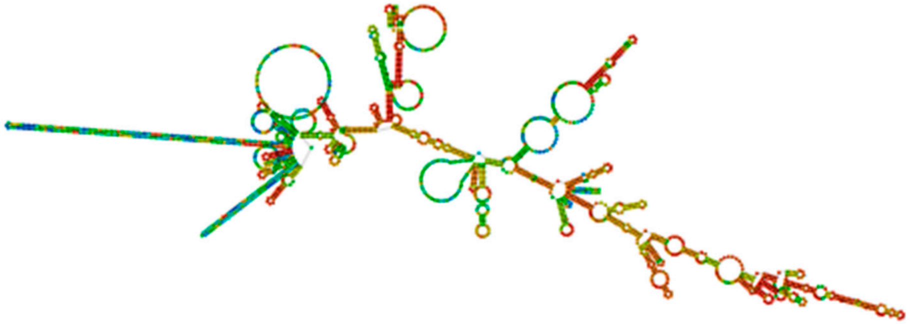

LINC01444

Results for minimum free energy prediction

The minimum free energy of **-244.70** kcal/mol

Results for thermodynamic ensemble prediction

The free energy of the thermodynamic ensemble is **-258.07** kcal/mol.

The ensemble diversity is **260.24**.

The minimum free energy of **-166.70** kcal/mol

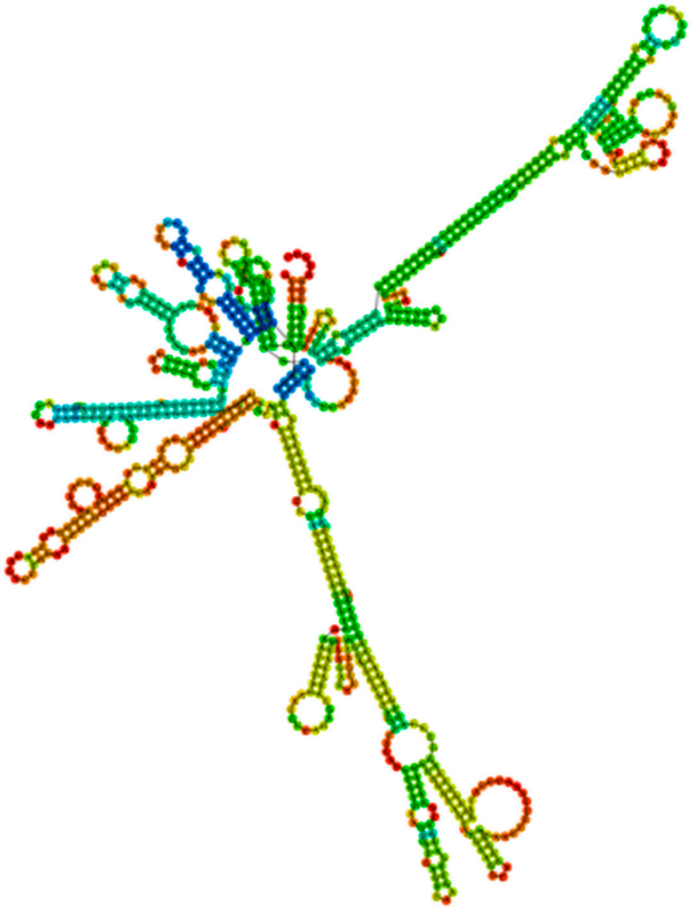

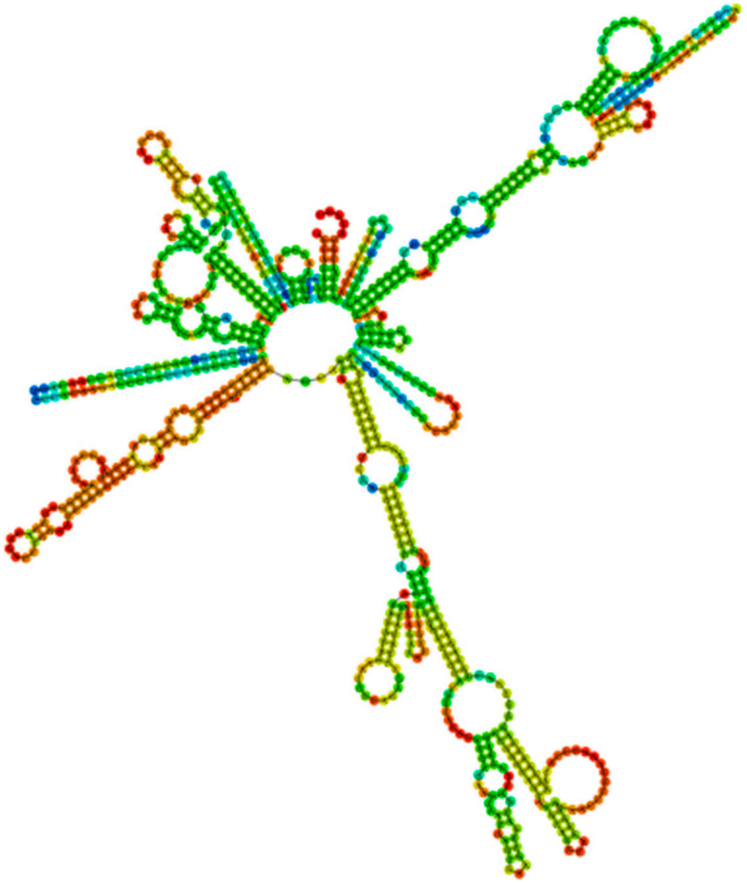

LINC01477

Results for minimum free energy prediction

The minimum free energy of **-292.30** kcal/mol

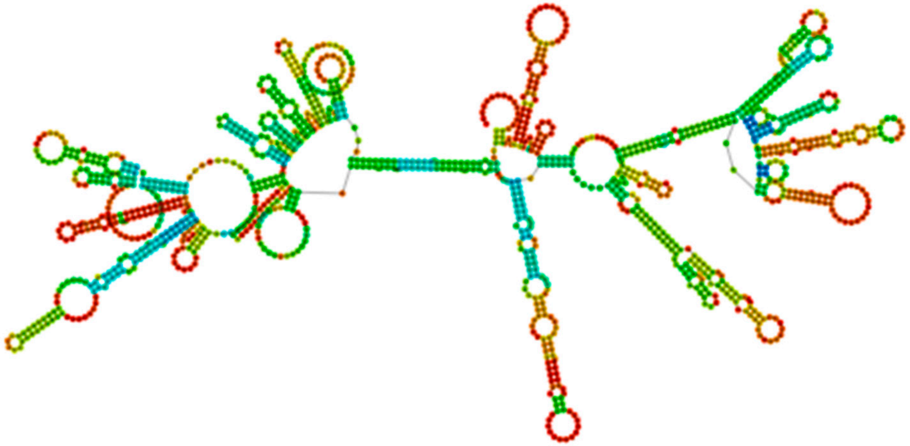

Results for thermodynamic ensemble prediction

The free energy of the thermodynamic ensemble is **-314.03** kcal/mol.

The ensemble diversity is **364.83**.

The minimum free energy of **-196.20** kcal/mol

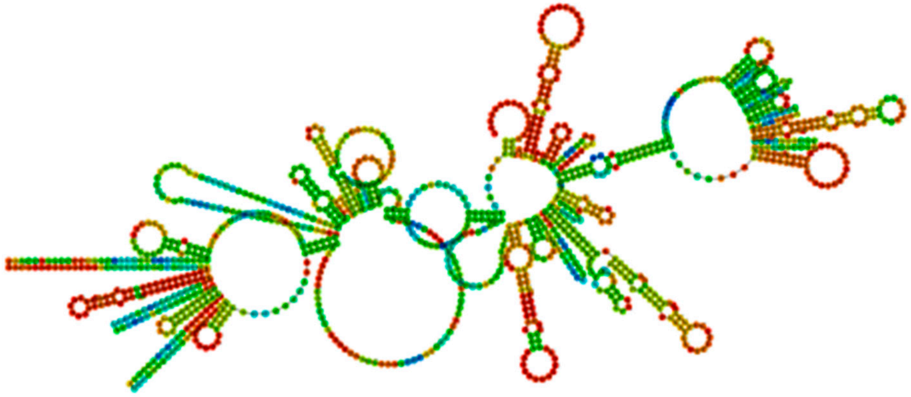

LINC01478

Results for minimum free energy prediction

The minimum free energy of **-485.30** kcal/mol

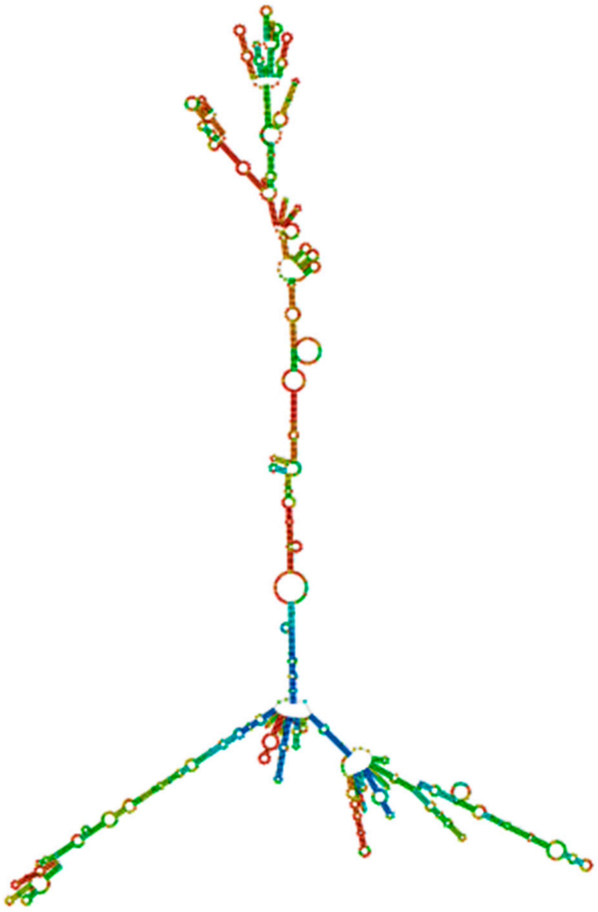

Results for thermodynamic ensemble prediction

The free energy of the thermodynamic ensemble is **-522.67** kcal/mol.

The ensemble diversity is **556.56**.

The minimum free energy of **-330.90** kcal/mol

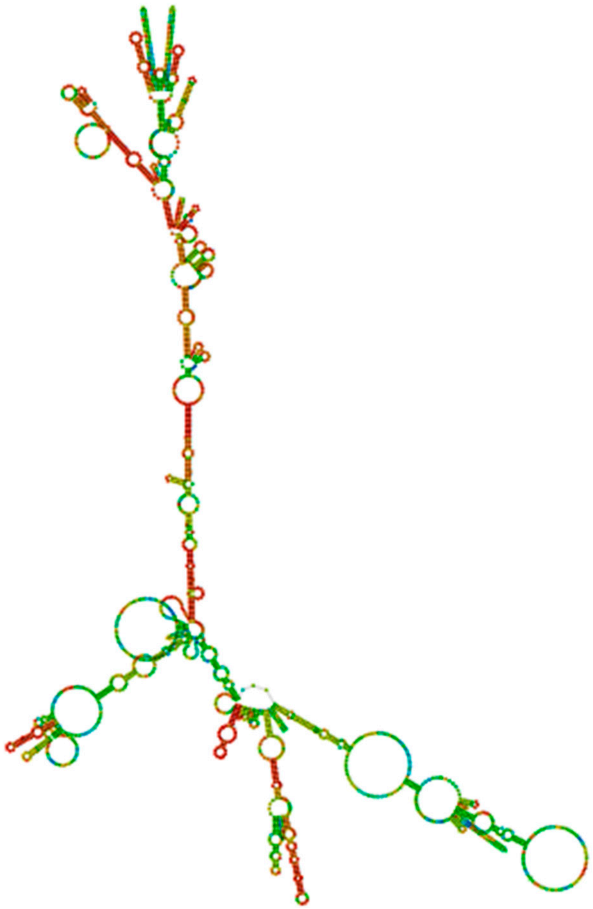

LINC01538

Results for minimum free energy prediction

The minimum free energy of **-540.64** kcal/mol

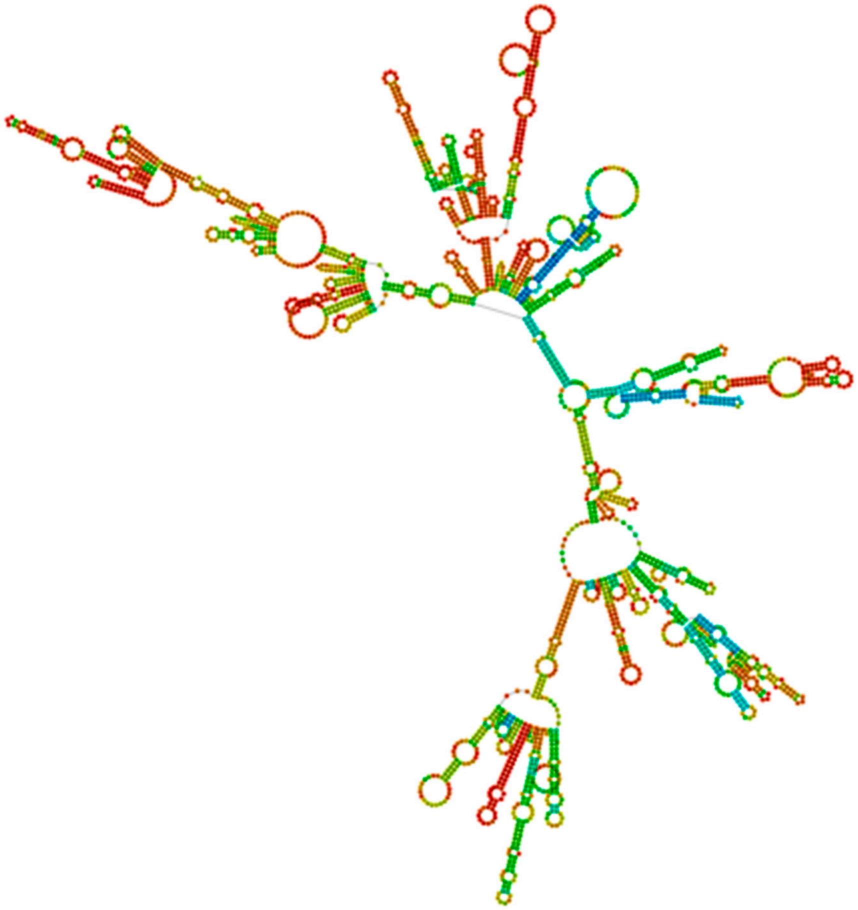

Results for thermodynamic ensemble prediction

The free energy of the thermodynamic ensemble is **-584.55** kcal/mol.

The ensemble diversity is **604.85**.

The minimum free energy of **-422.73** kcal/mol

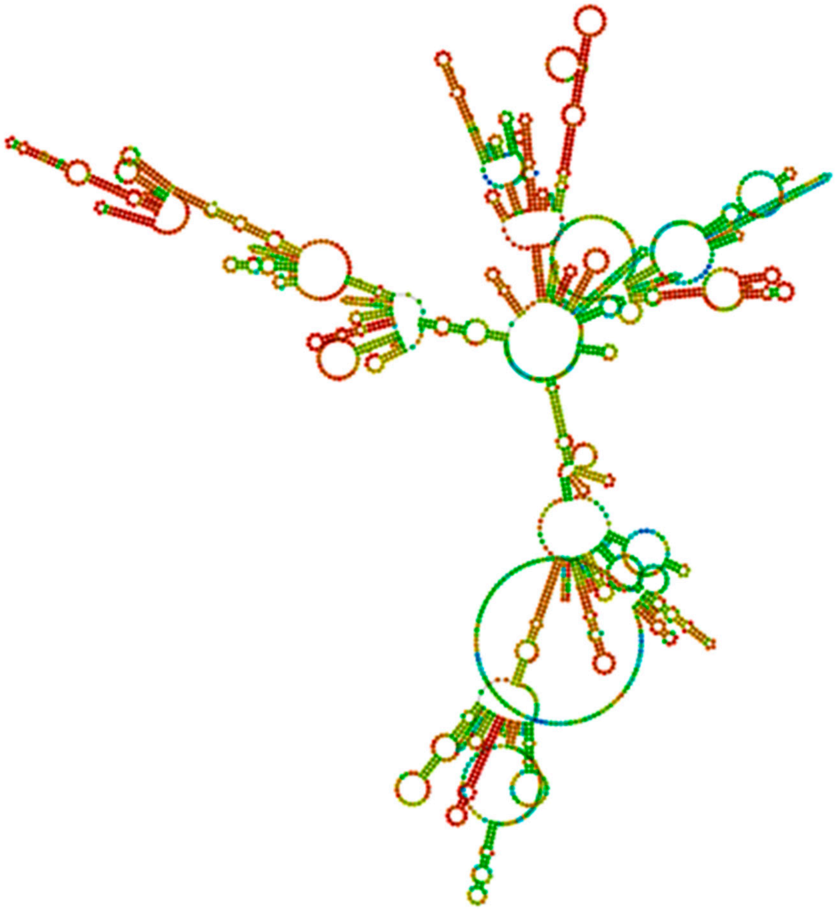

LINC01539

Results for minimum free energy prediction

The minimum free energy of **-544.10** kcal/mol

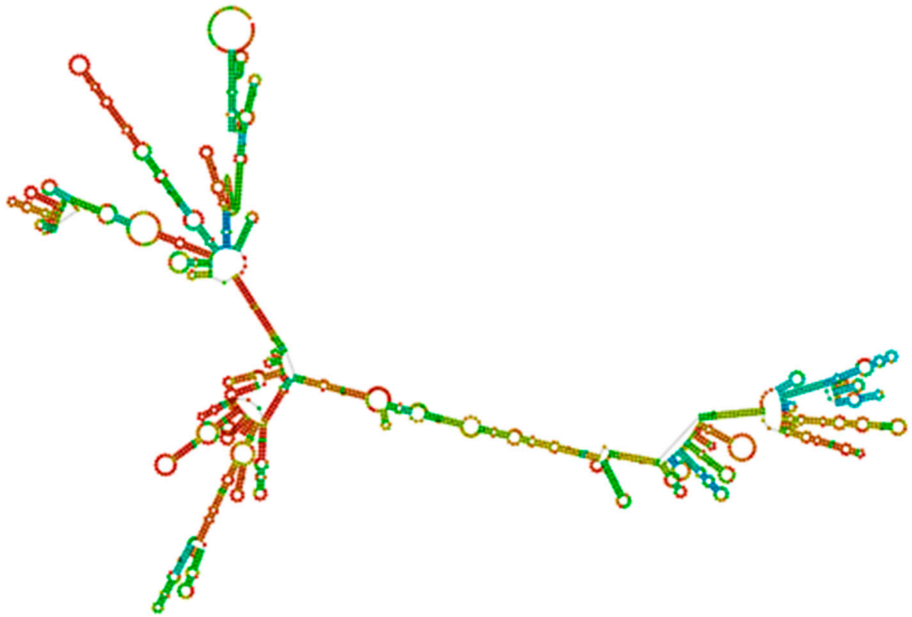

Results for thermodynamic ensemble prediction

The free energy of the thermodynamic ensemble is **-582.27** kcal/mol.

The ensemble diversity is **531.01**.

The minimum free energy of **-392.99** kcal/mol

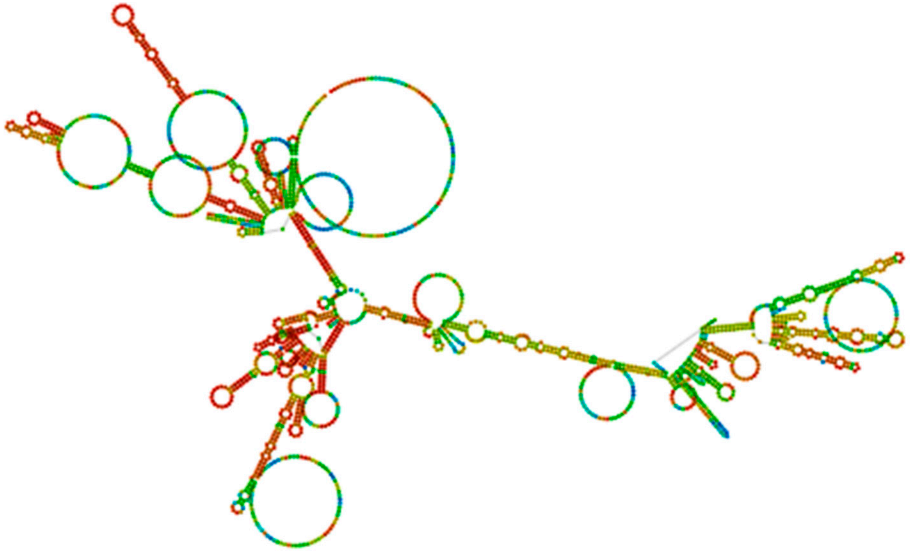

LINC01541

Results for minimum free energy prediction
The minimum free energy of **-736.30** kcal/mol

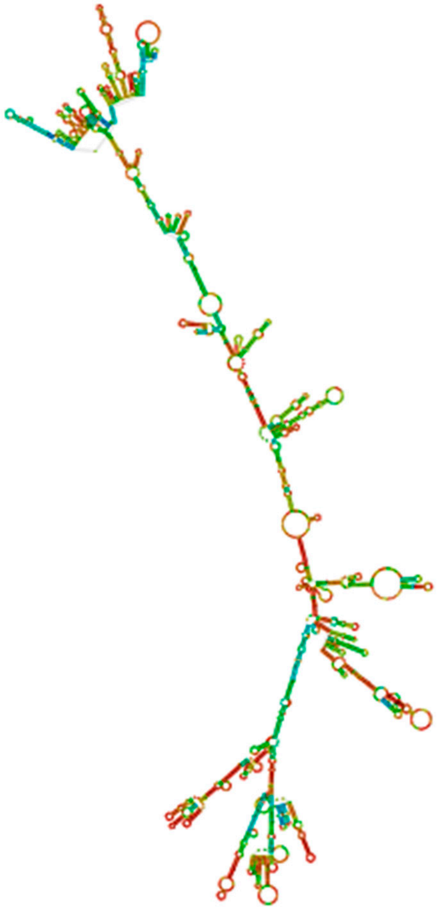

Results for thermodynamic ensemble prediction
The free energy of the thermodynamic ensemble is **-798.03** kcal/mol.

The ensemble diversity is **796.54**.
The minimum free energy of **-526.57** kcal/mol

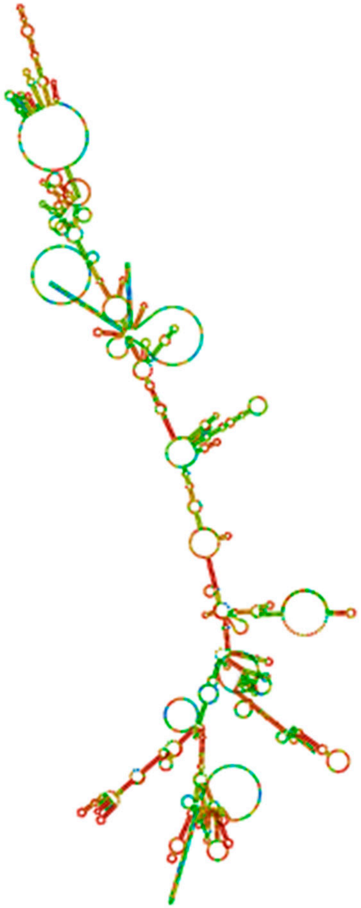

LINC01543

Results for minimum free energy prediction

The minimum free energy of **-213.60** kcal/mol

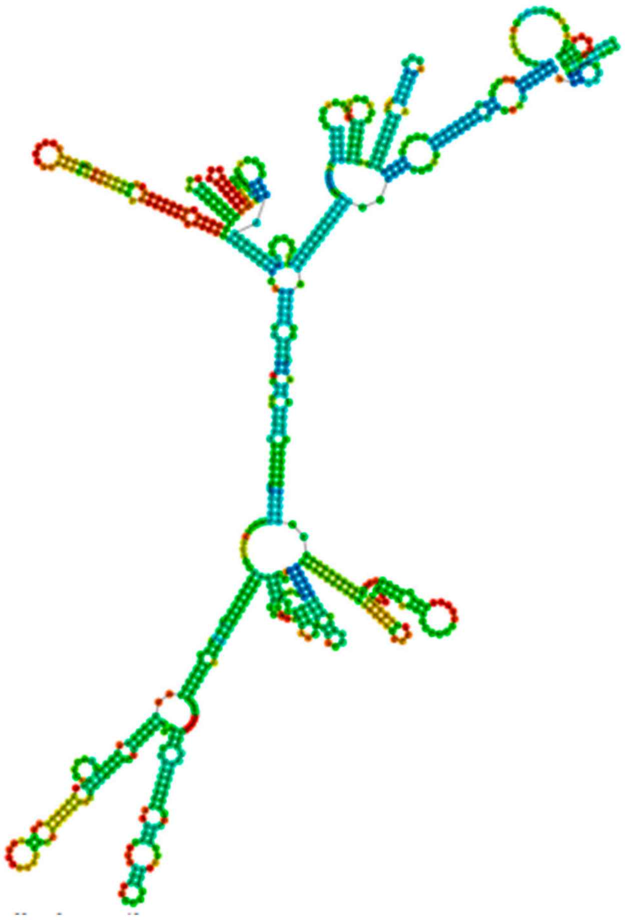

Results for thermodynamic ensemble prediction

The free energy of the thermodynamic ensemble is **-230.12** kcal/mol.

The ensemble diversity is **294.84**.

The minimum free energy of **-79.55** kcal/mol

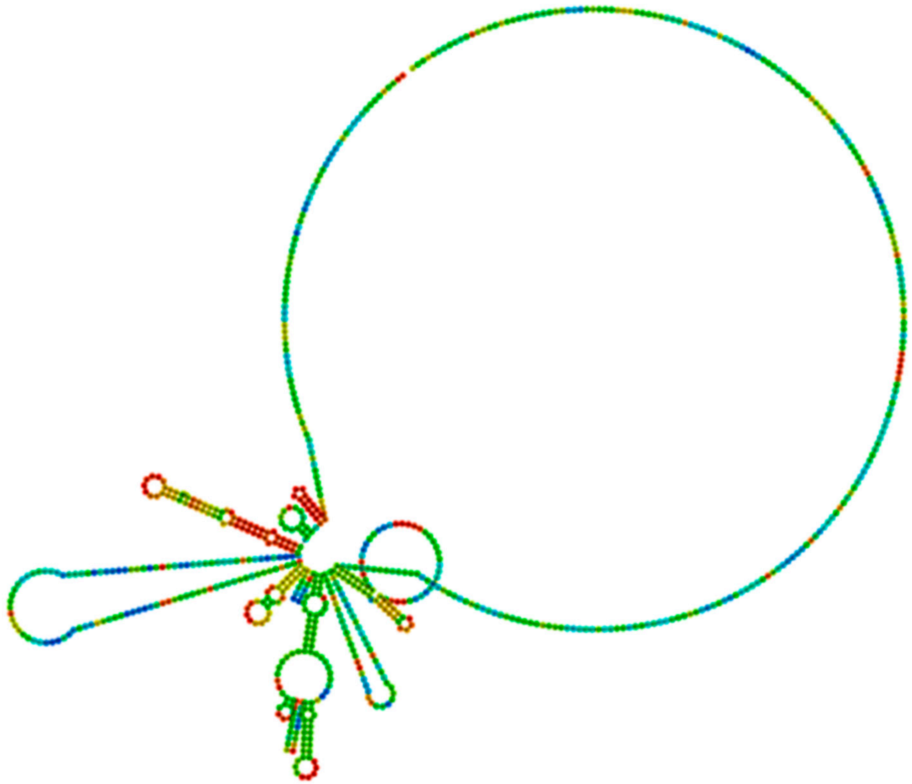

LINC01544

Results for minimum free energy prediction

The minimum free energy of **-1156.30** kcal/mol

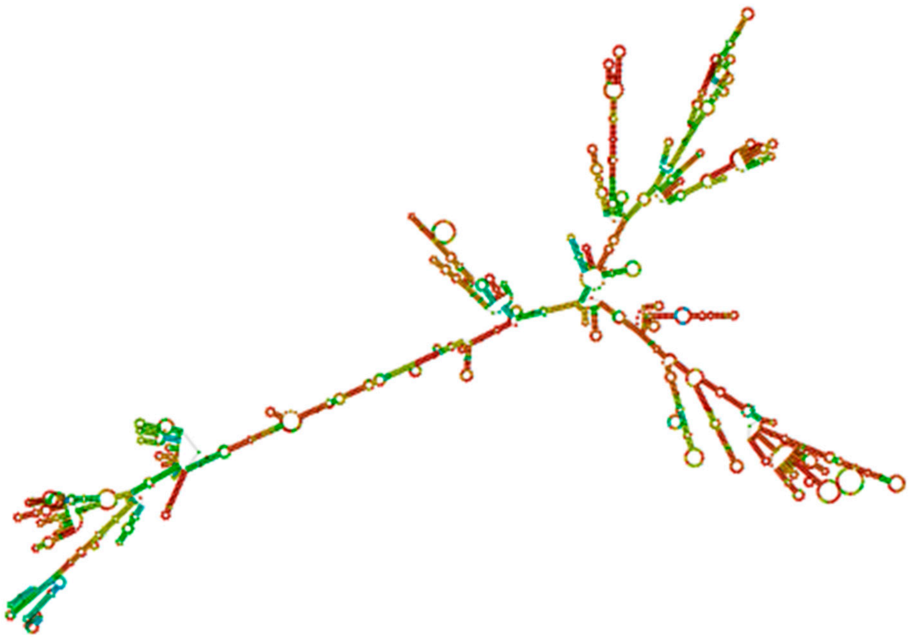

Results for thermodynamic ensemble prediction

The free energy of the thermodynamic ensemble is **-1204.93** kcal/mol.

The ensemble diversity is **684.32**.

The minimum free energy of **-995.00** kcal/mol

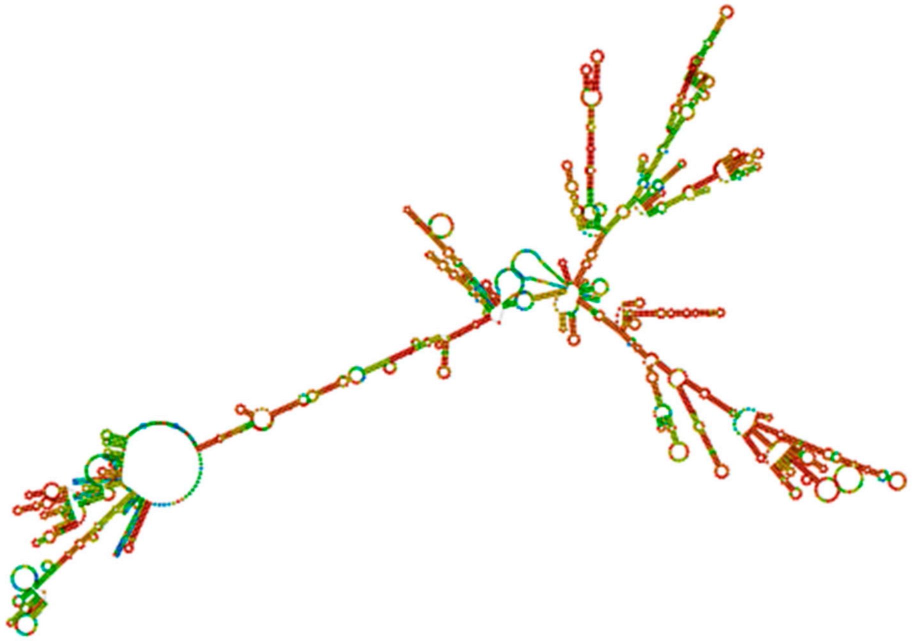

LINC01601

Results for minimum free energy prediction

The minimum free energy of **-228.40** kcal/mol

Results for thermodynamic ensemble prediction

The free energy of the thermodynamic ensemble is **-245.08** kcal/mol.

The ensemble diversity is **234.18**.

The minimum free energy of **-169.10** kcal/mol

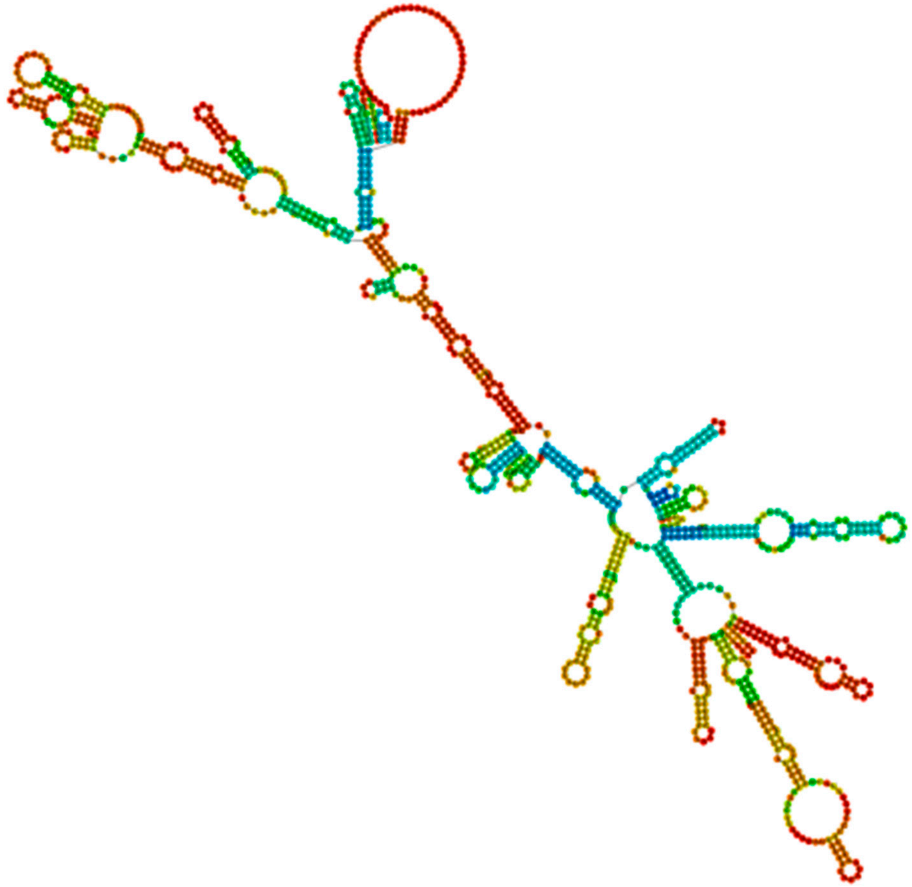

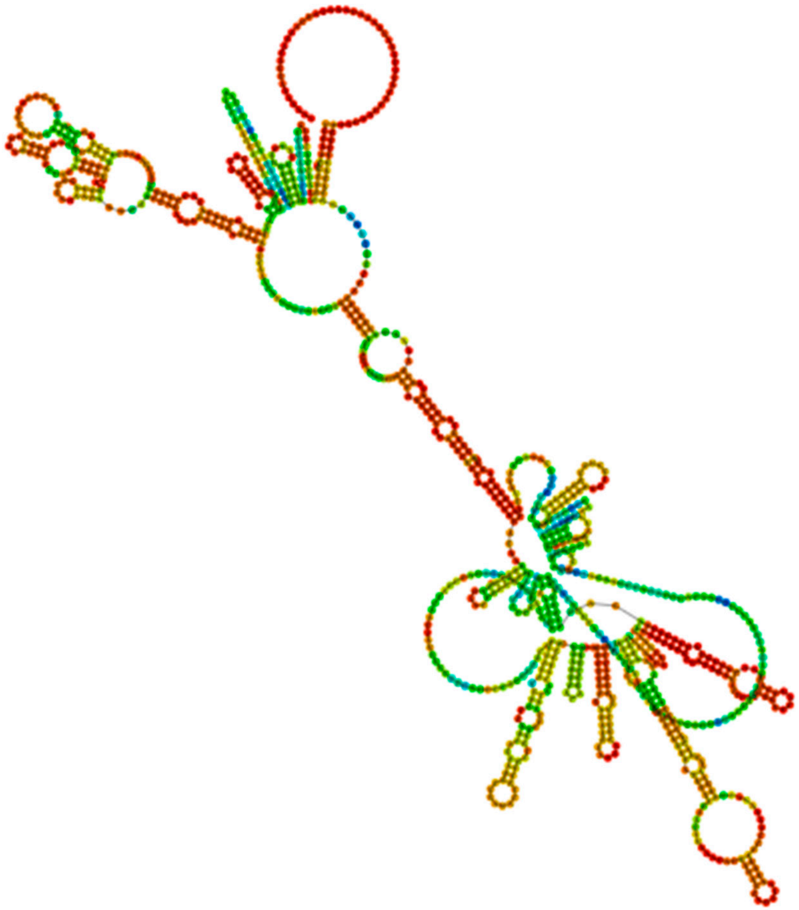

LINC01630

Results for minimum free energy prediction
The minimum free energy of **-539.20** kcal/mol

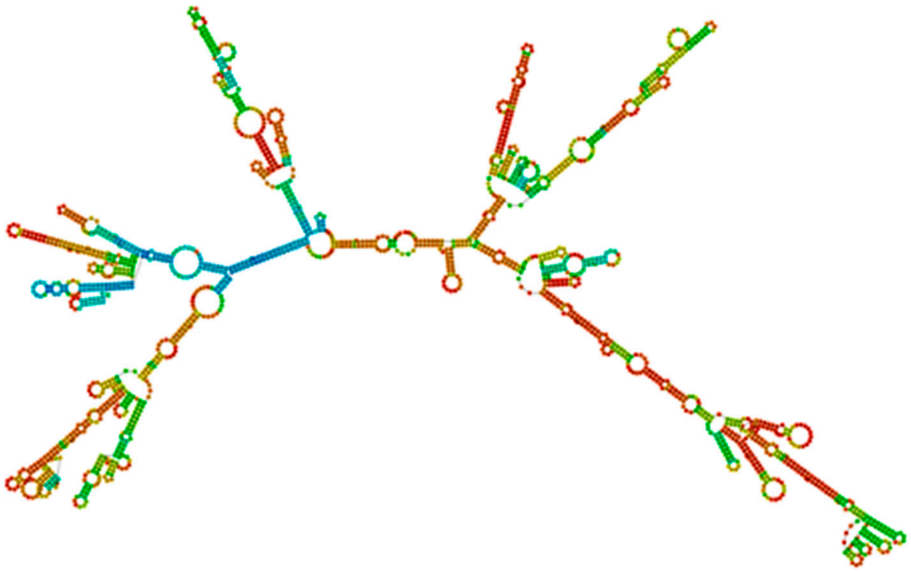

Results for thermodynamic ensemble prediction
The free energy of the thermodynamic ensemble is **-569.53** kcal/mol.

The ensemble diversity is **429.31**.
The minimum free energy of **-437.20** kcal/mol

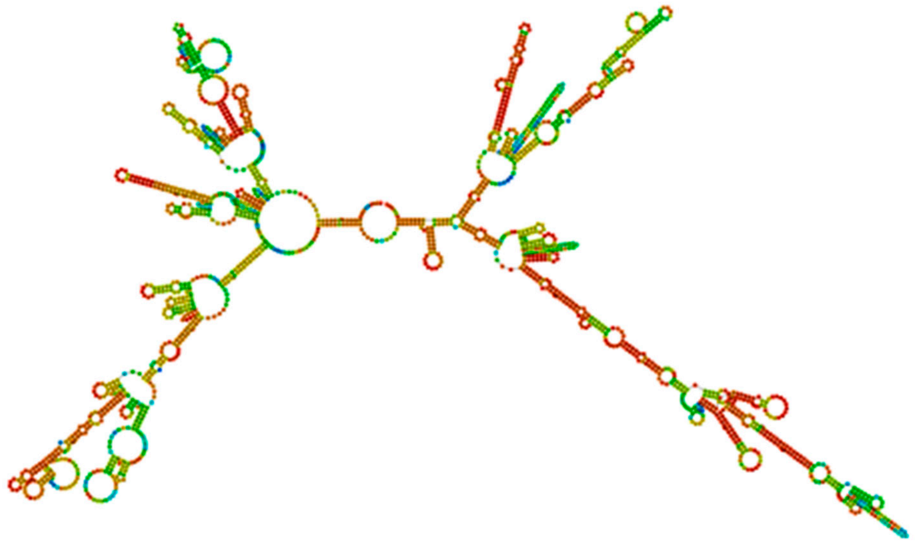

LINC01882

Results for minimum free energy prediction

The minimum free energy of **-167.00** kcal/mol

Results for thermodynamic ensemble prediction

The free energy of the thermodynamic ensemble is **-176.66** kcal/mol.

The ensemble diversity is **127.86**.

The minimum free energy of **-117.70** kcal/mol

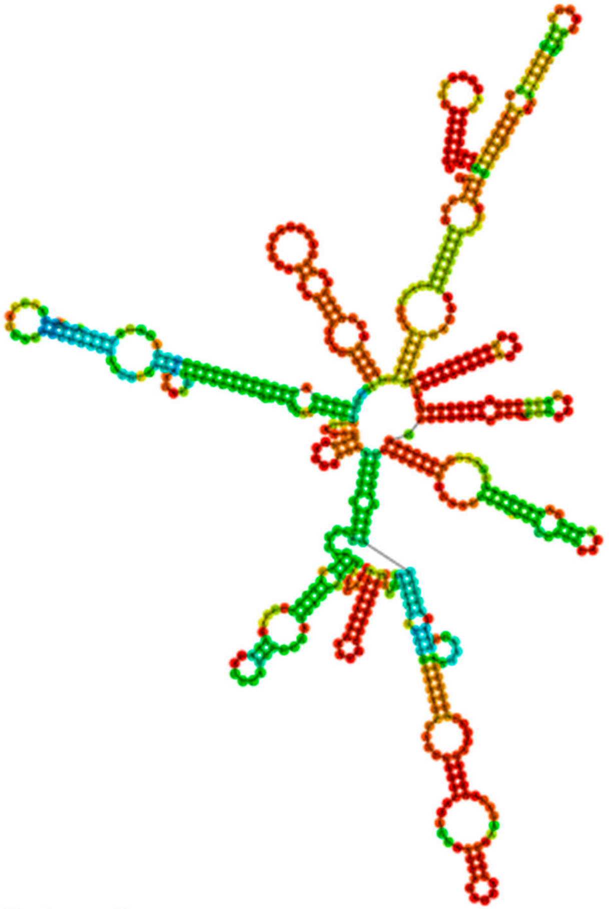

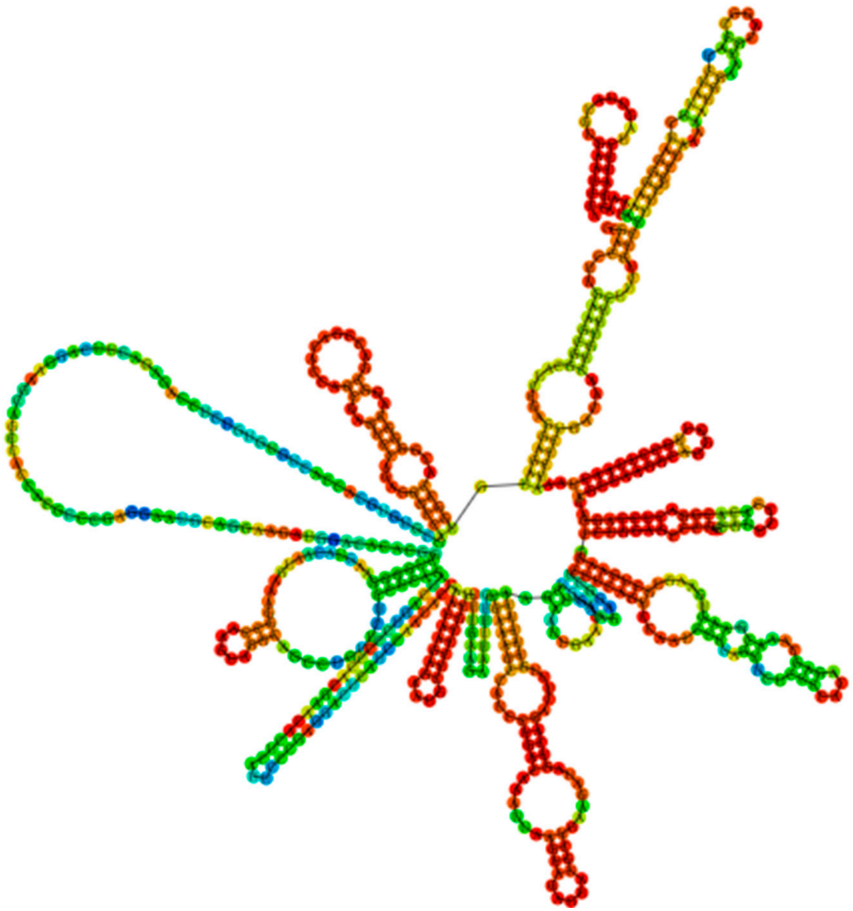

LINC01887

Results for minimum free energy prediction

The minimum free energy of **-595.80** kcal/mol

Results for thermodynamic ensemble prediction

The free energy of the thermodynamic ensemble is **-633.35** kcal/mol.

The ensemble diversity is **721.56**.

The minimum free energy of **-391.70** kcal/mol

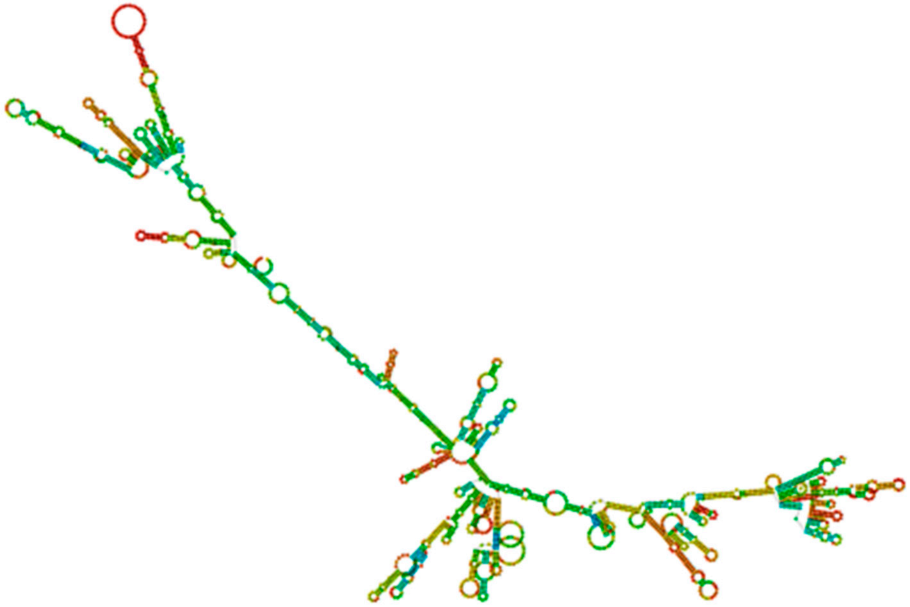

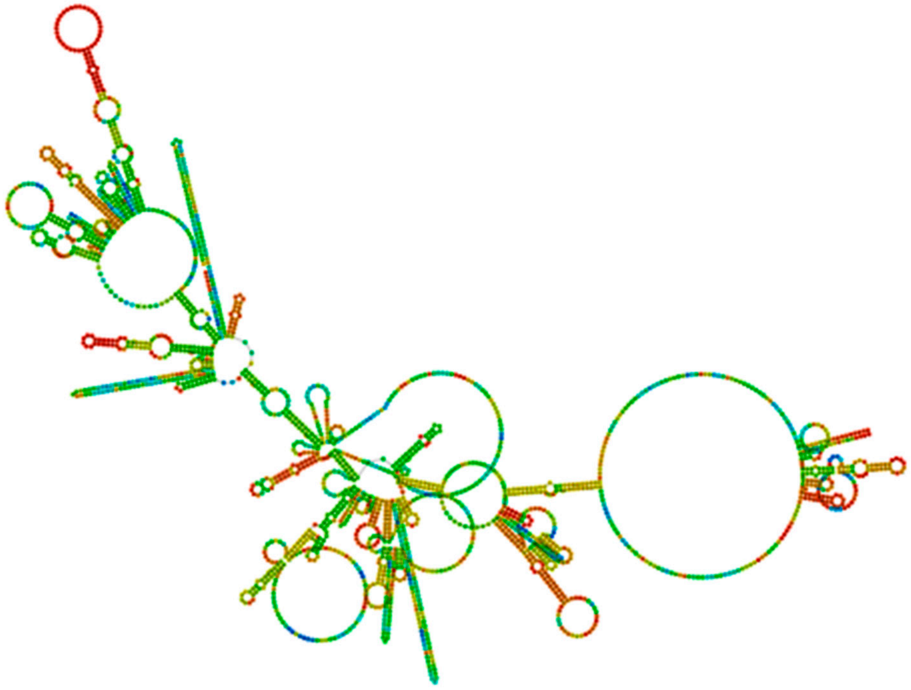

LINC01892

Results for minimum free energy prediction

The minimum free energy of **-296.30** kcal/mol

Results for thermodynamic ensemble prediction

The free energy of the thermodynamic ensemble is **-315.40** kcal/mol.

The ensemble diversity is **315.22**.

The minimum free energy of **-204.03** kcal/mol

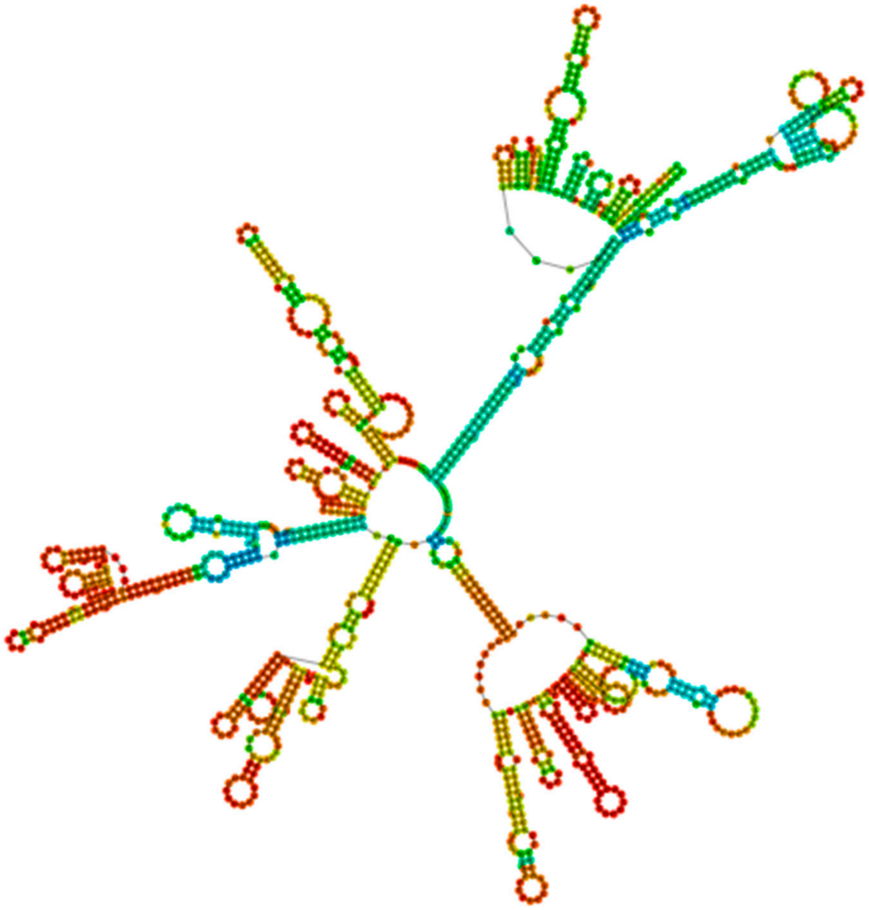

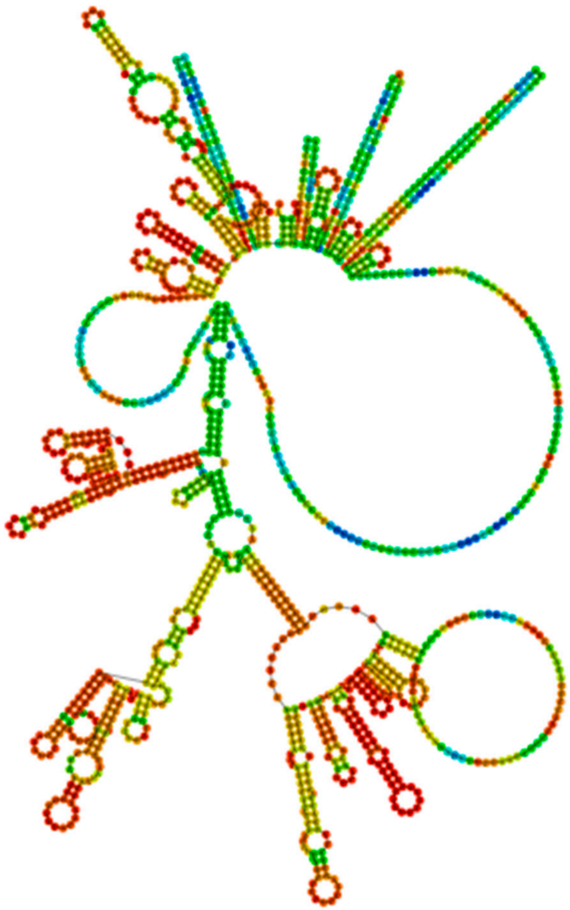

LINC01894

Results for minimum free energy prediction

The minimum free energy of **-439.20** kcal/mol

Results for thermodynamic ensemble prediction

The free energy of the thermodynamic ensemble is **-465.55** kcal/mol.

The ensemble diversity is **403.81**.

The minimum free energy of **-299.42** kcal/mol

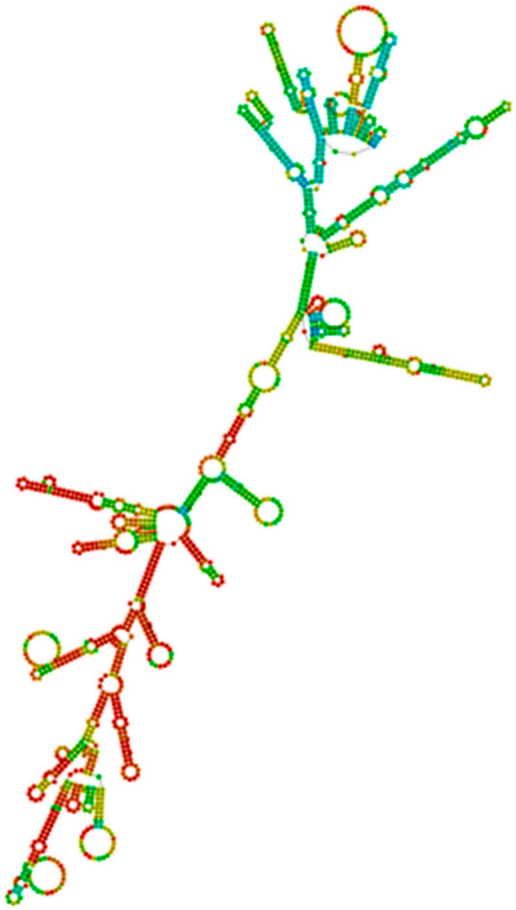

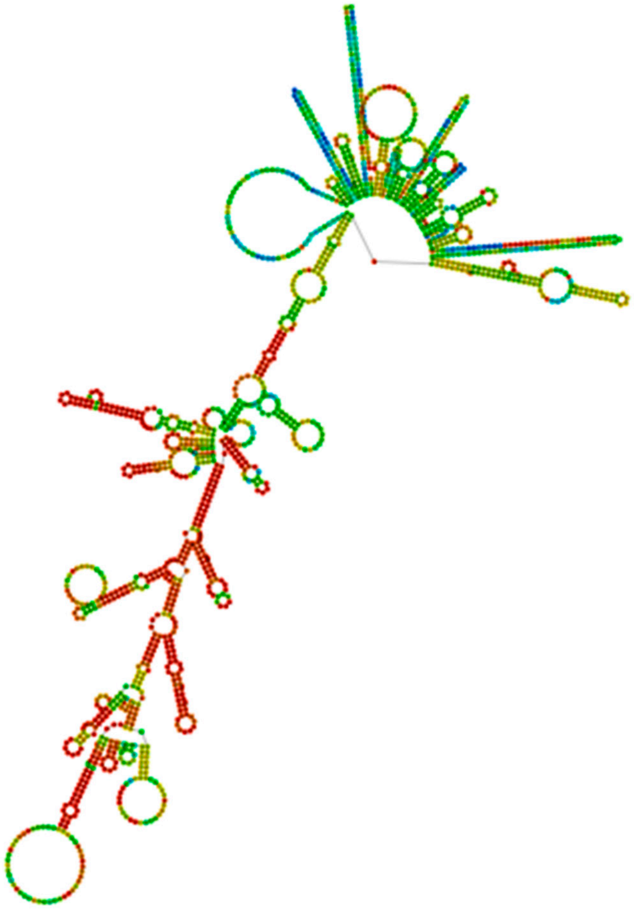

LINC01895

Results for minimum free energy prediction

The minimum free energy of **-141.00** kcal/mol

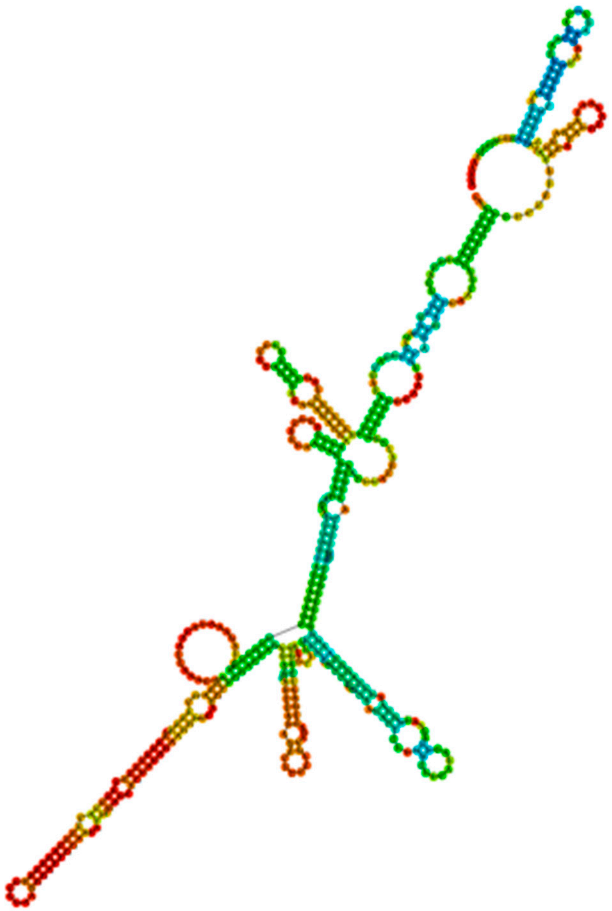

Results for thermodynamic ensemble prediction

The free energy of the thermodynamic ensemble is **-149.99** kcal/mol.

The ensemble diversity is **147.70**.

The minimum free energy of **-78.20** kcal/mol

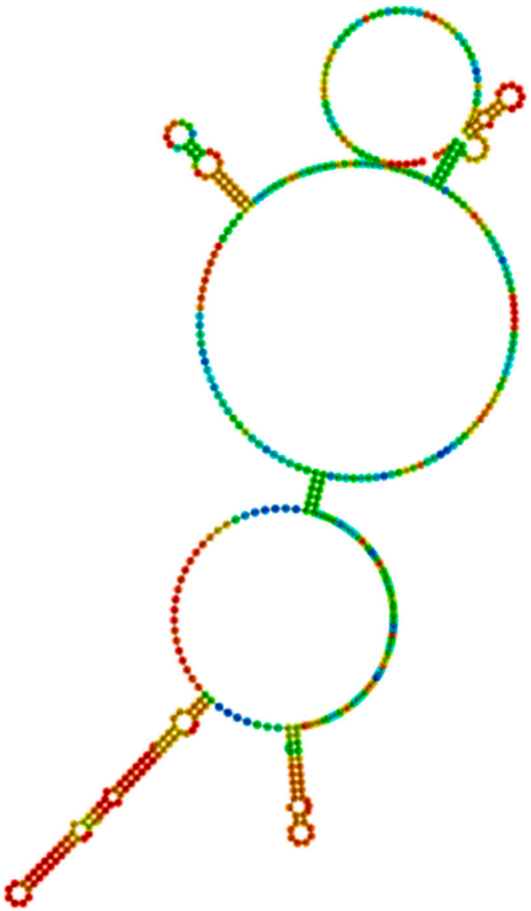

LINC01897

Results for minimum free energy prediction

The minimum free energy of **-68.80** kcal/mol

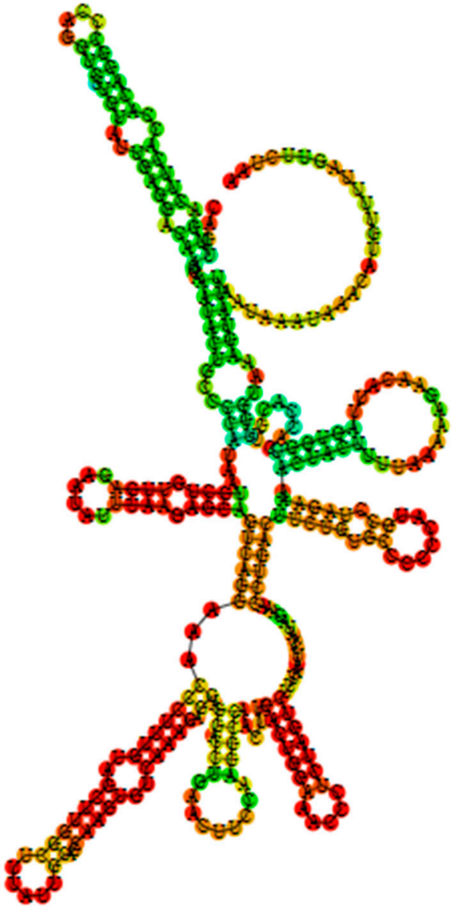

Results for thermodynamic ensemble prediction

The free energy of the thermodynamic ensemble is **-74.17** kcal/mol.

The ensemble diversity is **58.74**.

The minimum free energy of **-53.40** kcal/mol

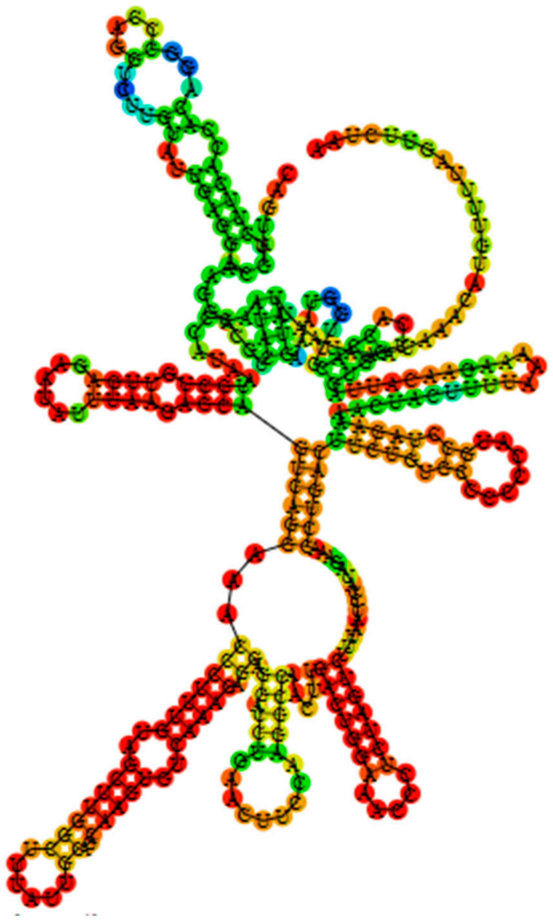

LINC01899

Results for minimum free energy prediction

The minimum free energy of **-112.90** kcal/mol

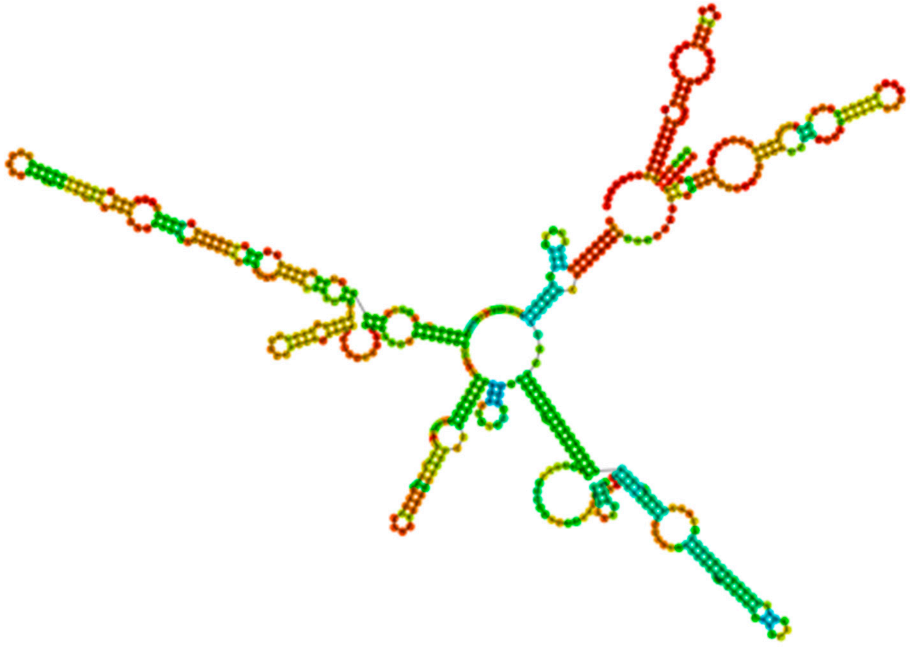

Results for thermodynamic ensemble prediction

The free energy of the thermodynamic ensemble is **-123.63** kcal/mol.

The ensemble diversity is **162.07**.

The minimum free energy of **-80.79** kcal/mol

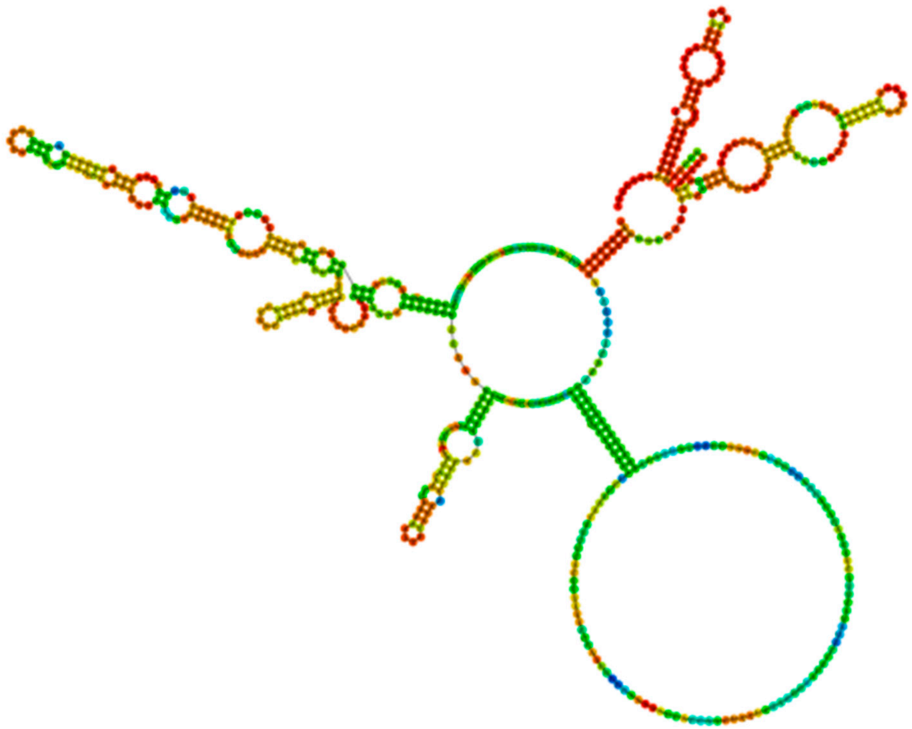

LINC01900

Results for minimum free energy prediction

The minimum free energy of **-121.40** kcal/mol

Results for thermodynamic ensemble prediction

The free energy of the thermodynamic ensemble is **-126.66** kcal/mol.

The ensemble diversity is **119.15**.

The minimum free energy of **-95.80** kcal/mol

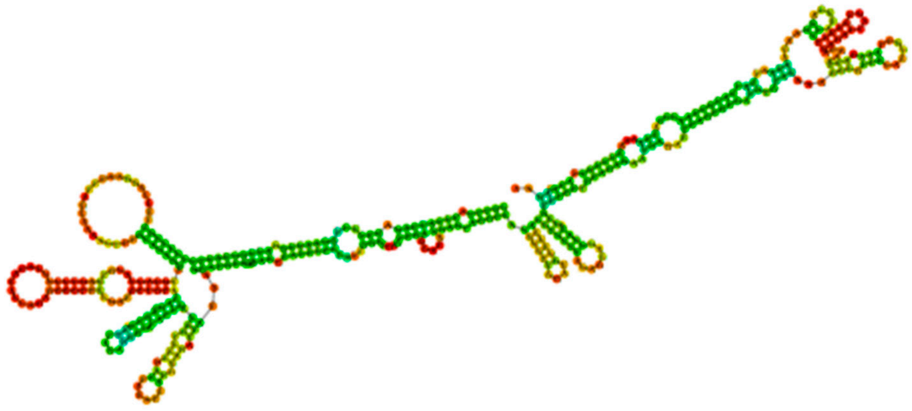

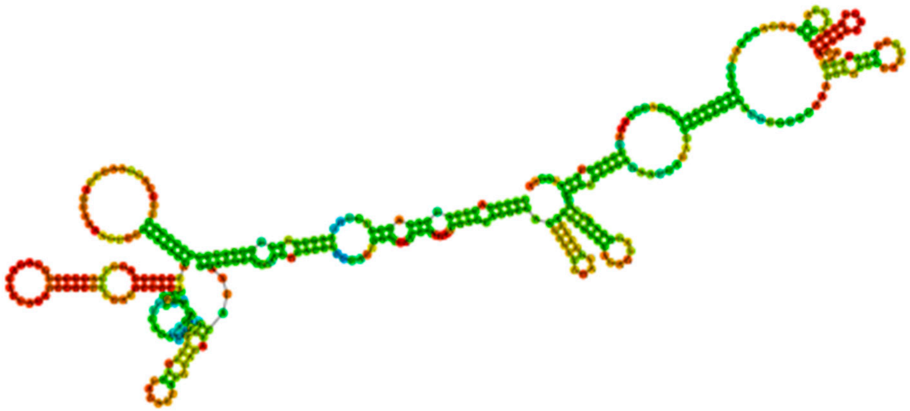

LINC01901

Results for minimum free energy prediction

The minimum free energy of **-398.50** kcal/mol

Results for thermodynamic ensemble prediction

The free energy of the thermodynamic ensemble is **-416.19** kcal/mol.

The ensemble diversity is **321.75**.

The minimum free energy of **-303.41** kcal/mol

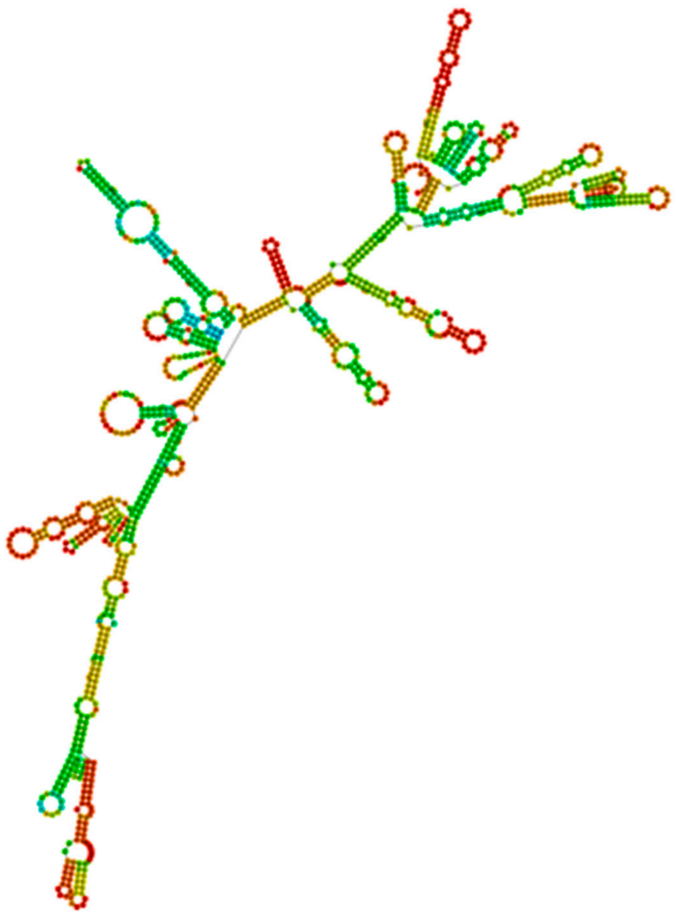

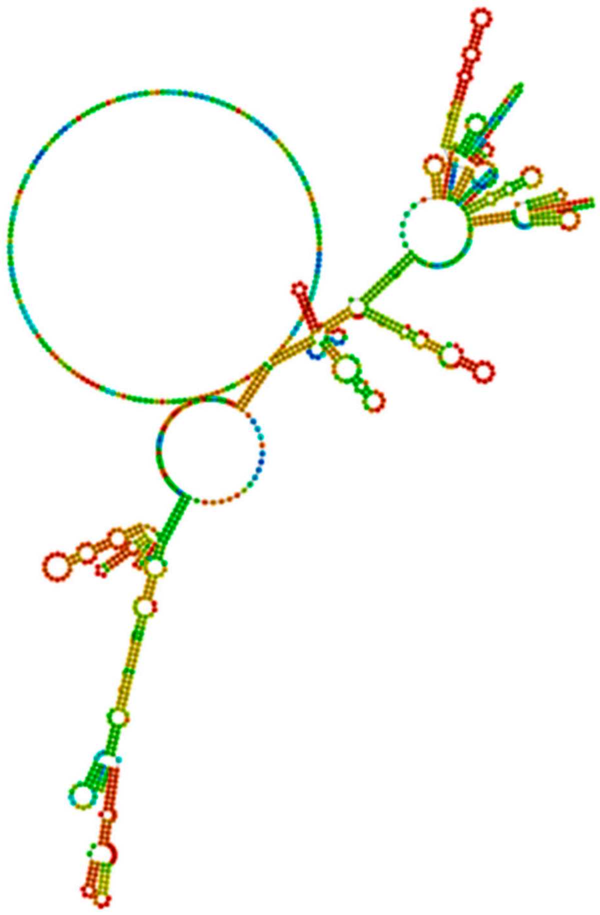

LINC01902

Results for minimum free energy prediction

The minimum free energy of **-1425.40** kcal/mol

Results for thermodynamic ensemble prediction

The free energy of the thermodynamic ensemble is **-1501.12** kcal/mol.

The ensemble diversity is **892.11**.

The minimum free energy of **-1245.47** kcal/mol

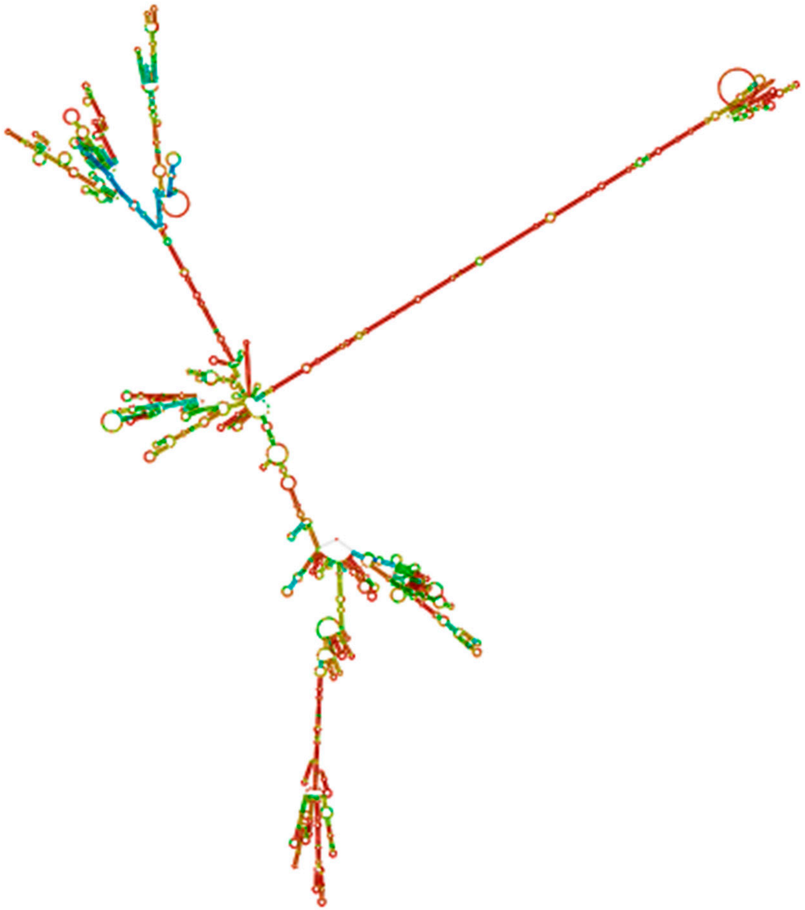

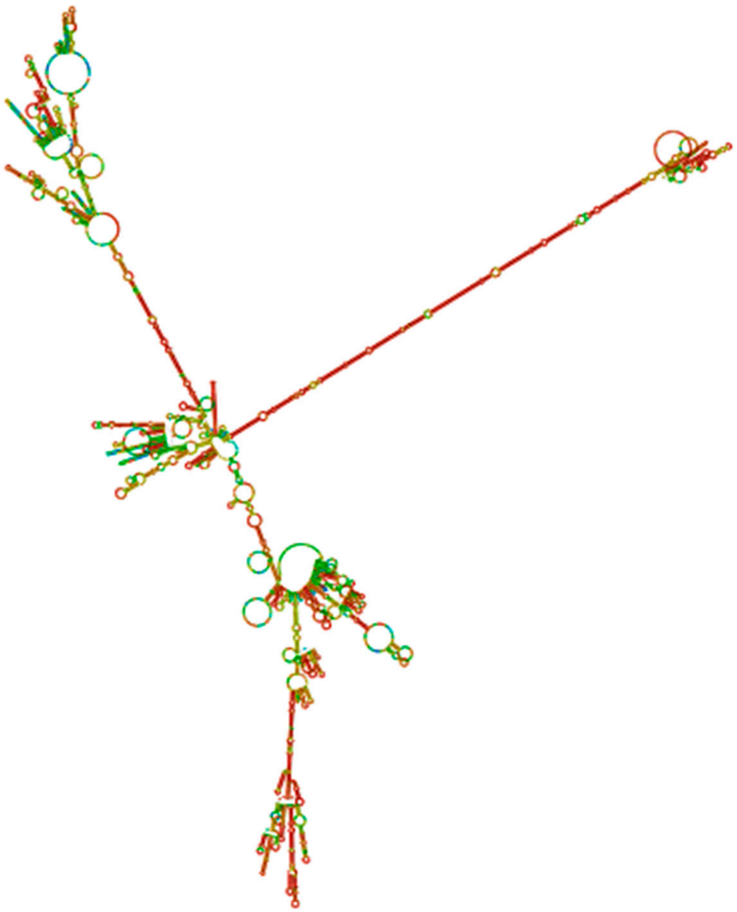

LINC01903

Results for minimum free energy prediction

The minimum free energy of **-484.20** kcal/mol

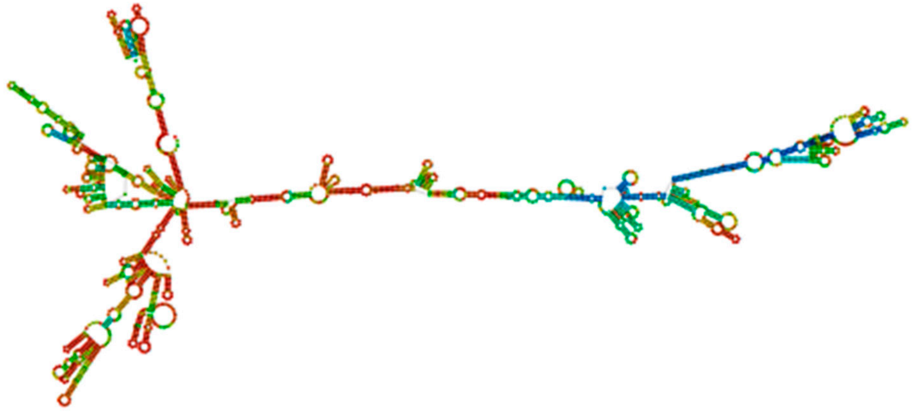

Results for thermodynamic ensemble prediction

The free energy of the thermodynamic ensemble is **-523.76** kcal/mol.

The ensemble diversity is **488.09**.

The minimum free energy of **-364.91** kcal/mol

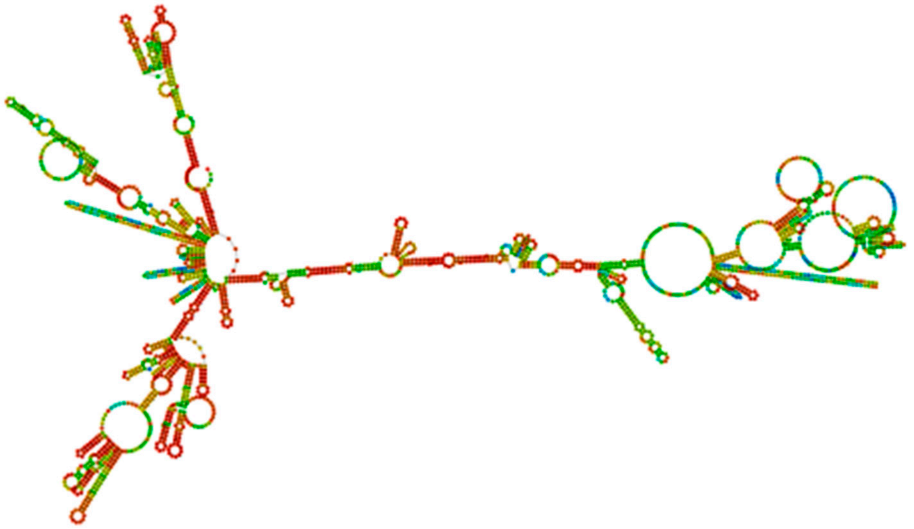

LINC01904

Results for minimum free energy prediction

The minimum free energy of **-253.80** kcal/mol

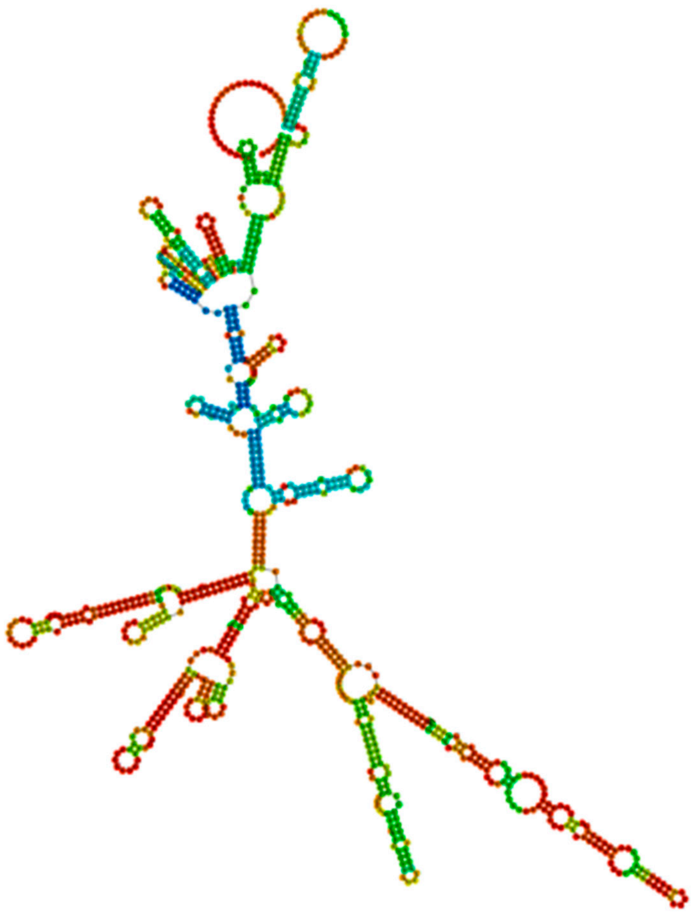

Results for thermodynamic ensemble prediction

The free energy of the thermodynamic ensemble is **-269.15** kcal/mol.

The ensemble diversity is **202.44**.

The minimum free energy of **-185.29** kcal/mol

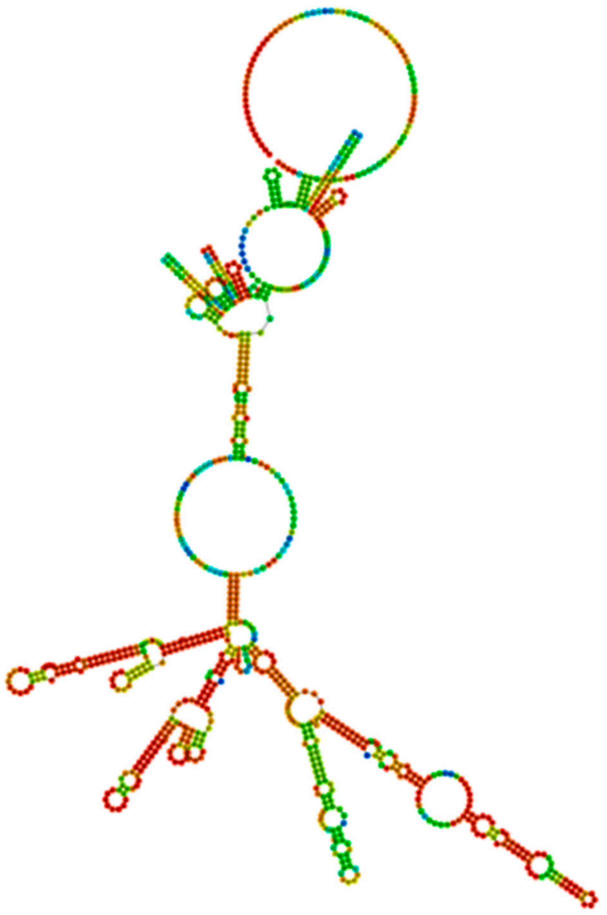

LINC01905

Results for minimum free energy prediction

The minimum free energy of **-150.70** kcal/mol

Results for thermodynamic ensemble prediction

The free energy of the thermodynamic ensemble is **-161.49** kcal/mol.

The ensemble diversity is **67.67**.

The minimum free energy of **-141.60** kcal/mol

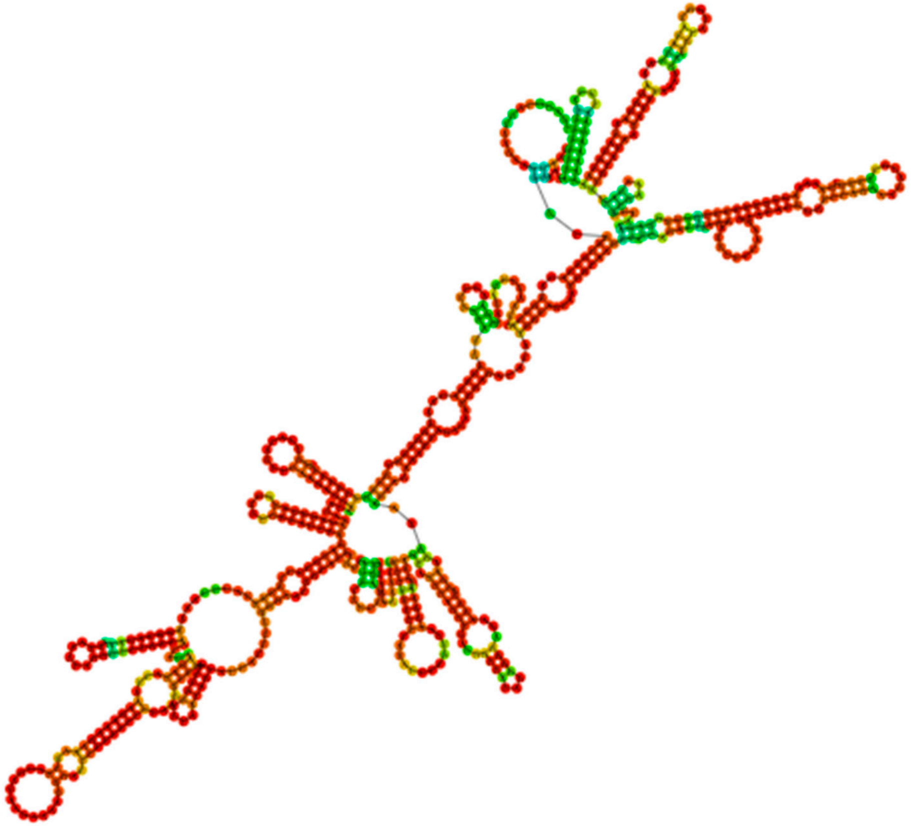

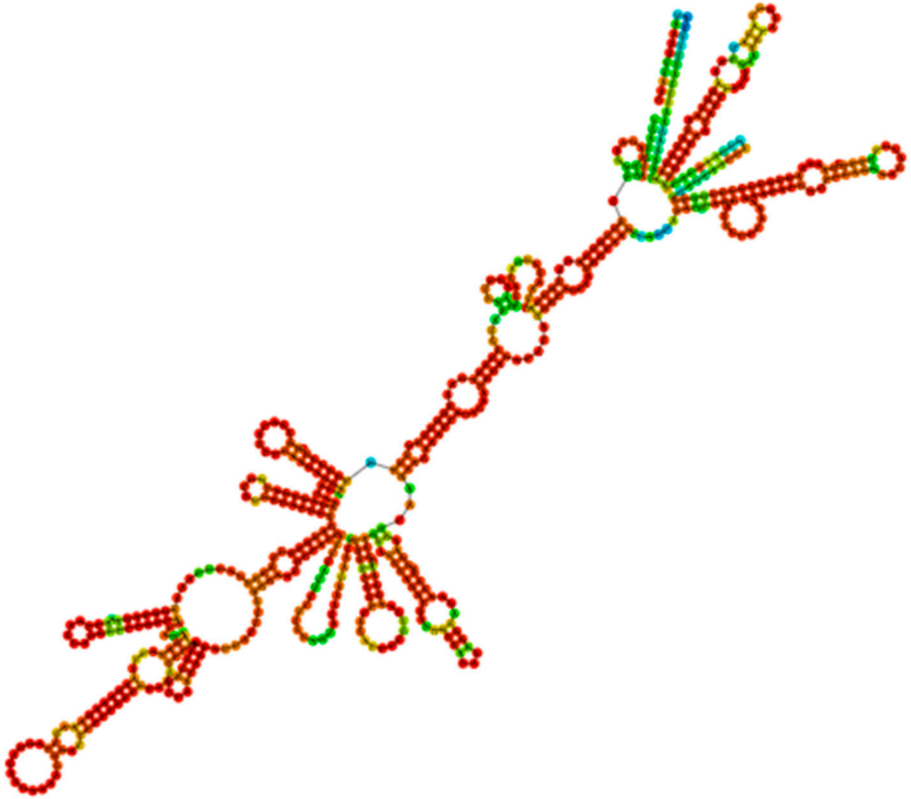

LINC01906

Results for minimum free energy prediction

The minimum free energy of **-129.70** kcal/mol

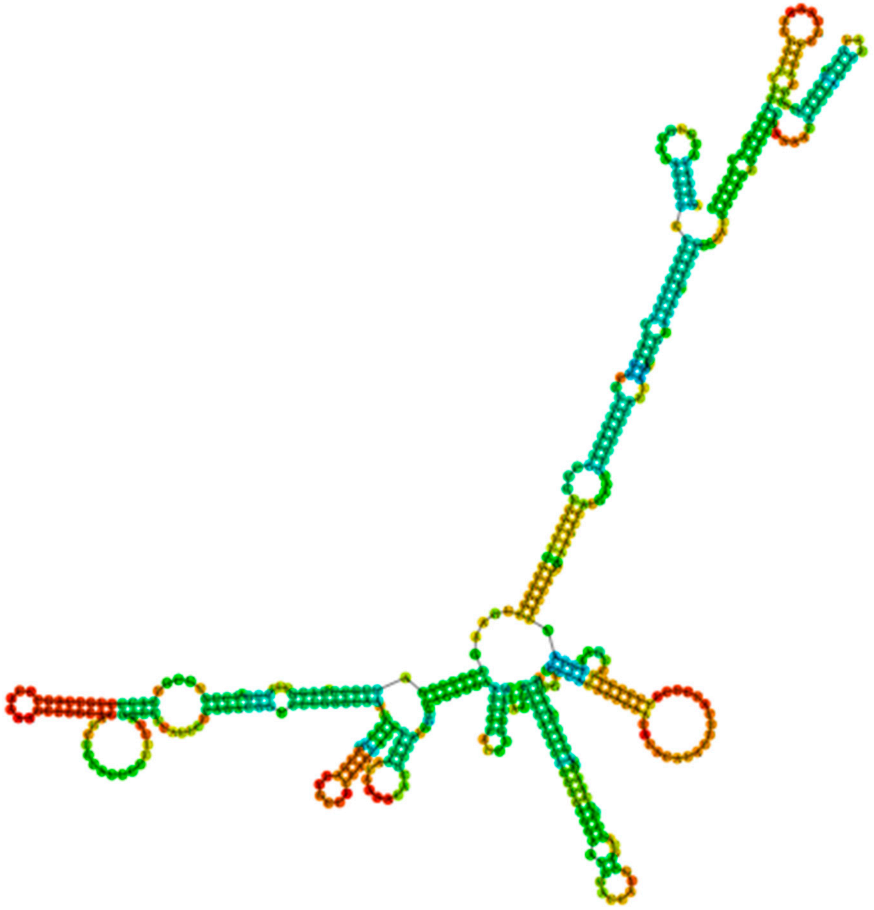

Results for thermodynamic ensemble prediction

The free energy of the thermodynamic ensemble is **-139.48** kcal/mol.

The ensemble diversity is **177.88**.

The minimum free energy of **-69.52** kcal/mol

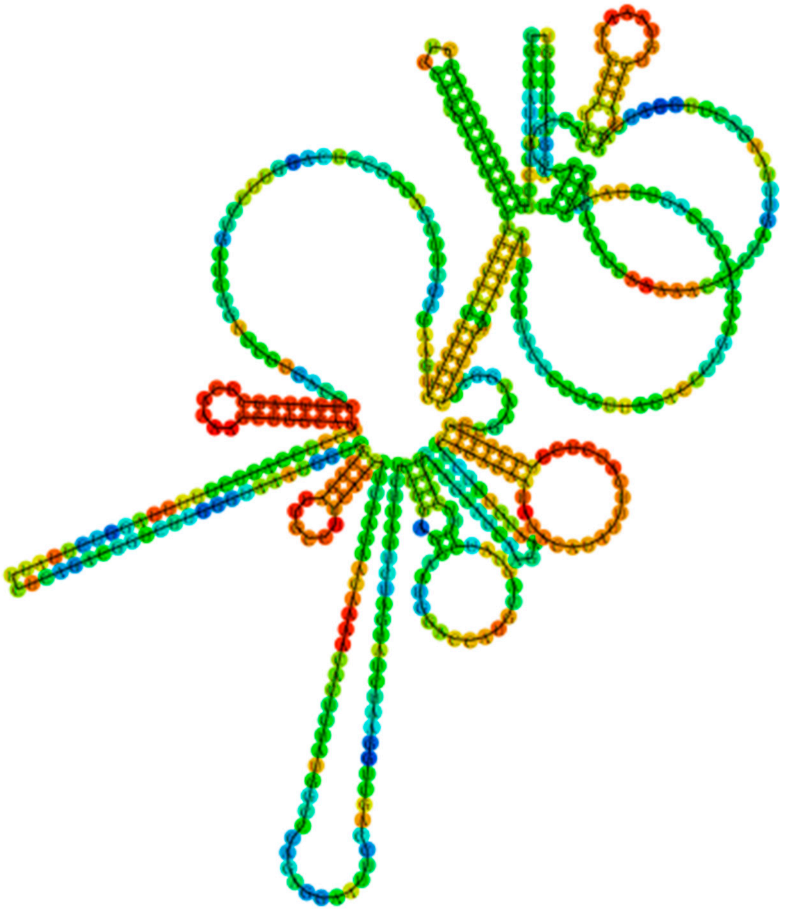

LINC01908

Results for minimum free energy prediction

The minimum free energy of **-251.10** kcal/mol

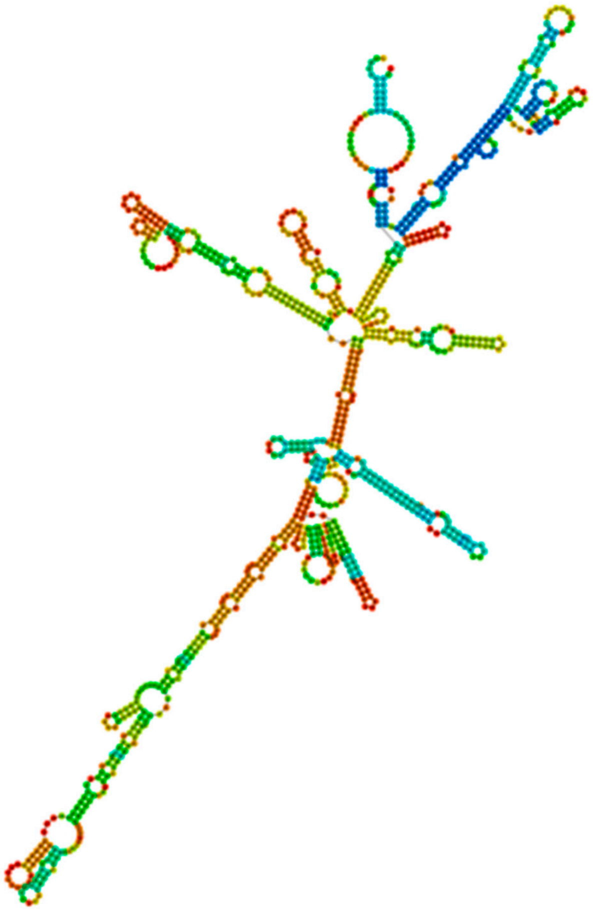

Results for thermodynamic ensemble prediction

The free energy of the thermodynamic ensemble is **-269.75** kcal/mol.

The ensemble diversity is **269.29**.

The minimum free energy of **-166.00** kcal/mol

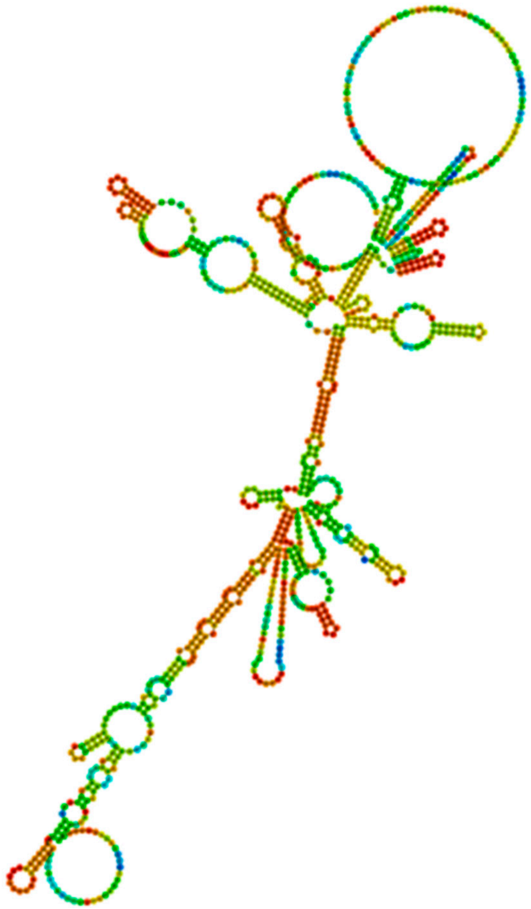

LINC01909

Results for minimum free energy prediction

The minimum free energy of **-221.20** kcal/mol

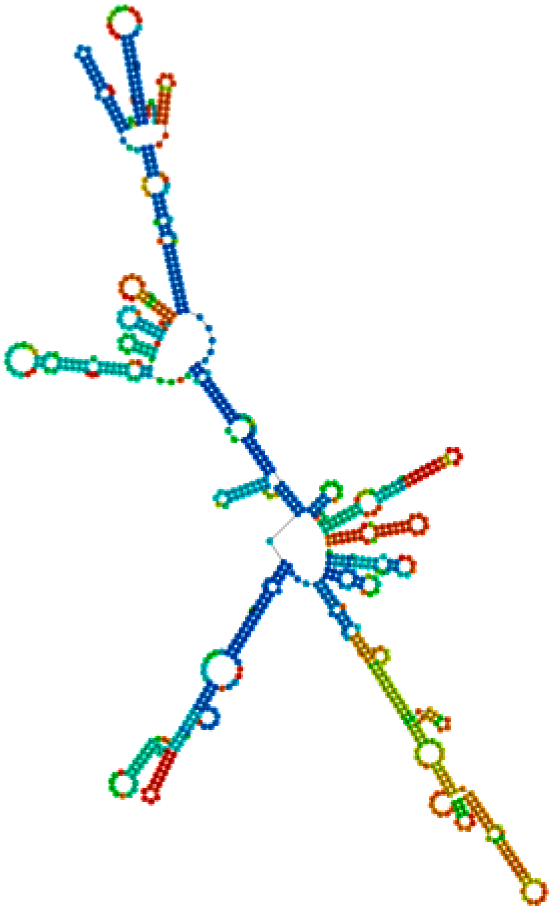

Results for thermodynamic ensemble prediction

The free energy of the thermodynamic ensemble is **-238.28** kcal/mol.

The ensemble diversity is **206.68**.

The minimum free energy of **-175.27** kcal/mol

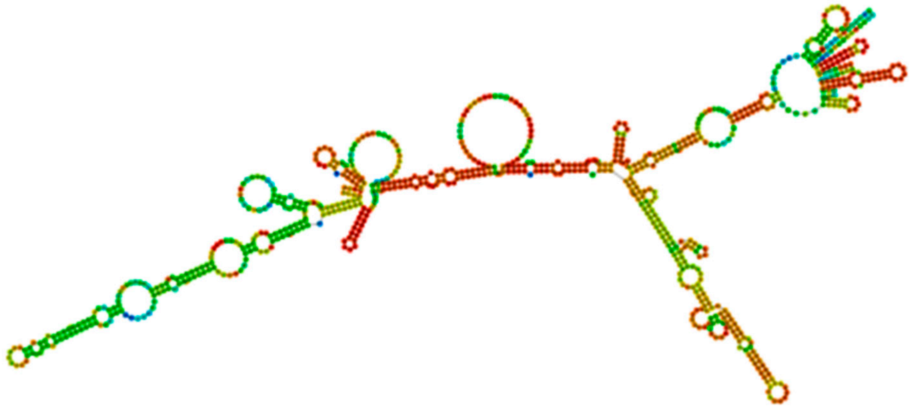

LINC01910

Results for minimum free energy prediction

The minimum free energy of **-772.10** kcal/mol

Results for thermodynamic ensemble prediction

The free energy of the thermodynamic ensemble is **-816.33** kcal/mol.

The ensemble diversity is **592.35**.

The minimum free energy of **-608.97** kcal/mol

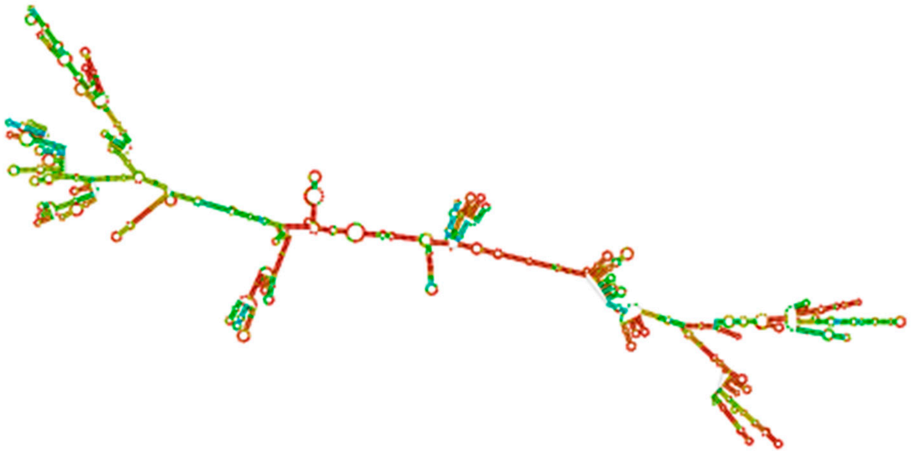

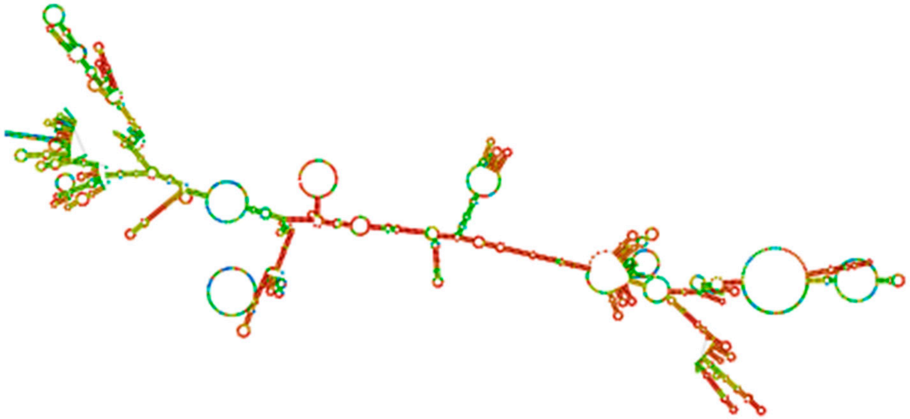

LINC01912

Results for minimum free energy prediction

The minimum free energy of **-490.30** kcal/mol

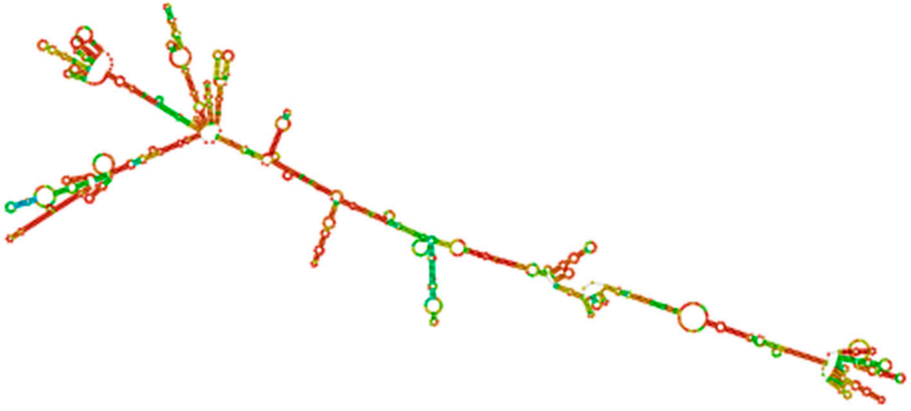

Results for thermodynamic ensemble prediction

The free energy of the thermodynamic ensemble is **-526.25** kcal/mol.

The ensemble diversity is **343.71**.

The minimum free energy of **-424.21** kcal/mol

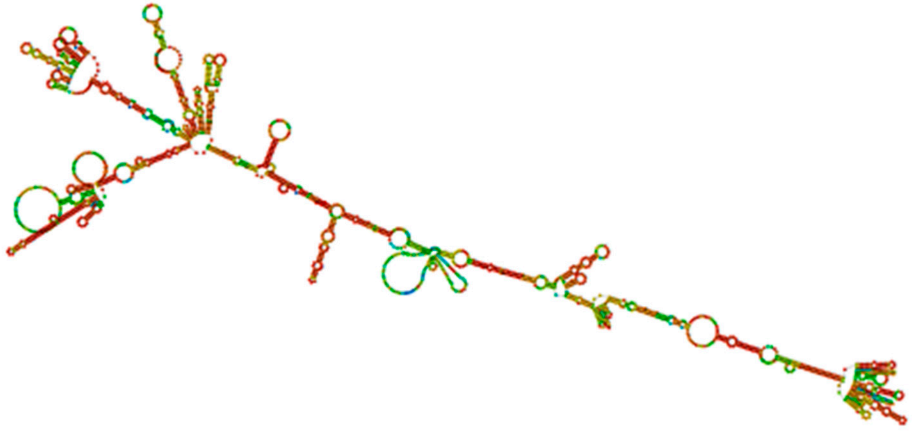

LINC01915

Results for minimum free energy prediction

The minimum free energy of **-616.10** kcal/mol

Results for thermodynamic ensemble prediction

The free energy of the thermodynamic ensemble is **-657.43** kcal/mol.

The ensemble diversity is **624.97**.

The minimum free energy of **-449.05** kcal/mol

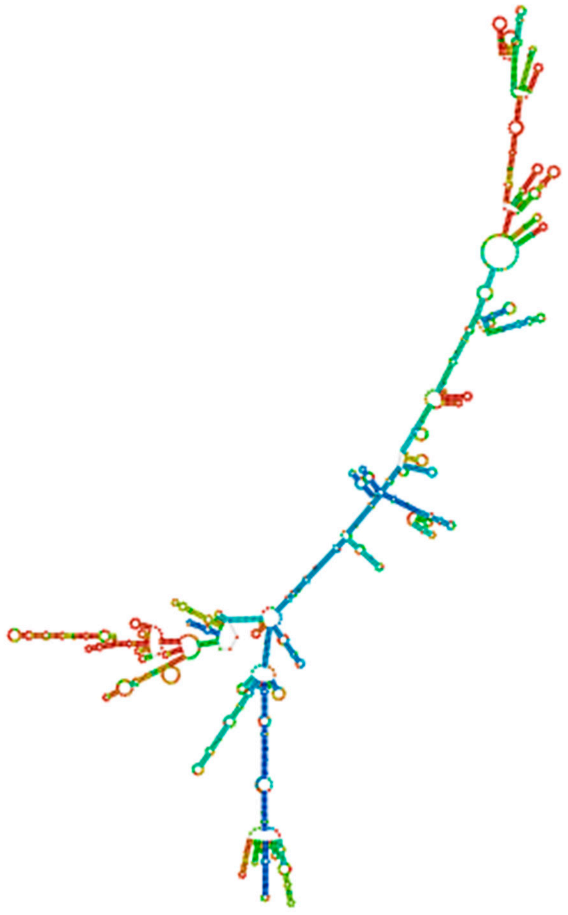

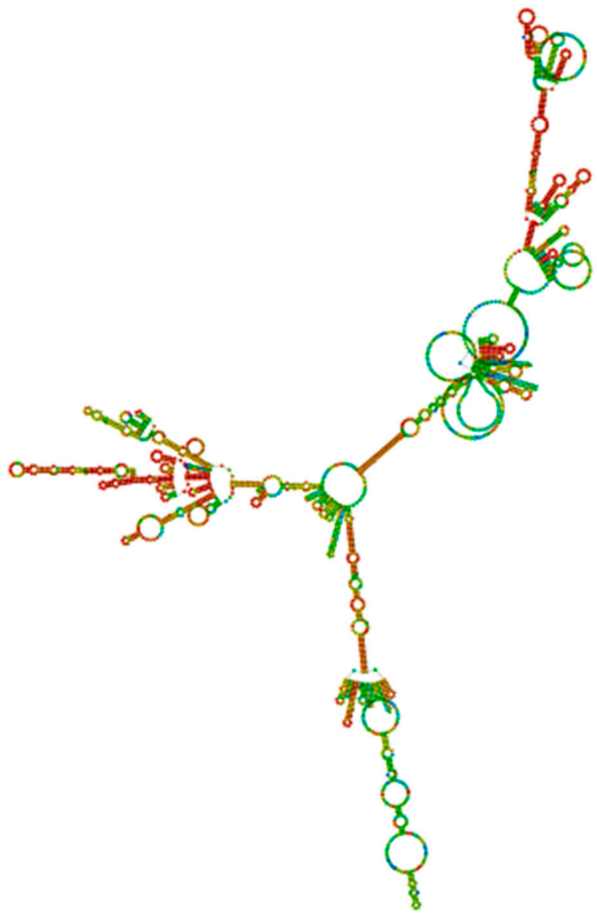

LINC01916

Results for minimum free energy prediction

The minimum free energy of **-292.70** kcal/mol

Results for thermodynamic ensemble prediction

The free energy of the thermodynamic ensemble is **-315.55** kcal/mol.

The ensemble diversity is **282.14**.

The minimum free energy of **-236.94** kcal/mol

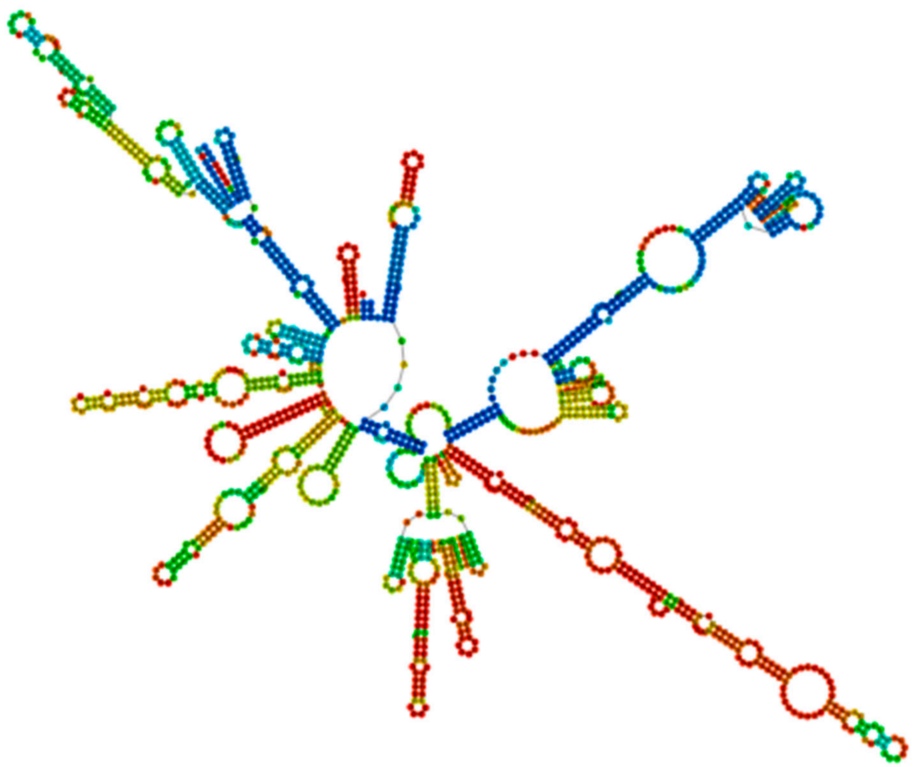

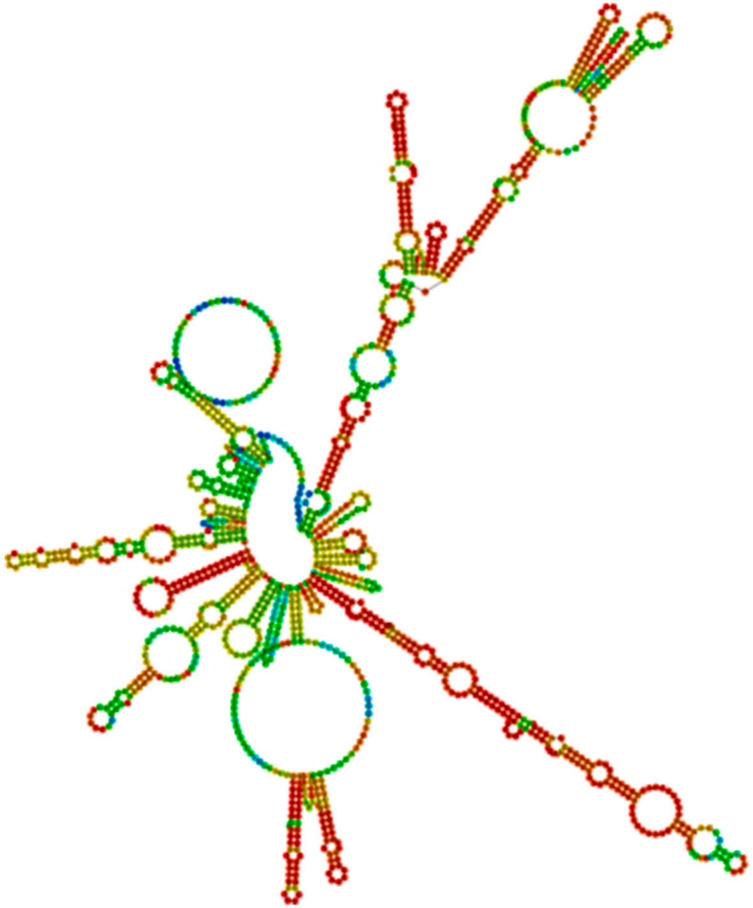

LINC01917

Results for minimum free energy prediction

The minimum free energy of **-243.20** kcal/mol

Results for thermodynamic ensemble prediction

The free energy of the thermodynamic ensemble is **-262.19** kcal/mol.

The ensemble diversity is **283.87**.

The minimum free energy of **-192.35** kcal/mol

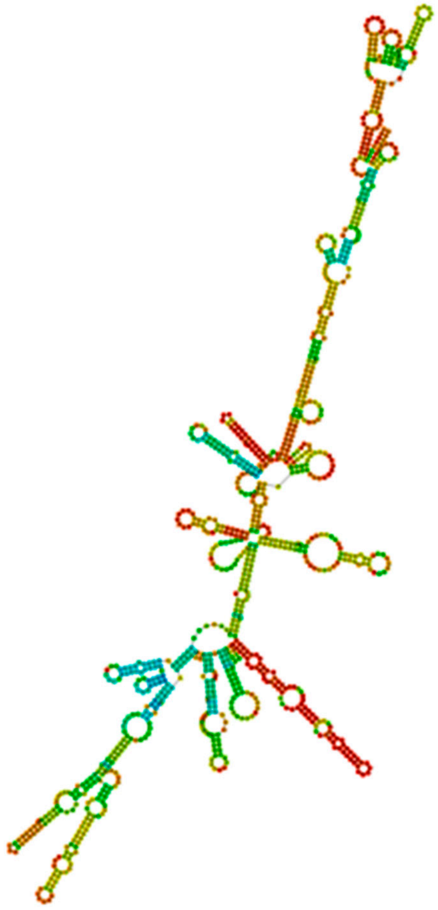

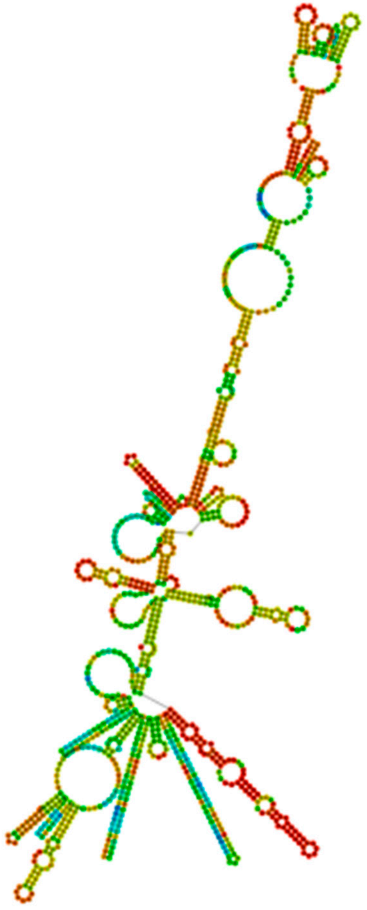

LINC01919

Results for minimum free energy prediction

The minimum free energy of **-141.90** kcal/mol

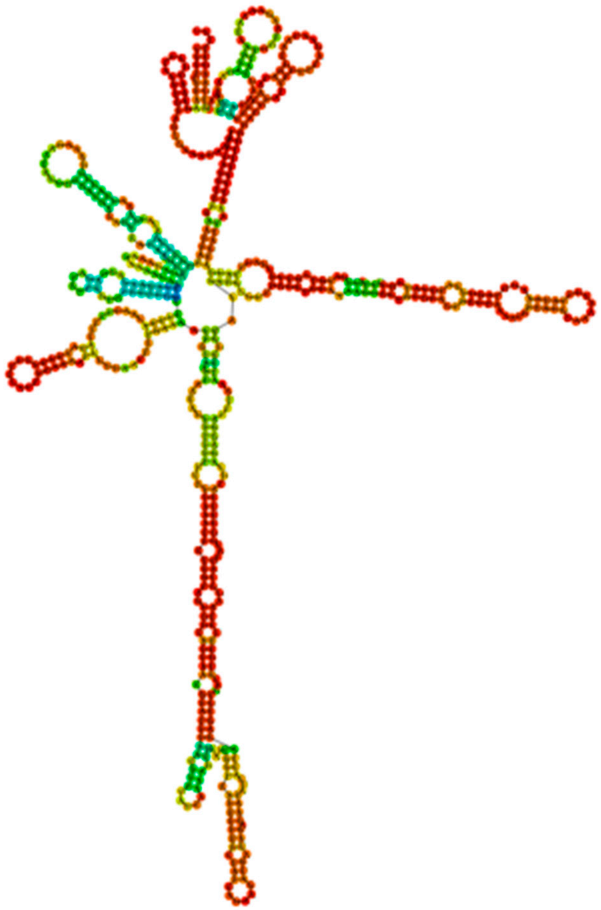

Results for thermodynamic ensemble prediction

The free energy of the thermodynamic ensemble is **-150.72** kcal/mol.

The ensemble diversity is **101.16**.

The minimum free energy of **-124.30** kcal/mol

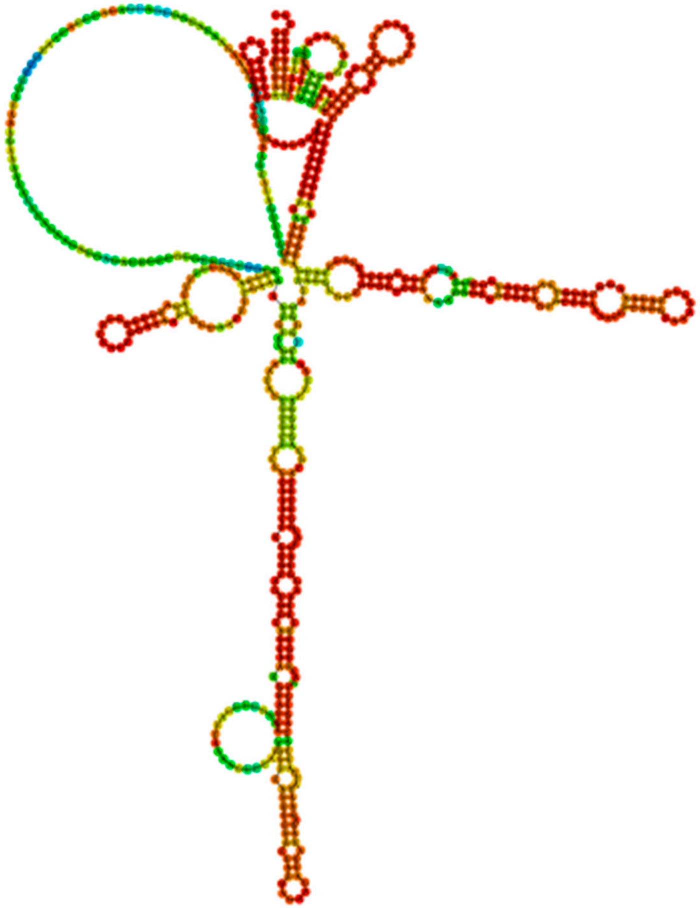

LINC01922

Results for minimum free energy prediction

The minimum free energy of **-638.10** kcal/mol

Results for thermodynamic ensemble prediction

The free energy of the thermodynamic ensemble is **-667.45** kcal/mol.

The ensemble diversity is **301.77**.

The minimum free energy of **-556.98** kcal/mol

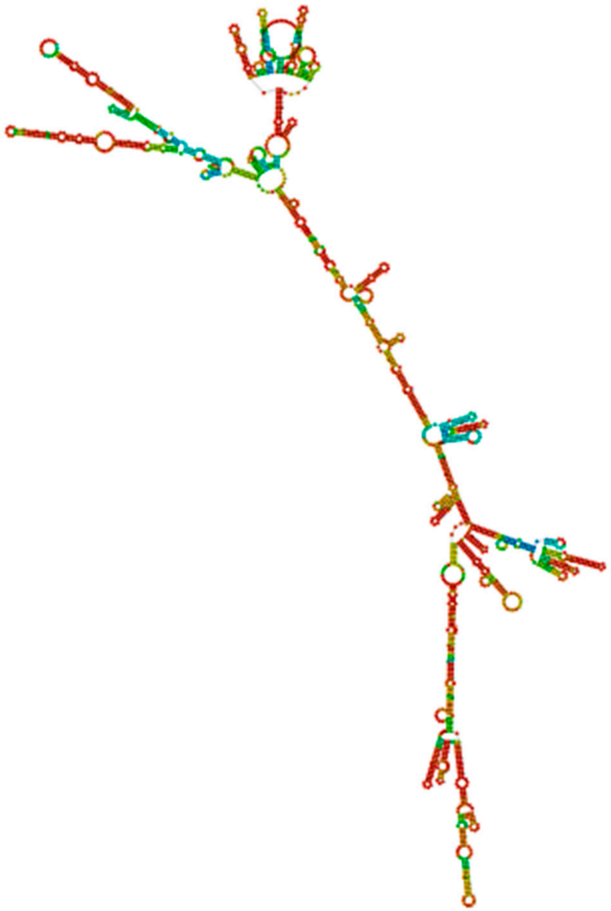

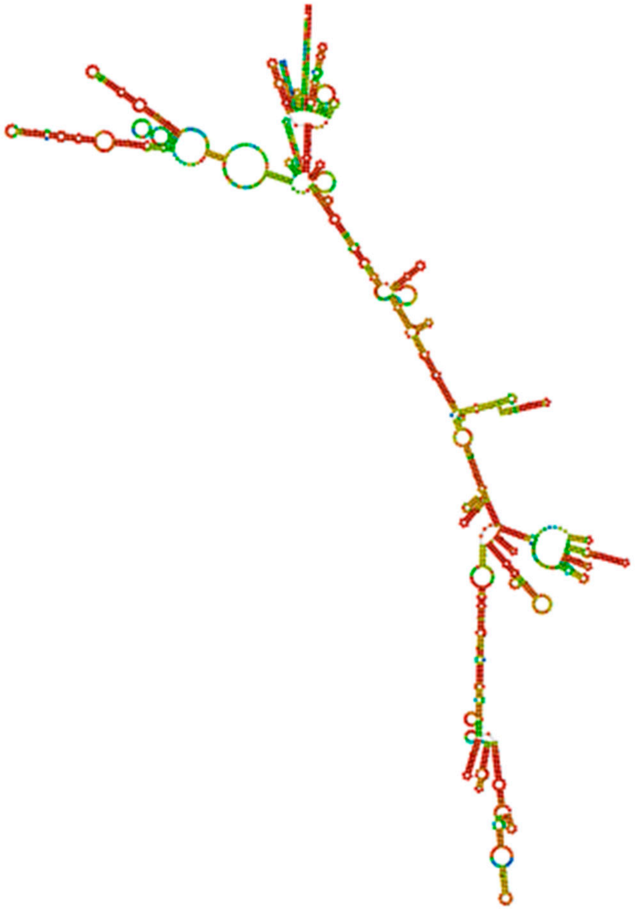

LINC01924

Results for minimum free energy prediction

The minimum free energy of **-469.70** kcal/mol

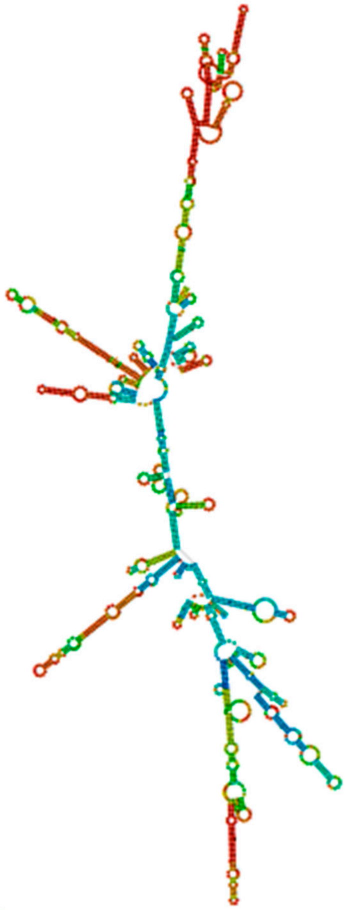

Results for thermodynamic ensemble prediction

The free energy of the thermodynamic ensemble is **-498.20** kcal/mol.

The ensemble diversity is **425.92**.

The minimum free energy of **-380.30** kcal/mol

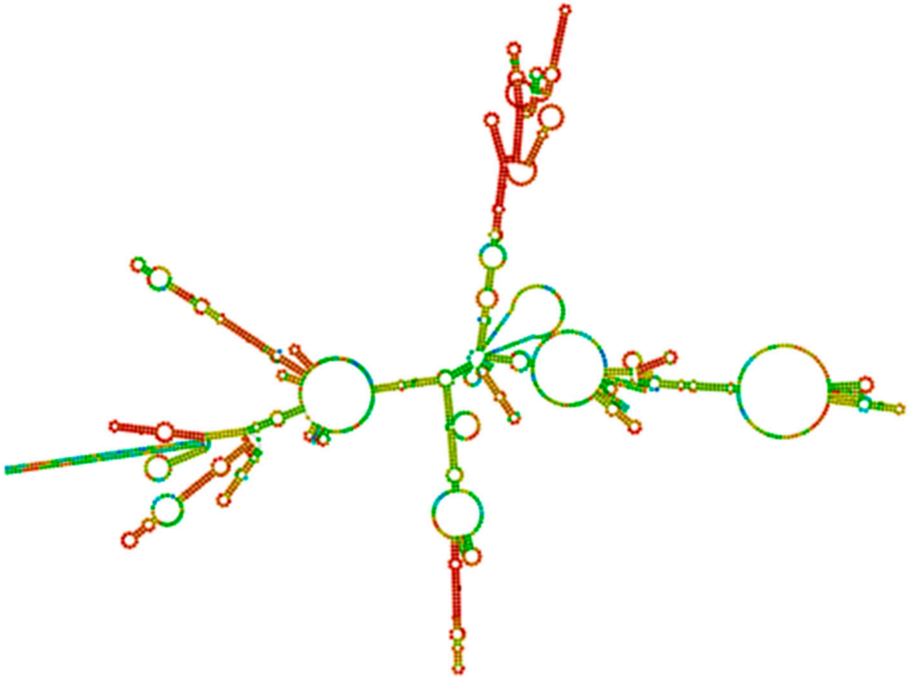

Supplement: Supplementary file 1 [file biomedicines-12-00544-s001.zip › Table_S22_Ershov.pdf]
