# Supplementary material for: Long Intergenic Non-Coding RNAs of Human Chromosome 18: Focus on Cancers
Source: Biomedicines. 2024 Feb 28;12(3):544. doi: 10.3390/biomedicines12030544 (PMC10968066; doi:10.3390/biomedicines12030544)
Supplement: Supplementary file 1 [file biomedicines-12-00544-s001.zip › Table_S24_Ershov.pdf]

Table S24. Local sequence alignment and similar secondary structures motifs of lincRNAs of human chromosome 18.

Local sequence alignment*	Protein interactors**
**LINC01916** **LINC01415**<br>**Rank** **Start** **Stop** **Strand** **Start** **Stop** **Strand** **Score** **%id** **P-Value** **E-Value**<br>1 826 1018 + 5544 5736 + 454 45 6.40E-02 6.60E-02<br><br>. . . . . 1 . . . . 2 . . . . 3 . . . . 4 . . . . 5 . . . . 6<br>A U U G A A U U U U A A U A U U U A U A U U A U G A A A U G A U C U G C C A C U G U U U U U U G U A A G U G U A C C U G<br>A U U U C U A U C G A A U C U U U A U G C A A U U A G G U U A C U G A A C U U U U G A A A U C A A A G G A G G A A A U C<br><br>. . ((((((. (((((. . . . . ((. . . . . .)) . . . . ((. ((((. . . . .)))) . . . .))<br>. . E E E E E E . C C C C C . . . . . A A . . . . . . a a . . . . B B . B B B B . . . . b b b b . . . . b b<br><br>. . . . . 7 . . . . 8 . . . . 9 . . . . 0 . . . . 1 . . . . 2<br>C U A U U A U G A C U A U A U U U U C A A G U U A A A G A U U C A U G A C G U U U U U G U G A G A A A A U A U U U - U A<br>A G A U U U U G U G G U U U U G C C C A G U A U A C U A U A G A A U G U C A A U A U U G U U C U A U U A U A A A A A U A<br><br>.))))) ((((. . . . .)))) . .)))))) . ((((((((((. . . . . ((. . . .)) .))<br>. c c c c c D D D D . . . . d d d d . . e e e e e e . J J J F F F F F F F . . . . . F F . . . . f f . f f<br><br>. . . . . 3 . . . . 4 . . . . 5 . . . . 6 . . . . 7 . . . . 8<br>A A A A C U U G U - U A A U U U U U U C A G A U U A A C U A C A A A A A A U C U A U U G U A A A A A U U U A U A A A A U<br>A U A U U C U A U A U A U U A U A U U C U U A U A - - U U A G A A C A A U A C U A U U C U A U A U A U A A U A U G C U A<br><br>))))) . . . . . ((((. . . . . . (((((. . . (((((. . . . .))))) . .))))) . ((. . . .<br>f f f f f . . . . . I I I I . . . . . . G G G G G . . . G G G G G . . . . g g g g g . . g g g g g . H H . . . .<br><br>. . . . . 9 . . . . .<br>A C U A A U A A U U A A G U C<br>A U A U A A G A A U A A G A C<br><br>. .)) . . .)))) .)))<br>. . h h . . . i i i i . j j j<br>Актив	PABPC1<br>MBNL1<br>RBMX<br>KHDRBS3<br>ELAVL1<br>SFRS13A
**LINC01916** **LINC01415**<br>**Rank** **Start** **Stop** **Strand** **Start** **Stop** **Strand** **Score** **%id** **P-Value** **E-Value**<br>2 599 657 + 4381 4440 + 447 57 7.45E-02 7.74E-02<br><br>. . . . . 1 . . . . 2 . . . . 3 . . . . 4 . . . . 5 . . . . 6<br>U A U A U G U C A U G A U U A A U G U U A U C U A U - A A U G A G U G U A U U A G G G A U A C A U U A A U A U U C A C A<br>U A C A U A A A A A U G U U A A A G A U U U A U U U A G A U A A A U G U A U C A A G U A U U U U U U A A U U U U U U U A<br><br>. . . . (((. ((. . (((((((. . (((. . . ((((. . . .)))) . .))) .))))))) .)) . .)))<br>. . . . A A A . A A . . A A A A A A A . . A A A . . . A A A A . . . . a a a a . . a a a . a a a a a a a . a a . . a a a	Pum2<br>KHDRBS3

	LINC02564			LINC01919						
Rank	Start	Stop	Strand	Start	Stop	Strand	Score	%id	P-Value	E-Value
1	65	265	+	212	408	+	645	34	2.50E-03	2.50E-03

```
.........1.........2.........3.........4.........5.........6
GCGGCGGUCCAGGGCCGCCUGGCGCAGCAGCCUGUCCCAGCCGCGGUCCCUGCAGUCCCU
UUUUUCUUCCUGAAACCUCUGCAGUGGUAG--CAAUGCACUGAAGUUUGAUUAAAAGUUU

(((((((....(((((((((((..((......))..)))((...((..............
IIIGGGG....GGGGGGEEAAA..AA......aa..aaaDD...BB..............

.........7.........8.........9.........0.........1.........2
CCCUGGCGGCUGCGCAGCCGUCCCACGACAGGGGCCAUAAACUCUCCAG-AGCGGAAAGC
ACUGGUCAUUCCUGCCAUGAGAAGAUGAUAGAGAAUAUCACCACAGUGUGACUUGCUUCC

))(((.((....)))))..))...........))...........((((..((((....)
bbCCC.CC....ccccc..dd...........ee...........FFFF..FFFF....f

.........3.........4.........5.........6.........7.........8
CGCACCCUGGUGGCCCGGCCCCGCGCCCAGACCUGGCGGCCGCUGGCACCUGACCCGCUG
AAGGCAGCAUGGUUUCAUGCAGAGGAUCUAAACUGGGGACCAUGUGC---UGGGCUGCAA

)))...))))))))))....))))....((.((((((..((...(((.........))).
fff...ffffgggggg....gggg....HH.HHHHHH..HH...HHH.........hhh.

.........9.........0..
CAUGGGUCUCCAGGGAGCUCGC
AAGGGCUGUGCAGUGCCUAAAA

...))))...))))...)))))
...hhhh...hhhh...hhiii
```

a2bp1
EIF4B
MBNL1
Vts1
RBMX
KHDRBS3

Актив

	LINC02564			LINC01919						
Rank	Start	Stop	Strand	Start	Stop	Strand	Score	%id	P-Value	E-Value
2	617	718	+	386	485	+	416	48	8.06E-02	8.41E-02

```
.........1.........2.........3.........4.........5.........6
AAGUAGCUGGAACUACAGAUGUGCACUGCCAUGCCAGGCUUGUCUAACAUU-UUUAUGUG
AAAGGGCUGUGCAG---UGCCUAAAAUGUUAUGCAAAAUUUUAUAAAGGUAGGUCAUGUA

((.((((...(((....(((((.((..(((......))).))....)))))....(((..
EE.CCCC...CCC....AAAAA.AA..AAA......aaa.aa....aaaaa....BBB..

.........7.........8.........9.........0...
UUGCUUCAUCCAGUUUGCUAGAGUUUUUGGAGAUUUCUGUCUU
UUGCUCCAUUGAUGCUGCCCUUAUUCAUGAUGAUUAAAGCCUU

......)))...))).))))(((.((.....)).)))....))
......bbb...ccc.ccccDDD.DD.....dd.ddd....ee
```

MBNL1
RBMX
ELAVL1
KHDRBS3

	LINC02564			LINC01925						
Rank	Start	Stop	Strand	Start	Stop	Strand	Score	%id	P-Value	E-Value
1	65	265	+	27	226	+	1200	41	1.80E-03	1.80E-03

KHSRP
RBMX

```
.........1.........2.........3.........4.........5.........6
GCGGCGGUCCAGGGCCGCCUGGCGCAGCAGCCUGUCCCAGCCGCGGUCCCUGCAGUCCCU
GCCCGGAGGUCUGGCCGAGGUCCG-AGGAGGGGCCCGGAGGUCUGGCCGAGGUCCGAGGA

(((((((((((((((((...(((......))).((....))..))))).))).((..((.
MMMKKKKKKCCCCCCCC...AAA......aaa.BB....bb..ccccc.ccc.JJ..DD.

.........7.........8.........9.........0.........1.........2
CCCUGGCGGCUGCGCAGCCG---UCCCACGACAGGGGCCAUAAACUCUCCAGAGCGGAAA
GGGGCCCGGAGGUCUGGCCGAGGUCCGAGGAGGGGCCCGGAGGUCUGGCC-GAGGUCCGA

....))((((...((.((((....((......((((((......((......)).((...
....ddIIII...GG.GGGG....GG......GGGGGG......EE......ee.FF...

.........3.........4.........5.........6.........7.........8
GCCGCACCCUGGUGGCCCGGCCCCGCGCCCAGACCUGGCGGCCGCUGGCACCUGACCCGC
GGAGGGGCCCGGAGGUCUGGCCGAGGUCCGAGG--AGGGGUCCGGAGGUCUGGCCGAGGU

.)))).))))...))..))))...))(((.......))).)))).......))))....)
.ffgg.gggg...gg..gggg...ggHHH.......hhh.iiii.......jjkk....k

.........9.........0....
UGCAUGGGUCUCCAGGGAGCUCGC
CCGAGGAGGGCCCGGAGGUCUGGC

)))...((.((((....))))))))
kkk...LL.LLL....lllllmmm
```

	LINC02564			LINC01925						
Rank	Start	Stop	Strand	Start	Stop	Strand	Score	%id	P-Value	E-Value
2	497	678	+	68	249	+	639	35	1.02E-02	1.03E-02

RBMX
SFRS1

```
.........1.........2.........3.........4.........5.........6
CACCCUGCAGCUCCAGAUGAGGCCUCACUCUGUCACCCAGGUUGGGGUGGAGUGGCACAG
CUGGCCGAGGUCCGAGGAGGGGCCCGGAGGUCUGGCCGAGGUCCGAGGAGGGGCCCGGAG

((((((...((((.....)).)).(((...((.....))...))).....)).))(((..
MMCCCC...AAAA.....aa.aa.BBB...BB.....bb...bbb.....cc.ccLLL..

.........7.........8.........9.........0.........1.........2
UCACAGCUCACUAUAACCUCAAGCUCCUGGGCUCAAGUGAUCCUGCCACCUCAGCCUCCU
GUCUGGCCGAGGUCCGAGGAGGGGCCCGGAGGUCUGGCCGAGGUCCGAGGAGGGGUCCGG

..((((((.............))..(((((...((((((..((.((.......))...((
..JJJJDD.............dd..IIIII...IIIIII..GG.EE.......ee...FF

.........3.........4.........5.........6.........7.........8
AAGUAGCUGGAACUACAGAUGUGCACUGCCAUGCCAGGCUUGUCUAACAUUUUUAUGUGU
AGGUCUGGCCGAGGUCCGAGGAGGGCCCGGAGGUCUGGCCGAGGUCCGAGGAGGGCCCGG

....))..))....((....)).))))....))))))))).)).(((.....))).))).
....ff..gg....HH....hh.iiii....iiiiiiijj.jj.KKK.....kkk.lll.

..
UG
AG

))
mm
```

	LINC02564			LINC01925						
Rank	Start	Stop	Strand	Start	Stop	Strand	Score	%id	P-Value	E-Value
3	34	173	+	227	371	+	519	39	1.48E-02	1.49E-02

```
.........1.........2.........3.........4.........5.........6
CCUGGGCUGCGAGGGCGGCUUAGGGGCAGAAGCGGCGGUCCAGGGCC-GCCUGGCGCAGC
CGAGGUCCGAGGAGGGCCCGGAGGUCUGGCCGAGGUGAUAAUAGUUAAGCCUAGAGCCAU

.(((((((.....)))(((((........))).(((......((((..((..((.(((..
.KKKKAAA.....aaaJJBBB........bbb.III......EEEE..CC..CC.CCC..

.........7.........8.........9.........0.........1.........2
AGCCUGUCCCAGCCGCG-GUCCCUGCAGUCCCUCC-CUGGCGGCUGCGCAGCCGUC-CCA
GAGAGGCCUGUGCUCCGUGGGACAGGAGGCUAAGAGCGGAACAGGGCCCACUGUUGGAUG

....)))))..)).((........)).))))..((.(((.((((......))))((....
....ccccc..cc.DD........dd.eeee..HH.HHH.FFFF......ffffGG....

.........3.........4.....
CGACAG-GGGCCAUAAACUCUCCAG
CUACUGAUCACCAGACUGUGGCCUU

.))))).))))).......))))))
.gghhh.hhiii.......jjkkkk
```

KHSRP
RBMX
KHDRBS3

	LINC02564			LINC01925						
Rank	Start	Stop	Strand	Start	Stop	Strand	Score	%id	P-Value	E-Value
4	327	496	+	116	284	+	464	35	1.75E-02	1.76E-02

```
.........1.........2.........3.........4.........5.........6
AGGGGCCUAUGUGAGCCAAAGAGGAGAGAAGGUGAUGCCUCAGCCCAGUGUUUCUGCCCC
AGGGGCCCGGAGGUCUGGCCGAGGUCCGA-GGAGGGGCCCGGAGGUCUGGCCGAGGUCCG

...(((....((((((((......((....((.....))((((((((...(((....(((
...GGG....GGGEEEEE......EE....AA.....aaDDDDDDDD...DDD....BBB

.........7.........8.........9.........0.........1.........2
ACCUCGCUUGUGGCCUUCGGAACUUGAUUUGCACCGCAGGAAAAUGGGCAAUGAAAACCC
AGGAGGGGUCCGGAGGUCUGGCCGAGGUCCGAGGAGGGCCCGGAGGUCUGGCCGAGGUCC

(.......)).)).(((((((.......))....)).)))))).)))))..)))......
B.......bb.bb.CCCCCCC.......cc....cc.cccddd.ddddd..ddd......

.........3.........4.........5.........6.........7
CUCCCUAACUGGCUUCUCAGUCCACUCUGACCAGCCCACUGCACAGCGCC
GAGGAGGGCCCGGAGGUCUGGCCGAGGUGAUAAUAGUUAAGCCUAGAGCC

)).......)))))...((.......((....)).....)))))...)))
ee.......eeeee...FF.......FF....ff.....ffggg...ggg
```

FUS
SFRS9
KHSRP
RBMX
SFRS1
KHDRBS3

	LINC02564			LINC01925						
Rank	Start	Stop	Strand	Start	Stop	Strand	Score	%id	P-Value	E-Value
5	284	371	+	26	110	+	439	45	1.89E-02	1.91E-02

```
.........1.........2.........3.........4.........5.........6
GGCUCGGCUCCCUCGUACACUCUCUGGCAAGCCCAAGAAAGUCAGGGGCCUAUGUGAGCC
GGCCCGGAGGUCUGGCCGAGGUCCGAGGAGGGGCCCGGAGGUCUGGCCGAGGUCCGAG--

((((.((.(((((......(((((..(((..(((...........)))....))).....
AAAA.AA.AAAAA......AAAAA..AAA..AAA...........aaa....aaa.....

.........7.........8........
AAAGAGGAGAGAAGGUGAUGCCUCAGCC
-GAGGGGCCCGGAGGUCUGGCCGAGGUC

...)).)))...))).))..))..))))
...aa.aaa...aaa.aa..aa..aaaa
```

SFRS9
SFRS1

**LINC02564** / **LINC01925**<br>Rank 6; LINC02564: Start 528, Stop 552, Strand +; LINC01925: Start 25, Stop 49, Strand +; Score 371; %id 44; P-Value 2.33E-02; E-Value 2.35E-02<br><br>.........1.........2.....<br>GUCACCCAGGUUGGGGUGGAGUGGC<br>GGGCCCGGAGGUCUGGCCGAGGUCC<br><br>((((((((........))).)))))<br>AAAAAAAA........aaa.aaaaa	RBMX
**LINC02564** / **LINC01925**<br>Rank 7; LINC02564: Start 200, Stop 371, Strand +; LINC01925: Start 288, Stop 460, Strand +; Score 342; %id 39; P-Value 2.54E-02; E-Value 2.58E-02<br><br>.........1.........2.........3.........4.........5.........6<br>GGCCCCGCGCCCAGACCUGGC-GGCCGC-UGGCACCU-GACCCGCUGCAUGGGUCUCCAG<br>AGAGGCCUGUGCUCCGUGGGACAGGAGGCUAAGAGCGGAACAGGGCCCA-CUGUUGGAUG<br><br>(((((...(((((....))((.(((.((...((.(((.(((((.......)))))...))<br>KKKII...EEEAA....aaDD.DDD.BB...BB.BBB.BBBBB.......bbbbb...bb<br><br>.........7.........8.........9.........0.........1.........2<br>GGAGCUCGCUGCCCACCCGGCGCUGCAGGCUCGGCUCCCUCGUACACUCUCUGGCAAGCC<br>CUACUGAUCACCAGACUGUGGCCUUUAUACACAUCAACUUGCUUCAUCUCCACCAGAACA<br><br>)..))..)).(((.....)))))))).)))...((........(((....((........<br>b..bb..bb.CCC.....cccddddd.eee...HH........HHH....FF........<br><br>.........3.........4.........5.........6.........7.....<br>CAAGAAAGUCAGGGGCCUAUGUGAGCCAAAGAGGAGAGAAGGUGAUGCCUCAGCC<br>AAUGAAUGACAAAACCCGAUGAAAGACAAAG-CCAGAGUAAAUCCCUUCUCAUCU<br><br>..))..((........)).)))..))......)).(((..........))).)))<br>..ff..GG........gg.hhh..hh......ii.JJJ..........jjj.kkk	SFRS9<br>MBNL1<br>RBMX<br>SFRS13A<br>SFRS1
**LINC02564** / **LINC01925**<br>Rank 8; LINC02564: Start 531, Stop 588, Strand +; LINC01925: Start 281, Stop 334, Strand +; Score 294; %id 45; P-Value 2.94E-02; E-Value 2.99E-02<br><br>.........1.........2.........3.........4.........5........<br>ACCCAGGUUGGGGUGGAGUGGCACAGUCACAGCUCACUAUAACCUCAAGCUCCUGGGC<br>AGCCAUGAGAGGCCUGUGCUCCGUGGG-ACAGGAGGCUAAGAGCGGAA---CAGGGCC<br><br>.(((.((....((((..(((((.........)).)))..)).)).......)).))).<br>.AAA.AA....AAAA..AAAAA.........aa.aaa..aa.aa.......aa.aaa.	RBMX<br>SFRS1
**LINC02879** / **LINC01926**<br>Rank 1; LINC02879: Start 1272, Stop 1441, Strand +; LINC01926: Start 31, Stop 205, Strand +; Score 401; %id 38; P-Value 8.40E-02; E-Value 8.80E-02<br><br>.........1.........2.........3.........4.........5.........6<br>UGUGUU--GCUGGGACACAGGGGCAGGAAAUGGAUGCAUCCCUUCCCAUGCAC-GCCCA-<br>UCUCCAAGUUUCCAAGAAGGGAAGAUGGAGUGGAGGGCGCCCACCCUAUCUCCAGUGGAC<br><br>((((((..((((((........(((.((...((......))..))...)))....)))).<br>EEEEEE..CCAAAA........AAA.AA...AA......aa..aa...aaa....aaaa.<br><br>.........7.........8.........9.........0.........1.........2<br>AACAGCUCCUCAACUCAAUCUCAGCCCCAUGCCUCUUCUAUUCUUCUGUGAGGAAGUGGC<br>AUCCCCUGGGCUACUCCUCUCCUGAUGAGAAAAUGCUCACUUCAGCCAAGUGCAGAAUGG<br><br>......((.(((....((....((.............))....))...))).)).))(((<br>......BB.BBB....BB....BB.............bb....bb...bbb.bb.ccDDD<br><br>.........3.........4.........5.........6.........7.....<br>UUCUCAUGACCUAUCCGAG-AGCUCAGCACAUGUUAAGUUCUCACACAGGCUUAA<br>UCCCCUUUGUUUUCGUGGGUAUCAGUGGAGAAUGAGAUCCUUCAUAUGGAAUCUC<br><br>(.(((..((....)).))).)))).))))))..(((((..((......)))))))<br>D.DDD..DD....dd.ddd.dddd.eeeeee..FFFFF..FF......fffffff	MBNL1<br>EIF4B<br>RBMX<br>SFRS1

*web-based tool Foldalign v.2.9.0 [**S1**] (https://rth.dk/resources/foldalign) was used for local sequence alignment at maximum alignment length 200 nt.

web-based tool ‘linc2function’ [S2**] (https://bioinformaticslab.erc.monash.edu/linc2function) was used for prediction of proteins, interacting with motifs of lincRNAs: modes – ‘ANN’ and ‘full model’, species – ‘Homo sapiens’.
Abbreviations:
a2bp1, RNA binding fox-1 homolog 1;
EIF4B, eukaryotic translation initiation factor 4B;
ELAVL1, ELAV like RNA binding protein 1;
FUS, FUS RNA binding protein;
KHDRBS3, KH RNA binding domain containing, signal transduction associated 3;
KHSRP, KH-type splicing regulatory protein;
MBNL1, muscleblind like splicing regulator 1;
PABPC1, poly(A) binding protein cytoplasmic 1;
Pum2, pumilio RNA binding family member 2;
RBMX RNA binding motif protein X-linked;
SFRS1, serine and arginine rich splicing factor 1;
SFRS13A, serine and arginine rich splicing factor 10;
SFRS9, serine and arginine rich splicing factor 9.

References

[S1] Sundfeld D, Havgaard JH, de Melo AC, Gorodkin J. Foldalign 2.5: multithreaded implementation for pairwise structural RNA alignment. Bioinformatics. 2016;32(8):1238-40. doi: 10.1093/bioinformatics/btv748.

[S2] Ramakrishnaiah Y, Morris AP, Dhaliwal J, Philip M, Kuhlmann L, Tyagi S. Linc2function: A Comprehensive Pipeline and Webserver for Long Non-Coding RNA (lncRNA) Identification and Functional Predictions Using Deep Learning Approaches. Epigenomes. 2023;7(3):22. doi: 10.3390/epigenomes7030022
