# Supplementary material for: Long Intergenic Non-Coding RNAs of Human Chromosome 18: Focus on Cancers
Source: Biomedicines. 2024 Feb 28;12(3):544. doi: 10.3390/biomedicines12030544 (PMC10968066; doi:10.3390/biomedicines12030544)
Supplement: Supplementary file 1 [file biomedicines-12-00544-s001.zip › Table_S2_Ershov.pdf]

Table S2. Gene fusions involving linc-genes of chromosome 18

| Gene fusions | Cell line | Cancer associations of a perturbed gene by lincRNA | Cytogenetic location |
|---|---|---|---|
| *LINC00526--RAD54L* | CAL78 | *Breast, lung, bladder cancer | 18p11.31-1p34.1 |
| *LINC00526--IDH3B* | DND41 | Not found | 18p11.31-20p13 |
| *LINC00907--PIK3C3* | Y79 | *Esophageal, colon cancer | 18q12.3-18q12.3 |
| *LINC01416--LMAN1* | SNU1214 | Not found | 18q21.2-18q21.32 |
| *LINC01443--ESCO1* | NCIH2887 | Not found | 18p11.21-18q11.2 |
| *LINC01630--SMAD4* | SNU738, KP4 | *Pancreatic, colon, gastric cancer; **Tumor suppressor gene, hallmarks of cancer | 18q21.2-18q21.2 |
| *LINC01630--ELAC1* | MDAMB468, COLO678 | Not found | 18q21.2-18q21.2 |
| *LINC01899--MBP* | NIHOVCAR3, ONCODG1 | *Breast cancer, melanoma, gastric cancer | 18q22.3-18q23 |
| *LINC01902--PFN2* | SNU46 | *Breast cancer, sarcoma | 18q12.3-3q25.1 |
| *LINC01902--ANKUB1* | SNU46 | Not found | 18q12.3-3q25.1 |
| *LINC01915--RALBP1* | JVE015 | *Breast, lung, oral cancer | 18q11.2-18p11.22 |
| *LINC01915--SDHA* | CCSW1 | *Gliomas; **Tumor suppressor gene, hallmarks of cancer | 18q11.2-5p15.33 |
| *LINC01917--MBD2* | MIAPACA2 | *Breast, prostate, colon cancer | 18q21.2-18q21.2 |
| *LINC01926--ZNF532* | SKOV3 | Not found | 18q21.32-18q21.32 |
| *LINC01928--YES1* | GIMEN | *Lung, gastric cancer | 18p11.21-18p11.32 |
| *LINC02582--RCC1* | ICC15, LS180 | *Lung cancer | 18q22.3-1p35.3 |
| *LINC02582--SNHG3*# | ICC15, LS180 | Not found | 18q22.3-1p35.3 |
| *LINC02582--LRRC75A-AS1*# | PATU8902, G415 | Not found | 18q22.3-17p11.2 |
| *LINC02864--CCDC102B* | NCIH1963 | Not found | 18q22.3-18q22.1 |

*MalaCards: a human disease database [S1] (http://www.malacards.org/) was used to search for associations of a perturbed gene with cancers (direct or indirect involvement in cancer biology, cancer promotion or suppression, drug sensitivity or resistance); cancer associations were selected by MalaCards InFormaTion Scores (MIFTS) > 70.
**The Cancer Gene Census of the Catalogue of Somatic Mutations in Cancer (COSMIC) web portal [S2] (https://cancer.sanger.ac.uk/census).
#non-coding RNAs.

## References


[S1] Rappaport N, Twik M, Plaschkes I, Nudel R, Iny Stein T, Levitt J, Gershoni M, Morrey CP, Safran M, Lancet D. MalaCards: an amalgamated human disease compendium with diverse clinical and genetic annotation and structured search. Nucleic Acids Res. 2017;45(D1):D877-D887.

[S2] Sondka Z, Bamford S, Cole CG, Ward SA, Dunham I, Forbes SA. The COSMIC Cancer Gene Census: describing genetic dysfunction across all human cancers. Nat Rev Cancer. 2018;18(11):696-705.
