# Supplementary material for: Long Intergenic Non-Coding RNAs of Human Chromosome 18: Focus on Cancers
Source: Biomedicines. 2024 Feb 28;12(3):544. doi: 10.3390/biomedicines12030544 (PMC10968066; doi:10.3390/biomedicines12030544)
Supplement: Supplementary file 1 [file biomedicines-12-00544-s001.zip › Table_S3_Ershov.pdf]

Table S3. Frequency of copy number variations (amplifications and deletions*) of linc-genes of human chromosome 18 in cancers.

Gene/Cancer	BLCA	mBRCA	ESCA	HNSC	LUAD	LUSC	OV	PAAD	PRAD	SARC	STAD
LINC00305		5.1	3.9		3.3			3.7			3.4
LINC00470	4.1	20	5.5	3.1				4.6	3.3		4.1
LINC00526	3.2	4.8	3.3						3.3		4.8
LINC00667	3.2	4.8	3.3					5.5	3.3		
LINC00668	3.2	4.8	3,3					5.5			
LINC00907		16.4	3.9					23	9.8		4.6
LINC01254											7.5
LINC01255											5.4
LINC01387								5.5	3.3		4.1
LINC01416			3.3								
LINC01443								6.5			
LINC01444								6.5			
LINC01477								4.3			4.8
LINC01538		5.1	4.1	3.3				23			
LINC01539			4.4								4.8
LINC01541			3.9	4.6				24.8		3.2	
LINC01543						3.7	5.1	4.3			6.1
LINC01544			4.4	3.4							
LINC01630			5					24.8			3.6
LINC01915			4.4				3.1	8.7			3.4
LINC01917								3.8			
LINC01919			4.4					3.8			
LINC01924		5.1	4.1	3.6				23.9			
LINC01929			4.4								
LINC02582			4.1	4.6				24.8		3.1	
LINC02864			4.1	4.6				24.8		3.1	

*gene amplifications and deletions with frequencies > 3% are highlighted green and yellow colors, respectively; Data for analysis of copy number variations was retrieved from cBioPortal [S1].
Cancer cohorts used were the following:
BLCA - bladder urothelial carcinoma (TCGA cohort, PanCancer Atlas, n=411); mBRCA – metastatic breast invasive carcinoma (The Metastatic Breast Cancer Project (Provisional, December 2021), n=379); ESCA - esophageal carcinoma (TCGA cohort, PanCancer Atlas, n=182); HNSC - head and neck squamous cell carcinoma (TCGA cohort, PanCancer Atlas, n=523); LUAD - lung adenocarcinoma (Broad cohort, Cell 2012, n=183); LUSC - lung squamous cell carcinoma (TCGA cohort, PanCancer Atlas, n=487); OV - ovarian serous cystadenocarcinoma (TCGA cohort, PanCancer Atlas, n=584); PAAD - pancreatic adenocarcinoma (UTSW cohort, Nat Commun 2015, n=109; TCGA cohort, PanCancer Atlas, n=184); PRAD - prostate adenocarcinoma (Fred Hutchinson CRC, Nat Med 2016, n=154; Broad/Cornell cohort, Nat Genet 2012, n=112); SARC – sarcoma (TCGA cohort, PanCancer Atlas, n=255); STAD - stomach adenocarcinoma (TCGA cohort, PanCancer Atlas, n=440; (OncoSG cohort, 2018, n=147).

References

[S1] Cerami, E.; Gao, J.; Dogrusoz, U.; Gross, B.E.; Sumer, S.O.; Aksoy, B.A.; Jacobsen, A.; Byrne, C.J.; Heuer, M.L.; Larsson, E.; et al. The cBio Cancer Genomics Portal: An Open Platform for Exploring Multidimensional Cancer Genomics Data. Cancer Discov 2012, 2, 401–404, doi:10.1158/2159-8290.CD-12-0095.
