# Supplementary material for: Long Intergenic Non-Coding RNAs of Human Chromosome 18: Focus on Cancers
Source: Biomedicines. 2024 Feb 28;12(3):544. doi: 10.3390/biomedicines12030544 (PMC10968066; doi:10.3390/biomedicines12030544)
Supplement: Supplementary file 1 [file biomedicines-12-00544-s001.zip › Table_S5_Ershov.pdf]

Table S5. Gene Expression Omnibus (GEO) datasets used to search for differentially expressed linc-genes

GSE1379, GSE3292, GSE3578, GSE3744, GSE5764, GSE6919, GSE8401, GSE10245, GSE10270, GSE12452, GSE14407, GSE15471, GSE15490, GSE15781, GSE16515, GSE21261, GSE21510, GSE22035, GSE24514, GSE25136, GSE26511, GSE27831, GSE28702, GSE28703, GSE28996, GSE32323, GSE32962, GSE35144, GSE35809, GSE38376, GSE43346, GSE43837, GSE49515, GSE51373, GSE55945
