# Supplementary material for: Long Intergenic Non-Coding RNAs of Human Chromosome 18: Focus on Cancers
Source: Biomedicines. 2024 Feb 28;12(3):544. doi: 10.3390/biomedicines12030544 (PMC10968066; doi:10.3390/biomedicines12030544)
Supplement: Supplementary file 1 [file biomedicines-12-00544-s001.zip › Table_S6_Ershov.pdf]

Table S6. Condition-specific expression of linc-genes of human chromosome 18.

Gene	GEO dataset	log_2FC* (p-value)	Description
LINC00305	GSE51373 [S1]	-1.39 ($p<0.05$)	High-grade serous ovarian cancer resistant (n=12) vs sensitive (n=16) to platinum-based therapy.
LINC00305	GSE43837 [S2]	-0.96 ($p<0.1$)	Breast cancer with brain metastasis (n=19) vs non-metastatic breast cancer (n=19).
LINC00907	GSE43837 [S2]	-0.97 ($p<0.1$)	
LINC01387	GSE43837 [S2]	-1.03 ($p<0.05$)	
LINC00305	GSE14407 [S3]	0.96 ($p<0.1$)	Serous ovarian cancer epithelia (n=120) vs normal ovarian surface epithelia (n=12)
LINC00668	GSE14407 [S3]	1.37 ($p<0.05$)	
LINC01254	GSE14407 [S3]	1.34 ($p<0.005$)	
LINC01415	GSE14407 [S3]	1.35 ($p<0.05$)	
LINC01477	GSE14407 [S3]	1.47 ($p<0.05$)	
LINC01538	GSE14407 [S3]	0.91 ($p<0.05$)	
LINC01539	GSE14407 [S3]	1.84 ($p<0.001$)	
LINC01478	GSE15781 [S4]	0.86 ($p<0.05$)	Irradiated colorectal tumor tissue (n=19) vs non-irradiated tumor tissue (n=23)

*log_2FC - log_2FoldChange

References

[S1] Koti, M.; Gooding, R.J.; Nuin, P.; Haslehurst, A.; Crane, C.; Weberpals, J.; Childs, T.; Bryson, P.; Dharsee, M.; Evans, K.; et al. Identification of the IGF1/PI3K/NF κB/ERK Gene Signalling Networks Associated with Chemotherapy Resistance and Treatment Response in High-Grade Serous Epithelial Ovarian Cancer. BMC Cancer 2013, 13, 549, doi:10.1186/1471-2407-13-549.
[S2] McMullin, R.P.; Wittner, B.S.; Yang, C.; Denton-Schneider, B.R.; Hicks, D.; Singavarapu, R.; Moulis, S.; Lee, J.; Akbari, M.R.; Narod, S.A.; et al. A BRCA1 Deficient-like Signature Is Enriched in Breast Cancer Brain Metastases and Predicts DNA Damage-Induced Poly (ADP-Ribose) Polymerase Inhibitor Sensitivity. Breast Cancer Res 2014, 16, R25, doi:10.1186/bcr3625.
[S3] Bowen, N.J.; Walker, L.D.; Matyunina, L.V.; Logani, S.; Totten, K.A.; Benigno, B.B.; McDonald, J.F. Gene Expression Profiling Supports the Hypothesis That Human Ovarian Surface Epithelia Are Multipotent and Capable of Serving as Ovarian Cancer Initiating Cells. BMC Med Genomics 2009, 2, 71, doi:10.1186/1755-8794-2-71.
[S4] Snipstad, K.; Fenton, C.G.; Kjaeve, J.; Cui, G.; Anderssen, E.; Paulssen, R.H. New Specific Molecular Targets for Radio-Chemotherapy of Rectal Cancer. Mol Oncol 2010, 4, 52–64, doi:10.1016/j.molonc.2009.11.002.
