# Supplementary material for: Long Intergenic Non-Coding RNAs of Human Chromosome 18: Focus on Cancers
Source: Biomedicines. 2024 Feb 28;12(3):544. doi: 10.3390/biomedicines12030544 (PMC10968066; doi:10.3390/biomedicines12030544)
Supplement: Supplementary file 1 [file biomedicines-12-00544-s001.zip › Table_S7_Ershov.pdf]

Table S7. A list of GeneHancer* IDs for analysis of transcriptional regulators of linc-genes of human chromosome 18

Genes	GeneHancer ID	Region**	GH-score***	Total number of transcriptional regulators
LINC00470	GH18J001407	P, E	1.3	73
LINC00526	GH18J005625	P, E	2.1	81
LINC00667	GH18J005293	P, E	2.5	210
LINC00907	GH18J042740	E	1.2	20
LINC01255	GH18J011497	E	1.0	105
LINC1387	GH18J006557	E	1.2	14
LINC01415	GH18J055581	P, E	2.3	182
LINC01416	GH18J055892	P, E	1.1	31
LINC01478	GH18J044579	P, E	1.1	9
LINC01630	GH18J050877	P, E	2.0	164
LINC01882	GH18J012878	P, E	2.1	203
LINC01894	GH18J024983	P, E	1.3	8
LINC01895	GH18J003383	E	1.4	61
LINC01900	GH18J021980	P, E	1.4	74
LINC01901	GH18J039840	P, E	1.4	47
LINC01902	GH18J039840	P, E	1.4	47
LINC01909	GH18J070331	P, E	1.9	138
LINC01910	GH18J070375	P, E	1.9	144
LINC01915	GH18J024655	P, E	1.3	9
LINC01926	GH18J058804	E	1.6	20
LINC01929	GH18J055581	P, E	2.3	182
LINC02565	GH18J057426	P, E	2.5	146
LINC02837	GH18J049500	P, E	1.2	13
LINC02856	GH18J009641	E	1.6	43
LINC02864	GH18J073259	P, E	1.3	45
LINC02958	GH18J023964	E	1.1	52
LINC02979	GH18J012878	P, E	2.1	203
LINC03035	GH18J055096	E	1.0	42
LINC03110	GH18J058667	P, E	2	143

* GeneHancer (GH) Regulatory Elements database [S1] was used to search transcriptional regulators of linc-genes encoding lincRNAs of lincRNAs of human chromosome 18.
**P – promoter, E – enhancer.
**GH-score cut-off value ≥ 1

References

[S1] Fishilevich S, Nudel R, Rappaport N, Hadar R, Plaschkes I, Iny Stein T, Rosen N, Kohn A, Twik M, Safran M, Lancet D, Cohen D. GeneHancer: genome-wide integration of enhancers and target genes in GeneCards. Database (Oxford). 2017, 2017:bax028. doi: 10.1093/database/bax028.
