# Supplementary material for: Long Intergenic Non-Coding RNAs of Human Chromosome 18: Focus on Cancers
Source: Biomedicines. 2024 Feb 28;12(3):544. doi: 10.3390/biomedicines12030544 (PMC10968066; doi:10.3390/biomedicines12030544)
Supplement: Supplementary file 1 [file biomedicines-12-00544-s001.zip › Table_S9_Ershov.pdf]

Table S9. Gene expression patterns and functional terms for predicted transcriptional regulators (transcriptional factors) of linc-genes encoding lincRNAs of human chromosome 18

Group No	Transcriptional regulators	Gene expression pattern*	Reactome terms**
Group I	POLR2A, RAD21, PKNOX1, SP1, YY1, REST	Up-regulation in glioblastoma, low-grade glioma, pancreatic adenocarcinoma, stomach adenocarcinoma and thymoma	‘estrogen-dependent gene expression’, ‘activation of anterior HOX genes in hindbrain development during early embryogenesis’
Group II	CREM, NR2F1, SIN3A, ZNF335, CTCF, MAX, BHLHE40, L3MBTL2, TBP, MNT, EGR1, TAF1, ZBTB26, PRDM1, ATF1, NRF1, ZNF384, ZFHX2, SP7, ZBTB11, CTBP1, CEBPB, BCL6, ZGPAT, MEIS2, GABPA, NCOR1, DPF2, ZFP37, KLF1, ZXDB, ZBTB17, GLIS2, ZNF600, ZNF341, ZNF398, TCF12, EP300, SMAD4, CHD2, IKZF1, ZNF316, SMC3	Up-regulation in pancreatic adenocarcinoma and thymoma	‘estrogen-dependent gene expression’, ‘activation of anterior HOX genes in hindbrain development during early embryogenesis’, ‘FOXO-mediated transcription’, ‘circadian clock’

*tumor/normal tissue expression patterns of all genes in a group were analyzed using the GEPIA2 [S1] at following settings: $|\log_2 FC|$ cut-off value = 1 and p-value <0.05, respectively.
**functional enrichment analysis (Reactome terms) was performed using the PathwAX II web-based tool (adj. p-value <0.01). PathwAX II, a web-server for pathway annotation based on crosstalk derived through the FunCoup genome wide functional association networks [S2].

References

[S1] Tang Z, Kang B, Li C, Chen T, Zhang Z. GEPIA2: an enhanced web server for large-scale expression profiling and interactive analysis. Nucleic Acids Res. 2019;47(W1):W556-W560.

[S2] Ogris C, Castresana-Aguirre M, Sonnhammer ELL. PathwAX II: network-based pathway analysis with interactive visualization of network crosstalk. Bioinformatics. 2022;38(9):2659-2660.
